# Erythropoietin alleviates syndrome-associated intellectual disability and autism-like behavior in Zbtb20-haploinsufficient Primrose syndrome mouse model

**DOI:** 10.1172/jci.insight.200021

**Published:** 2026-02-23

**Authors:** Martin Hindermann, Justus B.H. Wilke, Yasmina Curto, Stefan N. Oline, Vinicius Daguano Gastaldi, Umer Javed Butt, Rakshit Dadarwal, Umut Çakır, Anja Ronnenberg, Kurt Hammerschmidt, Susann Boretius, Anastassia Stoykova, Anton B. Tonchev, Klaus-Armin Nave, Manvendra Singh, Hannelore Ehrenreich

**Affiliations:** 1 Clinical Neuroscience, Max Planck Institute for Multidisciplinary Sciences, City Campus, Göttingen, Germany.; 2 Georg-August-University, Göttingen, Germany.; 3 Princeton Neuroscience Institute, Princeton University, Princeton, New Jersey, USA.; 4 Department of Neurogenetics, Max Planck Institute for Multidisciplinary Sciences, City Campus, Göttingen, Germany.; 5 Experimental Medicine, Central Institute of Mental Health, Medical Faculty Mannheim, Heidelberg University, Heidelberg, Germany.; 6 Functional Imaging Laboratory, and; 7 Cognitive Ethology, German Primate Center, Göttingen, Germany.; 8 Rhythms - Beating Cilia and Ticking Clocks Group, Max Planck Institute for Multidisciplinary Sciences, Fassberg Campus, Göttingen, Germany.; 9 Anatomy and Cell Biology, Faculty of Medicine & Stem Cell Biology, Research Institute, Medical University-Varna, Varna, Bulgaria.

**Keywords:** Clinical Research, Neuroscience, Behavior, Bioinformatics, Outcomes research

## Abstract

Among the known genetic causes of syndromic autism spectrum disorders (ASDs) are transcription factor deficiencies. In this regard, haploinsufficiency of the zinc finger and broad complex, tramtrack, bric and brac domain–containing protein 20 (*ZBTB20*) leads to a prototypical clinical picture, referred to as Primrose syndrome, comprising severe ASD symptoms together with intellectual disability. Here, we present a comprehensive behavioral and phenotypical characterization of *Zbtb20^+/–^* mice, a construct valid model of this thus far untreatable human condition. *Zbtb20^+/–^* mice exhibited diminished sociability, reduced vocalization, distinct repetitive behaviors, impaired cognitive flexibility, hyperactivity, and hypoalgesia. Magnetic resonance imaging revealed increased volumes of hippocampus, cerebellum, brain matter, and whole brain, confirmed by postmortem brain weight measurements. Due to our previous observation of enhanced ZBTB20 expression in CA1 pyramidal neurons upon recombinant human erythropoietin (rhEPO) injections, we anticipated a mitigating effect through rhEPO treatment of *Zbtb20* deficiency/Primrose syndrome. Indeed, after 3 weeks of alternate-day rhEPO injections, a remarkable improvement in the behavioral phenotype was observed. Our results highlight rhEPO as promising treatment for Primrose syndrome.

## Abstract 2

Mice with Primrose syndrome traits, comparable to the human condition, improve after erythropoietin treatment, suggesting a potential therapy for these autism-related symptoms and intellectual disability.

## Introduction

Autism spectrum disorder (ASD), with a prevalence of approximately 1% across cultures, is characterized by 3 primary behavioral domains: impaired sociability, reduced communication, and repetitive behaviors (1). Additional symptoms are decreased cognitive flexibility (2), hyperactivity (3, 4), and pain hyposensitivity (5, 6). Despite multifactorial causes, including dysfunctions of chromatin and transcription factors, the final common pathway of ASD converges at the synapse (7). A transcription factor gene associated with ASD is the zinc finger and broad complex, tramtrack, bric and brac domain–containing protein 20 (*ZBTB20*), located at chromosome 3q13.31. The encoded protein is particularly abundant in the hippocampus (8). ZBTB20 contains 5 C2H2 zinc finger domains (ZnFI–ZnFV) and an N-terminal broad complex, tramtrack, bric and brac (BTB) domain, which facilitates interaction with the DNA (9).

ZBTB20 plays a crucial role in glucose metabolism, postnatal growth, neurogenesis (9–11), and the specification of the medial pallium, which forms the hippocampus (8, 11–14). Furthermore, ZBTB20 is instrumental for maturation of hippocampal cornu ammonis 1 (CA1) neurons, the development of dendritic and synaptic structures, olfactory bulb neurogenesis, and the generation of neuronal layers in the developing cortex (15–18).

In humans, a heterozygous pathogenic variant of *ZBTB20* causes the extremely rare, autosomal dominant Primrose syndrome (19, 20), which is characterized by macrocephaly with developmental delay, intellectual disability, behavioral abnormalities, typical facial phenotypes, altered glucose metabolism, hypotonia, agenesis of the corpus callosum, hearing loss, ocular anomalies, cryptorchidism, and calcification of the ear cartilage (20–32). Behavioral deviations include attention deficit/hyperactivity disorder (ADHD), self-injurious behavior, sleep disturbances, tics, stereotypies, and ASD (23, 33–35).

Regarding animal models, only a few studies have reported on a limited number of behavioral tests performed with *Zbtb20^+/–^* mice. These tests examined visual capability, hippocampus-dependent learning and memory, anxiety, exploration, nociception, and circadian rhythm (12, 15, 36–38). Thus far, however, a comprehensive behavioral characterization or any kind of treatment approach are completely lacking.

The glycoprotein erythropoietin (EPO), named after its initially discovered effects on the hematopoietic system, has over the last decades attracted attention due to its neuroprotective, neuroregenerative, and cognition-enhancing properties. Moreover, EPO was just found to be a potent driver of neurodifferentiation (39). Recently, we detected an increase in ZBTB20 expression within the hippocampal CA1 region in mice following recombinant human EPO (rhEPO) treatment (40).

We thus hypothesized that rhEPO application might augment the residual expression of ZBTB20 in heterozygous mice, and thereby mitigate the overall phenotype. To test this hypothesis, we conducted an in-depth behavioral and phenotypical characterization of *Zbtb20^+/–^* mice following 3 weeks of rhEPO application. The results revealed multifaceted improvements, suggesting rhEPO as promising therapeutic approach for Primrose syndrome.

## Results

### Synopsis

**Figure 1:**
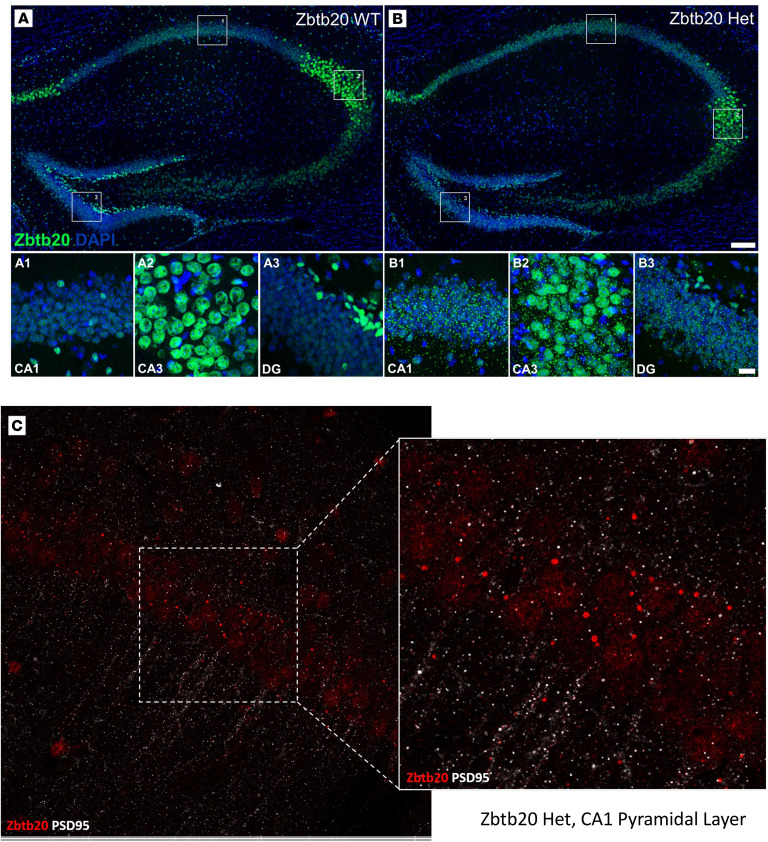
Histology. (**A** and **B**) Representative images of ZBTB20 expression (green) in the hippocampal region of *Zbtb20^+/+^* (WT) and *Zbtb20^+/–^* (Het) mice. A1–A3 and B1–B3: higher magnifications of the outlined boxes in **A** and **B**, representing CA1, CA3, and DG areas. Scale bars: 100 μm for **A** and **B**; 20 μm for higher magnifications. (**C**) CA1 pyramidal layer of *Zbtb20^+/–^* (Het) including higher magnification reveals no double-labeling of Zbtb20 with PSD95.

**Figure 2:**
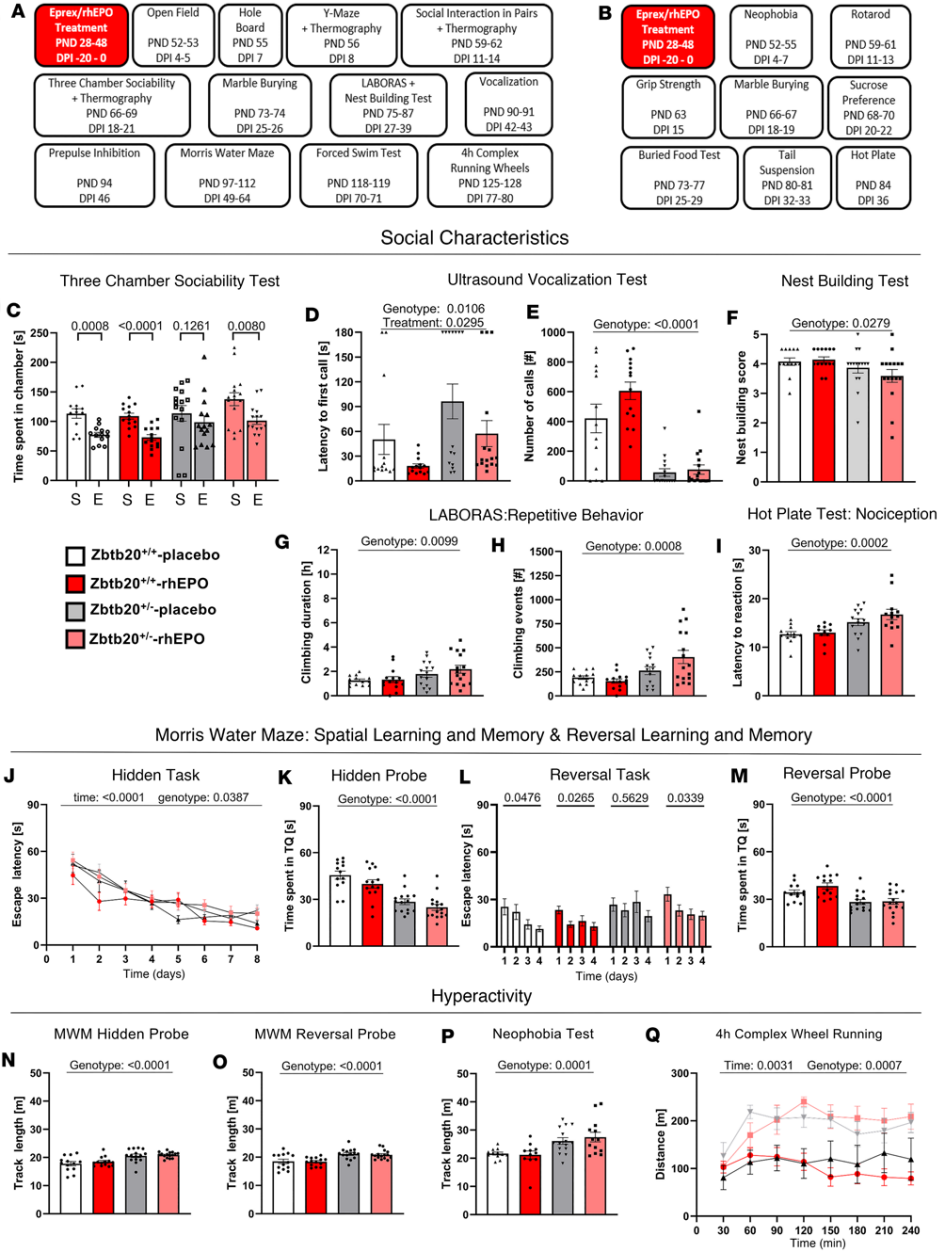
Experimental outline and *Zbtb20^+/–^* impairments in ASD domains. (**A** and **B**) Graphical experimental outlines for main (**A**) and additional (**B**) cohort of male *Zbtb20^+/–^* mice and their *Zbtb20^+/+^* littermates, used as control. After 11 consecutive placebo resp. rhEPO injections, behavior experiments were performed. PND, postnatal day; DPI, days post injection. (**C**–**F**) Assessment of social characteristics shows impaired social preference in the 3-chamber sociability test (**C**), since placebo-treated *Zbtb20^+/–^* mice did not distinguish between stimulus mouse (S) and empty cage (E). This impairment is rescued by rhEPO treatment (*t* test, mean ± SEM). Communication assessed with ultrasound vocalization (**D** and **E**) shows that *Zbtb20^+/–^* mice exhibit an increased latency to first call (**D**) with improvement after rhEPO treatment and a reduced number of calls (**E**) compared with controls (2-way ANOVA with Bonferroni’s post hoc test, mean ± SEM). Nest building (**F**) results in a reduced score in *Zbtb20^+/–^* mice regarding quality of constructed nests overnight (2-way ANOVA with Bonferroni’s post hoc test, mean ± SEM). (**G** and **H**) Repetitive behavior was assessed in home cages via LABORAS and shows increased duration (**G**) and events (**H**) of climbing in *Zbtb20^+/–^* mice (2-way ANOVA with Bonferroni’s post hoc test, mean ± SEM). (**I**) Hot plate test reveals hyposensitivity toward heat-mediated nociception in *Zbtb20^+/–^* mice, with an increased latency of the first reaction to heat perception (2-way ANOVA with Bonferroni’s post hoc test, mean ± SEM). (**J**–**M**) Spatial learning, memory, as well as reversal learning and memory were assessed in a Morris water maze. Hidden training over 8 days shows time and genotype effect in escape latency (**J**, 3-way ANOVA with Bonferroni’s post hoc test, mean ± SEM). Probe trial for hidden learning task (**K**) reveals impaired spatial memory in *Zbtb20^+/–^* mice compared with controls, shown by reduced time spent in target quadrant (TQ, 2-way ANOVA with Bonferroni’s post hoc test, mean ± SEM). Reversal hidden training over 4 days (**L**) displays impaired spatial reversal learning in placebo-treated *Zbtb20^+/–^* mice and a rescue effect after rhEPO treatment, indicated by escape latency to platform (2-way ANOVA with Bonferroni’s post hoc test, mean ± SEM). Reversal probe trial (**M**) demonstrates impaired reversal spatial memory in *Zbtb20^+/–^* mice, indicated by the time spent in target quadrant (2-way ANOVA with Bonferroni’s post hoc test, mean ± SEM). (**N**–**Q**) Hyperactivity of *Zbtb20^+/–^* mice was assessed in probe trials for both hidden (**N**) and reversal (**O**) tasks of the Morris water maze, neophobia test (**P**) (all 2-way ANOVA with Bonferroni’s post hoc test, mean ± SEM), and 4-hour-complex wheel running (**Q**, 3-way ANOVA with Bonferroni’s post hoc test, mean ± SEM).

**Figure 3:**
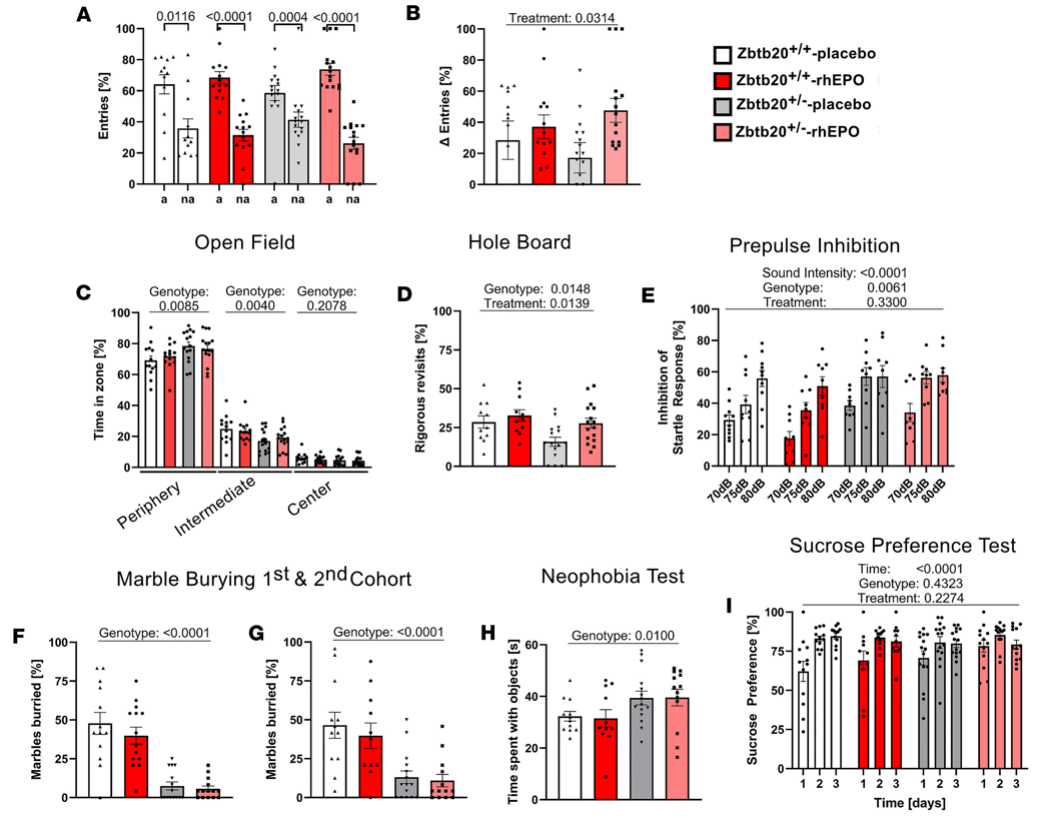
Working memory, exploration, pre-pulse inhibition, obsessive-compulsive readouts, and related control testing in *Zbtb20^+/–^* mice. (**A** and **B**) Working memory was assessed in the Y-maze test and shows significantly more alternating (a) than non-alternating (na) entries in all 4 groups (**A**, *t* test, mean ± SEM). Comparing Δ of entries (**B**) reveals further improved preference of alternating entries in rhEPO-treated *Zbtb20^+/–^* mice compared with placebo-treated controls (2-way ANOVA with Bonferroni’s post hoc test, mean ± SEM). (**C**) Placebo-treated *Zbtb20^+/–^* mice show increased time spent in peripheral zone and reduced time spent in intermediate zone in the open field test (2-way ANOVA with Bonferroni’s post hoc test, mean ± SEM). (**D**) Explorative behavior was also assessed in hole board test, revealing reduced rigorous revisits of placebo-treated *Zbtb20^+/–^* mice and a rescue effect upon rhEPO treatment (2-way ANOVA with Bonferroni’s post hoc test, mean ± SEM). (**E**) Prepulse inhibition test shows a genotype effect, namely increased inhibition of startle response in both placebo- and rhEPO-treated *Zbtb20^+/–^* mice (3-way ANOVA with Bonferroni’s post hoc test, mean ± SEM). (**F** and **G**) Marble burying test resulted in reduced numbers of buried marbles in the main (**F**) and, as control for this unexpected result, in an additional (**G**) cohort (2-way ANOVA with Bonferroni’s post hoc test, mean ± SEM). (**H**) Neophobia test shows increased interaction time with objects in placebo-treated *Zbtb20^+/–^* mice and no treatment effect (2-way ANOVA with Bonferroni’s post hoc test, mean ± SEM). (**I**) Sucrose preference test demonstrates absence of anhedonia-like (depressive) behavior in placebo- and rhEPO-treated *Zbtb20^+/–^* mice (3-way ANOVA with Bonferroni’s post hoc test, mean ± SEM).

**Figure 4:**
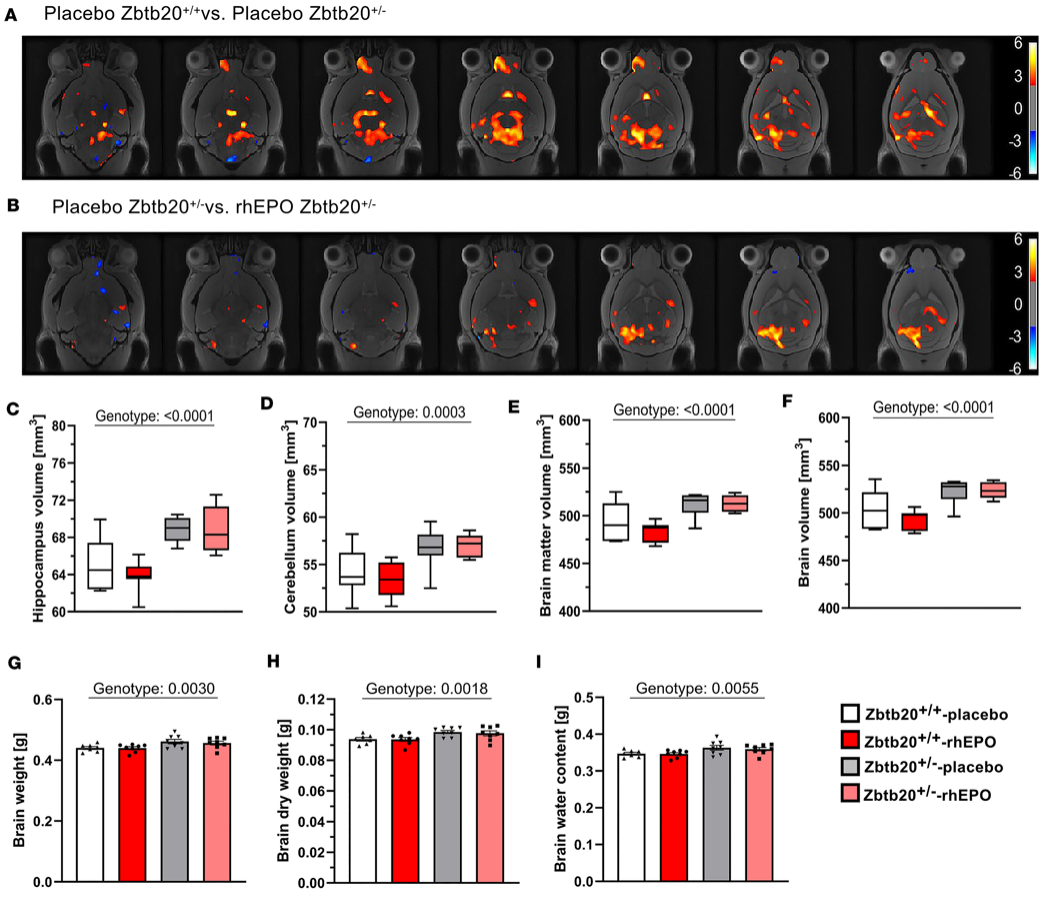
Magnetic resonance imaging. (**A** and **B**) Voxel-wise Jacobian determinants map indicates increased (red/yellow) or decreased (blue) volume changes in both placebo-treated *Zbtb20^+/+^* versus *Zbtb20^+/–^* (**A**) and placebo- versus rhEPO-treated *Zbtb20^+/–^* (**B**) mice. (**C**–**F**) Volumetric analyses of specific brain regions show in both placebo- versus rhEPO-treated *Zbtb20^+/–^* mice an increased volume of hippocampus (**C**), cerebellum (**D**), brain matter (**E**), and whole brain (**F**). (**G**–**I**) Weight assessment of whole brains. (**G**) Immediately after extraction, brains of both placebo- and rhEPO-treated *Zbtb20^+/–^* mice display increased weight compared with controls. (**H**) Likewise, after 48 hours of lyophilization, brain dry weight of both placebo- and rhEPO-treated *Zbtb20^+/–^* mice was enhanced. (**I**) Also, calculated water content was augmented in both placebo- and rhEPO-treated *Zbtb20^+/–^* mice (2-way ANOVA with Bonferroni’s post hoc test; mean ± SEM).

**Figure 5:**
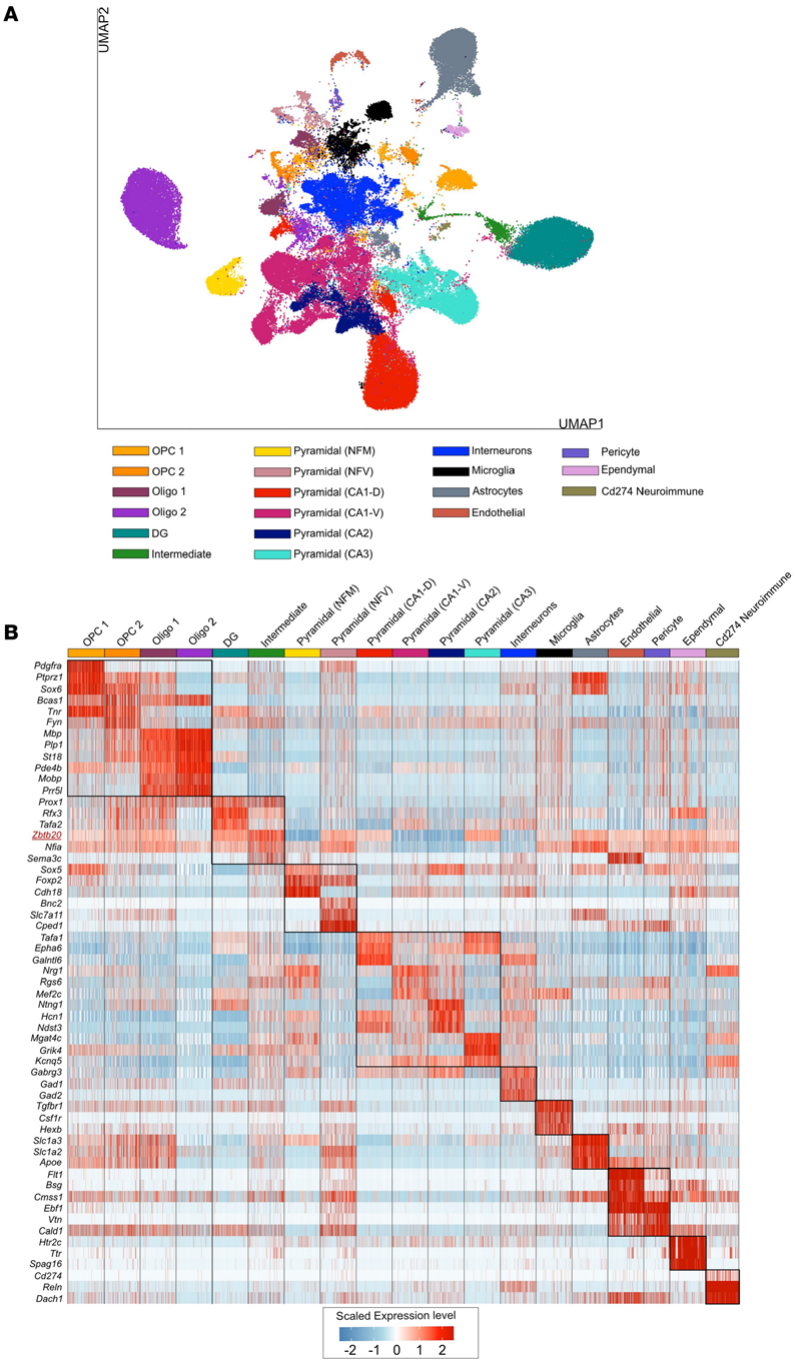
Classification and molecular markers of distinct lineages in the mouse hippocampus. (**A**) Two-dimensional Uniform Manifold Approximation Plot (UMAP) resolving the 19 distinct lineages of mouse hippocampus (integrated snRNA-seq derived from rhEPO [*N* = 6] and placebo [*N* = 6] mice). Cluster identity was assigned manually by retrieving known marker genes. The major cell types are annotated on the plot. Each dot represents a nucleus. (**B**) Heatmap illustrating the relative expression of the top 6 key genes defining the identity of each cluster in **A**. The top differentially expressed genes (DEGs) were calculated using Wilcoxon’s rank-sum test for each identified cell type. The color ranges from low (blue) to high (red) expression of marker genes (rows) in the single nuclei (columns). A full list of DEGs in each cell type is given in Supplemental Table 3.

**Figure 6:**
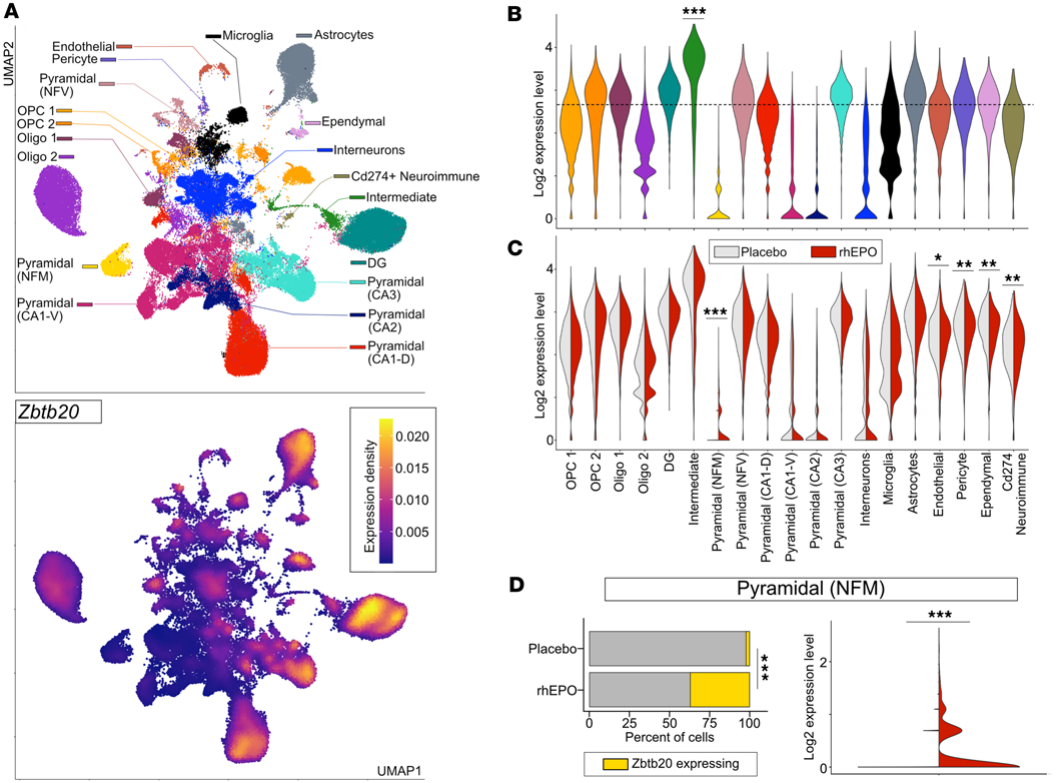
rhEPO induces *Zbtb20* expression in the newly formed migratory pyramidal lineage. (**A**) Feature plot (lower panel) based on UMAP (upper panel) demonstrating the wider pattern of *Zbtb20* levels, denoted as the density of relative expression. Midnight blue area represents lower expression whereas the nuclei under the golden area exhibit higher expression of *Zbtb20*. Note: *Zbtb20* expression in multiple locations suggests the intricacy of *Zbtb20* regulation in the hippocampus needs to be unveiled in future studies. (**B**) Violin plot showing the expression distribution of *Zbtb20* across the lineages coded by colors as denoted in **A**. Stars signify the adjusted *P* values obtained by Benjamini-Hochberg (BH) correction followed by Wilcoxon’s rank-sum test (1-way), comparing *Zbtb20* expression in intermediate cell versus the remaining lineages (***P < 0.001, BH test). (**C**) Grouped violin plot visualizes the comparison of expression dynamics of *Zbtb20* between placebo (light gray) and rhEPO (red) across lineages; level of significance is calculated similarly as shown in previous plot, except here, the comparison is made between placebo and rhEPO samples (*P < 0.05, **P < 0.01, ***P < 0.001, BH test). (**D**) Left panel: Stacked bar plot of pyramidal (NFM) cell type illustrating the percentage of cells expressing *Zbtb20* (gold) and the remaining cells (gray) separately in placebo- and rhEPO-treated samples. Asterisks denote the *P* value of difference in cells expressing *Zbtb20* between rhEPO- and placebo-treated samples (***P < 0.001, 2-sided Fisher’s exact test). Right panel: Violin plot quantifying the density and distribution of *Zbtb20* expression in pyramidal (NFM) cell type from rhEPO- and placebo-treated samples in pairwise manner (***P < 0.001, Wilcoxon’s rank-sum test, 1-way).

This study was designed with the objective of performing an exhaustive behavioral characterization of *Zbtb20^+/–^* mice, a construct-valid model of the thus far untreatable human Primrose syndrome. Based on our previous observations of EPO specifically targeting ZBTB20 expression, we incorporated a 3-week treatment trial using rhEPO. The findings unveiled impairments across all domains that are typically associated with ASD and intellectual disability. Importantly, improvements in several pathological parameters were observed following rhEPO administration (Figures 1–6, Supplemental Figures 1–3, and Supplemental Tables 1–4; supplemental material available online with this article; https://doi.org/10.1172/jci.insight.200021DS1), thus potentially offering a promising therapeutic approach to Primrose syndrome.

#### Immunohistochemistry illustrates ZBTB20 expression in the hippocampus of Zbtb20^+/+^ versus Zbtb20^+/–^ mice.

Immunohistochemical images of ZBTB20 expression in the hippocampal regions CA1, CA3, and dentate gyrus (DG) of *Zbtb20^+/+^* (WT) and *Zbtb20^+/–^* mice document the expectedly reduced expression/green signal in *Zbtb20^+/–^* mice (Figure 1, A and B). Interestingly, but still unclear in nature, are the small green puncta distributed all over the hippocampus exclusively of *Zbtb20^+/–^* mice. Exploratory costaining for PSD95 excluded a simple colocalization of these puncta with synaptic structures (Figure 1C). Furthermore, they were mainly located outside the nucleus and were not associated with astrocytic (GFAP) nor with neuronal processes (MAP2, data not shown).

#### Hematopoietic cell composition in lymphoid organs is largely unaffected in Zbtb20^+/–^ mice.

While ZBTB20 has been most extensively studied in the central nervous system, accumulating evidence, derived from cell-type-specific *Zbtb20*-knockout mice, highlights important roles for this transcription factor in several immunological processes (41–44). To assess whether ZBTB20 haploinsufficiency impacts the hematopoietic cell composition, we performed high-parameter flow cytometric immunophenotyping to quantify 6 major immune cell types, namely B cells, CD4^+^ and CD8^+^ T cells, NK cells, monocytes, and granulocytes, as well as 19 functionally distinct immune cell subsets across several lymphoid compartments, including blood, bone marrow, lymph nodes, and spleen (Supplemental Figure 3A and Supplemental Table 4). Qualitative comparison of immune cell clusters expectedly revealed major differences between the immune cell compartments but no gross abnormalities in *Zbtb20**^+/–^*** versus WT mice (Supplemental Figure 3B). Subsequent quantitative permutational multivariate analysis of variance (PERMANOVA) comparison of the immune cell compositions within each lymphoid compartment confirmed the absence of major changes in the immune cell composition of *Zbtb20^+/–^* mice (all *P* > 0.05, Supplemental Figure 3C). Detailed analysis of individual immune cell populations revealed a subtle but significant increase in CD4^+^ T cells in lymph nodes of *Zbtb20^+/–^* mice (FDR = 0.023, fold change = 1.1) as well as an increase in splenic NK cells (FDR = 0.0275, fold change = 1.2). No significant differences between *Zbtb20^+/–^* and WT mice were observed for distinct subsets of NK cells, T cells, B cells, and monocytes (all FDR > 0.05, Supplemental Table 4).

*Zbtb20^+/–^* males display impairments in all primary domains of ASD-like behavior. An in-depth behavioral characterization of *Zbtb20^+/–^* males (Figure 2, A and B) revealed impairments across all primary domains of an ASD phenotype. The compromised social interaction became evident in the 3-chamber sociability test. Here, placebo-treated *Zbtb20^+/–^* males failed to differentiate between the chamber housing a stimulus mouse and the opposite chamber containing an empty cage (*P* = 0.1261). In contrast, both placebo- and rhEPO-treated WT and rhEPO-treated *Zbtb20^+/–^* demonstrated a preference for the chamber of the stimulus mouse over the empty chamber (*P* = 0.0008, *P* < 0.0001, *P* = 0.0080; Figure 2C and Supplemental Table 1).

Communication deficits were discernible in the vocalization test. Both placebo- and rhEPO-treated *Zbtb20^+/–^* males exhibited an increased latency to their first vocalization upon exposure to an anesthetized female stimulus mouse (genotype effect: *P* = 0.0106). Interestingly, rhEPO treatment of both WT and *Zbtb20^+/–^* mice reduced the latency to their first vocalization (treatment effect: *P* = 0.0295). Furthermore, both *Zbtb20^+/–^* groups emitted significantly fewer calls toward the stimulus mice throughout the 3-minute recording, compared with their respective controls (genotype effect: *P* < 0.0001, Figure 2, D and E, and Supplemental Table 1). In nest building, another social skill test, *Zbtb20^+/–^* males constructed nests of inferior quality compared with WT controls and rhEPO had no effect (genotype effect: *P* = 0.0279, Figure 2F and Supplemental Table 1).

This result was also mirrored in their female littermates, where both *Zbtb20^+/–^* groups scored lower in nest building compared with their respective WT controls (genotype effect: *P* < 0.0001, Supplemental Figure 1A and Supplemental Table 2). Intriguingly, the diminished quality of nests constructed by both *Zbtb20^+/–^* sexes was already obvious in their home cages, an observation that experimenters became aware of only after being unblinded at project conclusion.

Assessment by the Laboratory Animal Behavior Observation Registration and Analysis System (LABORAS) revealed enhanced repetitive behavior, with *Zbtb20^+/–^* males displaying increased duration of climbing and number of climbing events (genotype effects: *P* = 0.0099 and *P* = 0.0008, respectively; Figure 2, G and H, and Supplemental Table 1) compared with WT controls. In females, the result was similar (genotype effects: *P* = 0.0145 and *P* = 0.0002, respectively; Supplemental Figure 1, B and C, and Supplemental Table 2).

#### Additional abnormalities of Zbtb20^+/–^ males linked to ASD-like behavior.

In the hot-plate test, an augmented tolerance for heat-mediated pain became aware. Both placebo- and rhEPO-treated *Zbtb20^+/–^* males exhibited a heightened latency to their initial visible reaction toward the stimulus (genotype effect: *P* = 0.0002, Figure 2I and Supplemental Table 1). In females, placebo-treated *Zbtb20^+/–^* mice also demonstrated an increased latency compared with their WT control (genotype effect: *P* = 0.0003). However, unlike in males, female rhEPO-treated *Zbtb20^+/–^* mice displayed a latency to the first reaction that was on par with WT controls and therefore lower than placebo-treated *Zbtb20^+/–^* females (treatment effect: *P* = 0.0093, Supplemental Figure 1D and Supplemental Table 2).

The Morris water maze was employed to evaluate spatial learning and memory. *Zbtb20^+/–^* males exhibited visual abilities equivalent to their WT controls (Supplemental Table 1). A genotype effect was observed during 8 days of hidden training (*P* = 0.0387, Figure 2J). During both hidden and reversal probe trials, *Zbtb20^+/–^* males spent significantly less time in the target quadrant that previously held the escape platform (both genotype effects: *P* < 0.0001, Figure 2, K–M, and Supplemental Table 1). Between both probe trials, 4 days of reversal training were conducted, revealing that placebo-treated *Zbtb20^+/–^* males were the only group not showing significant improvement in escape latency over time (*P* = 0.5629). In contrast, both placebo- and rhEPO-treated WT males (*P* = 0.0476, *P* = 0.0265) and rhEPO-treated *Zbtb20^+/–^* males improved significantly (*P* = 0.0339, Figure 2L and Supplemental Table 1). In females, a phenotype was only observable during both probe trials of hidden and reversal task where *Zbtb20^+/–^* mice spent less time in the target quadrant compared with controls, independent of treatment (genotype effects: *P* = 0.0153 and *P* = 0.0040, respectively; Supplemental Figure 1, F–H, and Supplemental Table 2). Both male and female *Zbtb20^+/–^* mice displayed increased locomotion across a variety of tests. In males, hidden and reversal probe trials (genotype effects: *P* < 0.0001), the neophobia test (genotype effect: *P* = 0.0001), and 4-hour complex wheel running (genotype effect: *P* = 0.0007, Figure 2, N–Q, and Supplemental Table 1) revealed significantly increased track lengths. In females, *Zbtb20^+/–^* mice demonstrated increased locomotion in the open field test (genotype effect: *P* = 0.0006), both hidden and reversal probe trials (genotype effects: *P* = 0.0009 and *P* = 0.0004, respectively), and in the LABORAS test (genotype effect: *P* = 0.0076, Supplemental Figure 1, L–O, and Supplemental Table 2).

#### Zbtb20^+/–^ phenotypes in working memory, sensorimotor gating, and motivation.

The Y-maze test was employed to assess working memory by contrasting the frequency of alternating and non-alternating entries into the maze arms. Despite all 4 male groups performing significantly more alternating than non-alternating entries, a comparison of the deltas from these 2 readouts revealed that placebo-treated *Zbtb20^+/–^* mice had the lowest delta of all groups, significantly lower than rhEPO-treated *Zbtb20^+/–^* males, thus demonstrating a treatment effect (*P* = 0.0314, Figure 3, A and B, and Supplemental Table 1). The open field and hole board tests were utilized to assess exploratory behavior and the motivation to engage in such. *Zbtb20^+/–^* males spent more time within the periphery and less time within the intermediate zone in the open field compared with their WT controls (genotype effects: *P* = 0.0085 and *P* = 0.0040, respectively; Figure 3C and Supplemental Table 1). The hole board experiment revealed a reduction in revisits to the most recently visited hole in placebo-treated *Zbtb20^+/–^* males compared with their WT controls (genotype effect: *P* = 0.0148). Additionally, an increase toward control levels was observed in rhEPO-treated *Zbtb20^+/–^* males (treatment effect: *P* = 0.0139, Figure 3D and Supplemental Table 1). A similar result was obtained in the female groups, where placebo-treated *Zbtb20^+/–^* mice showed fewer revisits compared with WT control, and rhEPO-treated *Zbtb20^+/–^* females exhibited a tendency of increasing the number of revisits to control level (genotype effect: *P* = 0.0982, treatment effect: *P* = 0.0526, Supplemental Figure 1I and Supplemental Table 2).

In the prepulse inhibition paradigm, which was used to evaluate sensorimotor gating, significant distinctions were observed among the various sound intensities in all 4 groups (sound intensity: *P* < 0.0001). Moreover, a genotype effect was identified when comparing both placebo- and rhEPO-treated *Zbtb20^+/–^* males with their respective WT controls (*P* = 0.0061, Figure 3E and Supplemental Table 1). Interestingly, in the marble burying test, both cohorts of placebo- and rhEPO-treated *Zbtb20^+/–^* males buried fewer marbles compared with their respective WT controls (both genotype effects: *P* < 0.0001, Figure 3, F and G, and Supplemental Table 1). A similar result was observed in females, where placebo- and rhEPO-treated *Zbtb20^+/–^* mice of both cohorts buried significantly fewer marbles compared with their respective controls (genotype effects: *P* < 0.0001 and *P* = 0.0034, respectively; Supplemental Figure 1, J and K, and Supplemental Table 2). To further investigate potential causes for these unusual results, additional tests for the assessment of neophobia, depression-like behavior, and anhedonia were conducted in a second experimental cohort of this project. In neophobia testing, both male and female *Zbtb20^+/–^* mice did not show decreased time spent with objects compared with their controls (Supplemental Tables 1 and 2), but *Zbtb20^+/–^* males exhibited an increased time spent with novel objects (genotype effect: *P* = 0.0100, Figure 3H and Supplemental Table 1). For depression-like behavior, tail suspension and forced swim tests were performed but did not reveal an overall increased time of immobility in *Zbtb20^+/–^* males or females (Supplemental Tables 1 and 2). For the assessment of anhedonia, the sucrose preference test was conducted, showing all 4 groups with significantly increasing preference towards sucrose over the experimental time of 3 days (time effect: *P* < 0.0001, Figure 3I and Supplemental Table 1). Females also revealed no differences across groups in this test (Supplemental Table 2).

#### MRI uncovered structural differences for brain regions.

A voxel-wise Jacobian determinants map was created to provide a visual representation of volumetric changes in the mouse brain (Figure 4, A and B). This map intensely delineates the increasing (yellow/red) and decreasing (blue/white) volumes when comparing both placebo-treated groups (Figure 4A) and both placebo- and rhEPO-treated *Zbtb20^+/–^* groups (Figure 4B). This comprehensive overview, combined with findings reported from previous studies and the technical prowess of this MRI technique, facilitated the volume analysis of an array of brain regions.

In males, both *Zbtb20^+/–^* groups exhibited an increased volume of hippocampus (*P* < 0.0001), cerebellum (*P* = 0.0003), brain matter (*P* < 0.0001), and whole brain (*P* < 0.0001, Figure 4, C–F, and Supplemental Table 1). The brain weight immediately after extraction, dry weight, and computed water content corroborated these findings (genotype effects: *P* = 0.0030, *P* = 0.0018, *P* = 0.0055; Figure 4, G–I, and Supplemental Table 1). The brain regions of the olfactory bulb, thalamus, corpus callosum, and ventricles unveiled no volumetric differences (Supplemental Table 1).

The volumetric analysis in females disclosed a diminished olfactory bulb volume in both placebo- and rhEPO-treated *Zbtb20^+/–^* mice (genotype effect: *P* < 0.0001, Supplemental Figure 2A and Supplemental Table 2). While the volumes of both hippocampus and cerebellum were increased in placebo-treated *Zbtb20^+/–^* mice (genotype effects: *P* = 0.0060 and *P* = 0.0047, respectively), rhEPO treatment led to a reduction in the volume (treatment effects: *P* = 0.0293 and *P* = 0.0156, respectively; Supplemental Figure 2, B–D, and Supplemental Table 2). Volumetric changes of the ventricles revealed a treatment-dependent reduction (treatment effect: *P* = 0.0007, Supplemental Figure 2C and Supplemental Table 2).

#### snRNA-seq discovered upregulation of ZBTB20 in pyramidal neurons on rhEPO.

To begin examining the ZBTB20 expression in the mouse hippocampus, we leveraged the recently published single-nuclei RNA-seq (snRNA-seq) dataset encompassing a broad range of neuronal and non-neuronal cell types (39). By refining these lineages deeper using the preset resolution of 0.4 from the Seurat package, we profiled the transcriptome of 19 distinct hippocampal lineages. This resolution was set to establish a balance between over-clustering and subpopulation level classification of abundant lineages such as pyramidal cells and oligodendrocytes (Figure 5A). With this approach, our analysis segregates pyramidal neurons into 6 distinct layers, including newly formed migratory (NFM), newly formed ventral (NFV), CA1-dorsal (CA1-D), CA1-ventral (CA1-V), CA2, and CA3 lineages. Additionally, oligodendrocyte and oligodendrocyte progenitor cells (OPCs) were distinguishable into 2 lineages each. The rest of the lineages resolved here, i.e., DG, intermediate neurons, interneurons, microglia, astrocytes, pericytes, endothelial, ependymal, and CD274^+^ neuroimmune cells, were classified as they exist in the original study (39). Finally, the high fidelity of cell type identification was set by cross-validating the pattern of observed differentially expressed genes (DEGs) with known markers (Figure 5B). Most of these DEGs were concomitant with known bona fide markers that are shown to be vital elements in the classified lineages (details provided in https://github.com/Manu-1512/Zbtb20/). Intriguingly, from the repertoire of DEGs, we find that the upregulation of *Zbtb20* was flagged in the “intermediate cell” cluster. “Intermediate cells” were identified based on the upregulation and coexpression of DG-specific genes (*Prox1* and *Rfx3*) and developing pyramidal neuronal genes (*Nfia* and *Rgs6*). Moreover, *Sema3c*, a gene that assists in developing the physiology of pyramidal neurons (45), was exclusively expressed in “intermediate cells” among all neuronal lineages. Of note, in consistency with genome wide in situ hybridization image data (mouse.brain-map.org), *Zbtb20* expression is perceivable in DG, CA1-D, CA3, astrocytes, and vascular cells (Figure 6, A and B). Taken together, the upregulation of *Zbtb20* marks the intermediate cells that seem to link DG and developing pyramidal neuronal populations, which accords with its postulated function of neuroplasticity in health and pathologies.

Next, we asked whether rhEPO treatment affects the *Zbtb20* expression pattern in any or many of the aforementioned lineages. By comparing the transcriptome of identical cell types between rhEPO- and placebo-treated mice, we identified a substantial dysregulation of *Zbtb20* in multiple cell types. While the dysregulation was nominal in vascular and neuroimmune cells, pyramidal (NFM) neurons exhibited the dramatic effect of rhEPO on *Zbtb20* expression (Figure 6C). Most of pyramidal (NFM) cells expressing *Zbtb20* are from rhEPO samples (Figure 6D), indicating that *Zbtb20* expression is induced upon rhEPO specifically in 1 lineage. Because pyramidal (NFM) cells are highly specialized in combating the disorders of mood, memory, and cognition, and in the potential to intricately integrate in neural circuits and the induction of immediate early genes upon metabolic challenges, our results point toward ZBTB20 as a player for future therapeutic purposes. Considering the transcription factor activities of ZBTB20, overall, the involvement of ZBTB20 in linking neuronal activity to gene expression and plasticity upon rhEPO is proposed.

## Discussion

The objective of the present study was to provide a comprehensive behavioral characterization of ZBTB20-deficient mice, a construct-valid animal model of Primrose syndrome, and to shed light on rhEPO as a potential therapeutic intervention. We report here that *Zbtb20^+/–^* mice exhibit primary ASD-like behavior, comprising impairments in ASD core domains sociability and communication, despite unaffected olfaction, as well as repetitive conduct (1). Second-line symptoms associated with ASD, such as hyposensitivity toward heat-mediated nociception, compromised cognitive flexibility, and hyperactivity, are additionally observed. Notably, hyperactivity is described here in murine ZBTB20 deficiency, while impaired cognition and hyposensitivity for heat-mediated pain have previously been identified in *Zbtb20^+/–^* mice (12, 37). Intriguingly, rhEPO treatment indeed attenuates the *Zbtb20^+/–^* phenotype; namely, it enhances sociability, cognitive flexibility, working memory, as well as the drive and motivation to explore.

The abundance of ZBTB20 in the hippocampus (8) has led this and other studies of ZBTB20-deficient mice (15, 36) to investigate hippocampus-dependent properties that revealed impaired learning and memory performance in various behavioral tests. This was similarly found in human patients with Primrose syndrome. Here, several children and adults were also diagnosed with ASD and/or ADHD in the sense of a double-diagnosis (23, 34). Since prior data indicated an increase in ZBTB20 expression in hippocampal CA1 neurons following rhEPO treatment (40), we hypothesized that rhEPO might attenuate the *Zbtb20^+/–^* phenotype. Indeed, sociability, cognitive flexibility, working memory, and motivation to explore improved in *Zbtb20^+/–^* mice upon rhEPO treatment. Previously, beneficial effects of EPO on social interaction in rats were demonstrated in an ASD model (46) and in models of perinatal brain injury (47, 48). Cognitive flexibility, assessed by reversal tasks in the Morris water maze, was compromised in *Zbtb20^+/–^* males and, in agreement with other studies (48, 49), rhEPO clearly enhanced their respective performance. Analogously, superior cognitive flexibility was observed in mice with a constitutively active EPO receptor (50), and rhEPO markedly increases hippocampus-dependent learning and memory (51–61).

EPO has a promoting impact on working memory in ZBTB20-deficient mice as shown in the Y-maze test. This aligns with a mouse study on rhEPO in traumatic brain injury (62). Furthermore, a synthetic EPO derivative without erythropoietic effect improved working memory in a rat model of multiple sclerosis (63). Patients with treatment-resistant major depression exhibited amended working memory upon rhEPO in a functional MRI task (64). Exploratory behavior, evaluated by the hole board test, delivered a surprise; *Zbtb20^+/–^* males demonstrated a lower number of rigorous revisits, an unexpected phenotype that was assuaged by EPO treatment. This could be in some line with rat studies, reporting mitigation by rhEPO of adverse effects like anxiety upon stress (65) or upon transient middle cerebral artery occlusion (66). A negative correlation between EPO levels and anxiety rating was found in patients with generalized anxiety disorder (67). Attenuating the ASD phenotype in ZBTB20-deficient mice, rhEPO might be a promising treatment approach for Primrose syndrome.

The marble burying test appraises stereotypical and repetitive behaviors (68). Unexpectedly, *Zbtb20^+/–^* mice of both sexes exhibited a markedly decreased number of buried marbles. Therefore, we replicated this test in a second experimental cohort and incorporated additional examinations to investigate potential reasons for this unforeseen phenotype. Once again, *Zbtb20^+/–^* mice demonstrated a decreased number of buried marbles. To rule out anxiety-like behavior toward novel objects, the neophobia test and to exclude anhedonia and depression-like behavior, sucrose preference, tail suspension, and forced swim tests were performed, all revealing normal conduct. Even though just a few cases of individuals with Primrose syndrome, co-diagnosed with anxiety, are known (22, 69, 70), future studies might want to include tests for anxiety-like behavior in mice. This needs careful planning, as these tests require them to be naive to handling (71).

The present study employed MRI volumetric analyses of the brain in an animal model of *Zbtb20* deficiency. It uncovered increased volumes of the hippocampus, cerebellum, brain matter, and whole brain in ZBTB20-deficient males. Weight assessment of *Zbtb20^+/–^* male brains immediately after extraction and following lyophilization showed an increase in both weight and dry weight. In contrast to *Zbtb20^+/–^* mice, MRI scans of human patients diagnosed with Primrose syndrome predominantly exhibited malformation or agenesis of the corpus callosum. There were also sporadic cases of colpocephaly, Chiari malformation, and partial calcification of the basal ganglia (24, 35, 72, 73). While most studies of Primrose syndrome report on macrocephaly (22–29, 31–33, 69, 72), only 2 describe megalencephaly (74), which aligns with the findings of the present study. The observed imbalance between reports of macrocephaly and megalencephaly might be attributable to the different methods applied. While macrocephaly can be determined through a simple measurement of head circumference, diagnosing megalencephaly requires either in vivo imaging or postmortem tissue analyses. Previously, a reduction in wet and dry weight of lungs and hearts of *Zbtb20^–/–^* mice was reported (75) which, however, disappeared when normalized to body weight (BW) or tibia length. While *Zbtb20^+/–^* males exhibited an increase in BW and tibia length, there were no differences in weight of lungs and hearts. Obviously, the genotype is the driving factor, with homozygous ZBTB20 deficiency causing a more severe impact on lungs and hearts.

While *Zbtb20^+/–^* males displayed a pronounced ASD phenotype, females did so just partially. Among the social tests conducted, only nest building revealed abnormalities, showing reduced quality of nests in both placebo- and rhEPO-treated *Zbtb20^+/–^* groups. Similarly to males, females demonstrated repetitive behavior due to excessive climbing in their home cages and hyposensitivity toward heat-mediated nociception, the latter being rescued by rhEPO treatment. While *Zbtb20^+/–^* males exhibited impairments in spatial learning and memory, as well as reversal learning and memory in the Morris water maze, *Zbtb20^+/–^* females showed only difficulties with spatial and reversal memory, in agreement with *Zbtb20^+/–^* females in the Barnes maze (12). A lack of motivation to explore in females was also evident in the tendency toward a reduced number of rigorous revisits and marbles buried, as was the phenotype of hyperactivity. Unlike in males, thermal analysis during the 3-chamber sociability test revealed a decreased centralization index during sociability testing, with no differences observed during habituation. This suggests a reduced stress level in *Zbtb20^+/–^* females when forced into a situation of social interaction. MRI revealed a reduced olfactory bulb volume in both placebo- and rhEPO-treated *Zbtb20^+/–^* females. The volumes of the hippocampus and cerebellum were higher in placebo-treated *Zbtb20^+/–^* females and reduced to control levels under rhEPO conditions. Additionally, ventricle volumes were reduced in rhEPO-treated mice.

In human studies, 50% of individuals diagnosed with Primrose syndrome were reported to be male, 40% female, and 10% without stated sex (21–35, 69, 70, 74, 76, 77), indicating a sex-independent distribution of Primrose syndrome across the population.

### Conclusions

The present study offers an in-depth behavioral characterization of male and female ZBTB20-deficient mice, which exhibit ASD-like behavior with slight sex differences. MRI revealed structural changes in specific brain regions. Together, these results suggest ZBTB20-deficient mice as a valuable experimental model for Primrose syndrome. Importantly, 3 weeks of rhEPO treatment, initiated at postnatal day (PND) 28, ameliorates the phenotype. This puts rhEPO in the spotlight as potential treatment for Primrose syndrome.

## Methods

### Mice

#### Sex as a biological variable.

Both sexes were tested in this study. Unless otherwise specified, mice were separated by sex, genotype, and treatment and housed in standard type II cages (Tecniplast) in groups of 3–5 within ventilated cabinets (Scantainers) in a temperature- and humidity-controlled environment (~22°C, ~50%) with a 12-hour light/dark cycle (lights on at 7 am) and water and food (Sniff Spezialdiäten) ad libitum. Cages were provided with wood-chip bedding and nesting material (Sizzle Nest, Datesand). Group sizes were predicated on previous experience and under consideration of the RRR principle. All experiments were conducted by investigators unaware of group assignment (fully blinded).

The 129/Sv mice, carrying the *Zbtb20* mutant allele, have been previously described (14). In this study, mice were bred by crossing C57BL/6N *Zbtb20^+/–^* males with C57BL/6N WT females. This ensured the birth of only *Zbtb20^+/–^* and *Zbtb20^+/+^* mice as littermates (control), thereby preventing the birth of neonatally lethal *Zbtb20^–/–^* animals. Mutations of the *Zbtb20* allele were confirmed by PCR-based genotyping (8, 11–14). Mice were weaned at PND 21 and separated by sex and genotype to avoid inclusion effects or aggressive behavior against potentially affected animals. Experiments started with the first cohort at an age of 4 weeks (PND 28; males), and with the second cohort at an age of 7 weeks (PND 50; females) and stretched over a period of approximately 5 months each. Later, a (partial) replication and extension cohort was necessary, with females starting on PND 28 and males on PND 36.

### EPO/placebo injections

Animals were categorized into 2 groups based on sex, and further subdivided into 2 subgroups based on genotype. At PND 28, 50% of the mice in each subgroup received a total of 11 intraperitoneal injections of 5000 IU/kg rhEPO (NeoRecormon, Roche) or placebo (solvent solution, 0.01 mL/g BW), every other day over a period of 3 weeks (51).

### Behavioral characterization

Behavioral experiments were performed during the light phase (unless stated otherwise) as reported in detail previously (68, 78, 79) and in the following order:

#### First (male) and second (female) cohort.

Open field, hole board, Y-maze, social interaction in pairs, 3-chamber sociability test, marble burying test, LABORAS, nest building test, vocalization test, prepulse inhibition, Morris water maze, forced swim test, and 4-hour complex wheel running.

#### Replication and extension cohort (males, females).

Neophobia, Rotarod, grip strength, marble burying, sucrose preference test, buried food test, tail suspension test, hot plate test. Individual tests are described in detail in Supplemental Methods.

#### Thermography.

Methodology including data extraction and processing have been described in detail previously (80). Briefly, during Y-maze, social interaction in pairs, and 3-chamber sociability test, an A655sc infrared thermography camera (FLIR Systems) was positioned above the respective arenas to capture images at a resolution of 640 × 480 pixels and frame rates of 5 Hz (for Y-maze and sociability test) or 25 Hz (for the social interaction in pairs), utilizing the ResearchIR software (FLIR Systems). OpenCV 4 in Python 3.6 was employed to extract thermal data. Images were loaded and normalized to values ranging from 0 to 255, with higher values indicative of higher temperatures. For each of the 3 tests, regions of interest (ROIs) were delineated for the extraction of thermal data. A binary mask, encompassing the whole body (including the tail), was generated by applying intensity thresholding and processing steps to mitigate image noise. Consequently, a large cluster of interconnected pixels within these ROIs could be identified, forming the contour of the mouse. Owing to variations in shape and temperature, the whole-body area could subsequently be segmented into a central body region and a distinct tail region, thereby facilitating the extraction of the mean temperatures of both areas. The parameter assessed was the temporal variation in temperature, expressed as centralization index, i.e., ratio of body temperature to tail temperature (80).

#### Perfusion.

Following the last test, i.e., complex wheel running for 4 hours, mice were immediately anesthetized using Avertin (Sigma-Aldrich; 600 mg/kg BW intraperitoneal). Subsequently, they were subjected to transcardial perfusion with Ringer solution for 5 minutes (Ringer Fresenius, Fresenius KABI), followed by 4% formaldehyde/PBS for 10 minutes.

### Multi-animal pose tracking

To analyze mouse behavior in the social interaction in pairs paradigm, we applied SLEAP 1.2.9 (Social LEAP, open source) (81) for pose and position tracking. A 15-node skeleton was used to label 1300 frames across all videos recorded for this experiment evenly divided between male and female mice. This training package was used to train a top-down model with 2 components: a centroid model to locate the mice, and a centered instance model to identify individual nodes within a 384-pixel-wide cropped box. Those models were then used to run inference on all videos, and the output was manually proofread for switches. The resulting proofread inference was exported as h5 files for further analysis.

### MRI

Mice were weighed, anesthetized with ketamine (75 mg/kg BW) and medetomidine (1 mg/kg BW), followed by endotracheal intubation. A specialized respirator, specifically designed for rodents (Animal Respirator Advanced, TSE Systems), was employed for controlled ventilation, supplying room air, enriched with oxygen and mixed with isoflurane (0.5%–2%). A pressure sensor allowed monitoring breathing rate, potential spontaneous breathing, or any movements. Body temperature was controlled using a rectal thermometer and regulated with a warm water circulation system. Mice were positioned in the MRI with heads secured in a tooth and palate holder (82). MRI was performed at a magnetic field strength of 9.4 Tesla using a 4-channel receive-only mouse head coil (Biospec, Bruker BioSpin). For the purpose of volumetric analyses, magnetization transfer–weighted (MT-weighted) images were acquired with a 3D fast low-angle shot (FLASH) sequence (with echo time/repetition time [TE/TR] = 3.4/15.2 ms, flip angle 5°, Gaussian-shaped off-resonance pulse [off-resonance frequency 7.5 ppm, RF power 6 μT], and 100 μm isotropic spatial resolution).

#### MRI data analyses.

For volumetric analyses, MT-weighted images were converted to the NIfTI format, then preprocessed using denoising and bias field correction techniques (83). Preprocessing aimed at generating an unbiased anatomical population template. This was achieved using the optimized ANTs template construction pipeline (GitHub, CoBrALab). To visualize volumetric changes, nonlinear deformation fields of extracted brain images were utilized to create voxel-wise Jacobian determinants. To quantify the volume of specific brain areas, ROIs were identified, including the olfactory bulb, hippocampus, thalamus, corpus callosum, ventricles, cerebellum, and whole brain. These ROIs were determined on the study template through a process of manual segmentation, facilitated by software package AMIRA (Visage Imaging GmbH). Subsequently, these ROIs were retransformed into the subject space. Each of these retransformed ROIs was individually checked and manually corrected if necessary. Finally, volume information was extracted from these corrected ROIs and used for statistical analyses and illustration purposes.

#### Organ collection.

Immediately following MRI, mice were weighed — still under anesthesia — and sacrificed for organ collection, including brain, heart, and lungs. Organs were weighed and stored in 2 mL Eppendorf tubes until subjected to lyophilization, carried out at –56°C for 48 hours under a vacuum pressure of 0.2 mBar, utilizing the Christ LMC-1 BETA 1-16 lyophilizer. In addition, tibiae were taken as secondary reference point (after BW) to create weight- and size-adjusted ratios for the various organs.

### Immunohistochemistry

Mice were deeply anesthetized with Avertin (Sigma-Aldrich; 600 mg/kg BW intraperitoneally) and transcardially perfused via left cardiac ventricle with Ringer’s solution followed by 4% paraformaldehyde (PFA) in PBS (0.1 M, pH 7.4). Dissected brains were postfixed overnight in 4% PFA and frozen at –80°C after cryoprotection with 30% sucrose. Coronal sections of 30 μm thickness were cut with cryostat Leica CM1950 (Leica Microsystems) and stored at –20°C in cryoprotective solution (25% ethylene glycol and 25% glycerol in PBS). For immunohistochemistry, sections were permeabilized in PBS containing 0.3% Triton X-100 (PBST; Sigma-Aldrich) and blocked with 5% normal horse serum (NHS; Jackson ImmunoResearch Laboratories) for 1 hour at room temperature. Subsequently, slices were incubated with main primary antibody anti-ZBTB20 (rabbit, 1:500; Synaptic Systems, 362003) diluted in 5% NHS with 0.3% PBST over 3 nights at 4°C. After washing, secondary antibody goat anti-rabbit Alexa Fluor 555 (1:500; Invitrogen, A-21428) was incubated in 3% NHS/0.3% PBST for 2 hours at room temperature. Sections were finally counterstained with 4′,6-diamidino-2-phenylindole (DAPI; 1:5000; MilliporeSigma) and mounted on SuperFrostPlus Slides (Epredia) with Aqua-Poly/mount (Polysciences, Inc). A total of 12 mice (6 WT and 6 *Zbtb20^+/–^*) were used to assess the hippocampus. Images were obtained with a TCS-SP5 inverted system (Leica) equipped with a 20× objective (NA = 0.70). Representative images were taken at Bregma –1.58 mm.

### Quantitative RT-PCR

Hippocampal RNA was extracted from either cryosections (4 sections per mouse) or from whole hippocampus. The extraction was performed using the NucleoSpin totalRNA FFPE Kit (Macherey-Nagel, 740982) or the miRNeasy Mini Kit (QIAGEN), respectively. The cDNA was then synthesized using the SuperScript III Reverse Transcriptase (Thermo Fisher Scientific/Life Technologies GmbH). This process involved the use of 1 μg of RNA, along with oligo (dT) and random hexamer primer, in a total reaction volume of 20 μL. Subsequently, reverse transcriptase qPCR (RT-qPCR) was performed using 5 μL of Power SYBR Green PCR Master Mix (Thermo Fisher Scientific, 4367660). This procedure utilized 4 μL of 1:10 diluted cDNA as template and 1 pmol of primers (dissolved in 1 μL of H_2_O). Fold changes in gene expression were calculated with the ΔΔCt method, using β-actin and *Hprt1* as reference genes. The qPCR reactions (3 technical replicates) were run on a LightCycler 480 System (Roche). The primers used were *Zbtb20* forward: 5′-CAGCCCTCATCCACTCGAC-3′ and *Zbtb20* reverse: 5′-TCCCCTTGCAACTGATGTCAC-3′; β-actin forward: 5′-CTTCCTCCCTGGAGAAGAGC-3′ and β-actin reverse: 5′-ATGCCACAGGATTCCATACC-3′; *Hprt1* forward: 5′-GCTTGCTGGTGAAAAGGACCTCTCGAAG-3′ and *Hprt1* reverse: 5′-CCCTGAAGTACTCATTATAGTCAAGGGCAT-3′.

### High-parameter flow cytometry

The frequencies of 6 major and 19 minor immune cell subsets were quantified in blood, bone marrow, lymph node, and spleen samples of WT versus *Zbtb20^+/–^* mice as described in the Supplemental Methods. Pairwise comparisons between *Zbtb20^+/–^* and WT mice were performed using either the Wilcoxon rank-sum test with continuity correction or unpaired 2-sided *t* tests with or without Welch’s correction depending on data normality and variance. To account for multiple testing, *P* values were adjusted within each organ for all cell populations using the Benjamini-Hochberg false discovery rate (FDR) procedure. Both raw *P* values and FDR-adjusted *P* values are reported. Multivariate analysis of the global immune cell composition was performed using principal component analysis (PCA) on *z*-scored features, with 95% confidence ellipses plotted per genotype and organ. Group differences in overall immune profiles were assessed separately within each organ by PERMANOVA based on Euclidean distances of the scaled data.

### snRNA-seq

This study was commenced by fetching the normalized data available from our previous study (39). We reanalyzed the observed lineages to provide a holistic catalog at finer resolution. After the batch correction, we performed unsupervised clustering of the single-cell gene expression profiles of all hippocampal samples, which identified 19 clusters at a resolution of 0.4 (preset by Seurat tool). We then examined the top DEGs for each cluster relative to all other clusters which enabled us to calculate the expression and differential expression of *Zbtb20*. UMAPs, feature plots, violin plots, and heatmaps were constructed using default functions of tools we implemented, except that we set the color scale manually. The detailed code to regenerate all the figures and analysis corresponding to this snRNA-seq dataset is available at https://github.com/Manu-1512/Zbtb20/ Raw and processed snRNA-seq data are publicly accessible in the NCBI GEO via accession code GSE220522.

### Statistics

Unless specified otherwise, statistical analyses were conducted utilizing Prism9 software (GraphPad Software) and results are presented as mean values ± SD. The normality of data distribution was assessed using the Shapiro-Wilk test, with an α error threshold set at 0.05. Depending on the distribution of the data, either 2-tailed unpaired Welch’s corrected *t* tests or Mann-Whitney *U* tests were employed to facilitate comparisons between groups. In the case of repeated measure data, a mixed-model analysis of variance (ANOVA) was applied. Statistical significance was determined based on *P* values less than 0.05.

### Study approval

All animal experiments were carried out with the approval of the local Animal Care and Use Committee (Niedersächsisches Landesamt für Verbraucherschutz und Lebensmittelsicherheit, LAVES, Oldenburg, Germany; license 42502-04-18/2803).

### Data availability

All reported data are obtainable. The detailed code to regenerate all the figures and analysis corresponding to the snRNA-seq dataset is available at https://github.com/Manu-1512/Zbtb20/ Raw and processed snRNA-seq data are publicly accessible in the NCBI GEO via accession code GSE220522. For extensive data accessibility, consult Supporting Data Values, Supplemental Figures 1–3, Supplemental Tables 1–4, and Supplemental Methods.

## Author contributions

HE supervised the study. MH and HE conceptualized and designed the study. HE and KAN acquired funding. MH, AR, VDG, JBHW, YC, UJB, SNO, KH, MS, RD, UÇ, SB, AS, ABT, KAN, and HE acquired, analyzed, and interpreted data. MH and HE drafted the manuscript. MH, JBHW, MS, and HE generated figures. All authors read and approved the final version of the manuscript.

## Funding support

- European Research Council (ERC) Advanced Grant under the European Union’s Horizon Europe research and innovation programme (acronym BREPOCI; grant agreement no. 101054369 to HE).
- Max Planck Society.
- Max Planck Förderstiftung.
- Sonderforschungsbereich/Collaborative Research Centers TRR 274/2 – 408885537 project C01 (to HE and KAN).
- IMPRS-Genome Science PhD program (to VDG and UÇ).
- NextGenerationEU via Bulgarian National Recovery and Resilience Plan, project no. BG-RRP-2.004-0009-C03 (MUVE-TEAM) (to ABT).

## Supplementary Material

Supplemental data

Supplemental tables 1-4

Supporting data values

## Version 1 02/23/2026

Electronic publication
