# Supplementary material for: Erythropoietin alleviates syndrome-associated intellectual disability and autism-like behavior in Zbtb20-haploinsufficient Primrose syndrome mouse model
Source: JCI Insight. 2026 Feb 23;11(4):e200021. doi: 10.1172/jci.insight.200021 (PMC12956014; doi:10.1172/jci.insight.200021)
Supplement: Supplemental data [file jciinsight-11-200021-s091.pdf]

Erythropoietin alleviates intellectual disability and autism-like behavior of mice caused by Zbtb20 haploinsufficiency, a construct-valid model of Primrose syndrome

- *Supplementary Methods* -

Behavioral characterization of the original *Zbtb20* cohort

Open Field: To assess exploratory behavior in a novel environment, mice were positioned in the center of a well-lit, gray, circular Perspex area (120 cm diameter, 25 cm height of outer wall). Initially, the animals latency to reach the outer wall was measured. Automated tracking (Viewer3, Biobserve, Bonn, Germany), commenced for a duration of 7min once the animals reached the outer wall independently, or were positioned there following a cut-off time of 180s. Mouse movement was tracked throughout the digitally defined zones of the outer periphery, center and the intermediate zone in between. Analyzed readouts were escape latency to the periphery, time spent, distance covered and average velocity within the respective zones and zone crossings. Animals were tested at the age of 7 (males) and 10 (females) weeks, under a light intensity of 125 lx in the center.

Hole Board: In order to assess exploratory behavior and impulsivity, mice underwent testing in a Hole Board setup. On a 50 x 50 cm plate, encircled by nontransparent outer walls (35 cm high) and 2 cm above the ground of the box, were 16 holes (2 cm diameter) arranged in a 4x4 grid. Each mouse was individually placed into the setup for a duration of 5 minutes. The occurrence of head dips was monitored by light beam sensors positioned beneath the holes. The primary metric analyzed were the total number of visits to the holes and rigorous re-visits, defined as consecutive visits to the same hole within a 3 second interval. Animals were tested at the age of 8 (males) and 11 (females) weeks, under a light intensity of 15 lx.

Y-Maze: To assess working memory, mice were positioned at the end of one randomly selected arm within a Y-shaped setup (each arm 43 cm long, 7.5 cm wide, 13 cm high). Over a period of 5 minutes, the mice were recorded (Viewer3, Biobserve, Bonn, Germany) as they were permitted to explore the setup freely. Upon entering a new arm and subsequently exiting it, it was noted whether the mice returned to the most recent arm they had visited (non-alternating entry) or ventured into the other arm (alternating

entry). Analyzed readouts were the amount of alternating, non-alternating and total entries and the delta between alternating and non-alternating entries. Animals were tested at the age of 8 (males) and 11 (females) weeks, under a light intensity of 15 lx.

Social Interaction in Pairs: Within a neutral enclosure (40x40x40 cm), supplemented with bedding, test mice were individually habituated over a period of two consecutive days (10 min/day). On the third day, a social interaction test was conducted by pairing two unfamiliar mice of the same genotype and sex within the neutral enclosure for a duration of 10 minutes. The movements and behaviors of the mice were captured by an A655sc infrared thermography camera (FLIR Systems, Oregon, USA), recording images at 640x480 pixels and a framerate of 25Hz. The camera was linked to a computer located in a separate room, where the ResearchIR software (FLIR Systems, Oregon, USA) facilitated the control and adjustment of the recording settings. The thermography camera was employed to enhance the contrast between the mice and the background (bedding), thereby improving the automated tracking and analysis of the interactions between the animals, a process overseen by Princeton University, USA. Animals were tested at the age of 9 (males) and 12 (females) weeks, under a light intensity of ~100 lx.

Three Chamber Sociability Test: The test for sociability and social memory was conducted as delineated in previous literature (1). The testing environment consisted of a rectangular box (61x40x22 cm), which was partitioned into three equal chambers (20x40x22 cm each) by Plexiglas walls. Each wall featured an opening 5 cm wide to facilitate access to all chambers. Prior to each trial, the entire chamber was cleaned and replenished with fresh bedding. During the initial habituation phase (1st trial), the test mouse was situated in the central chamber and granted a 5- minute exploration period. During this phase, access to the outer chambers was obstructed by plastic boxes. For the subsequent sociability test (2nd trial), an unfamiliar stimulus mouse of the same age and sex (referred to as stimulus mouse, C3H) was placed within a small metal cage (10 cm x 6 cm x 6 cm) located in one of the outer chambers. This arrangement permitted nose contact through the bars of the cage while preventing aggressive encounters. A similar cage was positioned in the other outer chamber but left vacant. Both cages were secured in place with a filled water bottle. All stimulus mice had been previously acclimated to the small cages and the novel environment. The test mouse was then reintroduced to the central chamber and allowed to freely explore the entire setup for a duration of 10 minutes. The mouse's

movements were automatically tracked and recorded using Viewer3 software (Biobserve, Bonn, Germany). The parameters assessed included the time spent within the respective chambers and in close proximity to the small metal cages (insets) housing the stimulus mice, the latency to the first approach, the number of zone crossings, and the total distance travelled. Animals were tested at the age of 10 (males) and 13 (females) weeks, under a light intensity of ~130 lx.

Marble Burying: This experimental design was employed to evaluate stereotypical and obsessive-compulsive behavior. The subject mouse was situated in a type III cage (Techniplast), which was filled with bedding to a depth of 5 cm. This cage was encircled by an insert (53x31x29 cm). Within, 24 glass marbles (1.0-1.5 cm diameter) were arranged in a 4x6 grid, with an approximate distance of 4 cm separating each marble. The test subjects were permitted to freely explore the area and interact with the marbles over a period of 30 minutes. The parameter analyzed was the number of marbles that were buried (at least to 2/3 their depth). Animals were tested at the age of 10 (males) and 13 (females) weeks, under a light intensity of ~7 lx.

LABORAS: The Laboratory Animal Behavior Observation Registration and Analysis System (LABORAS, Metris, Hoofddorp, Netherlands) has been comprehensively detailed in previous literature (2). To summarize, mice were individually housed in standard type 1 cages (Tecniplast) with wood-chip bedding, and provided with food and water ad libitum. This arrangement was maintained for two nights as the mice acclimated to the experimental room. On the third night, the individually housed mice, still within their respective cages, were positioned on a sensor platform that was interfaced with a computer located in a separate room. This computer was equipped with the LABORAS software (Metris, Hoofddorp, Netherlands). The movements of the animals were converted into electrical signals and subsequently recorded. The software was capable of distinguishing between various behavioral categories, such as scratching, climbing, circling, grooming, immobility, locomotion, eating, and drinking. It also quantified the duration (in seconds) of each event and their frequency of occurrence. The recording of behavior commenced two hours prior to the dark phase and continued for a duration of 15 hours, facilitating subsequent analysis of the behavior exhibited during the dark phase. Animals were tested at the age of 11-12 (males) and 14-15 (females) weeks, under a light intensity of ~0 lx.

Nestbuilding Test: During the second night of the habituation phase of the LABORAS test, the experimenters removed the existing nesting material from the enclosures of the individually housed mice. This occurred two hours prior to the onset of the dark phase, at which point it was replaced with a nestlet composed of compressed cotton, weighing approximately 3 grams. The following morning, the quality of the nest building was evaluated by rating the nests on a scale from 1 to 5, as per the methodology outlined by Deacon in 2006 (3). Subsequent to this assessment, the cotton nestlet was removed and the mice were once again provided with their regular nesting material. Animals were tested at the age of 11-12 (males) and 14-15 (females) weeks, under a light intensity of ~0 lx.

Ultrasound Vocalization: Mice that were individually housed were subjected to testing for ultrasonic vocalizations (USVs) upon the introduction of an unfamiliar, age-matched female mouse (C57BL/6) into their respective home cages, as detailed in previous studies (4). Following a 2-minute habituation period, an anesthetized mouse (administered Avertin at a dosage of 300mg/kg body weight) was introduced into the cage with the test mouse, which could be either male or female. The USVs were recorded for a duration of 3 minutes using a microphone connected to a preamplifier. The recordings were analyzed at a sampling frequency of 300 kHz utilizing the Avisoft Recorder 4.2 software (Avisoft Bioacoustics, Berlin, Germany). The parameters analyzed included the number and duration of USVs, as well as the latency to the first call. The USVs were distinguished from other sounds using the whistles detection algorithm of the Avisoft-SASLab5.2 software (Avisoft Bioacoustics, Berlin, Germany). The selection criteria for this process were as follows: possible changes per step equaled 4 (4687 Hz), minimal continuity was set at 8 ms, and the possible frequency range was between 35 and 150 kHz. These criteria have been tested and successfully implemented in previous mouse studies (2, 4). Animals were tested at the age of 13 (males) and 16 (females) weeks, under a light intensity of ~55 lx.

Prepulse Inhibition (PPI): The procedure for this test has been comprehensively outlined in earlier studies (5). In brief, to evaluate sensorimotor gating, mice were situated within compact metal cages measuring 9 cm x 4 cm x 4 cm. This arrangement was designed to limit significant movement. These cages were subsequently positioned within sound-attenuating cabinets (TSE Systems, Bad Homburg, Germany) that were mounted on platforms equipped with sensors to monitor

the animals' movements. Following a period of habituation to a background noise level of 65 dB, acoustic stimuli were delivered via loudspeakers to induce startle reflexes. These stimuli varied in intensity (70, 75, 80, 120 dB) and were administered in a pseudo-random sequence. The amplitudes of the startle responses were averaged for each mouse across the various intensities. The Pre-Pulse Inhibition (PPI) was computed as a percentage of the startle response using the formula: %PPI = 100 - [(startle response following the pre-pulse)/(startle amplitude following the pulse only) x 100]. For the purposes of analysis, data from non-performing mice (those exhibiting negative PPI) were excluded from consideration. Animals were tested at the age of 13 (males) and 16 (females) weeks, under a light intensity of 0 lx.

Morris Water Maze: The methodology for this paradigm has been extensively detailed in earlier studies (5, 6). In summary, to evaluate spatial learning and memory, mice were introduced into a circular tank with a diameter of 120 cm and a height of 60 cm. The tank was filled with opaque water maintained at room temperature (~22°C), and featured a slightly submerged escape platform with a diameter of 10 cm. The movements of the mice were automatically tracked and recorded using Viewer3 software (Biobserve, Bonn, Germany). During the initial two days of the visual platform task, the extra maze cues were obscured by the tank walls due to the low water level. The escape platform was visually indicated by a flag. On the second day of the visual platform task, the location of the platform was altered. For the subsequent eight days of the hidden platform task, the platform was relocated once more and the flag was removed. The water level was raised to make the extra maze cues visible. Following this, the platform was removed and the mice were observed during a 'hidden probe trial'. The probe trial was followed by a four-day reversal hidden platform task. In this phase, the platform was relocated yet again. The reversal task was succeeded by another probe trial. During the visual, hidden, and reversal tasks, each day comprised four trials per mouse, each with a cut-off time of 90 seconds and an intertrial interval of five minutes to prevent fatigue. Between trials, the mice were individually housed in cages lined with paper towels and positioned on a heating pad to prevent hypothermia. The primary parameter analyzed was the escape latency. In the absence of a platform to reach, both probe trials consisted of a single 90-second trial per mouse. The parameters analyzed included the time spent in and the number of visits to the target quadrant (TQ), which previously contained the escape platform.

Animals were tested at the age of 14-16 (males) and 17-19 (females) weeks, under a light intensity of ~130 lx.

Forced Swim Test: The assessment of depression-like behavior was conducted in accordance with the methodology previously delineated (7). The subject mouse was introduced into a glass cylinder filled with water at approximately 24°C. The cylinder dimensions were 27 cm in height and 17 cm in diameter, with the water level maintained at around 15 cm. The primary parameter under analysis was the duration of immobility, measured in seconds. Immobility was characterized as periods during which the mouse, while maintaining an upright position, executed only minimal movements necessary to keep its head above the water surface. Animals were tested at the age of 17 (males) and 20 (females) weeks, under a light intensity of ~125 lx.

Complex Wheel Running: To stimulate the expression of cFos, a marker of neuronal activity, within hippocampal neurons, mice were exposed to a complex running wheel (CRW) apparatus for a duration of 4 hours (8). The mice were single housed in type III cages (42x26x18 cm, Tecniplast), each equipped with a CRW. This CRW is distinguished by its standardized configuration of randomly omitted bars (9, 10). Prior to the onset of the dark phase, the mice were allowed a 2-hour habituation period to acclimate to their new environment and the CRW. With the commencement of the dark phase, the voluntary running activity of the mice was automatically monitored for a 4-hour span using the Phenomaster software (TSE Systems). Animals were tested at the age of 18 (males) and 21 (females) weeks, under a light intensity of ~0 lx.

Behavioral characterization of additional second cohort

Neophobia: In order to evaluate anxiety-like behavior in response to unfamiliar stimuli, mice were acclimatized to a neutral enclosure (dimensions: 40x40x40 cm) supplemented with bedding material, for a duration of 2 days at 10 minutes per day. On the subsequent third day, two identical objects (transparent glass bottles featuring an uneven surface, filled with violet-colored sand, with a height of 16.5 cm and a base diameter of 5.5 cm) were positioned at diametrically opposite locations within the enclosure. The subjects were then introduced into the enclosure and allowed a free exploration period of 5 minutes. The movements of the subjects were automatically monitored and documented using the Viewer3 software (developed by Biobserve,

Bonn, Germany). In addition to the entire enclosure area, circular regions of interest (ROIs) with a radius of 3.5 cm were digitally established around both objects to quantify the duration of interaction. Additional metrics included the distance traversed, the number of crossings between zones, and the frequency of visits to the ROIs. Animals were tested at the age of 7 (males) and 8 (females) weeks, under a light intensity of ~25 lx.

Rotarod: The rotarod system (Ugo Basile Srl, Comerio, Italy) features of a horizontal cylinder with a diameter of 3 cm. This cylinder, divided into 5 sections by 6 circular barriers, each with a width 6 cm, can accommodate up to five mice simultaneously for testing and can be set into rotation. Starting with 4 rpm, the speed increases automatically up to 40 rpm within 5 min. To acclimate the animals to the new setup, the Rotarod was activated, but the cylinder was kept stationary. Subsequently, up to 5 mice were positioned on the cylinder for a duration of 5min. To assess motor performance and learning, on the next two days up to 5 mice were placed onto the cylinder once per day. Once each mouse was safely positioned on top of the cylinder and facing the same direction, the cylinder was set into rotation, with the speed gradually increasing over a period of 5min. Analyzed readout is the latency to fall, measured by a light beam sensor below each of the 5 sections. Animals were tested at the age of 8 (males) and 9 (females) weeks with light intensity of ~300 lx.

Grip Strength: In order to evaluate muscular strength, mice were suspended by the tail and directed towards a horizontal metallic bar. Upon securing the bar with their forepaws, the mice were gently retracted from the setup, during which the maximum force exerted to maintain their grip on the bar was measured. This procedure was repeated for a total of three trials per mouse, and the average value was subsequently computed. Animals were tested at the age of 9 (males) and 10 (females) weeks, under a light intensity of ~350 lx.

Marble Burying: This test was performed as described before. Animals were tested at the age of 9 (males) and 10 (female) weeks, under a light intensity of ~7 lx.

Sucrose Preference Test: In order to evaluate anhedonia in mice, a two-bottle choice paradigm was employed, wherein the animals were granted unrestricted access to both tap water and a 2% sucrose solution (Merck, Darmstadt, Germany). Over a span of 48 hours, the mice were conditioned to consume water from two diminutive bottles, following which they were subjected to water deprivation, thereby initiating the sucrose preference test for the subsequent three days. The initial two days served as an

acclimatization period to the sucrose solution, while the data obtained on the third day was utilized to ascertain the actual sucrose preference. For the purpose of this test, mice that were previously housed in groups needed to be isolated. Consequently, they were individually accommodated in standard Type II cages (Tecniplast, Hohenpleißberg, Germany), each furnished with the aforementioned two bottles for a duration of 60 minutes. The volume of liquid consumed was quantified by weighing both bottles before and after each session. To account for potential side preference, the position of the bottle containing the sucrose solution was alternated. The calculation of sucrose preference was performed as follows: preference [%] = (sucrose solution intake [g] / total fluid intake [g]) × 100 Animals were tested at the age of 10 (males) and 11 (females) weeks, under a light intensity of ~40 lx.

Buried Food Test: In order to evaluate olfactory function, mice underwent a 5-day training regimen aimed at locating a piece of chocolate cereal concealed beneath wood-chip bedding. On the first day, the animals were acclimated to the testing cage (dimensions: 29.5×18.5×13 cm) through two separate 15-minute sessions. On the second day, the mice were once again acclimated to the testing cage for two 15-minute sessions, this time with a piece of chocolate cereal placed atop the bedding. Following this second acclimation session and until the conclusion of the buried food test, the mice were provided access to a standard diet for 1 hour post-acclimation, after which they were subjected to food deprivation until the next training session. The third and fourth days consisted of repetitions of the second day's training regimen. On the fifth day, a piece of chocolate cereal was buried approximately 1.5 cm beneath the bedding, and the mouse was introduced into the cage. The parameters assessed included the latency to the first digging attempts at the correct location and the latency to the first gnawing at the piece of cereal, with a cut-off time set at 300 seconds. To account for intrinsic motivation on that day, a final trial was conducted with the piece of cereal plainly visible atop the bedding. The parameter assessed during this trial was the latency until each mouse either grabbed the cereal or gnawed at it for the first time. Animals were tested at the age of ~11 (males) and ~12 (females) weeks, under a light intensity of ~60 lx.

Tail Suspension Test: In order to evaluate depression-like behavior, mice were subjected to the tail suspension test (11). Briefly, the subjects were affixed to the edge of a table by their tails, suspended at a height of 72 cm above the

ground, and observed for a duration of 6 minutes. Subsequent to this, the recorded videos were analyzed with the objective of determining periods of immobility, measured in seconds. These periods were assessed in 2-minute blocks within the total experimental timeframe of 6 minutes. Animals were tested at the age of ~12 (males) and ~13 (females) weeks, under a light intensity of ~130 lx.

Hot Plate Test: This paradigm was employed to evaluate pain perception. Subjects were positioned on a preheated metal plate (approximately 55°C, 24 cm in diameter, Ugo Basile Srl, Comerio, Italy) encircled by a transparent Plexiglas cylinder (17 cm in diameter, 30 cm in height) to deter the mice from vacating the plate. The parameter analyzed was the latency to exhibit responses to thermal sensation, measured in seconds, which were characterized as paw licking, lifting, or jumping. Upon the exhibition of the initial response, the mice were promptly removed from the plate and returned to their home cage. A cut-off time of 40 seconds was enforced to prevent potential tissue damage. Animals were tested at the age of 12 (males) and 13 (females) weeks, under a light intensity of ~125 lx.

Cell isolation from lymphoid organs

Mice were sacrificed by CO_2 and immediately processed for organ collection. Whole blood (250µL) was collected via cardiac puncture and transferred into EDTA coated tubes (REF 20.1341, Sarstedt). Red blood cells were lysed by incubating blood samples with 2mL ACK buffer (0.15M NH_4Cl, 10mM $KHCO_3$, 0.1mM EDTA, pH 7.2-7.4) for 15min at room temperature. White blood cells were harvested by centrifugation at 350g, washed with PBS, and stored on ice. Meanwhile, femur bones, spleen, and mesenteric lymph nodes were isolated and processed on ice. Bone marrow cells were harvested by flushing one femur bone per mouse with 10mL cold PBS using a 20G needle and filtering through 70µm nylon cell strainers (REF 431751, Corning). Lymph nodes and spleens were mechanically dissociated in 5mL PBS each, by gently grinding the organs through 70µm nylon cell strainers with syringe rubber plungers. Bone marrow cells and splenocytes were centrifuged and cell pellets were resuspended in 5mL ACK buffer and incubated 5min at room temperature for red blood cell lysis. To stop the lysis, 5mL PBS was added and samples were centrifuged. Cell pellets were resuspended in 10mL PBS and filtered through 70µm nylon cell strainers. For buffer exchange, the samples were centrifuged and resuspended in FACS buffer (2% BSA

in PBS). For staining, samples were transferred into V-bottom 96-well plates using the following volumes: blood = total sample; mesenteric lymph nodes = 200µL of 1mL total sample; bone marrow = 200µL of 2mL total sample; spleen = 200µL of 10mL total sample. After transfer, cells were centrifuged, resuspended with 100µL FACS buffer and 22µL staining master mix (composition see table below) and incubated 30min on ice. Stained cells were washed 2 times, resuspended in FACS buffer, and filtered through 35µm cell strainers into FACS tubes (REF 55.1579, Sarstedt). Data was acquired on a FACSymphony S6 (BD) and stopped after 5000 quantification beads were recorded per sample. A total of 120 samples (n=3-4 lymphoid tissues per mouse, n=6-8 mice per sex and genotype) were analyzed. One female wildtype mouse was excluded due to a hypertrophic spleen.

Staining master mix. Volumes per sample.

RRID	Marker	Fluorochrome	Clone	Cat#	Company	Vol (µL)
AB_2651134	CD45	BUV395	30-F11	565967	BD	0.25
AB_2874241	CD8a	BUV661	53-6.7	750023	BD	0.25
AB_2871390	CD138	BB700	281-2	742124	BD	0.5
-	CD44	BUV737	IM7	612799	BD	0.5
-	CD4	BUV563	RM4-5	569182	BD	0.25
AB_2743990	CD62L	BV480	MEL-14	746726	BD	0.25
AB_2740505	CD16/32	BV786	2.4G2	740851	BD	0.5
AB_396675	CD69	FITC	H1.2F3	553236	BD	1
AB_396958	CD19	AF700	1D3	557958	BD	0.25
AB_2565547	CD27	BV421	LG.3A10	124223	Biolegend	0.5
AB_2562679	CD115	BV711	AFS98	135515	Biolegend	1
AB_312793	CD11b	PE-Cy5	M1/70	101210	Biolegend	0.5
AB_389364	NK1.1	PE-Cy7	PK136	108714	Biolegend	0.5
-	Ly6G	AF750	1A8	FAB10371S-100UG	RND-Systems	0.5
-	Brilliant stain buffer	-	-	563794	BD	10
-	CaliBRITE beads	APC	LOT 20131	340487	BD	5
-	Dead cells	DAPI	-	D9542	Sigma	0.2ng

High parameter flow cytometry analysis:

FCS files were imported to FlowJo v.10.9.0, and compensated using single stained controls. An empty 540LP/35BP channel in the 445nm Laser line was used to record autofluorescence. The data was automatically cleaned using the flowAI (12) plugin with default parameters, followed by manual clean-up gating on FSC-A vs. SSC-A to discriminate cells from beads, FSC-A vs. FSC-H for crude removal of doublets, and CD45-BUV395 vs. DAPI for identification of live hematopoietic (DAPI-, CD45+) cells. Live hematopoietic cells were downsampled to 10000 cells per sample using the DownSample V3 plugin in FlowJo. Downsampled live hematopoietic cell populations of all 120 samples were concatenated. Uniform gating was performed on

the concatenated sample as described in Supplementary Figure 3A. First, granulocytes were identified based on high SSC-A and Ly6G-AF750. The remaining cells were gated for CD115 and NK1.1 to identify NK cells (NK1.1+) and monocytes (CD115+). CD115 and NK1.1 negative cells were gated for CD11b for residual myeloid cells (CD11b+, CD16/32+, SSC-A low, CD115-). CD115, CD11b, and NK1.1 triple negative cells were gated for CD16/32 (FcR, Fc receptor) and CD19 to identify B cells (CD19+, FcR+). Negative cells were gated for CD8 and CD4 for CD8+ and CD4+ T-cells, respectively. These major immune cell subsets were further subdivided based on their expression of CD27, CD138, CD44, and CD62L into the following distinct minor immune cell subsets: CD27-/CD138- B cells; CD27+/CD138- B cells; CD138+ antibody secreting cells (13); CD62L+/CD44- naive T-cells, CD62L-/CD44+ effector/ effector memory T-cells, CD62L+/CD44- central memory T- cells, and CD62L-/CD44- T-cells (14–16); CD11b-/CD62L+ monocytes (mostly in bone marrow), CD62L+/CD11b+ monocytes, and CD62L-/CD11b+ monocytes (17); CD62L+/CD27+ NK cells; CD62L+/CD27- NK cells, CD62L-/CD27+ NK cells, and CD62L-/CD27- NK cells (18). For dimensionality reduction, the UMAP-R plugin (19) was run using compensated parameters, uncompensated CD69-FITC, FSC-A, SSC-A, the Euclidian distance metric, 60 nearest neighbors, a minimum distance of 0.5, and 2 components. To quantify the major and minor immune cell subsets within individual samples, the concatenated file was split based on keywords specifying the animal identifier, sex, and organ. Uniform gates were applied on individual samples and cell counts of all immune cell subsets were exported. The major immune cell subsets were normalized to the total number of quantified live hematopoietic cells (10000 per sample). The minor immune cell subsets were normalized to the respective major immune cell subset (parent population).

References

1. Moy SS, et al. Sociability and preference for social novelty in five inbred strains: an approach to assess autistic-like behavior in mice. *Genes Brain Behav*. 2004;3(5):287–302.

2. El-Kordi A, et al. Development of an autism severity score for mice using Nlgn4 null mutants as a construct-valid model of heritable monogenic autism. *Behav Brain Res*. 2013;251:41–49.

3. Deacon RMJ. Assessing nest building in mice. *Nat Protoc*. 2006;1(3):1117–1119.

4. Hammerschmidt K, et al. The structure and usage of female and male mouse ultrasonic vocalizations reveal only minor differences. *PLoS One*. 2012;7(7):e41133.

5. Dere E, et al. Heterozygous ambra1 deficiency in mice: a genetic trait with autism-like behavior restricted to the female gender. *Front Behav Neurosci*. 2014;8:181.

6. Morris RGM. Spatial localization does not require the presence of local cues. *Learn Motiv*. 1981;12(2):239–260.

7. Porsolt RD, et al. Depression: a new animal model sensitive to antidepressant treatments. *Nature*. 1977;266(5604):730–732.

8. Wakhloo D, et al. Functional hypoxia drives neuroplasticity and neurogenesis via brain erythropoietin. *Nat Commun*. 2020;11(1):1313.

9. Liebetanz D, et al. A highly sensitive automated complex running wheel test to detect latent motor deficits in the mouse MPTP model of Parkinson's disease. *Exp Neurol*. 2007;205(1):207–213.

10. McKenzie IA, et al. Motor skill learning requires active central myelination. *Science*. 2014;346(6207):318–322.

11. Steru L, et al. The tail suspension test: a new method for screening antidepressants in mice. *Psychopharmacology (Berl)*. 1985;85(3):367–370.

12. Monaco G, et al. flowAI: automatic and interactive anomaly discerning tools for flow cytometry data. *Bioinformatics*. 2016;32(16):2473–2480.

13. Brynjolfsson SF, et al. Long-lived plasma cells in mice and men. *Front Immunol*. 2018;9:2673.

14. Gerberick GF, et al. Selective modulation of T cell memory markers CD62L and CD44 on murine draining lymph node cells following allergen and irritant treatment. *Toxicol Appl Pharmacol*. 1997;146(1):1–10.

15. Nakajima Y, et al. Critical role of the CD44lowCD62Llow CD8+ T cell subset in restoring antitumor immunity in aged mice. *Proc Natl Acad Sci U S A*. 2021;118(23):e2103730118.

16. Sckisel GD, et al. Differential phenotypes of memory CD4 and CD8 T cells in the spleen and peripheral tissues following immunostimulatory therapy. *J Immunother Cancer*. 2017;5:33.

17. Geissmann F, et al. Blood monocytes consist of two principal subsets with distinct migratory properties. *Immunity*. 2003;19(1):71–82.

18. Goh W, Huntington ND. Regulation of murine natural killer cell development. *Front Immunol*. 2017;8:130.

19. McInnes L, Healy J, and Melville J. UMAP: uniform manifold approximation and projection for dimension reduction. *arXiv preprint arXiv:180203426*. 2018.

Supplementary Figure 1 – Behavior Females

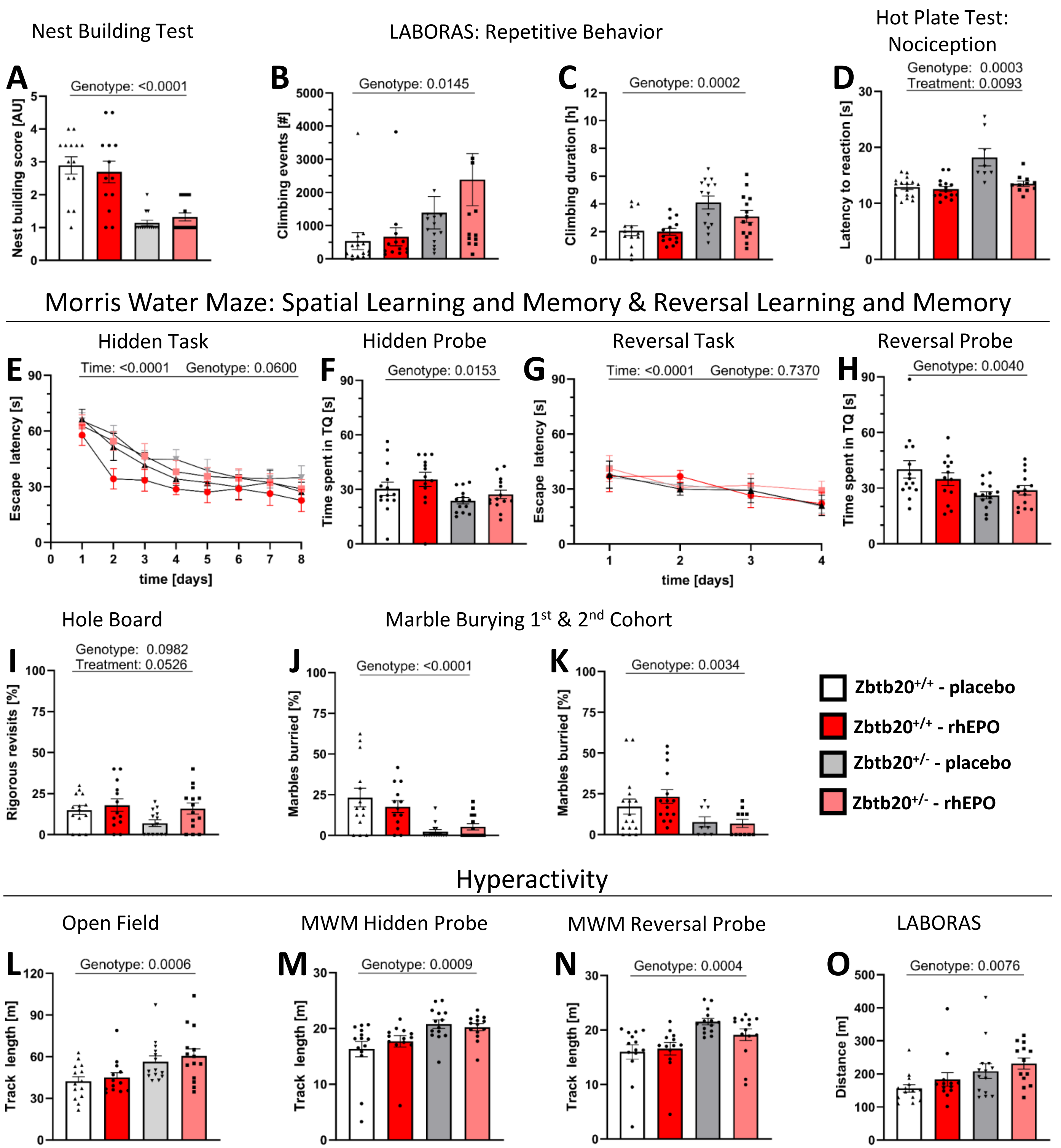

Supplementary Figure 2 – Behavior & MRI Females

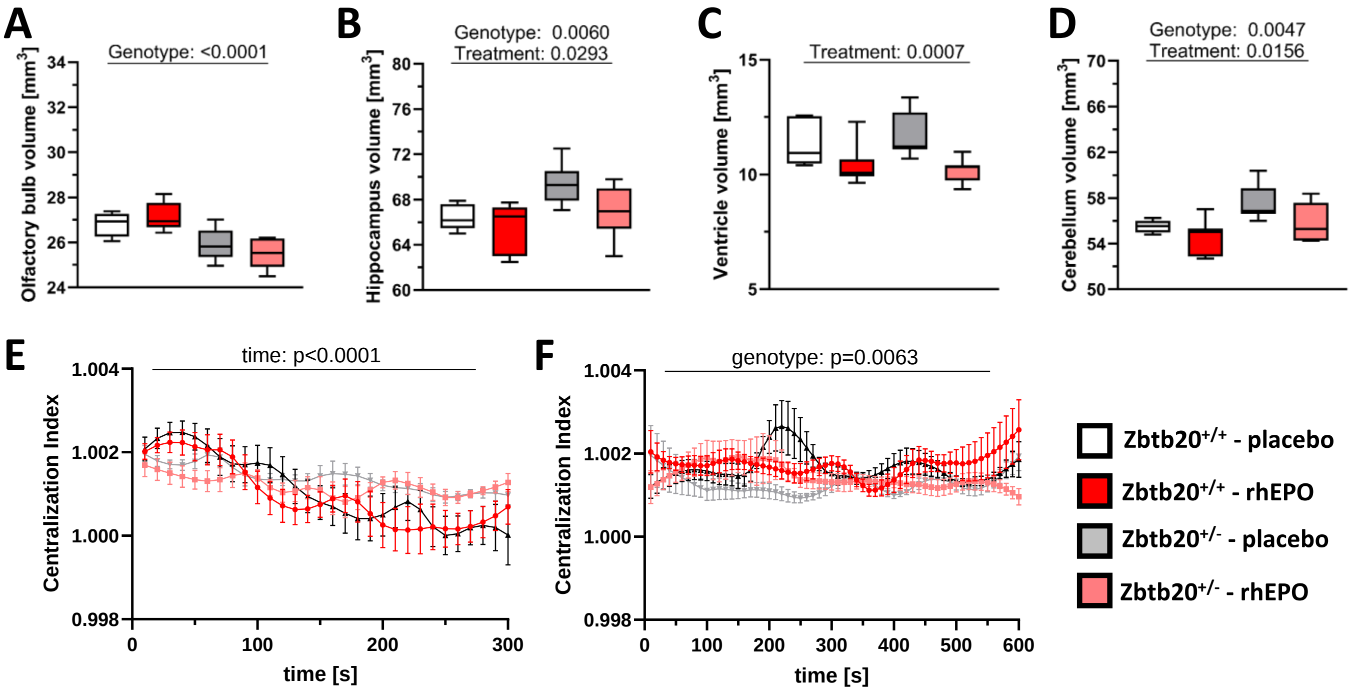

Supplementary Figure 3

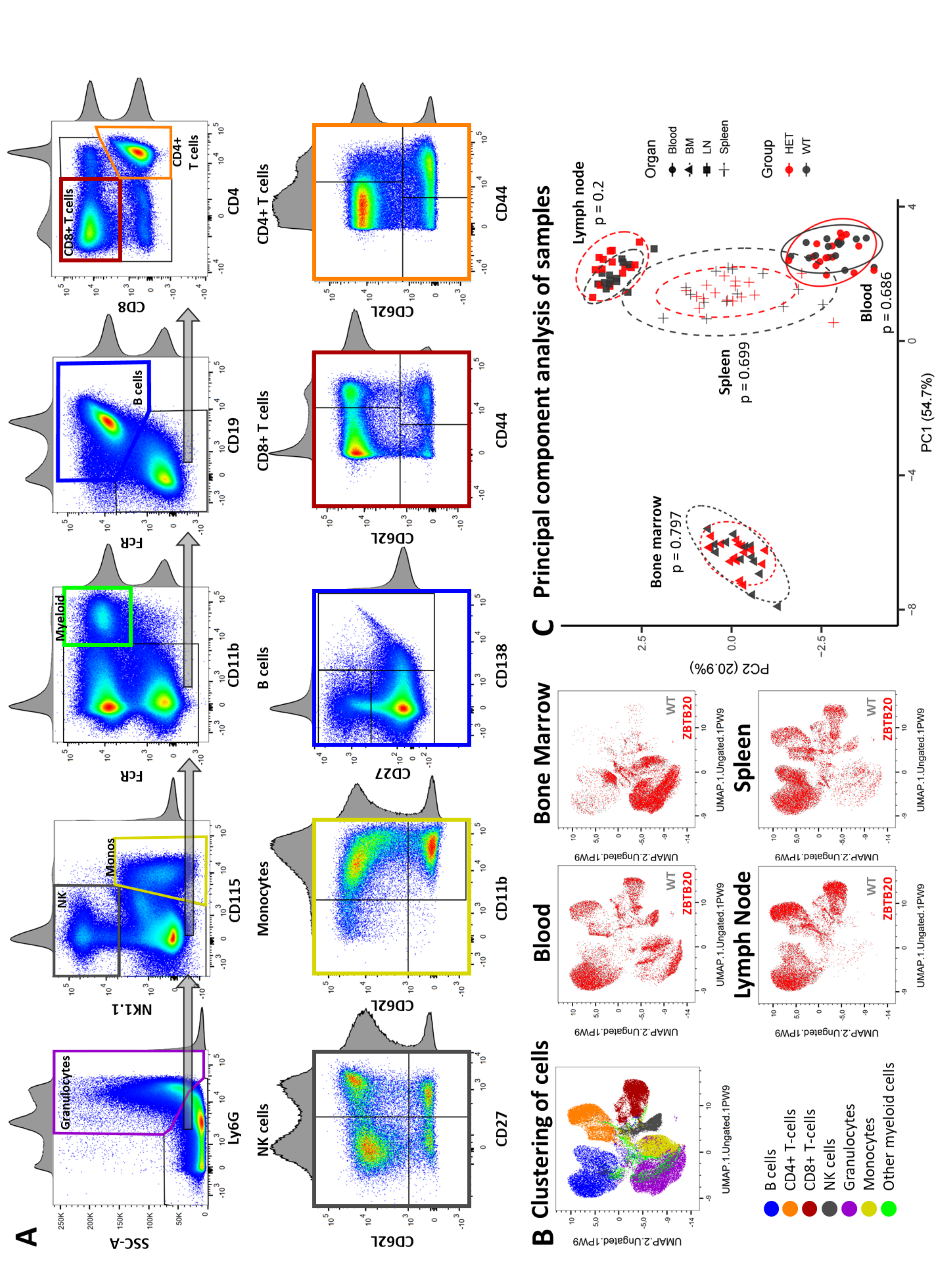

	Group 1		Group 2		Group 3		Group 4		Comparisons							
**1st Cohort Males**	**ZBTB20$^{+/+}$ placebo**		**ZBTB20$^{+/+}$ rhEPO**		**ZBTB20$^{+/-}$ placebo**		**ZBTB20$^{+/-}$ rhEPO**		**Group 1vs2**		**Group 1vs3**		**Group 2vs4**		**Group 3vs4**	
**Open Field**	mean ± SD	n	mean ± SD	n	mean ± SD	n	mean ± SD	n	test	p-value	test	p-value	test	p-value	test	p-value
Escape latency [s]	36,6 ± 28,1	13	28,5 ± 21,9	14	30,6 ± 28,1	15	38,0 ± 49,2	16	U	0,7836	U	0,5774	U	0,8455	U	0,9300
Track length total [cm]	3536,0 ± 736,4	13	4059,0 ± 589,8	14	4310,0 ± 1253,0	15	4175,0 ± 1285,0	16	t	0,0545	t	0,0549	t	0,7499	t	0,7688
Velocity avg [cm/s]	8,4 ± 1,8	13	9,7 ± 1,4	14	10,3 ± 3,0	15	9,9 ± 3,1	16	t	0,0545	t	0,0550	t	0,7496	t	0,7694
Zone crossings [#]	606,5 ± 96,0	13	656,4 ± 69,8	14	688,7 ± 136,3	15	670,9 ± 149,6	16	t	0,1386	t	0,0741	t	0,7329	t	0,7306
Periphery - time within [s]	290,2 ± 45,5	13	301,6 ± 33,9	14	329,4 ± 42,5	15	321,7 ± 40,6	16	t	0,4692	t	**0,0275**	t	0,1519	t	0,6162
Periphery - time within [s]	2way ANOVA Genotype: p=**0.0085** Treatment: p=0.9090															
Periphery - track length within [cm]	1870,0 ± 528,2	13	2342,0 ± 494,3	14	2791,0 ± 971,2	15	2593,0 ± 942,3	16	t	**0,0248**	t	**0,0044**	t	0,3623	t	0,5682
Periphery - velocity avg within [cm/s]	6,5 ± 1,6	13	7,8 ± 1,4	14	8,5 ± 2,7	15	8,1 ± 2,9	16	t	**0,0357**	t	**0,0248**	t	0,6910	t	0,7244
Periphery - visits to [#]	30,0 ± 9,6	13	31,4 ± 8,4	14	26,0 ± 10,2	15	29,5 ± 9,9	16	U	0,6929	t	0,2953	U	0,8137	t	0,3415
Intermediate - time within [s]	104,4 ± 37,5	13	97,6 ± 28,2	14	71,6 ± 31,1	15	80,2 ± 29,1	16	U	0,3754	t	**0,0198**	U	0,1306	t	0,4332
Intermediate - time within [s]	2way ANOVA Genotype: p=**0.0040** Treatment: p=0.8630															
Intermediate - track length within [cm]	1386,0 ± 411,4	13	1489,0 ± 362,6	14	1254,0 ± 518,3	15	1347,0 ± 526,5	16	t	0,4970	t	0,4617	t	0,3930	t	0,6250
Intermediate - velocity avg within [cm/s]	13,7 ± 2,1	13	15,5 ± 2,4	14	18,5 ± 5,0	15	16,9 ± 3,4	16	t	**0,0495**	t	**0,0033**	t	0,2128	t	0,3167
Intermediate - visits to [#]	42,9 ± 13,1	13	41,9 ± 10,5	14	37,5 ± 15,3	15	40,1 ± 14,5	16	t	0,8181	t	0,3184	t	0,6989	t	0,6326
Center - time within [s]	25,4 ± 13,8	13	20,7 ± 10,6	14	19,1 ± 14,7	15	18,1 ± 13,4	16	U	0,4020	U	0,2305	U	0,2939	U	0,8530
Center - time within [s]	2way ANOVA Genotype: p=0.2078 Treatment: p=0.4447															
Center - track length within [cm]	280,5 ± 121,7	13	228,4 ± 73,0	14	264,2 ± 189,3	15	234,9 ± 157,9	16	t	0,1966	t	0,7867	U	0,4726	U	0,7405
Center - velocity avg within [cm/s]	11,7 ± 3,5	13	12,4 ± 3,7	14	16,6 ± 6,0	15	14,7 ± 5,6	16	t	0,6437	t	**0,0139**	U	0,3084	U	0,2597
Center - visits to [#]	12,5 ± 5,4	13	10,4 ± 4,1	14	11,1 ± 8,7	15	10,3 ± 5,8	16	t	0,2507	U	0,2667	t	0,9534	U	0,9762
**Hole Board**																
Latency to first visit [s]	52,5 ± 68,0	11	23,7 ± 15,0	11	40,4 ± 52,3	15	39,2 ± 44,6	16	U	0,7969	U	0,8384	U	0,7160	U	0,9224
Visits [#] - total	16,9 ± 8,0	11	26,9 ± 14,1	11	14,0 ± 7,5	15	17,0 ± 6,7	16	t	0,0581	t	0,3598	t	**0,0491**	t	0,2509
Visits [#] - most consecutive	1,5 ± 0,9	11	2,5 ± 1,4	11	1,7 ± 1,2	15	1,7 ± 0,9	16	U	0,0688	U	0,6225	U	0,0774	U	0,9376
Visits [#] - total revisits	3,8 ± 3,0	11	7,3 ± 5,9	11	4,2 ± 3,9	15	3,9 ± 2,2	16	U	0,1171	t	0,7821	U	0,0993	t	0,7807
Visits (%) - rigorous revisits	28,5 ± 13,1	11	32,7 ± 12,4	11	15,9 ± 11,4	15	27,8 ± 12,9	16	t	0,4509	t	**0,0180**	t	0,3290	t	**0,0104**
Visits (%) - rigorous revisits	2way ANOVA Genotype: p=**0.0148** Treatment: p=**0.0139**															
**Y-Maze**																
Entries [#] - total	13,5 ± 7,0	13	19,9 ± 4,2	14	11,8 ± 5,1	15	11,7 ± 3,0	16	t	**0,0109**	t	0,4661	t	**<0.0001**	t	0,9415
Entries [#] - alternating	9,3 ± 4,5	13	12,9 ± 3,5	14	6,7 ± 3,6	15	7,6 ± 1,7	16	t	**0,0362**	t	0,1227	t	**<0.0001**	t	0,3583
Entries [#] - non-alternating	4,4 ± 2,8	13	6,0 ± 2,9	14	4,1 ± 2,2	15	3,1 ± 2,3	16	U	0,1691	U	0,8911	t	**0,0056**	U	0,2859
Entries [#] - alternating vs non-alternating	U p=**0.0224**		t p**<0.0001**		U p=**0.0227**		t p**<0.0001**									
Entries [%] - Δ alternating vs non-alternating	28,5 ± 42,7	13	37,2 ± 28,4	14	17,2 ± 37,7	15	47,6 ± 30,7	16	U	0,8496	U	0,1555	t	0,3411	U	**0,0146**
Entries [%] - Δ alternating vs non-alternating	2way ANOVA Genotype: p=0.9820 Treatment: p=**0.0314**															
Centralization Index - 0-100s	1,002 ± 0,0017	130	1,002 ± 0,0012	140	1,002 ± 0,0017	150	1,001 ± 0,0011	160	U	0,1615	U	0,1595	U	**<0.0001**	U	**<0.0001**
Centralization Index - 100-200s	1,000 ± 0,0018	130	1,001 ± 0,0012	140	1,001 ± 0,0022	150	1,000 ± 0,0013	160	U	**0,0039**	U	0,8736	U	**<0.0001**	U	0,2401
Centralization Index - 200-300s	1,001 ± 0,0013	130	1,001 ± 0,0015	140	1,001 ± 0,0016	150	1,001 ± 0,0012	160	U	**0,0023**	U	**0,0071**	U	**<0.0001**	U	**0,0004**
Centralization Index - 0-300s	Three-way ANOVA Time: p**<0.0001** Treatment: p=0.9158 Genotype: p=0.3957															
**Social Interaction in Pairs**																
Track length total [m]	33,3 ± 3,7	10	31,1 ± 2,9	12	38,2 ± 3,8	10	42,3 ± 4,3	14	U	0,7223	U	**0,0089**	U	**<0.0001**	U	**0,0358**
Distance nose-nose [cm] - avg over full time (10s)	Three-way ANOVA Time: p=0.3097 Treatment: p=0.1575 Genotype: p=0.2851															
Distance nose-nose [cm] - total avg	16,83 ± 0,97	5	16,68 ± 0,94	6	16,93 ± 2,05	5	15,50 ± 0,74	7	t	0,8048	t	0,9274	t	**0,0336**	t	0,2006
Distance nose-nose [cm] - avg over 1. minute (2s)	Three-way ANOVA Time: p=0.4573 Treatment: p=0.4423 Genotype: p=0.0562															
Distance nose-nose [cm] - 1. minute avg	17,7 ± 1,4	5	17,5 ± 1,9	6	16,7 ± 2,1	5	15,7 ± 1,0	7	t	0,9010	t	0,4017	t	0,0804	t	0,3868
Distance nose-nose [cm] - avg over 3 minutes (10s)	Three-way ANOVA Time: p=0.6036 Treatment: p=0.182 Genotype: p=0.2542															
Distance nose-nose [cm] - 1. minute avg	17,3 ± 1,1	5	17,2 ± 1,5	6	17,3 ± 2,1	5	15,7 ± 1,0	7	t	0,8863	t	>0.9999	U	0,1807	U	0,2677
**Three Chamber Sociability Test**																
Social preference (SP) - overall distance [cm]	4105,0 ± 408,1	13	3698,0 ± 557,9	14	4433,0 ± 1056,0	15	5057,0 ± 851,4	16	t	**0,0399**	t	0,2815	t	**<0.0001**	t	0,0819
SP - zone crossings [#]	566,8 ± 57,3	13	536,3 ± 61,8	14	635,1 ± 125,5	15	682,8 ± 105,6	16	t	0,1954	t	0,0727	t	**<0.0001**	t	0,2646
SP - time in stimulus chamber [s]	113,3 ± 28,8	13	109,0 ± 18,7	14	113,7 ± 51,2	15	137,7 ± 43,5	16	t	0,6522	U	0,4130	t	**0,0262**	U	0,2995
SP - time in empty chamber [s]	77,3 ± 14,5	13	73,0 ± 18,2	14	98,7 ± 41,6	15	101,4 ± 24,8	16	t	0,4998	U	0,1077	t	**0,0012**	U	0,5196
SP - time in stimulus vs empty chamber [s]	t p=**0.0008**		t p**<0.0001**		U p=0.1261		t p=**0.0080**									
SP - track length in stimulus chamber [cm]	1002,0 ± 208,4	13	877,0 ± 160,6	14	947,0 ± 499,5	15	1160,0 ± 410,7	16	t	0,0962	U	0,7168	t	**0,0192**	U	0,3183
SP - track length in empty chamber [cm]	845,5 ± 167,2	13	750,6 ± 172,7	14	1055,0 ± 348,4	15	1180,0 ± 292,1	16	t	0,1596	t	0,0511	t	**<0.0001**	t	0,2898
SP - track length in stimulus vs empty chamber [cm]	t p=**0.0458**		t p=0.0555		U p>0.9999		t p=0.8768									
SP - visits to stimulus chamber [#]	45,2 ± 9,2	13	39,7 ± 8,4	14	49,2 ± 27,0	15	48,8 ± 16,2	16	t	0,1218	t	0,5920	t	0,0615	t	0,9620
SP - visits to empty chamber [#]	31,7 ± 8,1	13	27,0 ± 8,0	14	45,1 ± 18,5	15	46,7 ± 13,0	16	t	0,1432	t	**0,0198**	t	**<0.0001**	t	0,7815
SP - visits to stimulus vs empty chamber [#]	t p=**0.0006**		t p=**0.0004**		t p=0.6288		t p=0.6856									
SP - latency to stimulus chamber [s]	35,6 ± 47,2	13	29,4 ± 38,6	14	20,6 ± 26,7	15	33,4 ± 42,2	16	U	0,7658	U	0,3874	U	0,7364	U	0,5453
SP - latency to empty chamber [s]	75,2 ± 64,3	13	129,2 ± 123,5	14	88,3 ± 75,7	15	53,4 ± 50,4	16	t	0,1651	t	0,6241	U	0,1417	U	0,2164
SP - latency to stimulus vs empty chamber [s]	U p=0.1254		U p=**0.0122**		U p=**0.0014**		U p=0.1713									
SP - time with stimulus insert [s]	205,2 ± 35,7	13	230,2 ± 52,1	14	151,9 ± 86,7	15	126,6 ± 55,1	16	t	0,1565	U	0,0957	t	**<0.0001**	U	0,2129
SP - time with empty insert [s]	131,3 ± 44,0	13	110,7 ± 39,3	14	128,0 ± 98,1	15	94,8 ± 45,1	16	t	0,2136	U	0,1696	t	0,3088	U	0,4701
SP - time with stimulus vs empty insert [s]	t p**<0.0001**		t p**<0.0001**		U p=0.1045		t p=0.0844									
SP - visits to stimulus insert [#]	30,9 ± 5,6	13	27,7 ± 7,6	14	33,7 ± 19,9	15	26,8 ± 11,3	16	t	0,2353	U	0,1045	t	0,7978	U	0,1317
SP - visits to empty insert [#]	20,9 ± 6,5	13	17,6 ± 4,5	14	28,1 ± 13,0	15	25,9 ± 9,8	16	t	0,1527	t	0,0716	t	**0,0063**	t	0,6020
SP - visits to stimulus vs empty insert [#]	t p=**0.0003**		t p=**0.0004**		U p=0.1133		t p=0.8035									
SP - latency to stimulus insert [s]	50,3 ± 49,0	13	51,0 ± 51,0	14	43,8 ± 54,1	15	59,9 ± 59,0	16	U	0,9810	U	0,7757	U	0,7587	U	0,4400
SP - latency to empty insert [s]	80,8 ± 64,3	13	141,9 ± 118,6	14	92,9 ± 74,5	15	58,3 ± 50,4	16	t	0,1083	t	0,6487	t	**0,0254**	t	0,1459
SP - latency to stimulus vs empty insert [s]	U p=0.3107		U p=**0.0310**		U p=**0.0290**		U p=0.8672									
SP - time spent in center [s]	72,8 ± 21,0	13	77,0 ± 34,3	14	107,7 ± 64,1	15	139,5 ± 33,1	16	t	0,7025	U	0,0581	t	**<0.0001**	U	**0,0031**
SP - track length within center [cm]	962,8 ± 274,7	13	862,1 ± 271,8	14	1292,0 ± 387,3	15	1732,0 ± 374,4	16	t	0,3480	U	**0,0222**	t	**<0.0001**	U	**0,0072**
SP - visits to center [#]	24,9 ± 7,2	13	21,1 ± 7,8	14	32,1 ± 12,0	15	42,5 ± 10,0	16	t	0,2029	t	0,0626	t	**<0.0001**	t	**0,0141**
Centralization Index - Habituation	Three-way ANOVA Time: p**<0.0001** Treatment: p=0.2432 Genotype: p=0.1597															
Centralization Index - Social Preference	Three-way ANOVA Time: p=0.1683 Treatment: p=0.0879 Genotype: p=0.4223															
**Marble Burying**																
Marbles burried - total [%]	47,8 ± 25,4	13	39,9 ± 20,2	14	7,5 ± 9,7	15	9,1 ± 11,3	16	t	0,3846	U	**<0.0001**	U	**<0.0001**	U	0,5195
Marbles burried - total [%]	2way ANOVA Genotype: p**<0.0001** Treatment: p=0.2973															
Marbles burried - center [%]	38,5 ± 32,1	13	39,3 ± 28,1	14	3,3 ± 7,4	15	3,9 ± 8,8	16	t	0,9441	U	**0,0005**	U	**<0.0001**	U	>0.9999
Marbles burried - periphery [%]	52,4 ± 25,2	13	40,2 ± 20,8	14	9,6 ± 12,2	15	11,7 ± 13,3	16	t	0,1837	U	**<0.0001**	U	**0,0003**	U	0,4657
**Nest Building Test**																
Nest Building Score [AU]	4,1 ± 0,4	13	4,1 ± 0,4	14	3,9 ± 0,7	15	3,6 ± 0,9	16	U	0,8129	U	0,3625	U	**0,0247**	U	0,2628
Nest Building Score [AU]	2way ANOVA Genotype: p=**0.0255** Treatment: p=0.4949															
**LABORAS**																
Climbing - duration [s]	4562,0 ± 1383,0	13	4815,0 ± 3256,0	14	6439,0 ± 3715,0	15	7858,0 ± 4652,0	16	t	0,7935	t	0,0856	t	**0,0458**	t	0,3546
Climbing - duration [s]	2way ANOVA Genotype: p=**0.0099** Treatment: p=0.3485															
Climbing - occurences [#]	190,3 ± 68,7	13	154,5 ± 82,2	14	398,4 ± 548,6	15	404,3 ± 270,6	16	t	0,2298	U	0,1963	U	**0,0033**	U	0,3527
Climbing - occurences [#]	2way ANOVA Genotype: p=**0.0008** Treatment: p=0.2208															
Locomotion - duration [s]	2414,0 ± 682,6	13	2067,0 ± 547,7	14	2357,0 ± 792,8	15	2413,0 ± 683,6	16	t	0,1614	t	0,8397	U	0,2082	U	0,7405
Locomotion - occurences [#]	4043,0 ± 1183,0	13	3586,0 ± 1044,0	14	3789,0 ± 1326,0	15	3860,0 ± 1048,0	16	t	0,2983	t	0,5960	t	0,4791	t	0,8700
Immobility - duration [min]	246,2 ± 68,7	13	238,5 ± 62,7	14	264,4 ± 86,3	15	230,6 ± 91,8	16	t	0,7639	t	0,5399	t	0,7824	t	0,2986
Immobility - occurences [#]	1075,0 ± 219,6	13	1174,0 ± 409,6	14	1003,0 ± 524,1	15	884,0 ± 161,0	16	U	0,6160	U	0,1184	U	**0,0104**	U	0,7405
Immobility - occurences [#] pooled by genotype	U p=0.0026															
Rearing - duration [s]	1979,0 ± 598,0	13	2477,0 ± 686,4	14	1671,0 ± 654,1	15	1887,0 ± 752,0	16	t	0,0547	t	0,2050	t	**0,0327**	t	0,4001
Rearing - occurences [#]	1250,0 ± 409,1	13	1310,0 ± 347,2	14	1103,0 ± 448,3	15	1170,0 ± 338,5	16	t	0,6860	t	0,3710	t	0,2754	t	0,6404
Grooming - duration [s]	2087,0 ± 491,0	13	1922,0 ± 690,5	14	1721,0 ± 626,0	15	1919,0 ± 630,3	16	t	0,4811	t	0,0956	t	0,9893	t	0,3874
Grooming - occurences [#]	521,7 ± 147,7	13	459,2 ± 200,4	14	395,2 ± 192,3	15	457,4 ± 176,0	16	t	0,3634	U	**0,0335**	U	0,9092	U	0,1784
Drinking - duration [s]	45,8 ± 53,3	13	57,7 ± 55,2	14	29,5 ± 33,8	15	19,8 ± 18,3	16	U	0,6500	U	0,2945	U	**0,0344**	U	0,4232
Drinking - occurences [#]	24,3 ± 31,8	13	31,6 ± 29,9	14	16,3 ± 21,1	15	12,6 ± 13,0	16	U	0,6073	U	0,2392	U	**0,0458**	U	0,7623
Eating - duration [s]	1439,0 ± 966,9	13	1154,0 ± 737,2	14	1002,0 ± 738,7	15	1746,0 ± 1252,0	16	U	0,4879	U	0,3389	t	0,1218	U	0,0717
Eating - occurences [#]	416,4 ± 275,2	13	296,5 ± 151,2	14	390,5 ± 325,5	15	566,9 ± 433,8	16	t	0,1818	U	0,7079	U	0,0789	U	0,2769
Scratching - duration [s]	455,5 ± 173,9	13	408,2 ± 245,5	14	417,4 ± 236,7	15	446,8 ± 202,4	16	U	0,3754	t	0,6281	U	0,2237	U	0,5717
Scratching - occurences [#]	503,3 ± 168,9	13	471,8 ± 226,3	14	457,0 ± 234,6	15	479,4 ± 171,3	16	U	0,5912	t	0,5507	U	0,4475	t	0,7647
Velocity - maximum [mm/s]	203,1 ± 23,7	13	202,1 ± 9,1	14	196,1 ± 38,2	15	193,0 ± 25,5	16	U	0,9324	t	0,5607	U	0,5799	t	0,7961
Velocity - duration of maximum speed [min]	746,9 ± 21,2	13	743,0 ± 17,5	14	757,1 ± 17,7	15	749,3 ± 21,8	16	t	0,6135	t	0,1827	U	0,3338	U	0,2814
Velocity - average speed [mm/s]	71,5 ± 3,7	13	72,8 ± 2,3	14	69,9 ± 7,3	15	68,6 ± 7,7	16	t	0,2808	t	0,4826	t	0,0507	t	0,6204
Track length [m]	149,3 ± 42,9	13	135,2 ± 42,1	14	147,3 ± 47,7	15	151,2 ± 48,6	16	t	0,3960	t	0,9065	t	0,3409	t	0,8219

Circling - events clockwise [#]	505,6 ± 216,2	13	498,9 ± 238,8	14	484,3 ± 216,4	15	486,1 ± 181,5	16	t	0,9397	t	0,7972	t	0,8715	t	0,9803
Circling - events counterclockwise [#]	509,4 ± 172,1	13	500,6 ± 232,5	14	551,8 ± 325,8	15	491,0 ± 173,1	16	t	0,9121	U	0,9375	t	0,8997	U	0,8529
Circling - total events [#]	1015,0 ± 374,6	13	999,6 ± 460,4	14	1036,0 ± 532,5	15	977,1 ± 345,1	16	t	0,9244	t	0,9034	t	0,8825	t	0,7194
Circling - average duration [s]	3,6 ± 0,1	13	3,6 ± 0,2	14	3,7 ± 0,2	15	3,7 ± 0,1	16	t	0,2496	t	0,3818	t	0,0950	t	0,8358
Circling - average duration [s] pooled by genotype	U p=0.0781															
Circling - average perimeter [mm]	165,5 ± 25,9	13	163,9 ± 18,7	14	172,0 ± 26,1	15	177,5 ± 16,3	16	U	0,5502	U	0,3162	U	**0,0079**	U	0,2020
Circling - average velocity [mm/s]	52,1 ± 7,2	13	52,7 ± 4,0	14	53,7 ± 10,3	15	55,0 ± 6,3	16	t	0,7970	U	0,8207	t	0,2316	U	0,2164
**Vocalization**																
Latency to first call [s]	50,2 ± 65,5	13	18,0 ± 10,3	14	96,3 ± 81,4	15	57,2 ± 63,0	16	U	0,2435	U	0,0689	U	**0,0018**	U	0,3650
Latency to first call [s]	2way ANOVA Genotype: p=**0.0106** Treatment: p=**0.0295**															
Number of calls [#]	420,2 ± 346,7	13	606,4 ± 219,0	14	56,8 ± 98,9	15	76,7 ± 124,9	16	t	0,1139	U	**0,0028**	U	**<0.0001**	U	0,2948
Number of calls [#]	2way ANOVA Genotype: p**<0.0001** Treatment: p=0.0904															
Duration of calling [s]	1305,0 ± 12,5	13	17,6 ± 8,4	14	1,3 ± 2,5	15	1,7 ± 2,6	16	t	0,2876	U	**0,0032**	U	**<0.0001**	U	0,2948
Duration of calling [s]	2way ANOVA Genotype: p**<0.0001** Treatment: p=0.2444															
**Prepulse Inhibition (PPI)**																
Hearing - 65 dB [AU]	0,6 ± 0,2	10	1,8 ± 1,7	9	1,0 ± 0,8	9	0,5 ± 0,2	9	U	0,1500	U	0,3050	U	**0,0150**	U	**0,0152**
Hearing - 120 dB [AU]	7,8 ± 4,5	10	4,8 ± 2,3	9	6,1 ± 4,2	9	4,4 ± 4,2	9	t	0,0911	t	0,4061	U	0,3865	U	0,1903
Hearing - 65 dB vs 120 dB	t p=**0.0008**		U p=**0.0106**		U p=**0.0002**		U p**<0.0001**									
PPI - 70dB [%]	29,3 ± 10,6	10	17,6 ± 13,3	9	38,5 ± 9,0	9	34,1 ± 17,2	9	t	0,0527	t	0,0551	t	**0,0375**	t	0,5090
PPI - 75dB [%]	39,2 ± 18,5	10	35,5 ± 15,0	9	57,0 ± 17,0	9	56,2 ± 11,8	9	t	0,6390	t	**0,0431**	t	**0,0054**	t	0,9108
PPI - 80dB [%]	55,8 ± 16,3	10	51,0 ± 17,7	9	57,0 ± 20,9	9	57,9 ± 13,5	9	t	0,5502	t	0,8850	t	0,3670	t	0,9204
PPI one-way/2way ANOVA sound intensity	**0,0026**		**0,0005**		**0,0362**		**0,0026**		2wA	0,1079	2wA	**0,0298**	2wA	**0,0007**	2wA	0,7319
PPI one-way/2way ANOVA sound intensity	Three-way ANOVA Sound Intensity: p**<0.0001** Treatment: p=0.3300 Genotype: p=**0.0061**															
**Morris Water Maze**																
Visible 1 - escape latency [s]	22,8 ± 12,0	13	21,6 ± 16,50	14	21,0 ± 9,7	15	25,0 ± 14,8	16	U	0,3022	U	0,8919	U	0,1794	U	0,3791
Visible 2 - escape latency [s]	7,4 ± 4,1	13	7,9 ± 4,40	14	7,6 ± 4,9	15	7,4 ± 4,4	16	U	0,6500	U	0,9639	U	0,9510	U	0,9224
Visible 1 vs 2 - escape latency [s]	U p**<0.0001**		U p=**0.0004**		U p**<0.0001**		U p**<0.0001**									
Visible 1 - track length [cm]	370,5 ± 153,9	13	360,3 ± 206,9	14	412,0 ± 165,5	15	529,0 ± 300,7	16	U	0,5502	t	0,4989	U	**0,0275**	U	0,2020
Visible 2 - track length [cm]	140,9 ± 72,1	13	135,3 ± 63,6	14	172,0 ± 103,8	15	179,0 ± 99,5	16	t	0,8336	U	0,5250	U	0,1661	U	0,6260
Visible 1 vs 2 - track length [cm]	t p=**0.0001**		U p**<0.0001**		U p**<0.0001**		U p**<0.0001**									
Visible 1 - velocity [cm/s]	17,7 ± 3,5	13	18,4 ± 3,0	14	21,3 ± 2,6	15	22,2 ± 2,4	16	t	0,5747	t	**0,0049**	t	**0,0008**	t	0,3442
Visible 2 - velocity [cm/s]	20,8 ± 4,0	13	19,8 ± 4,0	14	24,2 ± 4,1	15	26,1 ± 3,3	16	t	0,5319	t	**0,0364**	t	**<0.0001**	t	0,1568
Visible 1 vs 2 - velocity [cm/s]	t p=**0.0422**		t p=0.2874		t p=**0.0324**		t p=**0.0006**									
Hidden 1 - escape latency [s]	52,3 ± 21,4	13	44,6 ± 22,1	14	51,1 ± 15,6	15	54,2 ± 21,2	16	t	0,3664	t	0,8628	t	0,2360	t	0,6369
Hidden 2 - escape latency [s]	41,1 ± 25,9	13	27,9 ± 21,5	14	46,5 ± 20,6	15	43,9 ± 19,7	16	U	0,0945	t	0,5472	U	**0,0218**	t	0,6823
Hidden 3 - escape latency [s]	34,9 ± 22,1	13	29,8 ± 14,9	14	35,2 ± 20,4	15	35,2 ± 16,9	16	t	0,4901	t	0,9732	t	0,3613	t	0,9992
Hidden 4 - escape latency [s]	26,8 ± 13,8	13	27,9 ± 14,9	14	27,4 ± 19,4	15	29,8 ± 20,1	16	U	0,7203	U	0,4956	U	0,7901	U	0,6539
Hidden 5 - escape latency [s]	16,3 ± 9,2	13	29,0 ± 17,2	14	27,4 ± 20,7	15	26,3 ± 14,5	16	U	0,0683	U	0,1300	U	0,7277	U	0,8304
Hidden 6 - escape latency [s]	17,8 ± 6,2	13	15,4 ± 9,1	14	21,8 ± 14,0	15	25,6 ± 14,2	16	t	0,4325	t	0,3328	t	**0,0254**	t	0,4537
Hidden 7 - escape latency [s]	19,1 ± 10,6	13	14,7 ± 8,6	14	17,0 ± 11,6	15	20,7 ± 16,7	16	U	0,2020	U	0,4130	U	0,3134	U	0,5717
Hidden 8 - escape latency [s]	13,8 ± 6,5	13	10,8 ± 3,0	14	22,1 ± 13,6	15	20,2 ± 15,8	16	t	0,1533	t	**0,0457**	U	0,0925	U	0,3846
Hidden 1-8 - escape latency over time	Three-way ANOVA Time: p**<0.0001** Treatment: p=0.7100 Genotype: p=**0.0387**															
Probe Trial 1 (PT) - time in target quadrant (TQ) [s]	45,6 ± 9,4	13	39,9 ± 10,1	14	28,5 ± 7,1	15	24,9 ± 7,7	16	t	0,1445	t	**<0.0001**	t	**0,0001**	t	0,1886
Probe Trial 1 (PT) - time in target quadrant (TQ) [s]	2way ANOVA Genotype: p**<0.0001** Treatment: p=0.0747															
PT 1 - visits to TQ [#]	16,8 ± 3,7	13	16,5 ± 3,3	14	13,9 ± 2,7	15	12,3 ± 3,5	16	t	0,8419	U	**0,0402**	t	**0,0022**	U	0,1877
PT 1 - latency to former escape platform [s]	15,9 ± 10,7	13	21,1 ± 12,7	14	19,9 ± 12,6	15	27,9 ± 23,4	16	U	0,3022	U	0,3389	U	0,6886	t	0,2426
PT 1 - visits to former escape platform [#]	5,0 ± 2,0	13	4,6 ± 2,3	14	3,7 ± 1,5	15	2,8 ± 1,9	16	t	0,6069	U	**0,0416**	t	**0,0330**	U	0,1667
PT 1 - total track length [cm]	1775,0 ± 270,2	13	1847,0 ± 167,6	14	2058,0 ± 231,0	15	2089,0 ± 103,2	16	U	0,7203	t	**0,0070**	U	**<0.0001**	t	0,6354
PT 1 - total track length [cm]	2way ANOVA Genotype: p**<0.0001** Treatment: p=0.3388															
Reversal 1 - escape latency [s]	25,4 ± 18,3	13	23,3 ± 8,9	14	26,7 ± 16,3	15	33,3 ± 17,6	16	U	0,7930	U	0,6501	t	0,0576	U	0,2995
Reversal 2 - escape latency [s]	22,1 ± 17,1	13	14,2 ± 7,1	14	23,2 ± 16,2	15	23,0 ± 14,1	16	U	0,2588	U	0,7605	U	0,1103	U	0,8916
Reversal 3 - escape latency [s]	14,2 ± 10,9	13	16,4 ± 12,2	14	28,5 ± 26,3	15	20,6 ± 13,4	16	U	0,9051	U	**0,0464**	U	0,1934	U	0,7112
Reversal 4 - escape latency [s]	11,4 ± 6,4	13	12,9 ± 9,2	14	19,4 ± 13,5	15	19,8 ± 11,0	16	U	>0.9999	U	0,0581	U	**0,0245**	U	0,7405
Reversal 1-4 - escape latency over time // Gen./Treat.	p=**0.0476**		p=**0.0265**		p=0.5629		p=**0.0339**		2wA	0,5294	2wA	0,1676	2wA	**0,0044**	2wA	0,9425
Reversal 1-4 - escape latency over time	Three-way ANOVA Time: p=**0.0005** Treatment: p=0.7062 Genotype: p=**0.0073**															
Probe Trial 2 (PT) - time in target quadrant (TQ) [s]	34,4 ± 5,5	13	38,5 ± 7,1	14	28,3 ± 6,1	15	28,7 ± 7,2	16	t	0,1036	t	**0,0105**	t	**0,0008**	t	0,8792
Probe Trial 2 (PT) - time in target quadrant (TQ) [s]	2way ANOVA Genotype: p**<0.0001** Treatment: p=0.2260															
PT 2 - visits to TQ [#]	18,5 ± 3,8	13	18,1 ± 3,7	14	14,3 ± 3,3	15	12,8 ± 3,2	16	t	0,8262	t	**0,0054**	U	**0,0005**	U	0,1390
PT 2 - latency to former escape platform [s]	10,2 ± 11,3	13	11,0 ± 18,5	14	18,3 ± 17,7	15	15,3 ± 13,3	16	U	0,9430	U	0,1452	U	0,0701	U	0,6618
PT 2 - visits to former escape platform [#]	5,2 ± 2,9	13	5,5 ± 2,3	14	3,5 ± 1,6	15	2,3 ± 1,3	16	t	0,7337	t	0,0769	t	**0,0002**	t	**0,0368**
PT 2 - total track length [cm]	1854,0 ± 282,7	13	1841,0 ± 146,3	14	2114,0 ± 193,0	15	2083,0 ± 158,4	16	t	0,8869	t	**0,0108**	t	**0,0002**	t	0,6313
PT 2 - total track length [cm]	2way ANOVA Genotype: p**<0.0001** Treatment: p=0.6671															
**Complex Wheel Running**																
Distance [m] - over time	Three-way ANOVA Time: p=**0.0031** Treatment: p=0.5696 Genotype: p=**0.0007**															
Distance [m] - total	911,1 ± 602,53	6	806,1 ± 211,69	5	1614,7 ± 167,35	6	1511,8 ± 480,3	8	t	0,7033	t	**0,0344**	t	**0,0044**	t	0,5876
Time [min] - total	69,48 ± 40,7	6	66,13 ± 17,78	5	117 ± 12,02	6	102,9 ± 31,05	8	t	0,8606	t	**0,0343**	t	**0,0202**	t	0,2684
Velocity [m/s] - average	0,210 ± 0,029	6	0,205 ± 0,018	5	0,243 ± 0,021	6	0,257 ± 0,021	8	t	0,7140	t	**0,0496**	t	**0,0007**	t	0,2334
**Magnet Resonance Tomography**																
Olfactory bulb - volume [mm3]	27,12 ± 1,086	7	26,76 ± 0,314	8	26,63 ± 0,405	7	26,55 ± 0,744	8	t	0,4266	t	0,2971	t	0,4781	t	0,7973
Thalamus - volume [mm3]	17,04 ± 0,909	7	16,61 ± 0,360	8	17,16 ± 0,789	7	17,35 ± 0,902	8	t	0,2757	t	0,7953	U	0,083	U	0,3357
Hippocampus - volume [mm3]	65,04 ± 2,909	7	63,81 ± 1,632	8	68,8 ± 1,308	7	68,84 ± 2,469	8	t	0,3470	t	**0,0136**	t	**0,0004**	t	0,9664
Hippocampus - volume [mm3]	2way ANOVA Genotype: p**<0.0001** Treatment: p=0.4580															
Corpus Callosum - volume [mm3]	1,15 ± 0,068	7	1,126 ± 0,037	8	1,144 ± 0,080	7	1,183 ± 0,054	7	t	0,4327	t	0,8912	t	**0,0413**	t	0,3145
Ventricles - volume [mm3]	10,4 ± 1,505	7	9,719 ± 0,896	8	10,67 ± 0,926	7	10,52 ± 1,044	8	t	0,3205	t	0,6965	t	0,1224	t	0,7734
Cerebellum - volume [mm3]	54,32 ± 2,589	7	53,36 ± 1,817	8	56,68 ± 2,239	7	57,01 ± 1,18	8	t	0,4293	t	0,0936	t	**0,0005**	t	0,7345
Cerebellum - volume [mm3]	2way ANOVA Genotype: p=**0.0003** Treatment: p=0.6670															
Brain matter - volume [mm3]	494,5 ± 20,0	7	483,1 ± 10,53	8	511,8 ± 12,81	7	512,9 ± 8,849	8	t	0,2104	t	0,0812	t	**<0.0001**	t	0,8507
Brain matter - volume [mm3]	2way ANOVA Genotype: p**<0.0001** Treatment: p=0.3117															
Brain - volume [mm3]	504,9 ± 20,28	7	492,8 ± 10,67	8	522,5 ± 13,33	7	523,5 ± 8,882	8	t	0,1923	t	0,0825	t	**<0.0001**	t	0,8744
Brain - volume [mm3]	2way ANOVA Genotype: p**<0.0001** Treatment: p=0.2841															
**Organ Collection**																
Body weight [g]	29,43 ± 1,27	7	28,75 ± 1,83	8	31,50 ± 1,69	8	31,00 ± 1,20	8	t	0,4162	U	**0,0252**	U	**0,0093**	U	0,4915
Tibia length [mm]	17,78 ± 0,76	7	17,18 ± 2,13	8	18,79 ± 0,45	8	19,22 ± 0,53	8	U	>0.9999	U	**0,0081**	U	**0,0003**	U	0,0605
Brain - weight [g]	0,4406 ± 0,0125	7	0,4400 ± 0,0130	8	0,4615 ± 0,0206	8	0,4568 ± 0,0174	8	t	0,9240	t	**0,0337**	t	**0,0474**	t	0,6290
Brain - weight [g]	2way ANOVA Genotype: p=**0.0030** Treatment: p=0.6387															
Brain - dry weight [g]	0,0938 ± 0,0031	7	0,0937 ± 0,0034	8	0,0985 ± 0,0030	8	0,0977 ± 0,0046	8	t	0,9434	t	**0,0096**	t	0,0699	t	0,6582
Brain - dry weight [g]	2way ANOVA Genotype: p=**0.0018** Treatment: p=0.6900															
Brain - water content [g]	0,3469 ± 0,0097	7	0,3464 ± 0,0100	8	0,3630 ± 0,0189	8	0,3592 ± 0,0131	8	t	0,9199	t	0,0583	t	**0,0462**	t	0,6450
Brain - water content [g]	2way ANOVA Genotype: p=**0.0055** Treatment: p=0.6446															
Brain - weight [g]/Body weight [g]	0,0150 ± 0,0004	7	0,0154 ± 0,0011	8	0,0147 ± 0,0011	8	0,0148 ± 0,0008	8	t	0,3977	t	0,5297	t	0,2267	t	0,8997
Brain - weight [g]/Tibia length [mm]	0,0248 ± 0,0010	7	0,0261 ± 0,0043	8	0,0246 ± 0,0012	8	0,0238 ± 0,0011	8	U	0,7995	t	0,6892	U	0,1524	t	0,2005
Heart - weight [g]	0,1598 ± 0,0217	7	0,1541 ± 0,0218	8	0,1581 ± 0,0285	8	0,1554 ± 0,0156	8	t	0,6208	t	0,8978	t	0,8991	t	0,8136
Heart - dry weight [g]	0,0296 ± 0,0027	7	0,0283 ± 0,0036	8	0,0300 ± 0,0023	8	0,0296 ± 0,0025	8	t	0,4133	t	0,8178	U	0,4881	U	0,4895
Heart - water content [g]	0,1302 ± 0,0196	7	0,1259 ± 0,0191	8	0,1282 ± 0,0267	8	0,1258 ± 0,0136	8	t	0,6733	t	0,8695	t	0,9906	t	0,8231
Heart - weight [g]/Body weight [g]	0,0054 ± 0,0007	7	0,0054 ± 0,0007	8	0,0051 ± 0,0011	8	0,0050 ± 0,0006	8	U	0,5888	t	0,4217	U	0,2222	t	0,9346
Heart - weight [g]/Tibia length [mm]	0,0090 ± 0,0013	7	0,0091 ± 0,0015	8	0,0084 ± 0,0015	8	0,0081 ± 0,0009	8	t	0,9194	t	0,4168	t	0,1438	t	0,6308
Lungs - weight [g]	0,1752 ± 0,0310	7	0,1774 ± 0,0362	8	0,1715 ± 0,0175	8	0,1730 ± 0,0164	8	U	0,7789	t	0,7884	U	0,5737	t	0,8669
Lungs - dry weight [g]	0,0338 ± 0,0049	7	0,0315 ± 0,0040	8	0,0340 ± 0,0030	8	0,0348 ± 0,0021	8	t	0,3322	t	0,9373	t	0,0601	t	0,5419
Lungs - water content [g]	0,1414 ± 0,0262	7	0,1459 ± 0,0348	8	0,1375 ± 0,0159	8	0,1382 ± 0,0157	8	U	0,6943	t	0,7441	U	0,5919	t	0,9368
Lungs - weight [g]/Body weight [g]	0,0059 ± 0,0011	7	0,0062 ± 0,0013	8	0,0055 ± 0,0011	8	0,0056 ± 0,0005	8	U	0,9329	U	0,4140	U	0,3681	t	0,7360
Lungs - weight [g]/Tibia length [mm]	0,0099 ± 0,0017	7	0,0105 ± 0,0026	8	0,0091 ± 0,0009	8	0,0090 ± 0,0007	8	U	>0.9999	t	0,3393	U	0,3409	t	0,7823
**PCR**																
ZBTB20 normalized to beta actin	1,2 ± 0,7	4	0,8 ± 0,2	4	1,1 ± 0,4	4	1,0 ± 0,4	4	t	0,3329	t	0,8070	t	0,2925	t	0,9252
ZBTB20 normalized to hprt1	1,1 ± 0,4	4	0,8 ± 0,1	4	1,0 ± 0,3	4	1,0 ± 0,3	4	U	0,3429	t	0,9161	U	0,4857	t	0,9103
ZBTB20 normalized to beta actin and hprt1	1,1 ± 0,5	4	0,8 ± 0,1	4	1,0 ± 0,4	4	1,0 ± 0,4	4	t	0,3162	t	0,8651	t	0,2890	t	0,9143

2nd Cohort Males

**Neophobia**																
Track length - total [cm]	2164,0 ± 175,4	12	2117,0 ± 458,7	11	2610,0 ± 451,8	15	2749,0 ± 617,9	13	U	0,8801	t	**0,0024**	U	**0,0154**	t	0,5110
Track length - total [cm]	2way ANOVA Genotype: p=**0.0001** Treatment: p=0.6714															
Zone crossings - total [#]	337,9 ± 30,7	12	326,6 ± 68,2	11	381,4 ± 57,3	15	404,0 ± 76,9	13	t	0,6226	t	**0,0193**	t	**0,0160**	t	0,3932
Interaction time [s] - object 1	15,3 ± 4,8	12	14,9 ± 5,6	11	18,0 ± 4,6	15	17,1 ± 5,1	13	t	0,8414	t	0,1526	t	0,3397	t	0,6066
Interaction time [s] - object 2	17,0 ± 3,6	12	16,6 ± 6,6	11	21,3 ± 6,8	15	22,5 ± 8,4	13	t	0,8659	t	**0,0437**	t	0,0659	t	0,6930
Interaction time [s] - object 1 vs object 2	t p=0.3598		t p=0.5301		t p=0.1322		t p=0.0601									
Interaction time [s] - object 1 + object 2	32,3 ± 6,5	12	31,5 ± 11,3	11	39,4 ± 10,3	15	39,6 ± 11,6	13	t	0,8334	t	**0,0392**	U	0,1191	U	0,5860
Interaction time [s] - object 1 + object 2	2way ANOVA Genotype: p=**0.0100** Treatment: p=0.9258															
Visits [#] - object 1	16,8 ± 6,1	12	18,4 ± 5,7	11	23,9 ± 6,4	15	21,8 ± 9,0	13	t	0,5188	t	**0,0071**	t	0,2755	t	0,4930
Visits [#] - object 2	19,7 ± 3,9	12	20,7 ± 10,6	11	24,4 ± 6,0	15	28,6 ± 8,7	13	t	0,7589	t	**0,0211**	t	0,0630	t	0,1573
Visits [#] - object 1 vs object 2	t p=0.1779		t p=0.5239		t p=0.8166		t p=0.0608									
Visits [#] - object 1 + object 2	36,4 ± 7,4	12	39,1 ± 14,0	11	48,3 ± 11,7	15	50,4 ± 15,2	13	t	0,5802	t	**0,0038**	t	0,0719	t	0,6872
Latency to first visit [s] - object 1	3,9 ± 2,3	12	4,0 ± 3,5	11	4,1 ± 3,6	15	5,3 ± 4,6	13	t	0,9154	U	0,7645	t	0,4460	U	0,6832
Latency to first visit [s] - object 2	3,6 ± 4,2	12	4,9 ± 3,6	11	3,8 ± 2,4	15	5,2 ± 4,1	13	U	0,1950	U	0,3050	U	0,7762	U	0,4883
Latency to first visit [s] - object 1 vs object 2	U p=0.3114		t p=0.5374		U p=0.9755		U p=0.7623									
**Rotarod**																
Latency to fall [s] - day 1	88,8 ± 26,5	12	95,6 ± 53,7	11	84,3 ± 25,7	15	86,2 ± 34,3	13	U	0,8677	t	0,6561	U	0,9219	t	0,8723
Latency to fall [s] - day 2	142,9 ± 32,2	12	134,6 ± 37,8	11	131,2 ± 42,0	15	118,9 ± 41,8	13	t	0,5798	t	0,4198	t	0,3444	t	0,4464
Latency to fall [s] - day 1 vs day 2	t p=**0.0002**		U p=**0.0493**		t p=**0.0012**		t p=**0.0391**									
**Grip Strength**																
Body weight [g]	25,0 ± 1,7	12	25,5 ± 1,5	11	25,2 ± 1,4	15	24,9 ± 2,4	13	t	0,4668	t	0,8477	t	0,4359	t	0,7304
Grip strength [AU] - average	94,1 ± 12,9	12	88,6 ± 14,2	11	95,0 ± 14,0	15	87,3 ± 20,4	13	t	0,3356	t	0,8710	t	0,8636	t	0,2657
Grip strength average/body weight	3,8 ± 0,5	12	3,5 ± 0,5	11	3,8 ± 0,5	15	3,5 ± 0,6	13	t	0,1471	t	0,9802	U	0,6167	U	0,5462
Grip strength [AU] - max force	101,3 ± 14,2	12	95,8 ± 16,8	11	102,1 ± 15,6	15	94,5 ± 20,1	13	t	0,4137	t	0,8789	t	0,8663	t	0,2799
Grip strength max force/body weight	4,1 ± 0,6	12	3,7 ± 0,6	11	4,1 ± 0,5	15	3,8 ± 0,6	13	t	0,2041	t	0,9634	U	0,6382	U	0,4181
**Marble Burying**																
Marbles burried - total [%]	46,5 ± 29,0	12	39,8 ± 27,3	11	13,1 ± 15,6	15	10,9 ± 14,5	13	t	0,5708	U	**0,0008**	U	**0,0042**	U	0,6851
Marbles burried - total [%]	2way ANOVA Genotype: p<**0.0001** Treatment: p=0.4906															
Marbles burried - center [%]	31,3 ± 29,9	12	31,8 ± 29,2	11	7,5 ± 14,8	15	3,8 ± 9,4	13	U	0,8578	U	**0,0085**	U	**0,0006**	U	0,5792
Marbles burried - periphery [%]	54,2 ± 30,0	12	43,8 ± 28,0	11	15,8 ± 17,0	15	14,4 ± 19,2	13	t	0,3981	U	**0,0005**	U	**0,0066**	U	0,6858
**Sucrose Preference**																
Sucrose Preference [%] - day 1	62,1 ± 22,1	12	69,2 ± 19,6	11	70,7 ± 17,4	15	78,3 ± 13,5	13	t	0,2105	U	0,3330	t	0,4277	U	0,2125
Sucrose Preference [%] - day 2	83,0 ± 6,1	12	83,7 ± 5,4	11	80,6 ± 14,4	15	85,4 ± 7,7	13	t	0,5350	U	0,4739	U	0,6833	U	0,9903
Sucrose Preference [%] - day 3	84,6 ± 6,7	12	81,2 ± 11,1	11	79,9 ± 9,6	15	79,2 ± 10,5	13	t	0,6651	t	0,8561	t	0,3946	t	0,1533
Sucrose Preference [%] - over time (1way ANOVA)	p=**0.0004**		p=**0.0359**		p=0.1173		p=0.2033									
Sucrose Preference [%] - over time (3way ANOVA)	Three-way ANOVA Time: p**<0.0001** Treatment: p=0.2274 Genotype: p=0.4323															
**Buried Food Test**																
Latency to first contact [s] - buried day 1	84,6 ± 85,2	12	76,8 ± 49,9	11	112,7 ± 96,1	15	101,8 ± 94,2	13	U	0,7986	U	0,3225	U	0,7441	U	0,5320
Latency to first contact [s] - buried day 2	87,1 ± 84,7	12	81,6 ± 51,5	11	115,2 ± 95,7	15	105,3 ± 94,4	13	U	0,6613	U	0,3164	U	0,8091	U	0,6828
Latency to first contact [s] - visual control	6,3 ± 6,0	12	4,5 ± 2,7	11	6,0 ± 5,3	15	4,0 ± 2,2	13	U	0,8778	U	0,9693	U	0,6791	U	0,6491
**Tail Suspension Test**																
Time of immobility [s] - 0-2min	20,6 ± 8,9	12	22,7 ± 9,1	11	23,3 ± 9,2	15	25,6 ± 11,9	13	t	0,5853	t	0,4519	t	0,5157	t	0,5861
Time of immobility [s] - 2-4min	55,7 ± 8,5	12	58,1 ± 12,6	11	58,6 ± 11,5	15	58,3 ± 14,4	13	t	0,6040	t	0,4590	t	0,9732	t	0,9507
Time of immobility [s] - 4-6min	58,6 ± 10,3	12	56,3 ± 8,8	11	60,2 ± 18,1	15	65,5 ± 15,6	13	t	0,5674	t	0,7821	t	0,0866	t	0,4122
Immobility over time	Three-way ANOVA Time: p**<0.0001** Treatment: p=0.5729 Genotype: p=0.2493															
Time of immobility [s] - 0-6min	135,0 ± 17,5	12	137,2 ± 22,5	11	142,1 ± 33,3	15	149,3 ± 37,2	13	t	0,8008	t	0,4823	t	0,3354	t	0,5948
**Forced Swim Test**																
Time of immobility [s] - 0-2min	9,7 ± 13,7	12	8,0 ± 6,5	11	6,0 ± 5,9	15	3,1 ± 2,3	13	U	0,5653	U	0,6219	t	**0,0330**	U	0,4593
Time of immobility [s] - 2-4min	30,9 ± 26,2	12	39,3 ± 16,8	11	29,7 ± 23,7	15	27,3 ± 20,5	13	t	0,3722	t	0,9005	t	0,1316	t	0,7790
Time of immobility [s] - 4-6min	43,1 ± 31,0	12	59,0 ± 21,2	11	42,3 ± 24,7	15	45,1 ± 30,1	13	t	0,1665	t	0,9439	t	0,2023	t	0,7952
Immobility over time	Three-way ANOVA Time: p**<0.0001** Treatment: p=0.4950 Genotype: p=0.2157															
Time of immobility [s] - 0-6min	83,8 ± 64,7	12	106,2 ± 37,2	11	78,0 ± 49,6	15	75,5 ± 50,4	13	t	0,3164	t	0,8014	t	0,1011	t	0,8974
**Hot Plate**																
Latency to first reaction to heat [s]	12,7 ± 1,9	12	13,0 ± 1,9	11	15,2 ± 2,7	15	16,8 ± 3,8	13	t	0,6931	t	**0,0101**	t	**0,0057**	t	0,2215
Latency to first reaction to heat [s]	2way ANOVA Genotype: p=**0.0002** Treatment: p=0.1902															

**1st Cohort Females**	**Group 1** ZBTB20$^{+/+}$ placebo		**Group 2** ZBTB20$^{+/+}$ rhEPO		**Group 3** ZBTB20$^{+/-}$ placebo		**Group 4** ZBTB20$^{+/-}$ rhEPO		**Comparisons** Group 1vs2		Group 1vs3		Group 2vs4		Group 3vs4	
**Open Field**	mean ± SD	n	mean ± SD	n	mean ± SD	n	mean ± SD	n	test	p-value	test	p-value	test	p-value	test	p-value
Escape latency [s]	16,1 ± 10,4	14	15,6 ± 6,6	13	17,1 ± 9,8	14	18,7 ± 10,4	13	t	0,8758	t	0,7958	t	0,3800	t	0,6950
Track length total [cm]	4227,0 ± 1258,0	14	4503,0 ± 1252,0	13	5646,0 ± 1488,0	14	6059,0 ± 1942,0	14	U	0,8300	U	**0,0186**	U	**0,0145**	U	0,7006
Track length total [cm]	2way ANOVA Genotype: p=**0.0006** Treatment: p=0.3973															
Velocity avg [cm/s]	10,1 ± 3,0	14	10,7 ± 3,0	13	13,5 ± 3,5	14	14,4 ± 4,6	14	U	0,8300	U	**0,0186**	U	**0,0145**	U	0,7006
Zone crossings [#]	639,9 ± 156,4	14	701,5 ± 130,7	13	824,1 ± 180,8	14	868,6 ± 227,9	14	t	0,2763	U	**0,0146**	t	**0,0282**	U	0,7688
Periphery - time within [s]	328,7 ± 51,3	14	347,3 ± 31,1	13	358,9 ± 28,5	14	342,8 ± 34,6	14	U	0,2020	U	0,0690	U	0,5826	U	0,1672
Periphery - track length within [cm]	2725,0 ± 892,9	14	3086,0 ± 694,0	13	4302,0 ± 1492,0	14	4469,0 ± 1534,0	14	t	0,2506	U	**0,0021**	t	**0,0067**	U	0,7006
Periphery - velocity avg within [cm/s]	8,4 ± 2,6	14	9,0 ± 2,7	13	11,9 ± 3,6	14	13,0 ± 4,5	14	U	0,7296	U	**0,0079**	U	**0,0027**	U	0,4898
Periphery - visits to [#]	27,9 ± 11,9	14	28,0 ± 10,5	13	26,1 ± 4,8	14	30,1 ± 11,6	14	U	0,9143	t	0,6099	U	0,6237	t	0,2511
Intermediate - time within [s]	73,3 ± 42,0	14	62,1 ± 22,2	13	52,4 ± 23,8	14	64,9 ± 27,9	14	t	0,3929	U	0,1139	t	0,7761	U	0,2852
Intermediate - track length within [cm]	1271,0 ± 647,2	14	1255,0 ± 522,2	13	1177,0 ± 259,5	14	1377,0 ± 617,1	14	U	0,8674	t	0,6198	U	0,6500	t	0,2775
Intermediate - velocity avg within [cm/s]	18,3 ± 4,2	14	20,7 ± 5,5	13	24,6 ± 6,4	14	22,4 ± 6,6	14	t	0,2261	t	**0,0058**	U	0,3434	U	0,5943
Intermediate - visits to [#]	40,3 ± 18,4	14	34,9 ± 13,8	13	34,6 ± 8,9	14	41,6 ± 17,3	14	U	0,7653	t	0,3148	U	0,2740	t	0,1980
Center - time within [s]	17,8 ± 11,1	14	10,5 ± 10,1	13	8,6 ± 6,1	14	12,2 ± 8,0	14	U	**0,0139**	U	**0,0042**	U	0,2151	U	0,1936
Center - track length within [cm]	227,2 ± 137,1	14	160,6 ± 116,4	13	166,2 ± 64,3	14	211,6 ± 116,7	14	U	0,2388	U	0,3287	U	0,1546	t	0,2163
Center - velocity avg within [cm/s]	13,4 ± 4,4	14	18,3 ± 6,7	13	22,8 ± 7,4	14	20,2 ± 7,1	14	t	**0,0378**	t	**0,0005**	t	0,4715	t	0,3466
Center - visits to [#]	13,0 ± 9,2	14	7,7 ± 4,5	13	8,9 ± 5,2	14	11,6 ± 7,1	14	U	0,1064	U	0,2671	t	0,1023	U	0,3564
**Hole Board**																
Latency to first visit [s]	48,6 ± 75,3	14	36,1 ± 52,5	13	80,6 ± 93,9	14	64,4 ± 59,7	14	U	0,6500	U	0,2141	U	**0,0056**	U	0,6263
Visits [#] - total	14,7 ± 9,0	14	12,5 ± 4,6	13	10,1 ± 5,6	14	13,9 ± 6,7	14	t	0,4353	t	0,1182	t	0,5322	t	0,1122
Visits [#] - most consecutive	1,1 ± 0,9	14	1,1 ± 1,0	13	0,9 ± 0,8	14	1,3 ± 1,1	14	U	0,8809	U	0,4213	t	0,5966	U	0,3246
Visits [#] - total revisits	3,1 ± 3,5	14	2,5 ± 2,7	13	1,9 ± 2,2	14	3,0 ± 6,4	14	U	0,6891	U	0,4255	U	0,8216	U	0,5705
Visits (%) - rigorous revisits	14,9 ± 10,1	14	18,0 ± 14,1	13	7,0 ± 7,3	14	15,8 ± 12,6	14	t	0,5260	U	0,0331	t	0,6805	U	0,0530
Visits (%) - rigorous revisits	2way ANOVA Genotype: p=0.0982 Treatment: p=0.0526															
**Y-Maze**																
Entries [#] - total	18,6 ± 5,8	14	17,9 ± 4,3	13	22,9 ± 8,8	14	22,3 ± 8,6	14	t	0,7163	t	0,1434	t	0,1070	t	0,8466
Entries [#] - alternating	10,2 ± 4,4	14	10,9 ± 2,2	13	13,9 ± 5,5	14	14,0 ± 6,6	14	t	0,5960	t	0,0651	t	0,1178	t	0,9510
Entries [#] - non-alternating	7,5 ± 4,0	14	6,0 ± 3,2	13	8,1 ± 5,4	14	7,3 ± 4,2	14	t	0,2896	t	0,7526	t	0,3728	t	0,6689
Entries [#] - alternating vs non-alternating	t p=0.0990		t p=**0.0001**		t p=**0.0094**		t p=**0.0039**									
Entries [%] - Δ alternating vs non-alternating	16,9 ± 35,1	14	32,5 ± 23,0	13	29,1 ± 28,2	14	31,1 ± 27,3	14	t	0,1830	t	0,3227	t	0,8852	t	0,8490
Centralization Index - 0-100s	1,003 ± 0,0027	130	1,003 ± 0,0016	130	1,004 ± 0,0053	140	1,003 ± 0,0018	140	U	0,1759	U	0,7885	U	0,0504	U	0,3960
Centralization Index - 100-200s	1,003 ± 0,0028	130	1,002 ± 0,0026	130	1,003 ± 0,0049	140	1,001 ± 0,0015	140	U	0,5297	U	0,2674	U	**0,0239**	U	**<0,0001**
Centralization Index - 200-300s	1,001 ± 0,0017	130	1,001 ± 0,0031	130	1,002 ± 0,0031	140	1,001 ± 0,0017	140	U	0,3501	U	**0,0459**	U	0,1341	U	0,0921
Centralization Index - 0-300s	Three-way ANOVA Time: p**<0.0001** Treatment: p=0.3079 Genotype: p=0.6359															
**Social Interaction in Pairs**																
Track length total [m]	30,8 ± 2,7	14	35,1 ± 6,4	12	45,3 ± 3,8	14	45,9 ± 3,0	14	t	**0,0471**	t	**<0.0001**	t	**<0.0001**	t	0,6628
Distance nose-nose [cm] - avg over full time (10s)	Three-way ANOVA Time: p=0.3679 Treatment: p=0.1185 Genotype: p=0.5753															
Distance nose-nose [cm] - total avg	17,26 ± 1,25	7	18,14 ± 1,04	6	17,23 ± 0,94	7	17,68 ± 1,03	7	t	0,1908	t	0,9673	t	0,4407	t	0,4092
Distance nose-nose [cm] - avg over 1. minute (2s)	Three-way ANOVA Time: p=0.4559 Treatment: p=0.0593 Genotype: p=0.8218															
Distance nose-nose [cm] - 1. minute avg	17,4 ± 1,6	7	18,0 ± 1,0	6	17,0 ± 1,1	7	18,6 ± 1,5	7	t	0,4812	t	0,5972	t	0,4111	t	0,0555
Distance nose-nose [cm] - avg over 3 minutes (10s)	Three-way ANOVA Time: p=0.2062 Treatment: p=**0.0363** Genotype: p=0.7064															
Distance nose-nose [cm] - 1. minute avg	16,9 ± 1,5	7	18,4 ± 0,9	6	17,5 ± 0,9	7	18,2 ± 1,6	7	t	0,0591	t	0,4165	t	0,7930	t	0,3135
**Three Chamber Sociability Test**																
Social preference (SP) - overall distance [cm]	3290,0 ± 528,8	14	3595,0 ± 984,2	13	4499,0 ± 750,0	14	4037,0 ± 746,6	14	U	0,5186	t	**<0.0001**	U	**0,0377**	t	0,1147
SP - zone crossings [#]	559,9 ± 89,1	14	586,0 ± 132,2	13	697,8 ± 77,6	14	643,4 ± 108,1	14	U	0,7837	U	**0,0002**	U	**0,0263**	U	0,1035
SP - time in stimulus chamber [s]	81,3 ± 30,3	14	92,2 ± 28,6	13	61,9 ± 20,1	14	49,4 ± 17,7	14	t	0,3479	U	0,0637	t	**0,0002**	U	0,1251
SP - time in empty chamber [s]	65,9 ± 32,4	14	73,3 ± 31,5	13	53,5 ± 15,4	14	43,8 ± 12,1	14	U	0,8300	U	0,3287	U	**0,0003**	U	**0,0310**
SP - time in stimulus vs empty chamber [s]	t p=0.2051		U p=**0.0338**		U p=0.8388		t p=0.3329									
SP - track length in stimulus chamber [cm]	612,1 ± 204,0	14	666,3 ± 205,3	13	696,9 ± 141,1	14	555,9 ± 197,4	14	t	0,4976	t	0,2133	t	0,1675	t	**0,0401**
SP - track length in empty chamber [cm]	548,5 ± 224,3	14	666,0 ± 255,8	13	658,1 ± 189,4	14	546,8 ± 158,0	14	t	0,2181	U	0,1251	U	0,2199	U	**0,0310**
SP - track length in stimulus vs empty chamber [cm]	t p=0.4401		t p=0.9970		U p=0.8743		U p=0.5714									
SP - visits to stimulus chamber [#]	32,6 ± 9,5	14	35,9 ± 10,3	13	45,9 ± 8,2	14	35,1 ± 11,8	14	t	0,4106	t	**0,0006**	t	0,8700	t	**0,0104**
SP - visits to empty chamber [#]	25,4 ± 8,6	14	29,1 ± 9,0	13	37,9 ± 12,0	14	30,9 ± 6,0	14	t	0,2934	t	**0,0042**	t	0,5552	t	0,0633
SP - visits to stimulus vs empty chamber [#]	t p=**0.0460**		t p=0.0868		t p=0.0530		t p=0.2409									
SP - latency to stimulus chamber [s]	43,2 ± 65,4	14	55,6 ± 65,6	13	33,0 ± 39,2	14	60,8 ± 61,5	14	U	0,5186	U	0,8642	U	0,6160	U	0,1898
SP - latency to empty chamber [s]	131,5 ± 111,9	14	109,7 ± 99,0	13	61,3 ± 58,8	14	47,1 ± 60,1	14	U	0,6160	U	0,0767	U	0,0543	U	0,4274
SP - latency to stimulus vs empty chamber [s]	U p=**0.0091**		U p=0.1389		U p=0.1603		U p=0.4013									
SP - time with stimulus insert [s]	220,7 ± 84,5	14	209,9 ± 71,2	13	255,1 ± 51,9	14	265,4 ± 50,5	14	t	0,7205	t	0,2083	t	**0,0301**	t	0,5983
SP - time with empty insert [s]	134,0 ± 38,2	14	128,6 ± 55,2	13	153,7 ± 31,7	14	165,5 ± 39,7	14	t	0,7709	U	0,2100	U	0,0609	U	0,3761
SP - time with stimulus vs empty insert [s]	t p=**0.0026**		t p=**0.0036**		U p**<0.0001**		U p**<0.0001**									
SP - visits to stimulus insert [#]	22,4 ± 6,2	14	24,5 ± 9,0	13	29,8 ± 5,7	14	21,9 ± 6,5	14	t	0,5040	t	**0,0031**	t	0,4128	t	**0,0022**
SP - visits to empty insert [#]	17,4 ± 6,3	14	20,2 ± 7,6	13	23,5 ± 7,3	14	19,0 ± 3,6	14	t	0,3090	t	**0,0240**	t	0,6246	t	0,0512
SP - visits to stimulus vs empty insert [#]	t p=**0.0408**		t p=0.1990		t p=**0.0176**		t p=0.1554									
SP - latency to stimulus insert [s]	52,9 ± 69,5	14	59,2 ± 66,5	13	34,9 ± 39,1	14	63,5 ± 60,8	14	U	0,5502	U	0,9820	U	0,6500	U	0,1936
SP - latency to empty insert [s]	134,8 ± 110,7	14	113,3 ± 99,6	13	62,5 ± 60,1	14	48,4 ± 60,4	14	t	0,5992	U	**0,0395**	U	0,0609	U	0,5112
SP - latency to stimulus vs empty insert [s]	U p=**0.0106**		U p=0.1389		U p=0.3064		U p=0.3287									
SP - time spent in center [s]	98,0 ± 68,1	14	96,0 ± 38,8	13	75,8 ± 17,7	14	75,8 ± 16,9	14	U	0,7564	U	0,3287	U	0,2800	U	0,6027
SP - track length within center [cm]	870,2 ± 444,2	14	1027,0 ± 665,6	13	1163,0 ± 322,3	14	1046,0 ± 297,2	14	U	0,5826	U	**0,0241**	U	0,1546	t	0,3279
SP - visits to center [#]	18,4 ± 7,6	14	20,1 ± 6,8	13	30,9 ± 8,6	14	25,6 ± 9,5	14	U	0,5244	t	**0,0004**	U	0,1798	t	0,1357
Centralization Index - Habituation	Three-way ANOVA Time: p**<0.0001** Treatment: p=0.5670 Genotype: p=0.2959															
Centralization Index - Social Preference	Three-way ANOVA Time: p=0.4840 Treatment: p=0.3048 Genotype: p=**0.0063**															
**Marble Burying**																
Marbles burried - total [%]	23,2 ± 21,5	14	17,6 ± 13,2	13	2,4 ± 4,8	14	5,4 ± 7,4	14	t	0,4217	U	**0,0009**	U	**0,0078**	U	0,3156
Marbles burried - total [%]	2way ANOVA Genotype: p**<0.0001** Treatment: p=0.7368															
Marbles burried - center [%]	16,1 ± 26,6	14	5,8 ± 14,1	13	0,9 ± 3,3	14	1,8 ± 6,7	14	U	0,1000	U	**0,0213**	U	0,3467	U	>0.9999
Marbles burried - periphery [%]	26,8 ± 22,3	14	23,6 ± 16,0	13	3,1 ± 5,9	14	7,1 ± 9,4	14	t	0,6673	U	**0,0008**	U	**0,0047**	U	0,3011
**Nest Building Test**																
Nest Building Score [AU]	2,9 ± 1,0	14	2,7 ± 1,2	13	1,1 ± 0,3	14	1,3 ± 0,5	14	U	0,5818	U	**<0.0001**	U	**0,0009**	U	0,3427
Nest Building Score [AU]	2way ANOVA Genotype: p**<0.0001** Treatment: p=0.9728															
**LABORAS**																
Climbing - duration [s]	7140,0 ± 4710,0	14	7181,0 ± 3161,0	13	14764,0 ± 6335,0	14	11189,0 ± 5769,0	14	t	0,9785	t	**0,0014**	t	**0,0349**	t	0,1307
Climbing - duration [s]	2way ANOVA Genotype: p=**0.0002** Treatment: p=0.1652															
Climbing - occurences [#]	536,1 ± 963,7	14	664,8 ± 985,4	13	1389,0 ± 1845,0	14	2390,0 ± 2945,0	14	U	0,1852	U	**0,0025**	U	**0,0107**	U	0,5112
Climbing - occurences [#]	2way ANOVA Genotype: p=**0.0145** Treatment: p=0.2633															
Locomotion - duration [s]	2402,0 ± 579,1	14	2792,0 ± 719,7	13	3154,0 ± 1622,0	14	3724,0 ± 1645,0	14	t	0,1363	U	0,1936	U	0,1279	U	0,1636
Locomotion - occurences [#]	3994,0 ± 727,7	14	4496,0 ± 1479,0	13	4848,0 ± 2486,0	14	6454,0 ± 3167,0	14	U	0,6160	U	0,5409	U	**0,0291**	U	**0,0350**
Immobility - duration [min]	215,8 ± 66,9	14	203,2 ± 58,9	13	106,3 ± 67,3	14	105,5 ± 61,0	14	t	0,6067	t	**0,0002**	U	**0,0005**	U	0,9820
Immobility - occurences [#]	868,1 ± 319,1	14	874,3 ± 299,5	13	665,9 ± 420,5	14	735,9 ± 257,4	14	U	0,9329	U	**0,0304**	t	0,2116	U	0,1115
Immobility - occurences [#] pooled by genotype	U p=**0.0076**															
Rearing - duration [s]	2060,0 ± 381,5	14	1645,0 ± 473,4	13	1698,0 ± 468,3	14	1765,0 ± 321,2	14	U	**0,0168**	U	**0,0067**	t	0,4508	U	0,2649
Rearing - occurences [#]	1212,0 ± 186,1	14	1163,0 ± 263,1	13	1154,0 ± 244,3	14	1390,0 ± 417,1	14	t	0,5921	t	0,4892	U	0,2388	U	0,1035
Grooming - duration [s]	2532,0 ± 917,9	14	2525,0 ± 954,3	13	1810,0 ± 744,0	14	1733,0 ± 740,9	14	U	0,7564	U	**0,0186**	t	**0,0253**	t	0,7865
Grooming - occurences [#]	578,4 ± 201,2	14	616,5 ± 228,6	13	502,8 ± 220,9	14	483,5 ± 180,5	14	U	0,6160	U	0,2456	t	0,1088	t	0,8023
Drinking - duration [s]	26,9 ± 15,5	14	29,3 ± 23,4	13	6,7 ± 4,4	14	11,8 ± 12,1	14	U	0,9699	U	**<0.0001**	U	0,0925	U	0,4544
Drinking - occurences [#]	14,5 ± 6,5	14	17,2 ± 14,1	13	4,1 ± 3,3	14	7,8 ± 8,0	14	t	0,5424	t	**<0.0001**	U	0,1179	U	0,2973
Eating - duration [s]	1203,0 ± 672,3	14	2143,0 ± 2283,0	13	2671,0 ± 1479,0	14	3494,0 ± 2206,0	14	U	0,2800	t	**0,0033**	U	**0,0377**	t	0,2587
Eating - occurences [#]	408,8 ± 193,1	14	861,3 ± 1544,0	13	1213,0 ± 835,3	14	2049,0 ± 1964,0	14	U	0,5108	U	**0,0001**	U	**0,0033**	U	0,4013
Scratching - duration [s]	538,6 ± 200,7	14	595,6 ± 206,8	13	590,4 ± 245,5	14	582,5 ± 256,5	14	t	0,4749	t	0,5464	t	0,8850	t	0,9342
Scratching - occurences [#]	629,6 ± 199,3	14	655,9 ± 211,0	13	636,2 ± 241,3	14	617,2 ± 213,1	14	t	0,7426	t	0,9380	t	0,6396	t	0,8270
Velocity - maximum [mm/s]	219,1 ± 42,0	14	210,7 ± 25,9	13	238,6 ± 18,3	14	249,8 ± 25,2	14	U	0,6160	t	0,1293	U	**0,0004**	t	0,1896
Velocity - duration of maximum speed [s]	747,3 ± 12,3	14	742,8 ± 22,2	13	746,0 ± 19,0	14	758,9 ± 19,0	14	U	0,3754	t	0,8576	U	0,0541	t	0,0846
Velocity - average speed [mm/s]	3,6 ± 1,0	14	4,2 ± 1,7	13	4,8 ± 1,9	14	6,5 ± 4,6	14	U	0,1852	U	**0,0497**	U	**0,0168**	U	0,1936
Track length [m]	156,5 ± 42,5	14	183,5 ± 73,5	13	208,5 ± 83,4	14	282,5 ± 199,7	14	U	0,1852	U	**0,0497**	U	**0,0168**	U	0,1936
Track length [m]	2way ANOVA Genotype: p=**0.0076** Treatment: p=0.1717															
Circling - events clockwise [#]	510,9 ± 205,4	14	538,7 ± 434,7	13	636,2 ± 701,1	14	1195,0 ± 1042,0	14	U	0,3951	U	0,9358	U	**0,0064**	U	**0,0059**
Circling - events counterclockwise [#]	475,5 ± 162,2	14	550,2 ± 384,5	13	594,7 ± 425,8	14	1106,0 ± 735,8	14	U	0,8569	U	0,6259	U	**0,0174**	U	0,0637

Circling - total events [#]	986,4 ± 356,9	14	1089,0 ± 814,3	13	1231,0 ± 1121,0	14	2300,0 ± 1668,0	14	U	0,6410	U	0,7345	U	**0,0104**	U	**0,0162**
Circling - average duration [s]	3,7 ± 0,2	14	3,7 ± 0,2	13	3,7 ± 0,2	14	3,8 ± 0,2	14	t	0,4312	t	0,3174	t	0,3622	t	0,4572
Circling - average duration [s] pooled by genotype	U p=0.5798															
Circling - average perimeter [mm]	193,5 ± 44,2	14	198,3 ± 79,6	13	226,2 ± 56,9	14	269,6 ± 92,2	14	U	0,6848	U	**0,0162**	U	**0,0091**	U	0,4013
Circling - average velocity [mm/s]	61,6 ± 15,8	14	61,9 ± 25,9	13	72,4 ± 19,9	14	89,0 ± 36,0	14	U	0,5502	U	**0,0310**	U	**0,0145**	U	0,4544
**Vocalization**																
Latency to first call [s]	20,4 ± 46,8	14	24,6 ± 47,4	13	36,4 ± 62,6	14	17,8 ± 47,5	14	U	0,2960	U	0,4070	U	0,0673	U	0,0835
Number of calls [#]	440,4 ± 274,9	14	326,8 ± 282,4	13	443,1 ± 376,4	14	605,9 ± 297,4	14	U	0,3564	U	0,7343	U	**0,0297**	U	0,3578
Duration of calling [s]	14,7 ± 10,0	14	13,4 ± 14,6	13	17,5 ± 16,3	14	26,0 ± 14,8	14	U	0,5272	U	0,6915	U	**0,0492**	U	0,1893
**Prepulse Inhibition (PPI)**																
Hearing - 65 dB [AU]	0,9 ± 1,4	10	0,7 ± 0,3	9	0,9 ± 1,2	12	0,9 ± 1,2	7	U	0,1173	U	**0,0426**	U	0,0851	U	0,1369
Hearing - 120 dB [AU]	2,9 ± 1,0	10	5,2 ± 2,8	9	2,9 ± 2,8	12	3,4 ± 2,2	7	t	**0,0429**	U	0,1802	t	0,1829	U	0,1956
Hearing - 65 dB vs 120 dB	U p=**0.0015**		U p**<0.0001**		U p=**0.0003**		U p=**0.0070**									
PPI - 70dB [%]	41,7 ± 8,8	10	27,9 ± 17,7	9	30,1 ± 11,5	12	39,2 ± 12,7	7	t	0,0562	t	**0,0145**	t	0,1606	t	0,1488
PPI - 75dB [%]	52,4 ± 15,2	10	44,7 ± 19,2	9	44,4 ± 19,6	12	58,6 ± 10,6	7	t	0,3515	t	0,2964	t	0,0891	t	0,0568
PPI - 80dB [%]	60,5 ± 15,1	10	60,1 ± 18,9	9	55,4 ± 12,1	12	61,7 ± 14,2	7	t	0,9592	t	0,4019	U	>0.9999	U	0,3402
PPI one-way/2way ANOVA sound intensity	**0,0147**		**0,0048**		**0,0009**		**0,0073**		2wA	0,2578	2wA	0,0901	2wA	0,2187	2wA	0,0624
**Morris Water Maze**																
Visible 1 - escape latency [s]	20,6 ± 10,1	14	21,9 ± 9,3	13	36,5 ± 17,2	14	49,6 ± 24,5	14	t	0,7269	t	**0,0070**	t	**0,0011**	t	0,1165
Visible 2 - escape latency [s]	9,8 ± 7,2	14	9,2 ± 9,1	13	7,4 ± 5,5	14	12,4 ± 12,3	14	U	0,6160	U	0,2852	U	0,4296	U	0,2456
Visible 1 vs 2 - escape latency [s]	U p=**0.0014**		U p=**0.0003**		U p=**0.0003**		U p**<0.0001**									
Visible 1 - track length [cm]	409,7 ± 189,3	14	466,9 ± 182,6	13	829,5 ± 379,2	14	1082,0 ± 497,3	14	t	0,4316	t	**0,0015**	t	**0,0005**	t	0,1438
Visible 2 - track length [cm]	196,1 ± 96,5	14	199,7 ± 166,5	13	190,7 ± 118,0	14	303,5 ± 274,9	14	U	0,6500	U	0,8949	U	0,3022	U	0,2852
Visible 1 vs 2 - track length [cm]	t p=**0.0013**		U p=**0.0004**		t p**<0.0001**		U p**<0.0001**									
Visible 1 - velocity [cm/s]	21,6 ± 3,3	14	24,0 ± 7,5	13	23,7 ± 3,1	14	23,0 ± 2,7	14	U	0,1852	U	0,0556	U	0,6160	t	0,5151
Visible 2 - velocity [cm/s]	23,2 ± 4,1	14	23,8 ± 4,2	13	27,9 ± 4,3	14	26,9 ± 3,4	14	t	0,7068	t	**0,0067**	t	0,0520	t	0,4913
Visible 1 vs 2 - velocity [cm/s]	U p=**0.0444**		U p=0.5788		t p=**0.0074**		t p=**0.0027**									
Hidden 1 - escape latency [s]	66,4 ± 19,9	14	57,8 ± 19,8	13	65,5 ± 16,4	14	62,9 ± 22,3	14	t	0,2702	t	0,9010	t	0,5372	t	0,7198
Hidden 2 - escape latency [s]	51,5 ± 27,3	14	34,3 ± 19,7	13	58,3 ± 17,4	14	54,6 ± 18,8	14	t	0,0707	t	0,4384	t	**0,0113**	t	0,5915
Hidden 3 - escape latency [s]	41,6 ± 25,6	14	33,5 ± 20,8	13	44,8 ± 17,7	14	46,6 ± 24,9	14	U	0,4798	U	0,5938	U	0,1279	t	0,8340
Hidden 4 - escape latency [s]	34,2 ± 13,3	14	28,7 ± 10,6	13	44,7 ± 19,6	14	38,1 ± 22,3	14	t	0,2471	t	0,1096	U	0,3754	U	0,1936
Hidden 5 - escape latency [s]	32,2 ± 13,2	14	27,1 ± 20,8	13	38,7 ± 22,4	14	35,6 ± 17,6	14	U	0,0945	U	0,7688	U	0,0543	U	0,9820
Hidden 6 - escape latency [s]	29,8 ± 24,8	14	28,9 ± 22,1	13	34,8 ± 15,7	14	34,7 ± 18,8	14	U	0,7305	U	0,2273	U	0,3255	t	0,9816
Hidden 7 - escape latency [s]	32,3 ± 24,7	14	26,3 ± 22,6	13	34,4 ± 19,0	14	32,2 ± 17,7	14	U	0,4583	U	0,6027	U	0,1408	U	0,6347
Hidden 8 - escape latency [s]	27,2 ± 19,4	14	22,7 ± 21,6	13	34,9 ± 23,8	14	28,8 ± 18,7	14	U	0,4879	U	0,3287	U	0,2588	U	0,5409
Hidden 1-8 - escape latency over time	Three-way ANOVA Time: p**<0.0001** Treatment: p=0.1933 Genotype: p=0.0600															
Probe Trial 1 (PT) - time in target quadrant (TQ) [s]	30,4 ± 13,6	14	35,5 ± 13,8	13	23,7 ± 6,4	14	27,3 ± 8,7	14	t	0,3415	t	0,1164	t	0,0820	t	0,2342
Probe Trial 1 (PT) - time in target quadrant (TQ) [s]	2way ANOVA Genotype: p=**0.0153** Treatment: p=0.1504															
PT 1 - visits to TQ [#]	11,9 ± 5,9	14	14,5 ± 5,7	13	9,6 ± 3,2	14	9,6 ± 2,9	14	U	0,1286	t	0,2170	U	**0,0032**	t	0,9512
PT 1 - latency to former escape platform [s]	25,6 ± 23,3	14	16,1 ± 15,1	13	16,8 ± 19,4	14	21,2 ± 21,0	14	U	0,2849	U	0,2062	U	0,6147	U	0,5585
PT 1 - visits to former escape platform [#]	3,3 ± 2,8	14	4,6 ± 3,1	13	2,1 ± 2,2	14	1,8 ± 1,6	14	t	0,2534	U	0,2245	t	**0,0088**	U	0,9005
PT 1 - total track length [cm]	1632,0 ± 517,4	14	1771,0 ± 370,6	13	2075,0 ± 294,7	14	2022,0 ± 226,7	14	U	0,4879	U	**0,0049**	U	**0,0078**	t	0,5991
PT 1 - total track length [cm]	2way ANOVA Genotype: p=**0.0009** Treatment: p=0.6789															
Reversal 1 - escape latency [s]	37,8 ± 27,8	14	36,8 ± 30,0	13	36,4 ± 23,1	14	41,2 ± 26,4	14	U	0,8032	U	>0.9999	U	0,6160	t	0,6084
Reversal 2 - escape latency [s]	30,0 ± 12,5	14	36,9 ± 12,1	13	32,1 ± 20,7	14	30,9 ± 15,7	14	t	0,1554	t	0,7437	t	0,2775	t	0,8664
Reversal 3 - escape latency [s]	29,2 ± 24,9	14	26,5 ± 23,9	13	29,2 ± 16,7	14	32,0 ± 23,7	14	U	0,7564	U	0,6673	U	0,5186	t	0,7223
Reversal 4 - escape latency [s]	21,1 ± 21,0	14	22,1 ± 22,3	13	20,5 ± 13,9	14	29,1 ± 19,6	14	U	0,7203	U	0,6673	U	0,1852	U	0,1499
Reversal 1-4 - escape latency over time // Gen./Treat.	p=0.2790		p=0.2607		p=0.1667		p=0.4637		2wA	0,8712	2wA	0,9947	2wA	0,6842	2wA	0,4701
Reversal 1-4 - escape latency over time	Three-way ANOVA Time: p=**0.0008** Treatment: p=0.5597 Genotype: p=0.7370															
Probe Trial 2 (PT) - time in target quadrant (TQ) [s]	40,1 ± 17,8	14	34,8 ± 12,4	13	26,2 ± 7,1	14	28,9 ± 9,3	14	U	0,6500	U	**0,0067**	t	0,1712	t	0,3947
Probe Trial 2 (PT) - time in target quadrant (TQ) [s]	2way ANOVA Genotype: p=**0.0040** Treatment: p=0.7227															
PT 2 - visits to TQ [#]	14,5 ± 5,5	14	16,5 ± 6,9	13	12,6 ± 3,2	14	12,0 ± 5,6	14	t	0,4079	t	0,2891	t	0,0750	t	0,7141
PT 2 - latency to former escape platform [s]	21,8 ± 21,0	14	11,3 ± 10,2	13	19,2 ± 17,8	14	8,8 ± 11,4	14	t	0,1093	U	0,8387	U	0,3812	U	**0,0191**
PT 2 - visits to former escape platform [#]	3,6 ± 2,5	14	4,8 ± 3,1	13	2,9 ± 1,7	14	2,6 ± 2,5	14	t	0,2784	t	0,3373	t	0,0527	t	0,7899
PT 2 - total track length [cm]	1598,0 ± 485,6	14	1656,0 ± 413,6	13	2152,0 ± 221,6	14	1912,0 ± 407,4	14	U	0,9430	U	**<0.0001**	U	**0,0222**	U	0,3064
PT 2 - total track length [cm]	2way ANOVA Genotype: p=**0.0004** Treatment: p=0.3871															
**Complex Wheel Running**																
Distance [m] - over time	Three-way ANOVA Time: p**<0.0001** Treatment: p=0.8813 Genotype: p=0.4889															
Distance [m] - total	1935,2 ± 317,6	7	1895,1 ± 347,8	6	1801,7 ± 456,2	7	1786,7 ± 546,0	7	t	0,8334	U	>0.9999	t	0,6743	U	0,9015
Time [min] - total	148,7 ± 15,0	7	142,1 ± 23,1	6	141,6 ± 36,0	7	137,5 ± 43,3	7	t	0,5620	U	0,9015	t	0,8117	U	0,8048
Velocity [m/s] - average	0,233 ± 0,035	7	0,241 ± 0,019	6	0,223 ± 0,029	7	0,227 ± 0,031	7	t	0,5845	t	0,5899	t	0,3405	t	0,8063
**Magnet Resonance Tomography**																
Olfactory bulb - volume [mm3]	26,78 ± 0,492	7	27,17 ± 0,617	7	25,9 ± 0,736	7	25,49 ± 0,624	7	t	0,2244	t	**0,0231**	t	**0,0003**	t	0,2934
Olfactory bulb - volume [mm3]	2way ANOVA Genotype: p**<0.0001** Treatment: p=0.9708															
Thalamus - volume [mm3]	17,55 ± 0,605	7	17,73 ± 0,301	7	18,35 ± 0,533	7	17,43 ± 0,801	7	t	0,4930	t	**0,0216**	t	0,3828	t	**0,0286**
Thalamus - volume [mm3]	2way ANOVA Genotype: p=0.3097 Treatment: p=0.1421															
Hippocampus - volume [mm3]	66,48 ± 1,088	7	65,54 ± 2,124	7	69,34 ± 1,779	7	66,97 ± 2,34	7	t	0,3245	t	**0,0047**	t	0,2534	t	0,0561
Hippocampus - volume [mm3]	2way ANOVA Genotype: p=**0.0060** Treatment: p=**0.0293**															
Corpus Callosum - volume [mm3]	1,135 ± 0,027	7	1,158 ± 0,052	7	1,149 ± 0,050	7	1,137 ± 0,031	7	t	0,3203	t	0,5252	t	0,3670	t	0,5855
Corpus Callosum - volume [mm3]	2way ANOVA Genotype: p=0.8187 Treatment: p=0.7373															
Ventricles - volume [mm3]	11,43 ± 0,989	7	10,41 ± 0,889	7	11,66 ± 0,978	7	10,16 ± 0,532	7	U	**0,0262**	t	0,6753	U	0,9015	t	**0,0058**
Ventricles - volume [mm3]	2way ANOVA Genotype: p=0.9689 Treatment: p=**0.0007**															
Cerebellum - volume [mm3]	55,46 ± 0,544	7	54,7 ± 1,496	7	57,68 ± 1,55	7	55,72 ± 1,615	7	t	0,2472	t	**0,0080**	t	0,2443	t	**0,0391**
Cerebellum - volume [mm3]	2way ANOVA Genotype: p=**0.0047** Treatment: p=**0.0156**															
Brain matter - volume [mm3]	492 ± 9,738	7	496,8 ± 13,96	7	506,7 ± 15,93	7	492,8 ± 12	7	U	0,3176	t	0,0640	U	0,5350	t	0,0909
Brain matter - volume [mm3]	2way ANOVA Genotype: p=0.3137 Treatment: p=0.3858															
Brain - volume [mm3]	503,4 ± 10,01	7	507,2 ± 14,45	7	518,4 ± 16,54	7	502,9 ± 12,24	7	t	0,5851	t	0,0686	t	0,5633	t	0,0723
Brain - volume [mm3]	2way ANOVA Genotype: p=0.3295 Treatment: p=0.2857															
**Organ Collection**																
Body weight [g]	24,00 ± 1,16	7	23,43 ± 1,13	7	23,29 ± 1,89	7	22,57 ± 0,98	7	t	0,3686	t	0,4136	t	0,1560	t	0,3974
Tibia length [mm]	18,20 ± 0,30	7	18,26 ± 0,26	7	18,92 ± 0,32	7	18,53 ± 0,60	7	t	0,7074	t	**0,0009**	t	0,3041	t	0,1609
Brain - weight [g]	0,4498 ± 0,0250	7	0,4517 ± 0,0175	7	0,4539 ± 0,0158	7	0,4494 ± 0,0188	7	t	0,8779	U	0,9015	t	0,8232	U	0,5350
Brain - weight [g] pooled by genotype	t p=0.8991															
Brain - dry weight [g]	0,0994 ± 0,0059	7	0,0995 ± 0,0041	7	0,0993 ± 0,0045	7	0,0966 ± 0,0041	7	t	0,9796	t	0,9723	t	0,2230	t	0,2714
Brain - dry weight [g] pooled by genotype	U p=0.2599															
Brain - water content [g]	0,3505 ± 0,0191	7	0,3522 ± 0,0138	7	0,3546 ± 0,0119	7	0,3528 ± 0,0151	7	t	0,8486	U	>0.9999	t	0,9380	U	0,5350
Brain - water content [g] pooled by genotype	t p=0.6712															
Brain - weight [g]/Body weight [g]	0,0188 ± 0,0013	7	0,0193 ± 0,0004	7	0,0196 ± 0,0015	7	0,0199 ± 0,0008	7	t	0,3491	t	0,3041	t	0,1047	t	0,6151
Brain - weight [g]/Tibia length [mm]	0,0247 ± 0,0013	7	0,0247 ± 0,0007	7	0,0240 ± 0,0009	7	0,0243 ± 0,0008	7	t	0,9720	t	0,2452	t	0,2689	t	0,5629
Heart - weight [g]	0,1031 ± 0,0132	7	0,1147 ± 0,0149	7	0,1072 ± 0,0155	7	0,1046 ± 0,0079	7	t	0,1501	t	0,6118	t	0,1482	t	0,7115
Heart - dry weight [g]	0,0233 ± 0,0027	7	0,0246 ± 0,0018	7	0,0235 ± 0,0029	7	0,0222 ± 0,0014	7	t	0,3122	t	0,9254	t	**0,0167**	t	0,3210
Heart - water content [g]	0,0798 ± 0,0106	7	0,0901 ± 0,0135	7	0,0837 ± 0,0127	7	0,0824 ± 0,0066	7	t	0,1397	t	0,5477	t	0,2111	t	0,8253
Heart - weight [g]/Body weight [g]	0,0043 ± 0,0005	7	0,0049 ± 0,0006	7	0,0046 ± 0,0004	7	0,0046 ± 0,0002	7	U	0,0973	U	0,0973	t	0,3151	t	0,8141
Heart - weight [g]/Tibia length [mm]	0,0057 ± 0,0007	7	0,0063 ± 0,0008	7	0,0057 ± 0,0008	7	0,0056 ± 0,0004	7	t	0,1342	t	0,9888	t	0,0834	t	0,9758
Lungs - weight [g]	0,1471 ± 0,0103	7	0,1531 ± 0,0144	7	0,1690 ± 0,0266	7	0,1482 ± 0,0076	7	t	0,3913	t	0,0777	U	0,3176	U	**0,0379**
Lungs - dry weight [g]	0,0359 ± 0,0035	7	0,0357 ± 0,0038	7	0,0358 ± 0,0044	7	0,0291 ± 0,0068	7	t	0,9312	t	0,9529	U	**0,0175**	U	0,0530
Lungs - water content [g]	0,1112 ± 0,0081	7	0,1174 ± 0,0110	7	0,1333 ± 0,0232	7	0,1191 ± 0,0090	7	t	0,2576	t	**0,0473**	t	0,7496	t	0,1726
Lungs - weight [g]/Body weight [g]	0,0061 ± 0,0006	7	0,0065 ± 0,0005	7	0,0073 ± 0,0009	7	0,0066 ± 0,0004	7	t	0,2382	t	**0,0217**	t	0,8795	t	0,1050
Lungs - weight [g]/Tibia length [mm]	0,0081 ± 0,0006	7	0,0084 ± 0,0007	7	0,0089 ± 0,0013	7	0,0080 ± 0,0005	7	t	0,4357	t	0,1700	U	0,1649	U	0,1282

2nd Cohort Females

**Neophobia**																
Track length - total [cm]	2334,0 ± 324,4	16	2281,0 ± 304,6	15	2550,0 ± 321,5	8	2680,0 ± 442,1	11	t	0,6417	t	0,1433	t	**0,0196**	t	0,4678
Zone crossings - total [cm]	275,2 ± 36,8	16	265,3 ± 36,3	15	308,5 ± 21,4	8	309,1 ± 48,8	11	t	0,4561	t	**0,0107**	U	**0,0373**	U	0,7331
Interaction time [s] - object 1	36,5 ± 14,0	16	30,0 ± 12,4	15	37,5 ± 7,7	8	34,1 ± 10,7	11	t	0,1866	t	0,8142	U	0,5400	U	0,2723
Interaction time [s] - object 2	28,4 ± 7,9	16	33,5 ± 13,0	15	30,1 ± 7,7	8	32,0 ± 9,4	11	t	0,2027	t	0,6182	U	0,7988	U	0,6574
Interaction time [s] - object 1 vs object 2	t p=0.0554		t p=0.4666		t p=0.0734		U p=0.7477									
Interaction time [s] - object 1 + object 2	64,9 ± 20,4	16	63,5 ± 21,6	15	67,6 ± 13,3	8	66,2 ± 13,3	11	t	0,8612	t	0,6935	t	0,7035	t	0,8171
Visits [#] - object 1	34,7 ± 12,4	16	31,5 ± 11,1	15	44,1 ± 11,1	8	38,2 ± 13,4	11	t	0,4606	t	0,0778	t	0,1942	t	0,3057
Visits [#] - object 2	20,8 ± 5,0	16	23,5 ± 7,4	15	24,5 ± 7,0	8	26,2 ± 4,7	11	t	0,2434	t	0,2104	U	0,2012	U	0,6997
Visits [#] - object 1 vs object 2	t p=**0.0005**		t p=**0.0285**		t p=**0.0012**		U p=**0.0048**									
Visits [#] - object 1 + object 2	55,5 ± 15,6	16	55,1 ± 14,4	15	68,6 ± 16,4	8	64,4 ± 13,6	11	t	0,9366	t	0,0825	t	0,1076	t	0,5591
Latency to first visit [s] - object 1	4,1 ± 3,1	16	4,9 ± 4,0	15	8,1 ± 5,3	8	6,9 ± 5,2	11	U	0,7194	t	0,0796	U	0,4130	t	0,6366
Latency to first visit [s] - object 2	6,3 ± 2,8	16	4,6 ± 3,8	15	3,9 ± 2,7	8	6,1 ± 2,2	11	t	0,1784	t	0,0692	t	0,2174	t	0,0854
Latency to first visit [s] - object 1 vs object 2	t p=**0.0498**		U p=0.9349		t p=0.0738		t p=0.6235									
**Rotarod**																
Latency to fall [s] - day 1	125,7 ± 48,4	16	157,3 ± 78,1	15	140,4 ± 40,4	8	145,1 ± 41,0	11	t	0,1915	t	0,4435	t	0,6099	t	0,8061
Latency to fall [s] - day 2	159,4 ± 58,4	16	186,7 ± 55,6	15	125,6 ± 51,2	8	160,0 ± 40,5	11	t	0,1940	t	0,1654	t	0,1699	t	0,1395
Latency to fall [s] - day 1 vs day 2	t p=0.0855		t p=0.2472		t p=0.5332		t p=0.4007									
**Grip Strength**																
Body weight [g]	19,4 ± 1,0	16	19,9 ± 1,0	15	19,0 ± 0,9	8	18,5 ± 0,9	11	t	0,1433	t	0,3153	t	**0,0014**	t	0,2902
Grip strength [AU] - average	74,6 ± 16,5	16	67,8 ± 12,2	15	68,7 ± 10,7	8	65,0 ± 7,7	11	t	0,2065	t	0,3053	t	0,4764	t	0,4274
Grip strength average/body weight	3,8 ± 0,8	16	3,4 ± 0,6	15	3,6 ± 0,6	8	3,5 ± 0,4	11	t	0,0901	t	0,4587	t	0,5934	t	0,6892
Grip strength [AU] - max force	83,1 ± 15,7	16	79,7 ± 17,0	15	78,6 ± 13,9	8	75,1 ± 7,9	11	t	0,5616	t	0,4842	t	0,3712	t	0,5318
Grip strength max force/body weight	4,3 ± 0,7	16	4,0 ± 0,8	15	4,1 ± 0,8	8	4,0 ± 0,4	11	t	0,2841	t	0,6610	t	0,8063	t	0,7680
**Marble Burying**																
Marbles burried - total [%]	17,2 ± 18,6	16	23,3 ± 16,1	15	7,8 ± 8,8	8	6,8 ± 8,2	11	U	0,1953	U	0,1819	U	**0,0033**	U	0,5785
Marbles burried - center [%]	14,1 ± 22,8	16	10,0 ± 13,5	15	7,8 ± 9,3	8	2,3 ± 5,1	11	U	0,8565	U	0,8847	U	0,1354	U	0,2489
Marbles burried - periphery [%]	18,8 ± 17,8	16	30,0 ± 20,8	15	7,8 ± 9,9	8	9,1 ± 11,3	11	t	0,1178	U	0,1441	U	0,0035	U	0,9422
Marbles burried - periphery [%]	2way ANOVA Genotype: p=**0.0034** Treatment: p=0.4103															
**Sucrose Preference**																
Sucrose Preference [%] - day 1	56,8 ± 19,8	16	55,3 ± 14,4	15	48,8 ± 18,1	8	48,7 ± 16,1	11	U	0,5196	U	0,2636	t	0,2905	t	0,9885
Sucrose Preference [%] - day 2	82,0 ± 7,6	16	81,0 ± 8,5	15	81,3 ± 9,8	8	80,4 ± 5,5	11	t	0,7194	U	0,7523	t	0,8264	U	0,2375
Sucrose Preference [%] - day 3	87,9 ± 5,7	16	87,0 ± 4,3	15	86,2 ± 4,4	8	87,4 ± 4,4	11	t	0,6486	t	0,4482	t	0,8328	t	0,5729
Sucrose Preference [%] - over time (one-way ANOVA)	p**<0.0001**		p**<0.0001**		p=**0.0028**		p**<0.0001**									
**Buried Food Test**																
Latency to first contact [s] - buried day 1	54,1 ± 69,6	16	73,1 ± 94,8	15	115,8 ± 104,3	8	93,5 ± 83,4	11	U	0,6752	U	0,1021	U	0,2869	U	0,7168
Latency to first contact [s] - buried day 2	65,1 ± 70,3	15	76,2 ± 85,5	13	123,9 ± 113,7	7	133,6 ± 98,9	11	U	0,9729	U	0,2898	U	**0,0287**	U	0,5204
Latency to first contact [s] - visual control	4,8 ± 1,9	16	3,6 ± 1,9	15	6,5 ± 2,1	8	5,5 ± 3,2	11	U	0,0722	t	0,0718	U	0,0958	t	0,4032
**Tail Suspension Test**																
Time of immobility [s] - 0-2min	14,7 ± 8,8	16	22,5 ± 11,5	15	16,1 ± 9,4	8	17,1 ± 9,8	10	U	0,0801	U	0,6206	t	0,2274	t	0,8320
Time of immobility [s] - 2-4min	51,1 ± 18,5	16	65,9 ± 16,3	15	55,0 ± 18,4	8	55,9 ± 14,8	10	U	**0,0221**	t	0,6333	U	0,0995	t	0,9109
Time of immobility [s] - 4-6min	55,7 ± 25,9	16	63,8 ± 14,6	15	66,5 ± 17,7	8	60,5 ± 22,8	10	t	0,2910	t	0,2441	t	0,6932	t	0,5387
Immobility over time	Three-way ANOVA Time: p**<0.0001** Treatment: p=0.2304 Genotype: p=0.9098															
Time of immobility [s] - 0-6min	121,5 ± 38,3	16	152,1 ± 31,2	15	137,6 ± 41,2	8	133,5 ± 38,1	10	U	**0,0240**	U	0,3265	t	0,2165	U	0,7618
**Forced Swim Test**																
Time of immobility [s] - 0-2min	6,0 ± 10,7	16	3,8 ± 3,9	15	1,8 ± 2,3	8	4,0 ± 4,4	11	U	0,5893	U	0,3127	U	0,8278	U	0,2965
Time of immobility [s] - 2-4min	31,9 ± 25,6	16	25,4 ± 15,9	15	15,4 ± 15,2	8	11,0 ± 15,0	11	U	0,8386	U	0,1358	U	**0,0087**	U	0,3459
Time of immobility [s] - 4-6min	43,7 ± 28,8	16	51,5 ± 27,1	15	26,9 ± 24,6	8	19,5 ± 24,8	11	t	0,4408	t	0,1564	U	**0,0056**	U	0,5843
Immobility over time	Three-way ANOVA Time: p**<0.0001** Treatment: p=0.7074 Genotype: p=**0.0037**															
Time of immobility [s] - 0-6min	81,5 ± 58,4	16	80,8 ± 40,4	15	44,1 ± 39,3	8	34,5 ± 39,2	11	t	0,9666	t	0,0781	U	**0,0088**	U	0,5302
**Hot Plate**																
Latency to first reaction to heat [s]	12,9 ± 1,7	16	12,6 ± 1,8	15	18,2 ± 4,4	8	13,5 ± 1,5	11	t	0,6318	t	**0,0105**	t	0,1812	t	**0,0185**
Latency to first reaction to heat [s]	2way ANOVA Genotype: p=**0.0003** Treatment: p=**0.0093**															

P value	avg_log2FC	Percent of cells (corresponding cell type)	Percent of cells (rest of the cell types)	Adjusted p value	Celltype	Genes
0	3,619508138	0,993	0,21	0	OPC1	Lhfpl3
0	2,30306798	0,947	0,141	0	OPC1	Nxph1
0	2,1644994	0,95	0,176	0	OPC1	Sox6
0	2,124896774	0,843	0,022	0	OPC1	Pdgfra
0	2,120186263	0,981	0,313	0	OPC1	Ptprz1
0	2,075370643	0,97	0,312	0	OPC1	Sox2ot
0	2,034324342	0,979	0,584	0	OPC1	Xylt1
0	2,034125695	0,998	0,854	0	OPC1	Tnr
0	2,005397581	0,994	0,567	0	OPC1	Pcdh15
0	1,888371554	0,806	0,061	0	OPC1	Vcan
0	1,868812382	0,965	0,565	0	OPC1	Epn2
0	1,852406295	0,998	0,887	0	OPC1	Dscam
0	1,755164675	1	0,904	0	OPC1	Lrrc4c
0	1,661358377	0,768	0,093	0	OPC1	Gm38505
0	1,628564082	0,772	0,112	0	OPC1	Gm4876
0	1,619487222	0,815	0,135	0	OPC1	Arhgap31
0	1,565790301	0,963	0,438	0	OPC1	Brinp3
0	1,546771828	0,958	0,632	0	OPC1	Zeb1
0	1,535423697	0,998	0,77	0	OPC1	Grid2
0	1,491439718	0,702	0,04	0	OPC1	Cacng4
0	1,473644057	0,987	0,543	0	OPC1	Gpc5
0	1,352864596	0,969	0,537	0	OPC1	Luzp2
0	1,30914908	0,922	0,513	0	OPC1	Slc35f1
0	1,306752551	0,678	0,11	0	OPC1	Megf11
0	1,272894814	0,818	0,26	0	OPC1	Cspg5
0	1,272795787	0,768	0,278	0	OPC1	Pcdh11x
0	1,266495117	0,811	0,399	0	OPC1	Mir9-3hg
0	1,215964109	0,669	0,109	0	OPC1	Ppfibp1
0	1,184312	0,864	0,388	0	OPC1	Nckap5
0	1,182732397	0,971	0,436	0	OPC1	Npas3
0	1,141663139	0,82	0,405	0	OPC1	Fchsd2
0	1,136029141	0,949	0,489	0	OPC1	Ptprt
0	1,109060211	0,959	0,652	0	OPC1	Sgcd
0	1,104303783	0,731	0,319	0	OPC1	Chst11
0	1,081968192	0,811	0,471	0	OPC1	Usp24
0	1,077151747	0,951	0,543	0	OPC1	Maml2
0	1,067113556	0,76	0,413	0	OPC1	Kcnip3
0	1,061406098	0,674	0,202	0	OPC1	Grin3a
0	1,04893399	0,525	0,028	0	OPC1	Stk32a
0	1,044231273	0,58	0,086	0	OPC1	Abtb2
0	1,035659326	0,828	0,443	0	OPC1	Sh3d19
0	1,026696461	0,651	0,115	0	OPC1	Sox10

0	1,025818687	0,974	0,367	0	OPC1	Erbb4
0	1,022116357	0,914	0,728	0	OPC1	Rev3l
0	1,0216958	0,903	0,557	0	OPC1	Sox5
0	1,017092198	0,842	0,485	0	OPC1	Dpyd
0	1,01181915	0,52	0,035	0	OPC1	Kcnh8
0	1,010780099	0,644	0,139	0	OPC1	Itpr2
0	1,010042964	0,743	0,384	0	OPC1	Zfp462
0	1,007246525	0,82	0,484	0	OPC1	Pid1
0	1,002371001	0,522	0,03	0	OPC1	Emid1
0	0,999923395	0,561	0,113	0	OPC1	Unc13c
0	0,989150316	0,575	0,115	0	OPC1	Myt1
0	0,978663047	0,585	0,142	0	OPC1	Adam12
0	0,969534942	0,508	0,041	0	OPC1	Sema3d
0	0,967685388	0,968	0,729	0	OPC1	Sema6d
0	0,966326531	0,998	0,973	0	OPC1	Csmd3
0	0,963547872	0,637	0,149	0	OPC1	Bcas1
0	0,948230549	0,811	0,349	0	OPC1	Sorcs1
0	0,933244687	0,71	0,405	0	OPC1	Gm32509
0	0,897027931	0,55	0,089	0	OPC1	Kank1
0	0,895697254	0,617	0,238	0	OPC1	Nova1
0	0,890605344	0,592	0,16	0	OPC1	Mtss2
0	0,884095778	0,663	0,289	0	OPC1	Srgap1
0	0,87802334	0,802	0,582	0	OPC1	Cask
0	0,873053456	0,581	0,142	0	OPC1	Wipf1
0	0,866569402	0,57	0,135	0	OPC1	Serpine2
0	0,856341054	0,98	0,921	0	OPC1	Cntn1
0	0,85101193	0,979	0,554	0	OPC1	Qk
0	0,850174378	0,701	0,304	0	OPC1	Tmem132d
0	0,849510917	0,574	0,236	0	OPC1	Plppr1
0	0,845225856	0,894	0,605	0	OPC1	Gm3764
0	0,832195439	0,718	0,413	0	OPC1	Nrxn2
0	0,826425646	0,615	0,193	0	OPC1	Bcan
0	0,818366459	0,999	0,963	0	OPC1	Il1rapl1
0	0,808541596	0,936	0,657	0	OPC1	Astn2
0	0,788374083	0,995	0,913	0	OPC1	Rora
0	0,787510552	0,88	0,653	0	OPC1	Klf12
0	0,782204466	0,774	0,336	0	OPC1	Cdh13
0	0,770947987	0,698	0,339	0	OPC1	Pde7b
0	0,769878067	0,998	0,988	0	OPC1	Anks1b
0	0,767680625	0,836	0,405	0	OPC1	Gpc6
0	0,764080964	0,668	0,355	0	OPC1	Spon1
0	0,763760487	0,784	0,526	0	OPC1	Ncald
0	0,754457016	0,988	0,912	0	OPC1	01. Mrz

0	0,754211036	0,814	0,626	0	OPC1	Nav1
0	0,743205463	0,609	0,285	0	OPC1	Hip1
0	0,736703725	0,844	0,7	0	OPC1	Ankrd11
0	0,730726456	0,625	0,229	0	OPC1	Dock1
0	0,729459945	0,999	0,988	0	OPC1	Ctnna2
0	0,718686777	0,573	0,193	0	OPC1	Gm20713
0	0,718503445	0,928	0,506	0	OPC1	Gm42418
0	0,7162523	0,999	0,989	0	OPC1	Mdga2
0	0,710357055	0,713	0,346	0	OPC1	Cdh20
0	0,708221466	0,994	0,831	0	OPC1	Ncam2
0	0,70035168	0,568	0,212	0	OPC1	Chd7
0	0,697511598	1	0,986	0	OPC1	Nlgn1
0	0,696682652	0,953	0,716	0	OPC1	Pcdh7
0	0,695739312	0,992	0,978	0	OPC1	Nav3
0	0,691720836	0,827	0,624	0	OPC1	Agap1
0	0,684457132	0,922	0,663	0	OPC1	Dnm3
0	0,682998322	1	0,991	0	OPC1	Nrxn1
0	0,673450681	0,982	0,955	0	OPC1	Sntg1
0	0,673184261	0,538	0,141	0	OPC1	Gjc3
0	0,664924398	0,925	0,793	0	OPC1	Csmd2
0	0,655548134	1	0,965	0	OPC1	Lrp1b
0	0,655044289	0,953	0,857	0	OPC1	Mmp16
0	0,654405555	1	0,988	0	OPC1	Lsamp
0	0,64438089	0,965	0,885	0	OPC1	Grid1
0	0,643522937	0,523	0,269	0	OPC1	Snx25
0	0,640991607	0,933	0,459	0	OPC1	Adarb2
0	0,63504977	0,513	0,265	0	OPC1	Abhd2
0	0,626598044	0,967	0,843	0	OPC1	Dscaml1
0	0,618232357	0,99	0,892	0	OPC1	Dcc
0	0,612712024	0,995	0,962	0	OPC1	Adgrl3
0	0,612205162	0,5	0,25	0	OPC1	Evi5l
0	0,606858519	0,649	0,435	0	OPC1	2610035D17Rik
0	0,593170755	0,729	0,561	0	OPC1	Phactr3
0	0,588059274	0,989	0,949	0	OPC1	Nav2
0	0,587883927	0,992	0,806	0	OPC1	Ntm
0	0,587283508	0,536	0,252	0	OPC1	Cobl
0	0,583829657	0,996	0,973	0	OPC1	Kcnd2
0	0,583704541	0,783	0,547	0	OPC1	Lrrtm3
0	0,580935406	0,824	0,616	0	OPC1	Alcam
0	0,569635962	0,52	0,201	0	OPC1	Cntn6
0	0,565069145	0,913	0,639	0	OPC1	Cntn4
0	0,557224362	0,584	0,324	0	OPC1	Kif13a
0	0,549902535	0,623	0,292	0	OPC1	Tox

0	0,53782072	0,951	0,856	0	OPC1	Ptprg
0	0,535513255	0,525	0,249	0	OPC1	Sdk1
0	0,526646808	0,61	0,32	0	OPC1	Sema5a
0	0,526523617	0,898	0,779	0	OPC1	Hnrnpa2b1
0	0,510657665	0,534	0,214	0	OPC1	Phldb1
0	0,505174485	0,99	0,911	0	OPC1	Ncam1
9,92E-303	0,59687226	0,523	0,305	2,67E-298	OPC1	Tpm1
3,61E-293	0,545152045	0,647	0,393	9,73E-289	OPC1	Inpp4b
2,17E-270	0,538243619	0,949	0,831	5,83E-266	OPC1	Ppp2r2b
2,47E-252	0,534418271	0,707	0,559	6,66E-248	OPC1	Ankrd28
2,48E-243	0,528817734	0,756	0,622	6,68E-239	OPC1	Galnt13
8,70E-216	0,508471091	0,506	0,326	2,34E-211	OPC1	Fip1l1
4,17E-200	0,536893693	0,534	0,374	1,12E-195	OPC1	Scfd2
7,44E-193	0,512569828	0,642	0,509	2,00E-188	OPC1	Nmnat2

0	2,658683144	0,562	0,162	0	OPC2	Bcas1
0	2,324716	0,971	0,858	0	OPC2	Tnr
0	2,222066014	0,833	0,536	0	OPC2	Fyn
0	1,967645691	0,681	0,124	0	OPC2	Tcf7l2
0	1,962315688	0,599	0,141	0	OPC2	Tns3
0	1,704365535	0,779	0,197	0	OPC2	Sox6
0	1,596107358	0,548	0,125	0	OPC2	Ppfibp1
0	1,586820411	0,664	0,151	0	OPC2	Itpr2
0	1,580237597	0,949	0,706	0	OPC2	Tmem108
0	1,554563386	0,575	0,165	0	OPC2	Epb41l2
0	1,455307887	0,834	0,399	0	OPC2	Nckap5
0	1,419774304	0,858	0,323	0	OPC2	Sema5a
0	1,367841327	0,979	0,564	0	OPC2	Qk
0	1,366454872	0,61	0,216	0	OPC2	Sirt2
0	1,338744582	0,608	0,182	0	OPC2	Mpzl1
0	1,296882949	0,504	0,099	0	OPC2	Abtb2
0	1,153223771	0,938	0,553	0	OPC2	Maml2
0	1,016971415	0,801	0,334	0	OPC2	St18
0	0,995616126	0,733	0,322	0	OPC2	Csgalnact1
0	0,993534061	0,632	0,242	0	OPC2	Ptpro
0	0,844688884	0,558	0,1	0	OPC2	Kank1
0	0,814617757	0,67	0,202	0	OPC2	Bcan
0	0,580925873	0,562	0,161	0	OPC2	Plpp3
4,96E-300	0,75583366	0,782	0,332	1,34E-295	OPC2	Ptprz1
4,78E-280	1,570937978	0,791	0,454	1,29E-275	OPC2	Ust
2,32E-273	1,280434368	0,913	0,776	6,24E-269	OPC2	Ptprj
3,56E-270	0,738633808	0,717	0,304	9,58E-266	OPC2	Stard13
2,70E-243	0,721685577	0,59	0,206	7,27E-239	OPC2	Plp1

2,81E-239	0,623881401	0,561	0,212	7,56E-235	OPC2	Cald1
9,59E-235	1,06761718	0,74	0,355	2,58E-230	OPC2	Cdh20
2,89E-228	1,150415831	0,735	0,418	7,79E-224	OPC2	Chd3
1,61E-224	0,554585933	0,591	0,231	4,33E-220	OPC2	Acsl3
4,48E-220	0,836530138	0,652	0,316	1,21E-215	OPC2	Phyhipl
8,14E-220	0,744871787	0,793	0,455	2,19E-215	OPC2	Arhgap5
1,30E-215	0,583597479	0,863	0,575	3,51E-211	OPC2	Msi2
6,05E-215	0,885213445	0,984	0,835	1,63E-210	OPC2	Ncam2
2,65E-210	0,852557054	0,841	0,549	7,13E-206	OPC2	Luzp2
1,09E-208	2,039413031	0,99	0,979	2,94E-204	OPC2	Frmd4a
9,52E-207	0,932674041	0,651	0,359	2,56E-202	OPC2	Rapgef1
3,26E-196	0,911946854	0,735	0,435	8,78E-192	OPC2	Lcorl
1,24E-194	0,913097755	0,667	0,374	3,33E-190	OPC2	Arhgap35
1,54E-194	0,927950662	0,977	0,911	4,14E-190	OPC2	Cdk14
1,56E-189	0,865037657	0,965	0,89	4,19E-185	OPC2	Dscam
9,44E-188	0,753632211	0,962	0,889	2,54E-183	OPC2	Map1b
4,38E-183	0,737306622	0,768	0,462	1,18E-178	OPC2	Gm35188
1,89E-170	0,676087576	0,737	0,43	5,09E-166	OPC2	Pcdh17
5,39E-169	1,212112893	0,651	0,334	1,45E-164	OPC2	Mbp
8,12E-168	0,518217824	0,529	0,221	2,19E-163	OPC2	Chd7
2,41E-165	0,980082532	0,669	0,405	6,49E-161	OPC2	Slc22a23
7,92E-161	0,572106715	0,505	0,228	2,13E-156	OPC2	Klhl5
2,51E-158	1,041646637	0,67	0,422	6,77E-154	OPC2	Pdcd4
3,88E-156	1,663792875	0,507	0,263	1,05E-151	OPC2	Pik3r3
9,82E-156	0,584005496	0,653	0,36	2,65E-151	OPC2	Ptpre
3,10E-152	0,607333944	0,52	0,229	8,36E-148	OPC2	Slc1a3
3,38E-148	0,836717639	0,696	0,439	9,10E-144	OPC2	2610035D17Rik
1,12E-143	0,761595314	0,684	0,436	3,01E-139	OPC2	Rictor
3,09E-143	0,655988888	1	0,991	8,32E-139	OPC2	Cadm2
1,34E-142	0,541837829	0,716	0,402	3,61E-138	OPC2	Thsd7a
8,42E-140	0,645278162	0,653	0,349	2,27E-135	OPC2	Cdh13
2,09E-139	0,78861405	0,698	0,452	5,62E-135	OPC2	Lrch3
3,97E-138	0,794500829	0,792	0,622	1,07E-133	OPC2	Slc44a1
4,25E-138	0,764442516	0,681	0,431	1,14E-133	OPC2	Slc1a1
7,32E-137	0,894498872	0,552	0,305	1,97E-132	OPC2	Sema4d
8,41E-137	0,518956413	0,545	0,249	2,27E-132	OPC2	Plekhg1
4,84E-134	0,765169439	0,766	0,529	1,30E-129	OPC2	Map4k4
1,19E-127	0,714984181	0,992	0,935	3,21E-123	OPC2	Dock4
4,48E-124	0,75217633	0,544	0,301	1,21E-119	OPC2	Etv6
2,35E-122	0,764181541	0,688	0,454	6,32E-118	OPC2	Sh3d19
1,20E-121	0,674064002	0,944	0,776	3,22E-117	OPC2	Grid2
1,49E-117	0,537276743	0,945	0,887	4,01E-113	OPC2	Grid1
4,10E-117	0,704526198	0,976	0,913	1,10E-112	OPC2	Ncam1

8,27E-117	0,659497106	0,582	0,318	2,23E-112	OPC2	Glis3
3,55E-115	0,994094541	0,856	0,723	9,56E-111	OPC2	Pcdh7
4,57E-115	1,812457348	0,811	0,721	1,23E-110	OPC2	Nfasc
1,04E-113	0,603475205	0,668	0,417	2,81E-109	OPC2	Fchsd2
2,39E-111	0,54253035	0,545	0,3	6,44E-107	OPC2	Prex2
2,74E-111	0,514451814	0,775	0,556	7,38E-107	OPC2	Gpc5
1,29E-109	0,968061227	0,82	0,671	3,46E-105	OPC2	Dnm3
1,47E-101	0,668306207	0,82	0,68	3,95E-97	OPC2	Fmnl2
3,31E-101	0,614524963	0,55	0,338	8,92E-97	OPC2	Camsap2
1,16E-100	0,666739651	0,55	0,327	3,14E-96	OPC2	Mitf
2,77E-98	0,546524285	0,988	0,894	7,45E-94	OPC2	Zeb2
4,42E-96	0,537970446	0,962	0,815	1,19E-91	OPC2	Kirrel3
1,95E-95	0,516604418	0,719	0,554	5,26E-91	OPC2	Slc1a2
2,53E-93	0,569959333	0,564	0,358	6,81E-89	OPC2	Ccser2
6,65E-92	0,527574519	0,622	0,39	1,79E-87	OPC2	Prkd1
2,24E-91	0,663043377	0,963	0,95	6,02E-87	OPC2	Nav2
3,77E-87	0,513144662	0,608	0,4	1,02E-82	OPC2	Ophn1
4,51E-86	0,541861449	0,585	0,388	1,21E-81	OPC2	D630045J12Rik
1,18E-83	0,529748194	0,632	0,405	3,18E-79	OPC2	Stxbp6
3,46E-83	0,560881728	0,915	0,878	9,33E-79	OPC2	Trio
3,74E-83	0,952285436	0,997	0,984	1,01E-78	OPC2	Opcml
9,92E-83	0,717685185	0,761	0,643	2,67E-78	OPC2	Rfx3
1,29E-82	0,506816388	0,55	0,351	3,47E-78	OPC2	4430402I18Rik
9,76E-82	0,5143872	0,638	0,424	2,63E-77	OPC2	Pacrg
3,79E-80	0,658204288	0,713	0,563	1,02E-75	OPC2	Ankrd28
5,56E-79	0,641804776	0,712	0,524	1,50E-74	OPC2	Fam13c
9,24E-77	1,216033432	0,77	0,758	2,49E-72	OPC2	9530059O14Rik
6,23E-76	0,702953169	0,666	0,512	1,68E-71	OPC2	Slc4a4
2,97E-73	0,519899189	0,708	0,532	8,01E-69	OPC2	Cdc37l1
1,42E-72	0,542842562	0,757	0,612	3,82E-68	OPC2	Ago3
1,51E-70	0,52234782	0,928	0,847	4,08E-66	OPC2	Dscaml1
1,17E-69	0,595418134	0,743	0,631	3,16E-65	OPC2	Nav1
4,47E-67	0,621411411	0,542	0,343	1,21E-62	OPC2	Ephb1
2,19E-63	0,589561395	0,716	0,588	5,90E-59	OPC2	Cask
7,40E-58	0,81304848	0,735	0,674	1,99E-53	OPC2	Zdhhc14
1,47E-52	0,583495755	0,756	0,732	3,97E-48	OPC2	Ahcyl2
1,53E-40	0,566509404	0,75	0,717	4,13E-36	OPC2	Mical3
5,18E-32	0,550117095	0,511	0,411	1,40E-27	OPC2	Sh3rf1
0	1,61442914	0,99	0,325	0	Oligo1	Mbp
0	1,532092957	0,971	0,196	0	Oligo1	Plp1
0	1,493990476	0,975	0,327	0	Oligo1	St18
0	1,404598079	0,917	0,175	0	Oligo1	Prr5l

0	1,336807367	0,99	0,534	0	Oligo1	Dock10
0	1,319956704	0,916	0,3	0	Oligo1	Rnf220
0	1,285151211	0,998	0,733	0	Oligo1	Pde4b
0	1,199927847	0,817	0,148	0	Oligo1	Mobp
0	1,154510845	0,853	0,154	0	Oligo1	Mag
0	1,149161613	0,996	0,867	0	Oligo1	Tmeff2
0	1,138911959	0,995	0,645	0	Oligo1	Plcl1
0	1,134497872	0,991	0,561	0	Oligo1	Qk
0	1,114654366	0,795	0,145	0	Oligo1	Mog
0	1,113012029	0,806	0,149	0	Oligo1	Trf
0	1,074527227	0,772	0,141	0	Oligo1	Aspa
0	1,028123083	0,979	0,711	0	Oligo1	Phlpp1
0	1,003778389	0,979	0,683	0	Oligo1	Fnbp1
0	1,002172703	0,544	0,176	0	Oligo1	Apoe
0	1,001715357	0,926	0,376	0	Oligo1	Zfp536
0	0,995560513	0,762	0,238	0	Oligo1	Gm16168
0	0,991724922	0,67	0,224	0	Oligo1	Slc1a3
0	0,976966541	0,809	0,213	0	Oligo1	Phldb1
0	0,954567248	0,82	0,276	0	Oligo1	Erbin
0	0,929850634	0,827	0,314	0	Oligo1	Cdk19
0	0,924906819	0,707	0,135	0	Oligo1	D7Ertd443e
0	0,915794285	0,917	0,556	0	Oligo1	Grm3
0	0,908572429	0,902	0,473	0	Oligo1	07. Sep
0	0,904209947	0,831	0,4	0	Oligo1	Stxbp6
0	0,898828616	0,857	0,253	0	Oligo1	Enpp2
0	0,888489861	0,814	0,313	0	Oligo1	Tubb4a
0	0,888005399	0,685	0,13	0	Oligo1	Ugt8a
0	0,884511058	0,647	0,194	0	Oligo1	Lama2
0	0,88098901	0,73	0,144	0	Oligo1	Gjc3
0	0,872165775	0,909	0,506	0	Oligo1	Slc4a4
0	0,871695186	0,681	0,124	0	Oligo1	Sec14l5
0	0,868803542	0,644	0,162	0	Oligo1	Neat1
0	0,868059822	0,893	0,497	0	Oligo1	Frmd4b
0	0,868054464	0,687	0,164	0	Oligo1	Car2
0	0,86529292	0,683	0,128	0	Oligo1	Fa2h
0	0,86331324	0,824	0,358	0	Oligo1	Arhgap23
0	0,85816709	0,763	0,282	0	Oligo1	Ypel2
0	0,847227467	0,669	0,296	0	Oligo1	Prex2
0	0,843545928	0,988	0,754	0	Oligo1	Frmd5
0	0,838893889	0,951	0,684	0	Oligo1	Map7
0	0,836876946	0,693	0,183	0	Oligo1	Gab1
0	0,836645722	0,669	0,137	0	Oligo1	Pde8a
0	0,831521969	0,666	0,176	0	Oligo1	Daam2

0	0,829522345	0,985	0,861	0	Oligo1	Mast4
0	0,825407689	0,721	0,243	0	Oligo1	Plekhg1
0	0,823535749	0,633	0,138	0	Oligo1	Cdh19
0	0,818314101	0,517	0,16	0	Oligo1	Plpp3
0	0,812755799	0,624	0,12	0	Oligo1	Tspan2
0	0,802299715	0,97	0,763	0	Oligo1	St6galnac3
0	0,800585419	0,605	0,103	0	Oligo1	C030029H02Rik
0	0,797879095	0,787	0,352	0	Oligo1	Ppp1r16b
0	0,797607672	0,654	0,121	0	Oligo1	Plekhh1
0	0,794385018	0,997	0,983	0	Oligo1	Nkain2
0	0,789331373	0,98	0,722	0	Oligo1	Edil3
0	0,789142154	0,759	0,312	0	Oligo1	Glis3
0	0,781360079	0,597	0,105	0	Oligo1	1700047M11Rik
0	0,777135346	0,942	0,55	0	Oligo1	Maml2
0	0,771810077	0,694	0,192	0	Oligo1	04. Sep
0	0,770417399	0,734	0,155	0	Oligo1	Bcas1
0	0,765929699	0,784	0,352	0	Oligo1	Aatk
0	0,763475646	0,574	0,095	0	Oligo1	Opalin
0	0,762127009	0,742	0,316	0	Oligo1	Clmn
0	0,758119162	0,653	0,143	0	Oligo1	Gatm
0	0,734494175	0,977	0,723	0	Oligo1	Nfia
0	0,732626299	0,613	0,132	0	Oligo1	Ccp110
0	0,729380787	0,952	0,774	0	Oligo1	Dtna
0	0,722220826	0,532	0,096	0	Oligo1	Ninj2
0	0,721360215	0,616	0,19	0	Oligo1	Sytl2
0	0,711712676	0,997	0,944	0	Oligo1	Slc24a2
0	0,705429064	0,584	0,157	0	Oligo1	Hepacam
0	0,700436331	0,665	0,235	0	Oligo1	Dock1
0	0,700097669	0,895	0,325	0	Oligo1	Sox2ot
0	0,69364349	0,748	0,329	0	Oligo1	Ptprz1
0	0,690311959	0,587	0,134	0	Oligo1	Creb5
0	0,677183523	0,945	0,668	0	Oligo1	Dnm3
0	0,676001708	0,782	0,44	0	Oligo1	Pak1
0	0,66318701	0,773	0,385	0	Oligo1	Prkd1
0	0,663039685	0,709	0,326	0	Oligo1	Kif13a
0	0,657098844	0,642	0,246	0	Oligo1	Rab31
0	0,655330146	0,651	0,269	0	Oligo1	Sparcl1
0	0,654481521	0,846	0,528	0	Oligo1	Cdc37l1
0	0,647386492	0,505	0,081	0	Oligo1	Mal
0	0,640725724	0,886	0,642	0	Oligo1	Rtn4
0	0,625958218	0,595	0,217	0	Oligo1	Prox1
0	0,619225639	0,695	0,308	0	Oligo1	Slc12a2
0	0,614829307	0,533	0,117	0	Oligo1	Csrp1

0	0,613834413	1	0,987	0	Oligo1	Pcdh9
0	0,607291275	0,567	0,172	0	Oligo1	Arhgef10
0	0,603861169	0,57	0,214	0	Oligo1	Appl2
0	0,596971782	0,522	0,142	0	Oligo1	Scd2
0	0,595124039	0,965	0,867	0	Oligo1	Mapt
0	0,594085272	0,626	0,269	0	Oligo1	Usp54
0	0,590899864	0,56	0,126	0	Oligo1	Sox10
0	0,564543591	0,536	0,154	0	Oligo1	Pstpip2
0	0,563394174	0,597	0,229	0	Oligo1	Sema6a
0	0,545499374	0,535	0,164	0	Oligo1	Cntn2
0	0,539987366	0,516	0,111	0	Oligo1	Ptgds
0	0,529283281	0,577	0,218	0	Oligo1	Chd7
0	0,525927538	0,511	0,152	0	Oligo1	Wipf1
0	0,512611221	0,508	0,161	0	Oligo1	Rffl
6,21E-306	0,738292577	0,772	0,42	1,67E-301	Oligo1	Pacrg
5,78E-305	0,627552043	0,937	0,71	1,56E-300	Oligo1	Limch1
3,34E-304	0,750986526	0,796	0,45	9,01E-300	Oligo1	Myo6
5,00E-301	0,520339971	0,851	0,457	1,35E-296	Oligo1	Plxdc2
8,91E-301	0,664263281	0,757	0,412	2,40E-296	Oligo1	Kif13b
1,94E-296	0,658063657	0,565	0,23	5,22E-292	Oligo1	Acsl3
6,22E-294	0,564446911	0,679	0,302	1,68E-289	Oligo1	Stard13
6,43E-284	0,63279326	0,804	0,488	1,73E-279	Oligo1	Vmp1
1,66E-282	0,638412358	0,828	0,51	4,47E-278	Oligo1	Daam1
1,71E-282	0,654743593	0,779	0,4	4,60E-278	Oligo1	Pitpnc1
1,94E-282	0,628354703	0,834	0,543	5,23E-278	Oligo1	Bin1
1,25E-278	0,630536698	0,756	0,413	3,37E-274	Oligo1	Hipk2
3,31E-278	0,616211916	0,83	0,54	8,92E-274	Oligo1	Ttll7
2,79E-273	0,551666043	0,653	0,316	7,52E-269	Oligo1	Slain1
5,28E-270	0,522300656	0,568	0,239	1,42E-265	Oligo1	Smad7
6,61E-265	0,610009955	0,974	0,885	1,78E-260	Oligo1	Fgfr2
1,89E-264	0,703894327	0,72	0,353	5,08E-260	Oligo1	Cdh20
1,34E-262	0,81457633	0,806	0,448	3,60E-258	Oligo1	Npas3
2,38E-252	0,662582951	0,986	0,813	6,42E-248	Oligo1	Kirrel3
6,77E-250	0,59854433	0,804	0,498	1,82E-245	Oligo1	Pard3
3,19E-249	0,566178337	0,892	0,595	8,60E-245	Oligo1	Foxn3
4,46E-248	0,502869032	0,507	0,21	1,20E-243	Oligo1	Kndc1
6,59E-248	0,611488483	0,837	0,573	1,77E-243	Oligo1	Tulp4
1,92E-241	0,515220577	0,592	0,276	5,18E-237	Oligo1	Myo1d
4,41E-239	0,590451825	0,867	0,586	1,19E-234	Oligo1	Ano4
1,66E-238	0,588334146	0,836	0,577	4,48E-234	Oligo1	Mast3
3,04E-236	0,50826324	0,674	0,361	8,20E-232	Oligo1	Aplp1
5,22E-235	0,55253004	0,736	0,429	1,41E-230	Oligo1	Nbas
2,70E-230	0,574090391	0,999	0,809	7,28E-226	Oligo1	Zbtb20

6,09E-225	0,523270266	0,651	0,337	1,64E-220	Oligo1	Otud7b
6,17E-222	0,546802649	0,846	0,627	1,66E-217	Oligo1	Agap1
1,24E-219	0,61896014	0,684	0,363	3,34E-215	Oligo1	Ptn
5,20E-218	0,517381006	0,674	0,365	1,40E-213	Oligo1	Grb14
2,76E-217	0,534373903	0,867	0,681	7,44E-213	Oligo1	Tbc1d5
1,22E-211	0,524172995	0,984	0,834	3,29E-207	Oligo1	Ncam2
6,78E-208	0,549316502	0,923	0,769	1,83E-203	Oligo1	Sik3
8,65E-206	0,661600026	0,855	0,683	2,33E-201	Oligo1	Tspan7
1,77E-204	0,515311253	0,708	0,408	4,77E-200	Oligo1	Abhd17b
1,38E-200	0,536957159	0,911	0,67	3,72E-196	Oligo1	Ctnna3
2,88E-193	0,50348557	0,65	0,34	7,76E-189	Oligo1	Ephb1
3,01E-177	0,502088035	0,721	0,447	8,12E-173	Oligo1	Adk
3,40E-162	0,599621711	0,766	0,576	9,17E-158	Oligo1	Il1rap
1,54E-160	0,570733546	0,586	0,325	4,15E-156	Oligo1	Sema5a
7,22E-157	0,76030429	0,786	0,642	1,95E-152	Oligo1	Rfx3
6,70E-151	0,504611107	0,637	0,395	1,80E-146	Oligo1	Plekha1
8,16E-147	0,520143991	0,796	0,61	2,20E-142	Oligo1	Hdac8
1,38E-146	0,659087948	0,758	0,575	3,71E-142	Oligo1	Msi2
2,37E-142	0,528942164	0,646	0,401	6,39E-138	Oligo1	Thsd7a
2,41E-133	0,656824406	0,651	0,446	6,50E-129	Oligo1	Synpr
4,71E-97	1,063559357	0,632	0,554	1,27E-92	Oligo1	Slc1a2
2,23E-78	0,67787352	0,707	0,655	6,00E-74	Oligo1	Wdr17
2,85E-75	0,58347702	0,685	0,614	7,68E-71	Oligo1	Gm3764
1,59E-50	0,569888565	0,703	0,733	4,29E-46	Oligo1	Ahcyl2
1,32E-48	0,870154245	0,584	0,559	3,55E-44	Oligo1	Gpc5
0	3,312431511	1	0,708	0	Oligo2	Pde4b
0	3,303737335	0,987	0,123	0	Oligo2	Plp1
0	3,181579612	0,997	0,263	0	Oligo2	Mbp
0	2,856812929	1	0,855	0	Oligo2	Tmeff2
0	2,792183718	0,976	0,267	0	Oligo2	St18
0	2,744358114	0,939	0,104	0	Oligo2	Prr5l
0	2,524701204	0,924	0,242	0	Oligo2	Rnf220
0	2,352312271	1	0,986	0	Oligo2	Pcdh9
0	2,319018171	0,996	0,492	0	Oligo2	Dock10
0	2,312620425	0,864	0,081	0	Oligo2	Mobp
0	2,267620312	0,997	0,612	0	Oligo2	Plcl1
0	2,245872838	1	0,981	0	Oligo2	Nkain2
0	2,196609771	1	0,939	0	Oligo2	Slc24a2
0	2,191145853	0,873	0,088	0	Oligo2	Mag
0	2,177895275	0,997	0,521	0	Oligo2	Qk
0	2,168299398	0,996	0,696	0	Oligo2	Edil3
0	2,085261342	0,998	0,731	0	Oligo2	Frmd5

0	2,073627363	0,817	0,088	0	Oligo2	Trf
0	2,055628321	0,836	0,08	0	Oligo2	Mog
0	2,037753442	0,934	0,43	0	Oligo2	07. Sep
0	2,010022388	0,996	0,685	0	Oligo2	Phlpp1
0	2,005002497	0,901	0,192	0	Oligo2	Enpp2
0	1,992672448	0,992	0,655	0	Oligo2	Fnbp1
0	1,952202042	0,992	0,655	0	Oligo2	Map7
0	1,935984392	0,79	0,08	0	Oligo2	Aspa
0	1,879226756	0,991	0,849	0	Oligo2	Mast4
0	1,870835225	0,907	0,327	0	Oligo2	Zfp536
0	1,856305736	0,74	0,192	0	Oligo2	Gm16168
0	1,847540372	0,819	0,157	0	Oligo2	Phldb1
0	1,831122994	0,976	0,547	0	Oligo2	Ano4
0	1,801876236	0,745	0,078	0	Oligo2	D7Ertd443e
0	1,752039073	0,825	0,265	0	Oligo2	Tubb4a
0	1,749962008	0,761	0,087	0	Oligo2	Gjc3
0	1,747627514	0,992	0,637	0	Oligo2	Dnm3
0	1,738084691	0,806	0,228	0	Oligo2	Erbin
0	1,725115378	0,733	0,074	0	Oligo2	Ugt8a
0	1,694425152	0,834	0,314	0	Oligo2	Arhgap23
0	1,677526665	0,994	0,741	0	Oligo2	St6galnac3
0	1,676584606	0,711	0,083	0	Oligo2	Pde8a
0	1,629828018	0,903	0,272	0	Oligo2	Sox2ot
0	1,628619613	0,628	0,054	0	Oligo2	C030029H02Rik
0	1,616999847	0,961	0,611	0	Oligo2	Rtn4
0	1,616874409	0,703	0,07	0	Oligo2	Sec14l5
0	1,598809237	0,77	0,274	0	Oligo2	Cdk19
0	1,598277953	0,7	0,075	0	Oligo2	Fa2h
0	1,594548687	0,676	0,118	0	Oligo2	Car2
0	1,588541238	0,754	0,239	0	Oligo2	Ypel2
0	1,576271204	0,995	0,854	0	Oligo2	Mapt
0	1,575455393	1	0,995	0	Oligo2	Magi2
0	1,574769993	0,755	0,1	0	Oligo2	Bcas1
0	1,555093721	0,618	0,121	0	Oligo2	Neat1
0	1,553791156	0,651	0,071	0	Oligo2	Tspan2
0	1,546681481	0,894	0,506	0	Oligo2	Ttll7
0	1,544798616	0,993	0,759	0	Oligo2	Pak7
0	1,530731154	0,791	0,408	0	Oligo2	Pak1
0	1,520482075	0,673	0,069	0	Oligo2	Plekhh1
0	1,513687592	0,655	0,147	0	Oligo2	Sytl2
0	1,493949392	0,682	0,137	0	Oligo2	Gab1
0	1,479495008	0,765	0,314	0	Oligo2	Aatk
0	1,475713604	0,696	0,146	0	Oligo2	04. Sep

0	1,472316723	0,977	0,64	0	Oligo2	Ctnna3
0	1,451524978	0,6	0,048	0	Oligo2	Opalin
0	1,437763446	0,739	0,318	0	Oligo2	Ppp1r16b
0	1,434529741	0,66	0,096	0	Oligo2	Gatm
0	1,424949628	0,846	0,498	0	Oligo2	Cdc37l1
0	1,423386163	0,585	0,066	0	Oligo2	Ptgds
0	1,417144475	0,962	0,518	0	Oligo2	Grm3
0	1,412449179	0,624	0,086	0	Oligo2	Ccp110
0	1,404093209	0,871	0,511	0	Oligo2	Bin1
0	1,39903659	0,603	0,059	0	Oligo2	1700047M11Rik
0	1,398539301	0,605	0,096	0	Oligo2	Cdh19
0	1,388316447	0,991	0,812	0	Oligo2	Pex5l
0	1,383083346	0,75	0,381	0	Oligo2	Kif13b
0	1,356645406	0,796	0,46	0	Oligo2	Vmp1
0	1,34492574	0,548	0,037	0	Oligo2	Mal
0	1,330992071	0,591	0,092	0	Oligo2	Creb5
0	1,300142474	0,951	0,655	0	Oligo2	Tbc1d5
0	1,294757305	0,524	0,056	0	Oligo2	Ninj2
0	1,291304626	0,706	0,395	0	Oligo2	Pacrg
0	1,283974462	0,684	0,274	0	Oligo2	Slc12a2
0	1,283119991	0,953	0,611	0	Oligo2	Gm4258
0	1,279400459	0,93	0,639	0	Oligo2	Shtn1
0	1,276346839	0,998	0,874	0	Oligo2	Fgfr2
0	1,267760503	0,861	0,42	0	Oligo2	Plxdc2
0	1,26727981	0,665	0,206	0	Oligo2	Plekhg1
0	1,255309285	0,675	0,295	0	Oligo2	Kif13a
0	1,244994884	0,993	0,915	0	Oligo2	Prickle2
0	1,244754327	0,772	0,34	0	Oligo2	Elmo1
0	1,233113813	0,959	0,66	0	Oligo2	Klhl2
0	1,225389998	0,529	0,037	0	Oligo2	Cldn11
0	1,196925285	0,995	0,884	0	Oligo2	Zeb2
0	1,19647231	0,852	0,547	0	Oligo2	Tulp4
0	1,195391014	0,885	0,462	0	Oligo2	Frmd4b
0	1,160344338	0,98	0,748	0	Oligo2	Sik3
0	1,158192691	0,637	0,286	0	Oligo2	Slain1
0	1,1574686	0,585	0,196	0	Oligo2	Sema6a
0	1,153939179	0,901	0,598	0	Oligo2	Wnk1
0	1,146369771	0,978	0,762	0	Oligo2	Tcf12
0	1,143648112	0,997	0,927	0	Oligo2	Mbnl2
0	1,136084774	0,56	0,136	0	Oligo2	Arhgef10
0	1,1357073	0,557	0,087	0	Oligo2	Sox10
0	1,120162377	0,679	0,323	0	Oligo2	Cdh20
0	1,10664288	0,992	0,875	0	Oligo2	Exoc6b

0	1,104698775	0,593	0,247	0	Oligo2	Myo1d
0	1,102462103	0,851	0,548	0	Oligo2	Naaladl2
0	1,102269303	0,992	0,843	0	Oligo2	Clasp2
0	1,100433404	0,949	0,696	0	Oligo2	Nfasc
0	1,097744285	0,988	0,828	0	Oligo2	Atp8a1
0	1,092424287	0,741	0,49	0	Oligo2	Daam1
0	1,089710826	0,508	0,122	0	Oligo2	Pstpip2
0	1,087164606	0,711	0,327	0	Oligo2	Sorcs1
0	1,084170074	0,665	0,409	0	Oligo2	Nbas
0	1,074752789	0,971	0,685	0	Oligo2	Limch1
0	1,063303237	0,523	0,131	0	Oligo2	Cntn2
0	1,062969177	0,867	0,549	0	Oligo2	Mast3
0	1,058421796	0,533	0,106	0	Oligo2	Scd2
0	1,054550125	0,619	0,312	0	Oligo2	Otud7b
0	1,050402184	0,664	0,392	0	Oligo2	Hipk2
0	1,04879184	0,592	0,378	0	Oligo2	Plekha1
0	1,031486822	0,567	0,142	0	Oligo2	Daam2
0	1,028395012	0,578	0,217	0	Oligo2	Rab31
0	1,027457155	0,994	0,898	0	Oligo2	Kif1b
0	1,018158738	0,895	0,602	0	Oligo2	Agap1
0	1,016621463	0,996	0,905	0	Oligo2	Ncam1
0	1,014157512	0,606	0,292	0	Oligo2	Clmn
0	1,01289152	0,573	0,242	0	Oligo2	Usp54
0	1,01200349	0,661	0,487	0	Oligo2	Zdhhc20
0	1,006054094	0,793	0,539	0	Oligo2	Ankrd28
0	0,998645359	0,598	0,308	0	Oligo2	Dip2a
0	0,993557428	0,538	0,212	0	Oligo2	Smad7
0	0,991789875	0,968	0,323	0	Oligo2	Erbb4
0	0,988553244	0,99	0,87	0	Oligo2	App
0	0,971601866	0,969	0,722	0	Oligo2	Hecw2
0	0,970782745	0,953	0,635	0	Oligo2	Ptprk
0	0,969998805	0,702	0,429	0	Oligo2	Myo6
0	0,966559649	0,975	0,758	0	Oligo2	Baz2b
0	0,95991308	0,99	0,873	0	Oligo2	Tmcc1
0	0,953581571	0,998	0,914	0	Oligo2	Dst
0	0,931016609	0,686	0,432	0	Oligo2	Sh3d19
0	0,930084279	0,994	0,911	0	Oligo2	Cdc42bpa
0	0,918836989	0,566	0,207	0	Oligo2	Dock1
0	0,910871332	0,628	0,466	0	Oligo2	Tmod2
0	0,910448786	0,502	0,192	0	Oligo2	Rcbtb1
0	0,898078576	0,583	0,347	0	Oligo2	Grb14
0	0,897320966	0,95	0,676	0	Oligo2	Dlg1
0	0,89486769	0,608	0,392	0	Oligo2	Abhd17b

0	0,885650578	0,567	0,368	0	Oligo2	Lpgat1
0	0,884466408	0,557	0,301	0	Oligo2	Nrbp2
0	0,876497859	0,514	0,261	0	Oligo2	Ywhaq
0	0,875600229	0,993	0,838	0	Oligo2	Tmem178b
0	0,875561903	1	0,951	0	Oligo2	Ptprd
0	0,874400505	0,913	0,692	0	Oligo2	Pakap
0	0,871823759	0,59	0,405	0	Oligo2	Ttll5
0	0,862857328	0,812	0,58	0	Oligo2	Map4
0	0,862066165	0,558	0,345	0	Oligo2	Aplp1
0	0,859931585	0,638	0,464	0	Oligo2	Ppp2r3a
0	0,847829109	0,887	0,568	0	Oligo2	Foxn3
0	0,839352033	0,973	0,773	0	Oligo2	Dip2b
0	0,838744865	0,83	0,6	0	Oligo2	Slc44a1
0	0,836456648	0,71	0,515	0	Oligo2	Srcin1
0	0,836245558	0,563	0,374	0	Oligo2	Slc38a2
0	0,806049433	0,998	0,962	0	Oligo2	Lrp1b
0	0,79148039	0,565	0,366	0	Oligo2	Pip4k2a
0	0,787055321	0,994	0,886	0	Oligo2	Ptk2
0	0,786690153	0,527	0,337	0	Oligo2	5031439G07Rik
0	0,782359855	0,781	0,581	0	Oligo2	Itch
0	0,781839935	0,613	0,342	0	Oligo2	Ptn
0	0,780173108	0,999	0,994	0	Oligo2	Ank2
0	0,767008976	0,905	0,649	0	Oligo2	Zdhhc14
0	0,766548536	0,988	0,829	0	Oligo2	Unc5c
0	0,762386277	0,523	0,339	0	Oligo2	Rnf13
0	0,750769972	0,54	0,36	0	Oligo2	Picalm
0	0,748823012	0,994	0,819	0	Oligo2	Ncam2
0	0,747000237	0,532	0,258	0	Oligo2	Adamtsl1
0	0,743553601	0,729	0,535	0	Oligo2	Dleu2
0	0,736709499	0,903	0,717	0	Oligo2	Zfp638
0	0,736088379	0,536	0,324	0	Oligo2	Ephb1
0	0,730336232	0,977	0,833	0	Oligo2	Dscaml1
0	0,727770906	1	1	0	Oligo2	Malat1
0	0,726472211	0,577	0,349	0	Oligo2	Tmcc3
0	0,725593085	0,531	0,359	0	Oligo2	Shisa4
0	0,713639151	0,914	0,702	0	Oligo2	Pkp4
0	0,705557111	0,535	0,423	0	Oligo2	Ankib1
0	0,702187381	0,969	0,793	0	Oligo2	Ssh2
0	0,701513559	0,531	0,388	0	Oligo2	Cyth1
0	0,686724125	0,978	0,856	0	Oligo2	Trim2
0	0,67824967	1	0,989	0	Oligo2	Ank3
0	0,67701732	0,978	0,695	0	Oligo2	Mir100hg
0	0,676598775	0,71	0,566	0	Oligo2	Itsn2

0	0,670073526	0,639	0,489	0	Oligo2	Pard3
0	0,666338804	0,71	0,559	0	Oligo2	Fryl
0	0,663877005	0,958	0,699	0	Oligo2	Cdh10
0	0,658371432	0,977	0,844	0	Oligo2	Kif21a
0	0,653034585	0,842	0,55	0	Oligo2	Il1rap
0	0,647125378	0,962	0,756	0	Oligo2	Gnao1
0	0,622772961	0,644	0,52	0	Oligo2	Map4k4
0	0,617925886	0,88	0,659	0	Oligo2	Fmnl2
0	0,611347128	0,6	0,527	0	Oligo2	Hook3
0	0,603231399	0,577	0,493	0	Oligo2	Gas7
0	0,597402587	0,985	0,863	0	Oligo2	Gpm6b
0	0,597290018	0,536	0,374	0	Oligo2	Tmtc2
0	0,595336363	0,543	0,45	0	Oligo2	Epb41l3
0	0,565493106	0,502	0,381	0	Oligo2	4930419G24Rik
0	0,564292673	0,506	0,393	0	Oligo2	Arhgef28
0	0,560483831	0,577	0,514	0	Oligo2	Acaca
0	0,559780121	0,642	0,558	0	Oligo2	Ablim2
0	0,558932974	0,874	0,69	0	Oligo2	Bcas3
0	0,557483072	0,789	0,638	0	Oligo2	Ralgps1
0	0,554108233	0,825	0,636	0	Oligo2	Apbb2
0	0,553800191	0,501	0,398	0	Oligo2	Osbpl1a
0	0,55368131	0,902	0,707	0	Oligo2	Ddx17
0	0,550833705	0,609	0,527	0	Oligo2	Neo1
0	0,550704022	0,904	0,725	0	Oligo2	Pafah1b1
0	0,52807966	0,988	0,882	0	Oligo2	4930402H24Rik
0	0,527585994	0,736	0,602	0	Oligo2	Trim35
0	0,526121416	0,705	0,602	0	Oligo2	Arid4b
0	0,519821263	0,767	0,624	0	Oligo2	Elavl3
0	0,508564892	0,688	0,582	0	Oligo2	Ank
0	0,507207257	0,617	0,385	0	Oligo2	Stxbp6
8,39E-286	0,549496763	0,505	0,458	2,26E-281	Oligo2	Map4k5

0	1,972886247	0,989	0,602	0	Dentate Gyrus	Rfx3
0	1,822446631	0,974	0,702	0	Dentate Gyrus	Ahcyl2
0	1,759477928	1	0,87	0	Dentate Gyrus	Lrrtm4
0	1,684826819	0,99	0,696	0	Dentate Gyrus	Tafa2
0	1,623787553	1	0,937	0	Dentate Gyrus	Ppfia2
0	1,605006791	0,967	0,631	0	Dentate Gyrus	Dgkh
0	1,541040347	0,899	0,249	0	Dentate Gyrus	Glis3
0	1,487765017	0,917	0,345	0	Dentate Gyrus	Stxbp6
0	1,435246172	0,951	0,607	0	Dentate Gyrus	Ccdc85a
0	1,417598987	0,907	0,258	0	Dentate Gyrus	Sema5a
0	1,412306628	0,929	0,391	0	Dentate Gyrus	Synpr

0	1,398728267	0,917	0,363	0	Dentate Gyrus	Cdh9
0	1,307362372	0,961	0,508	0	Dentate Gyrus	Maml2
0	1,30621147	0,989	0,644	0	Dentate Gyrus	Trpm3
0	1,27293663	0,976	0,719	0	Dentate Gyrus	Epha7
0	1,270274016	0,987	0,86	0	Dentate Gyrus	Plxna4
0	1,257075604	0,999	0,896	0	Dentate Gyrus	Slit3
0	1,254274479	0,976	0,816	0	Dentate Gyrus	Btbd9
0	1,253905005	1	0,966	0	Dentate Gyrus	Erc2
0	1,246518469	0,892	0,345	0	Dentate Gyrus	Thsd7a
0	1,244227659	0,982	0,67	0	Dentate Gyrus	Zfpm2
0	1,240301933	0,963	0,752	0	Dentate Gyrus	Sphkap
0	1,23274669	0,974	0,677	0	Dentate Gyrus	Tmem108
0	1,231119871	1	0,845	0	Dentate Gyrus	Lingo2
0	1,230544779	1	0,965	0	Dentate Gyrus	Ppp3ca
0	1,230513695	0,907	0,405	0	Dentate Gyrus	Tiam1
0	1,229922473	0,982	0,414	0	Dentate Gyrus	Adarb2
0	1,225322839	0,99	0,805	0	Dentate Gyrus	Gm20754
0	1,215584409	0,916	0,51	0	Dentate Gyrus	Shisa9
0	1,21061256	0,774	0,195	0	Dentate Gyrus	C1ql3
0	1,203549696	1	0,951	0	Dentate Gyrus	Dab1
0	1,191462526	0,977	0,746	0	Dentate Gyrus	Mctp1
0	1,182715638	0,91	0,585	0	Dentate Gyrus	Pip5k1b
0	1,179829686	0,903	0,54	0	Dentate Gyrus	Il1rap
0	1,179193737	0,976	0,732	0	Dentate Gyrus	Cntnap5a
0	1,171982951	0,697	0,203	0	Dentate Gyrus	Trpc6
0	1,166313105	0,881	0,494	0	Dentate Gyrus	Lyst
0	1,164619563	0,925	0,463	0	Dentate Gyrus	Slc4a4
0	1,141949272	1	0,945	0	Dentate Gyrus	Ryr2
0	1,137859953	0,997	0,935	0	Dentate Gyrus	Prkce
0	1,125159612	0,955	0,691	0	Dentate Gyrus	Grm1
0	1,121115285	0,989	0,826	0	Dentate Gyrus	Galnt17
0	1,116806755	0,811	0,342	0	Dentate Gyrus	Pdzd2
0	1,115847158	0,998	0,794	0	Dentate Gyrus	Kirrel3
0	1,086642436	0,969	0,86	0	Dentate Gyrus	Rasal2
0	1,085752275	1	0,947	0	Dentate Gyrus	Negr1
0	1,084338942	0,995	0,904	0	Dentate Gyrus	Cacna1c
0	1,074179291	0,658	0,194	0	Dentate Gyrus	Chst9
0	1,067057706	1	0,789	0	Dentate Gyrus	Zbtb20
0	1,062714598	0,911	0,694	0	Dentate Gyrus	Gm28376
0	1,048140642	1	0,986	0	Dentate Gyrus	Auts2
0	1,0223302	1	0,952	0	Dentate Gyrus	Grin2a
0	0,988005689	0,825	0,439	0	Dentate Gyrus	Jph1
0	0,980323694	0,995	0,891	0	Dentate Gyrus	Cacna1e

0	0,972606077	0,984	0,822	0	Dentate Gyrus	Nr3c2
0	0,970141111	0,883	0,548	0	Dentate Gyrus	2010300C02Rik
0	0,965064908	0,968	0,88	0	Dentate Gyrus	Map1b
0	0,963130138	0,958	0,804	0	Dentate Gyrus	Cdh8
0	0,959701711	0,894	0,633	0	Dentate Gyrus	Camk2b
0	0,959690386	1	0,956	0	Dentate Gyrus	Kcnip4
0	0,94911111	0,998	0,951	0	Dentate Gyrus	Dpp6
0	0,948894542	0,789	0,34	0	Dentate Gyrus	Pitpnm2
0	0,944125406	0,998	0,936	0	Dentate Gyrus	Camk2a
0	0,940657156	0,993	0,854	0	Dentate Gyrus	Egfem1
0	0,938484097	0,81	0,561	0	Dentate Gyrus	Cntnap5b
0	0,934935678	0,987	0,909	0	Dentate Gyrus	Ntrk3
0	0,9286368	0,973	0,846	0	Dentate Gyrus	Mmp16
0	0,92860797	0,922	0,737	0	Dentate Gyrus	Kcnj6
0	0,928007324	0,812	0,34	0	Dentate Gyrus	Prkd1
0	0,919573462	0,743	0,321	0	Dentate Gyrus	Cyp7b1
0	0,911466057	1	0,987	0	Dentate Gyrus	Dlgap1
0	0,908586307	0,916	0,726	0	Dentate Gyrus	Vps13c
0	0,908048927	0,864	0,5	0	Dentate Gyrus	Tanc1
0	0,906747624	0,806	0,34	0	Dentate Gyrus	Maml3
0	0,894789129	1	0,962	0	Dentate Gyrus	Lrp1b
0	0,894266444	1	0,97	0	Dentate Gyrus	Kcnd2
0	0,893355567	1	0,968	0	Dentate Gyrus	Kalrn
0	0,89134526	0,779	0,343	0	Dentate Gyrus	Nrg2
0	0,887497455	0,81	0,36	0	Dentate Gyrus	Ptchd4
0	0,882414746	0,806	0,404	0	Dentate Gyrus	Plekhg5
0	0,878507483	0,783	0,391	0	Dentate Gyrus	Adcy1
0	0,875730896	0,772	0,407	0	Dentate Gyrus	Slc7a14
0	0,874300449	0,832	0,486	0	Dentate Gyrus	Slit1
0	0,873824493	0,67	0,169	0	Dentate Gyrus	Prox1
0	0,872063102	0,692	0,269	0	Dentate Gyrus	Htr4
0	0,870477998	0,951	0,831	0	Dentate Gyrus	Nsf
0	0,868855935	0,794	0,368	0	Dentate Gyrus	Itga8
0	0,86509862	1	0,928	0	Dentate Gyrus	Gria1
0	0,859379101	0,916	0,688	0	Dentate Gyrus	Ak5
0	0,859199162	0,888	0,627	0	Dentate Gyrus	Slc2a13
0	0,818547115	0,988	0,891	0	Dentate Gyrus	Clstn2
0	0,818096777	0,933	0,81	0	Dentate Gyrus	Fam135b
0	0,816423333	0,997	0,949	0	Dentate Gyrus	Lrfn5
0	0,809257931	0,999	0,958	0	Dentate Gyrus	Grik2
0	0,808300795	0,901	0,702	0	Dentate Gyrus	Tenm1
0	0,797674055	0,892	0,665	0	Dentate Gyrus	Slc35f3
0	0,793164218	0,95	0,843	0	Dentate Gyrus	Rasgrf1

0	0,792464122	0,681	0,253	0	Dentate Gyrus	Vav3
0	0,783993385	0,864	0,46	0	Dentate Gyrus	Frmd4b
0	0,782953236	1	0,98	0	Dentate Gyrus	Fgf14
0	0,777487892	1	0,978	0	Dentate Gyrus	Celf2
0	0,777458398	0,736	0,374	0	Dentate Gyrus	Pgbd5
0	0,776506122	0,603	0,178	0	Dentate Gyrus	F730043M19Rik
0	0,776487369	0,999	0,96	0	Dentate Gyrus	Il1rapl1
0	0,775696826	0,819	0,508	0	Dentate Gyrus	Ptk2b
0	0,774716572	0,998	0,95	0	Dentate Gyrus	Cttnbp2
0	0,771778566	0,92	0,783	0	Dentate Gyrus	Mrtfb
0	0,771496573	0,683	0,292	0	Dentate Gyrus	Adamts17
0	0,762048571	0,776	0,416	0	Dentate Gyrus	4930509J09Rik
0	0,758619663	0,734	0,358	0	Dentate Gyrus	Olfm1
0	0,757356702	0,724	0,306	0	Dentate Gyrus	Pde7b
0	0,753512713	1	0,99	0	Dentate Gyrus	Cadm2
0	0,748332188	0,791	0,509	0	Dentate Gyrus	Lgi1
0	0,744724908	0,937	0,828	0	Dentate Gyrus	Kcnj3
0	0,737409694	0,996	0,949	0	Dentate Gyrus	Gabrb3
0	0,736832264	0,79	0,472	0	Dentate Gyrus	Wipf3
0	0,736223942	0,967	0,49	0	Dentate Gyrus	Dock10
0	0,728994141	0,858	0,682	0	Dentate Gyrus	Add2
0	0,727696898	0,849	0,647	0	Dentate Gyrus	Dapk1
0	0,717874713	0,999	0,99	0	Dentate Gyrus	Mycbp2
0	0,716820195	0,779	0,431	0	Dentate Gyrus	Slc44a5
0	0,708877869	0,9	0,626	0	Dentate Gyrus	Iqgap2
0	0,706166485	0,593	0,203	0	Dentate Gyrus	Kif26b
0	0,700817414	0,932	0,86	0	Dentate Gyrus	Nedd4l
0	0,700111345	0,788	0,462	0	Dentate Gyrus	Dpyd
0	0,698927956	0,891	0,685	0	Dentate Gyrus	A830018L16Rik
0	0,697661398	0,981	0,902	0	Dentate Gyrus	Nebl
0	0,696159147	0,981	0,928	0	Dentate Gyrus	Dgki
0	0,69588767	0,893	0,733	0	Dentate Gyrus	Ano3
0	0,694620837	0,912	0,813	0	Dentate Gyrus	Rgs7bp
0	0,693384896	0,686	0,371	0	Dentate Gyrus	Cblb
0	0,692123373	0,752	0,41	0	Dentate Gyrus	Ntng1
0	0,691188319	1	0,96	0	Dentate Gyrus	Dlgap2
0	0,673864533	0,968	0,834	0	Dentate Gyrus	Nell2
0	0,670940318	0,726	0,347	0	Dentate Gyrus	Bcl11b
0	0,670891594	0,99	0,914	0	Dentate Gyrus	Kctd16
0	0,668759107	0,704	0,4	0	Dentate Gyrus	Pcdh17
0	0,664117333	0,877	0,705	0	Dentate Gyrus	Rtn1
0	0,658297228	0,621	0,326	0	Dentate Gyrus	Cep112
0	0,654964227	0,868	0,767	0	Dentate Gyrus	Ptprj

0	0,651508864	0,504	0,155	0	Dentate Gyrus	Calb1
0	0,649425407	0,639	0,333	0	Dentate Gyrus	Sntb2
0	0,648049861	0,988	0,818	0	Dentate Gyrus	Ncam2
0	0,644111634	0,601	0,242	0	Dentate Gyrus	Rasgrf2
0	0,642422318	0,821	0,62	0	Dentate Gyrus	Elmod1
0	0,641423403	0,911	0,806	0	Dentate Gyrus	Chn1
0	0,639344717	0,702	0,408	0	Dentate Gyrus	Snca
0	0,637537993	0,941	0,879	0	Dentate Gyrus	Plekha5
0	0,630818103	0,676	0,364	0	Dentate Gyrus	Scn3a
0	0,627807497	0,8	0,552	0	Dentate Gyrus	Syt7
0	0,627267095	0,714	0,403	0	Dentate Gyrus	Osbp2
0	0,626862222	0,878	0,699	0	Dentate Gyrus	Epha4
0	0,626683434	0,81	0,673	0	Dentate Gyrus	Ralgapa2
0	0,624356577	1	0,989	0	Dentate Gyrus	Nbea
0	0,622827479	0,824	0,66	0	Dentate Gyrus	Cers6
0	0,619885815	0,644	0,336	0	Dentate Gyrus	Arhgap20
0	0,619794154	0,637	0,351	0	Dentate Gyrus	Clvs2
0	0,607103594	0,721	0,451	0	Dentate Gyrus	Ppp1r13b
0	0,60356068	0,877	0,78	0	Dentate Gyrus	Eml5
0	0,603268087	0,873	0,699	0	Dentate Gyrus	Nkain3
0	0,597784745	0,558	0,327	0	Dentate Gyrus	Hectd2
0	0,597716282	0,967	0,882	0	Dentate Gyrus	Dscam
0	0,596172459	0,532	0,194	0	Dentate Gyrus	Mkx
0	0,595077316	0,629	0,341	0	Dentate Gyrus	Gabra4
0	0,594807444	0,737	0,429	0	Dentate Gyrus	Lmo7
0	0,593633835	0,712	0,433	0	Dentate Gyrus	Gfod1
0	0,593104489	0,87	0,79	0	Dentate Gyrus	Tmem132b
0	0,591788827	0,547	0,226	0	Dentate Gyrus	Gnal
0	0,59076646	0,957	0,902	0	Dentate Gyrus	Cacna1d
0	0,586978793	0,987	0,948	0	Dentate Gyrus	Tnik
0	0,584284027	1	0,978	0	Dentate Gyrus	Fam155a
0	0,583415261	0,712	0,464	0	Dentate Gyrus	Gabrg2
0	0,581330908	1	0,987	0	Dentate Gyrus	Dlg2
0	0,57666619	0,586	0,275	0	Dentate Gyrus	Rras2
0	0,574961261	0,548	0,227	0	Dentate Gyrus	Cplx2
0	0,574246266	0,811	0,635	0	Dentate Gyrus	Nrp1
0	0,574210572	0,866	0,757	0	Dentate Gyrus	Diaph2
0	0,573958847	0,52	0,191	0	Dentate Gyrus	Ablim3
0	0,573592654	0,865	0,694	0	Dentate Gyrus	Kcnt2
0	0,572857614	0,922	0,837	0	Dentate Gyrus	Sorbs1
0	0,572323636	0,673	0,389	0	Dentate Gyrus	Carmil1
0	0,565239241	0,715	0,432	0	Dentate Gyrus	Fstl4
0	0,563620669	0,982	0,887	0	Dentate Gyrus	Nfib

0	0,563511188	0,873	0,795	0	Dentate Gyrus	Sybu
0	0,561669628	0,957	0,894	0	Dentate Gyrus	Sorbs2
0	0,559852531	0,915	0,836	0	Dentate Gyrus	Rbfox3
0	0,557935303	0,527	0,207	0	Dentate Gyrus	Kcnc3
0	0,556332782	0,905	0,799	0	Dentate Gyrus	Atrnl1
0	0,55470073	0,696	0,405	0	Dentate Gyrus	Baiap2
0	0,55410238	0,788	0,632	0	Dentate Gyrus	Sez6l
0	0,552943803	1	0,989	0	Dentate Gyrus	Ank3
0	0,551649905	0,769	0,545	0	Dentate Gyrus	Wasf1
0	0,5415687	0,737	0,494	0	Dentate Gyrus	Ndrg4
0	0,53856426	0,953	0,848	0	Dentate Gyrus	Tnr
0	0,535457758	0,858	0,728	0	Dentate Gyrus	Gabra2
0	0,534425228	0,702	0,44	0	Dentate Gyrus	Gm15738
0	0,533593224	0,548	0,259	0	Dentate Gyrus	Btbd3
0	0,533451711	0,611	0,326	0	Dentate Gyrus	Mtmr12
0	0,532579819	0,727	0,447	0	Dentate Gyrus	Sipa1l3
0	0,531550561	0,897	0,821	0	Dentate Gyrus	Gabbr2
0	0,530056401	0,771	0,516	0	Dentate Gyrus	Rnf112
0	0,528768337	0,65	0,404	0	Dentate Gyrus	Arsb
0	0,520515238	0,729	0,431	0	Dentate Gyrus	Spock3
0	0,518536245	1	0,97	0	Dentate Gyrus	Lrrc7
0	0,518465798	0,979	0,898	0	Dentate Gyrus	Cacna2d1
0	0,516860698	0,635	0,33	0	Dentate Gyrus	Ptpre
0	0,51142724	0,999	0,983	0	Dentate Gyrus	Gria2
0	0,51123118	0,846	0,746	0	Dentate Gyrus	Cdh12
0	0,511106271	0,762	0,567	0	Dentate Gyrus	Kcnd3
0	0,509255291	0,866	0,743	0	Dentate Gyrus	Dgkg
0	0,508931802	0,657	0,374	0	Dentate Gyrus	Xpr1
0	0,508170362	0,912	0,877	0	Dentate Gyrus	Pde4dip
0	0,506338576	0,972	0,927	0	Dentate Gyrus	Syne1
0	0,50469735	0,582	0,366	0	Dentate Gyrus	Syn3
0	0,503025986	0,816	0,702	0	Dentate Gyrus	Pakap
0	0,502204225	0,954	0,907	0	Dentate Gyrus	Cdk14
0	0,500977403	0,894	0,833	0	Dentate Gyrus	Rapgef2

0	1,836698827	0,997	0,811	0	Intermediate cells	Zbtb20
0	1,547422658	0,994	0,896	0	Intermediate cells	Nfib
0	1,525462196	0,994	0,936	0	Intermediate cells	Mir99ahg
0	1,27423957	0,71	0,272	0	Intermediate cells	Ttc28
0	1,136908105	0,974	0,517	0	Intermediate cells	Gm42418
0	1,111250749	0,561	0,149	0	Intermediate cells	Sncaip
1,47E-307	1,292845034	0,987	0,726	3,97E-303	Intermediate cells	Nfia
2,71E-302	1,202482265	0,997	0,988	7,30E-298	Intermediate cells	Ctnna2

2,26E-286	1,11008312	0,999	0,986	6,08E-282	Intermediate cells	Tcf4
3,15E-285	1,694836848	0,989	0,895	8,47E-281	Intermediate cells	Dcc
1,53E-215	1,11640121	0,918	0,763	4,12E-211	Intermediate cells	Nol4
6,34E-207	1,111273079	0,945	0,703	1,71E-202	Intermediate cells	Zfpm2
9,01E-199	0,965384832	0,999	0,988	2,43E-194	Intermediate cells	Auts2
3,86E-184	0,909379764	0,991	0,935	1,04E-179	Intermediate cells	Pbx1
4,51E-175	0,858531505	0,55	0,237	1,21E-170	Intermediate cells	Mtss1
4,57E-175	0,903971276	0,538	0,221	1,23E-170	Intermediate cells	Prox1
1,69E-165	0,573586521	1	0,991	4,55E-161	Intermediate cells	Nrxn1
1,66E-159	1,214353929	0,63	0,327	4,47E-155	Intermediate cells	Sema5a
8,14E-158	1,180141363	0,52	0,245	2,19E-153	Intermediate cells	Ptpro
2,87E-156	0,669781393	0,991	0,934	7,73E-152	Intermediate cells	Map2
3,19E-128	0,745065536	0,885	0,809	8,59E-124	Intermediate cells	C130071C03Rik
6,67E-118	1,012204341	0,679	0,514	1,80E-113	Intermediate cells	Plxna2
1,13E-116	0,799049062	0,767	0,631	3,04E-112	Intermediate cells	Nav1
5,02E-116	0,691058209	0,935	0,867	1,35E-111	Intermediate cells	Nedd4l
1,66E-111	0,729684892	0,856	0,58	4,47E-107	Intermediate cells	Pcdh15
1,72E-103	0,658809043	0,824	0,66	4,64E-99	Intermediate cells	Klf12
5,52E-100	0,528087472	0,987	0,913	1,49E-95	Intermediate cells	Ncam1
3,98E-99	0,733924852	0,928	0,736	1,07E-94	Intermediate cells	Sema6d
7,76E-97	0,747396746	0,526	0,3	2,09E-92	Intermediate cells	Nnat
3,17E-94	0,673501403	0,518	0,297	8,53E-90	Intermediate cells	Zmiz1
3,95E-93	0,695427234	0,626	0,451	1,06E-88	Intermediate cells	Nfix
3,72E-91	0,747920436	0,687	0,518	1,00E-86	Intermediate cells	Bach2
2,50E-88	0,545745946	0,891	0,782	6,73E-84	Intermediate cells	Hnrnpa2b1
6,88E-85	0,826366012	0,708	0,503	1,85E-80	Intermediate cells	Frmd4b
5,67E-79	0,632467919	0,57	0,375	1,53E-74	Intermediate cells	Fgf13
1,20E-78	0,638186082	0,982	0,971	3,23E-74	Intermediate cells	Ccser1
1,81E-76	0,885981658	0,872	0,771	4,86E-72	Intermediate cells	Mctp1
4,24E-76	0,606888097	0,787	0,68	1,14E-71	Intermediate cells	Fmnl2
5,26E-74	0,681940769	0,967	0,931	1,42E-69	Intermediate cells	Setbp1
9,32E-74	0,531176987	0,973	0,952	2,51E-69	Intermediate cells	Tanc2
1,73E-67	0,617838104	0,556	0,389	4,66E-63	Intermediate cells	C530008M17Rik
2,17E-66	0,654409516	0,582	0,446	5,86E-62	Intermediate cells	Lncppara
1,53E-64	0,934557775	0,877	0,758	4,12E-60	Intermediate cells	Cntnap5a
7,70E-63	0,561211086	0,824	0,783	2,07E-58	Intermediate cells	Ptprs
1,03E-61	0,574359524	0,961	0,913	2,77E-57	Intermediate cells	Cacna1c
1,42E-59	0,653912566	0,516	0,353	3,83E-55	Intermediate cells	Tead1
3,65E-59	0,611176028	0,514	0,356	9,83E-55	Intermediate cells	Sh3kbp1
4,03E-56	0,54923997	0,967	0,909	1,09E-51	Intermediate cells	Kcnb2
1,01E-53	0,500695332	0,684	0,594	2,72E-49	Intermediate cells	Igf1r
1,30E-52	0,729470166	0,706	0,644	3,51E-48	Intermediate cells	Zeb1
1,44E-48	0,547812009	0,552	0,391	3,87E-44	Intermediate cells	Maml3

3,42E-48	0,530045663	0,915	0,814	9,22E-44	Intermediate cells	Robo1
4,29E-46	0,552622439	0,619	0,534	1,16E-41	Intermediate cells	Neo1
1,12E-43	0,501391261	0,578	0,477	3,00E-39	Intermediate cells	Akap9
9,76E-41	0,64792302	0,851	0,723	2,63E-36	Intermediate cells	Mir100hg
1,20E-39	0,552625072	0,754	0,718	3,23E-35	Intermediate cells	Hecw1
1,61E-39	0,528629251	0,625	0,555	4,33E-35	Intermediate cells	Dleu2
1,99E-36	0,528009606	0,671	0,646	5,36E-32	Intermediate cells	Gramd1b
1,01E-27	0,620317157	0,694	0,654	2,73E-23	Intermediate cells	Nrp1

0	2,923935098	0,983	0,281	0	Pyramidal (NFM)	Cdh18
0	2,742610307	1	0,629	0	Pyramidal (NFM)	Dpp10
0	2,14163021	0,999	0,55	0	Pyramidal (NFM)	Hs3st4
0	2,065262193	1	0,738	0	Pyramidal (NFM)	Etl4
0	1,767114244	0,928	0,309	0	Pyramidal (NFM)	Garnl3
0	1,732773507	0,952	0,367	0	Pyramidal (NFM)	Dlc1
0	1,654461259	0,732	0,086	0	Pyramidal (NFM)	Foxp2
0	1,649197906	0,867	0,444	0	Pyramidal (NFM)	Thsd7b
0	1,579886958	0,989	0,705	0	Pyramidal (NFM)	Pde1a
0	1,55715714	0,725	0,451	0	Pyramidal (NFM)	Brinp3
0	1,507430439	0,871	0,282	0	Pyramidal (NFM)	Grm8
0	1,41044204	0,841	0,228	0	Pyramidal (NFM)	Sdk2
0	1,409757958	0,852	0,216	0	Pyramidal (NFM)	Grik3
0	1,38491337	0,93	0,422	0	Pyramidal (NFM)	Tle4
0	1,368442222	0,837	0,289	0	Pyramidal (NFM)	Htr1f
0	1,367278118	0,954	0,518	0	Pyramidal (NFM)	Slc35f1
0	1,343621072	0,938	0,508	0	Pyramidal (NFM)	Chsy3
0	1,333883716	0,998	0,949	0	Pyramidal (NFM)	Frmpd4
0	1,310954091	0,979	0,745	0	Pyramidal (NFM)	Nrg1
0	1,284565657	0,741	0,152	0	Pyramidal (NFM)	Cntnap4
0	1,272878706	1	0,955	0	Pyramidal (NFM)	Ptprd
0	1,221725349	0,988	0,915	0	Pyramidal (NFM)	Pde4d
0	1,190661438	1	0,92	0	Pyramidal (NFM)	Arpp21
0	1,190340507	0,941	0,572	0	Pyramidal (NFM)	Fut9
0	1,144799866	0,957	0,702	0	Pyramidal (NFM)	A830018L16Rik
0	1,143629955	0,972	0,745	0	Pyramidal (NFM)	Ano3
0	1,115399835	0,774	0,332	0	Pyramidal (NFM)	Tmem178
0	1,111188617	0,86	0,324	0	Pyramidal (NFM)	Rasgef1b
0	1,102068648	1	0,972	0	Pyramidal (NFM)	Phactr1
0	1,093722295	0,933	0,546	0	Pyramidal (NFM)	Lrrtm3
0	1,085042761	0,975	0,857	0	Pyramidal (NFM)	Mmp16
0	1,071152872	0,981	0,774	0	Pyramidal (NFM)	Agbl4
0	1,061387328	0,933	0,699	0	Pyramidal (NFM)	Zfpm2
0	1,047756972	0,701	0,289	0	Pyramidal (NFM)	Pcsk5

0	1,04177462	0,695	0,309	0	Pyramidal (NFM)	Pdzrn4
0	1,036036725	0,679	0,153	0	Pyramidal (NFM)	Oprm1
0	1,025715458	0,997	0,934	0	Pyramidal (NFM)	Pbx1
0	1,021425325	0,916	0,527	0	Pyramidal (NFM)	Ncald
0	1,000807513	0,626	0,07	0	Pyramidal (NFM)	Rai14
0	0,980175251	0,993	0,904	0	Pyramidal (NFM)	Asic2
0	0,978630349	0,98	0,713	0	Pyramidal (NFM)	Khdrbs3
0	0,972898416	0,621	0,168	0	Pyramidal (NFM)	Plcxd3
0	0,971252621	0,942	0,642	0	Pyramidal (NFM)	Cntn4
0	0,958537833	0,991	0,792	0	Pyramidal (NFM)	Kcnh7
0	0,952812829	0,966	0,887	0	Pyramidal (NFM)	Celf4
0	0,936244354	1	0,989	0	Pyramidal (NFM)	Dlg2
0	0,92749681	0,973	0,819	0	Pyramidal (NFM)	Ext1
0	0,925861595	0,988	0,884	0	Pyramidal (NFM)	Rims2
0	0,925377349	1	0,967	0	Pyramidal (NFM)	Ahi1
0	0,913042236	1	0,989	0	Pyramidal (NFM)	Mdga2
0	0,912592158	0,812	0,444	0	Pyramidal (NFM)	Slc35f4
0	0,912575508	0,688	0,309	0	Pyramidal (NFM)	Tmem132d
0	0,878324568	0,608	0,144	0	Pyramidal (NFM)	Igsf21
0	0,87285609	0,951	0,861	0	Pyramidal (NFM)	Gm26871
0	0,863290564	0,994	0,92	0	Pyramidal (NFM)	Gria3
0	0,858032083	0,85	0,443	0	Pyramidal (NFM)	Camk4
0	0,857590592	0,782	0,311	0	Pyramidal (NFM)	Fnbp1l
0	0,853344093	0,96	0,767	0	Pyramidal (NFM)	Mctp1
0	0,850704713	0,915	0,697	0	Pyramidal (NFM)	Sh3gl2
0	0,850458087	1	0,978	0	Pyramidal (NFM)	Nav3
0	0,847311521	0,997	0,914	0	Pyramidal (NFM)	Snap25
0	0,844324533	0,734	0,27	0	Pyramidal (NFM)	Tshz3
0	0,839347367	0,956	0,759	0	Pyramidal (NFM)	Prkcb
0	0,838529095	0,546	0,102	0	Pyramidal (NFM)	Hs3st2
0	0,836611036	0,58	0,117	0	Pyramidal (NFM)	Ipcef1
0	0,825733141	1	0,982	0	Pyramidal (NFM)	Nrg3
0	0,823680744	0,958	0,668	0	Pyramidal (NFM)	Ctnna3
0	0,820703723	0,9	0,705	0	Pyramidal (NFM)	Nos1ap
0	0,806189354	0,952	0,743	0	Pyramidal (NFM)	Hecw2
0	0,805873897	0,967	0,834	0	Pyramidal (NFM)	5730522E02Rik
0	0,796815428	0,846	0,493	0	Pyramidal (NFM)	Gas7
0	0,795400986	0,819	0,377	0	Pyramidal (NFM)	Ldb2
0	0,793939757	0,854	0,406	0	Pyramidal (NFM)	Sv2b
0	0,772384838	0,923	0,788	0	Pyramidal (NFM)	Khdrbs2
0	0,764597062	0,929	0,82	0	Pyramidal (NFM)	Cacnb4
0	0,762026626	0,994	0,938	0	Pyramidal (NFM)	Prickle1
0	0,756266127	0,963	0,884	0	Pyramidal (NFM)	Samd12

0	0,751164177	0,999	0,968	0	Pyramidal (NFM)	Kcnma1
0	0,750812351	0,952	0,807	0	Pyramidal (NFM)	Slc4a10
0	0,750455486	0,598	0,166	0	Pyramidal (NFM)	Vxn
0	0,746672521	0,774	0,391	0	Pyramidal (NFM)	Flrt2
0	0,737934267	0,586	0,15	0	Pyramidal (NFM)	Atp2b4
0	0,731507882	0,89	0,562	0	Pyramidal (NFM)	Sox5
0	0,730679818	0,99	0,951	0	Pyramidal (NFM)	Tanc2
0	0,729015916	0,777	0,381	0	Pyramidal (NFM)	Esrrg
0	0,728375487	1	0,98	0	Pyramidal (NFM)	Fam155a
0	0,724948109	1	0,977	0	Pyramidal (NFM)	Syt1
0	0,722799179	0,792	0,417	0	Pyramidal (NFM)	Kcnip3
0	0,713112018	0,708	0,281	0	Pyramidal (NFM)	Mpped1
0	0,689988593	0,571	0,151	0	Pyramidal (NFM)	Adgra1
0	0,680941978	0,921	0,774	0	Pyramidal (NFM)	Cdh11
0	0,680227492	1	0,988	0	Pyramidal (NFM)	Adgrb3
0	0,67743675	1	0,987	0	Pyramidal (NFM)	Csmd1
0	0,676231698	0,968	0,895	0	Pyramidal (NFM)	Ptk2
0	0,676182454	0,797	0,455	0	Pyramidal (NFM)	Srrm4
0	0,674251646	0,615	0,251	0	Pyramidal (NFM)	Osbpl10
0	0,669198844	0,909	0,72	0	Pyramidal (NFM)	Tenm1
0	0,660763994	0,997	0,967	0	Pyramidal (NFM)	Rims1
0	0,66073275	1	0,981	0	Pyramidal (NFM)	Fgf14
0	0,660288944	0,921	0,575	0	Pyramidal (NFM)	Sgcz
0	0,653830907	0,792	0,509	0	Pyramidal (NFM)	Plxna2
0	0,653801122	0,966	0,895	0	Pyramidal (NFM)	Dock3
0	0,648689263	1	0,991	0	Pyramidal (NFM)	Nrxn1
0	0,647885507	0,919	0,712	0	Pyramidal (NFM)	Asap1
0	0,64623452	0,994	0,951	0	Pyramidal (NFM)	Myt1l
0	0,641206713	0,981	0,898	0	Pyramidal (NFM)	Pcsk2
0	0,627136701	0,872	0,592	0	Pyramidal (NFM)	Xylt1
0	0,622711651	0,652	0,288	0	Pyramidal (NFM)	Sel1l3
0	0,619883522	0,929	0,787	0	Pyramidal (NFM)	A230057D06Rik
0	0,613399356	0,945	0,838	0	Pyramidal (NFM)	Dync1i1
0	0,601637629	0,598	0,258	0	Pyramidal (NFM)	Galnt9
0	0,594470338	0,994	0,966	0	Pyramidal (NFM)	Strbp
0	0,588326956	0,632	0,293	0	Pyramidal (NFM)	B230209E15Rik
0	0,571123534	1	0,987	0	Pyramidal (NFM)	Pcdh9
0	0,570285162	0,624	0,291	0	Pyramidal (NFM)	Elavl4
0	0,533907835	0,91	0,555	0	Pyramidal (NFM)	Grm3
0	0,520366287	0,509	0,19	0	Pyramidal (NFM)	St6gal2
0	0,515368447	0,519	0,212	0	Pyramidal (NFM)	Etv5
1,39E-303	0,547889188	0,581	0,265	3,73E-299	Pyramidal (NFM)	Igsf9b
1,71E-302	0,592708801	0,793	0,486	4,60E-298	Pyramidal (NFM)	A330076H08Rik

2,70E-299	0,666428202	1	0,905	7,28E-295	Pyramidal (NFM)	Snhg11
2,02E-297	0,703046704	0,699	0,355	5,43E-293	Pyramidal (NFM)	Rgs6
2,26E-297	0,626837337	0,989	0,925	6,09E-293	Pyramidal (NFM)	Dmd
2,82E-295	0,539578556	0,67	0,344	7,59E-291	Pyramidal (NFM)	Rap1gap2
2,43E-291	0,501852908	0,631	0,299	6,54E-287	Pyramidal (NFM)	Rasgrp1
7,78E-291	0,614080424	0,996	0,879	2,10E-286	Pyramidal (NFM)	Slc8a1
4,51E-290	0,517503329	0,96	0,905	1,22E-285	Pyramidal (NFM)	Unc80
4,89E-289	0,527051153	0,984	0,931	1,32E-284	Pyramidal (NFM)	Fgf12
5,85E-287	0,633330238	0,81	0,548	1,58E-282	Pyramidal (NFM)	Syt16
1,47E-283	0,569362086	0,915	0,808	3,96E-279	Pyramidal (NFM)	Atrnl1
3,73E-281	0,58737423	0,997	0,931	1,01E-276	Pyramidal (NFM)	Ralyl
1,55E-277	0,672736268	0,662	0,355	4,17E-273	Pyramidal (NFM)	Brinp2
7,42E-273	0,580170928	0,662	0,342	2,00E-268	Pyramidal (NFM)	Npas2
1,06E-269	0,543441188	0,981	0,941	2,86E-265	Pyramidal (NFM)	Mapk10
1,69E-269	0,534902406	0,981	0,913	4,55E-265	Pyramidal (NFM)	Oxr1
9,02E-265	0,820265533	0,726	0,423	2,43E-260	Pyramidal (NFM)	Homer1
1,74E-264	0,572109676	0,72	0,416	4,69E-260	Pyramidal (NFM)	Efr3a
2,22E-264	0,559791885	0,946	0,88	5,98E-260	Pyramidal (NFM)	Nrg3os
2,12E-263	0,533185532	0,898	0,762	5,70E-259	Pyramidal (NFM)	Atp2b2
3,47E-259	0,512644731	0,694	0,378	9,34E-255	Pyramidal (NFM)	Pfkp
2,81E-258	0,689427477	0,839	0,669	7,58E-254	Pyramidal (NFM)	Caln1
3,44E-251	0,537696509	0,688	0,399	9,27E-247	Pyramidal (NFM)	Arhgap44
6,68E-251	0,552589943	0,509	0,23	1,80E-246	Pyramidal (NFM)	Olfm3
5,47E-250	0,520356859	0,547	0,257	1,47E-245	Pyramidal (NFM)	Gng12
6,95E-250	0,717879784	0,589	0,308	1,87E-245	Pyramidal (NFM)	Serpini1
8,17E-250	0,587234044	0,849	0,694	2,20E-245	Pyramidal (NFM)	Dtnb
1,02E-245	0,552167407	0,866	0,677	2,74E-241	Pyramidal (NFM)	Arhgap26
1,41E-244	0,526134254	0,94	0,866	3,81E-240	Pyramidal (NFM)	Gm20642
3,84E-244	0,539162943	0,998	0,952	1,04E-239	Pyramidal (NFM)	Negr1
4,67E-242	0,514807894	0,793	0,51	1,26E-237	Pyramidal (NFM)	Ablim1
5,62E-242	0,514535906	0,925	0,725	1,51E-237	Pyramidal (NFM)	Chrm3
6,71E-242	0,574960086	0,891	0,796	1,81E-237	Pyramidal (NFM)	Csmd2
2,03E-239	0,550489784	0,764	0,46	5,46E-235	Pyramidal (NFM)	Arap2
2,25E-239	0,535975614	0,665	0,364	6,06E-235	Pyramidal (NFM)	Fmn1
2,90E-238	0,674167541	0,865	0,662	7,80E-234	Pyramidal (NFM)	Astn2
6,97E-238	0,500168085	0,648	0,354	1,88E-233	Pyramidal (NFM)	11. Sep
6,07E-234	0,532885206	0,935	0,719	1,63E-229	Pyramidal (NFM)	Mir100hg
3,67E-231	0,523718506	0,709	0,422	9,90E-227	Pyramidal (NFM)	Plcl2
1,19E-228	0,582989317	0,834	0,577	3,22E-224	Pyramidal (NFM)	Pcdh15
2,10E-223	0,553337524	0,551	0,283	5,67E-219	Pyramidal (NFM)	Map3k5
3,65E-221	0,566401458	1	0,978	9,82E-217	Pyramidal (NFM)	Meg3
1,09E-217	0,526684329	0,531	0,258	2,94E-213	Pyramidal (NFM)	Satb2
1,27E-210	0,577725525	0,943	0,89	3,42E-206	Pyramidal (NFM)	Pde10a

3,43E-203	0,523045694	0,989	0,945	9,24E-199	Pyramidal (NFM)	Nrxn3
7,08E-199	0,590396449	1	0,979	1,91E-194	Pyramidal (NFM)	Rbfox1
1,69E-189	0,857684813	0,593	0,361	4,54E-185	Pyramidal (NFM)	Meis2
4,81E-172	0,610182537	0,797	0,666	1,30E-167	Pyramidal (NFM)	Smyd3
1,36E-171	0,570365368	0,769	0,615	3,65E-167	Pyramidal (NFM)	Me3
6,35E-163	0,923193049	0,782	0,646	1,71E-158	Pyramidal (NFM)	Mgat4c
4,18E-140	0,548576983	0,777	0,618	1,13E-135	Pyramidal (NFM)	Car10
4,10E-97	0,599147717	0,613	0,423	1,10E-92	Pyramidal (NFM)	Gm49678
2,04E-87	0,540311543	0,781	0,724	5,49E-83	Pyramidal (NFM)	Pcdh7

0	3,310532364	0,646	0,112	0	Pyramidal (NFV)	Ptgds
0	2,779201605	0,653	0,067	0	Pyramidal (NFV)	Slc7a11
0	2,696459381	0,911	0,035	0	Pyramidal (NFV)	Cped1
0	2,403801911	0,568	0,052	0	Pyramidal (NFV)	Ranbp3l
0	2,387897224	0,591	0,019	0	Pyramidal (NFV)	Slc6a20a
0	2,200712377	0,697	0,16	0	Pyramidal (NFV)	Atp1a2
0	2,140206012	0,679	0,151	0	Pyramidal (NFV)	Adam12
0	2,109810145	0,893	0,619	0	Pyramidal (NFV)	Alcam
0	1,960885493	0,728	0,389	0	Pyramidal (NFV)	Slc38a2
0	1,893175848	0,582	0,022	0	Pyramidal (NFV)	Bmp6
0	1,766691423	0,662	0,245	0	Pyramidal (NFV)	Fbxl7
0	1,734479942	0,768	0,15	0	Pyramidal (NFV)	9530026P05Rik
0	1,730345201	0,59	0,094	0	Pyramidal (NFV)	Foxp2
0	1,730207564	0,766	0,227	0	Pyramidal (NFV)	Bicc1
0	1,721367697	0,689	0,08	0	Pyramidal (NFV)	Sned1
0	1,692941703	0,575	0,036	0	Pyramidal (NFV)	Colec12
0	1,62486377	0,662	0,018	0	Pyramidal (NFV)	Eya2
0	1,478102694	0,5	0,009	0	Pyramidal (NFV)	Lama1
0	1,471588043	0,797	0,306	0	Pyramidal (NFV)	Rbms3
0	1,469042198	0,986	0,882	0	Pyramidal (NFV)	Foxp1
0	1,461950738	0,851	0,415	0	Pyramidal (NFV)	Gpc6
0	1,416997366	0,742	0,365	0	Pyramidal (NFV)	Ptn
0	1,385508535	0,598	0,071	0	Pyramidal (NFV)	Arhgap29
0	1,380812933	0,768	0,195	0	Pyramidal (NFV)	Lama2
0	1,341198212	0,6	0,05	0	Pyramidal (NFV)	Phldb2
0	1,302512821	0,747	0,261	0	Pyramidal (NFV)	Rbms1
0	1,290783378	0,669	0,136	0	Pyramidal (NFV)	Eya1
0	1,279318732	0,992	0,915	0	Pyramidal (NFV)	Rora
0	1,264156551	0,575	0,061	0	Pyramidal (NFV)	Gulp1
0	1,259494247	0,616	0,091	0	Pyramidal (NFV)	Nxn
0	1,251968868	0,501	0,068	0	Pyramidal (NFV)	Apod
0	1,245076009	0,674	0,227	0	Pyramidal (NFV)	Slc1a3
0	1,221515685	0,929	0,516	0	Pyramidal (NFV)	Gm42418

0	1,198576999	0,596	0,11	0	Pyramidal (NFV)	Ptpn13
0	1,171472748	0,681	0,165	0	Pyramidal (NFV)	Neat1
0	1,15149671	0,568	0,172	0	Pyramidal (NFV)	Pbx3
0	1,115292689	0,683	0,267	0	Pyramidal (NFV)	Tgfbr3
0	1,091939262	0,679	0,259	0	Pyramidal (NFV)	Zfhx4
0	1,091738308	0,511	0,094	0	Pyramidal (NFV)	Arhgap6
0	1,083168525	0,591	0,117	0	Pyramidal (NFV)	Svil
0	1,071412822	0,971	0,721	0	Pyramidal (NFV)	Mir100hg
0	1,043793762	0,602	0,103	0	Pyramidal (NFV)	Cpq
0	1,031399005	0,523	0,023	0	Pyramidal (NFV)	Zic1
0	1,00345921	0,68	0,225	0	Pyramidal (NFV)	Utrn
0	0,998713535	0,732	0,322	0	Pyramidal (NFV)	Cmss1
0	0,970841898	0,568	0,142	0	Pyramidal (NFV)	Rin2
0	0,93034089	0,997	0,936	0	Pyramidal (NFV)	Mir99ahg
0	0,926294901	0,543	0,115	0	Pyramidal (NFV)	Notch2
0	0,922243667	0,606	0,211	0	Pyramidal (NFV)	Cald1
0	0,892282794	0,587	0,161	0	Pyramidal (NFV)	Plpp3
0	0,867041189	0,558	0,126	0	Pyramidal (NFV)	Tcf7l2
0	0,838385879	0,566	0,195	0	Pyramidal (NFV)	Tmem164
0	0,795031124	0,535	0,17	0	Pyramidal (NFV)	Wls
0	0,668609713	0,604	0,177	0	Pyramidal (NFV)	Apoe
1,06E-296	0,805631109	0,573	0,199	2,87E-292	Pyramidal (NFV)	Pard3b
3,97E-294	0,679322457	0,522	0,166	1,07E-289	Pyramidal (NFV)	Epb41l2
2,56E-292	1,104186339	0,944	0,757	6,90E-288	Pyramidal (NFV)	Slc24a3
1,33E-275	0,978326174	0,543	0,207	3,58E-271	Pyramidal (NFV)	Hlf
1,79E-269	1,005667049	0,562	0,232	4,83E-265	Pyramidal (NFV)	Airn
9,40E-267	0,865425873	0,976	0,725	2,53E-262	Pyramidal (NFV)	Nfia
5,94E-258	0,963710949	0,779	0,488	1,60E-253	Pyramidal (NFV)	Gmds
1,80E-247	1,010761927	0,863	0,575	4,86E-243	Pyramidal (NFV)	Msi2
4,17E-220	1,001431229	0,825	0,576	1,12E-215	Pyramidal (NFV)	Naaladl2
1,05E-213	1,0411146	0,7	0,421	2,83E-209	Pyramidal (NFV)	Mpp6
1,32E-210	0,782652496	0,975	0,893	3,56E-206	Pyramidal (NFV)	Ddx5
2,50E-209	0,923772678	0,631	0,324	6,73E-205	Pyramidal (NFV)	Gab2
2,99E-209	0,780424378	0,835	0,63	8,05E-205	Pyramidal (NFV)	Nedd4
4,48E-207	2,692538476	0,919	0,68	1,21E-202	Pyramidal (NFV)	Trpm3
1,60E-205	0,801078329	0,685	0,375	4,32E-201	Pyramidal (NFV)	Cdk8
4,70E-197	0,884217675	0,685	0,377	1,27E-192	Pyramidal (NFV)	Dlc1
5,09E-185	0,735406235	0,872	0,71	1,37E-180	Pyramidal (NFV)	Nfat5
1,55E-184	0,76540421	0,547	0,253	4,16E-180	Pyramidal (NFV)	Ghr
9,40E-179	0,705238198	0,571	0,273	2,53E-174	Pyramidal (NFV)	Ttc28
4,29E-178	0,663016169	0,934	0,782	1,16E-173	Pyramidal (NFV)	Tcf12
1,93E-177	0,788838085	0,568	0,25	5,20E-173	Pyramidal (NFV)	Pdzrn3
3,39E-176	0,851382145	0,796	0,626	9,12E-172	Pyramidal (NFV)	Pcca

1,29E-175	0,550351608	0,999	0,981	3,48E-171	Pyramidal (NFV)	Son
2,05E-173	0,755162448	0,686	0,42	5,52E-169	Pyramidal (NFV)	Add3
3,38E-172	0,725178262	0,874	0,679	9,11E-168	Pyramidal (NFV)	Fmnl2
1,36E-170	0,791165835	0,579	0,289	3,67E-166	Pyramidal (NFV)	Ror1
5,81E-165	0,885810161	0,642	0,349	1,57E-160	Pyramidal (NFV)	Pde7b
2,95E-160	0,703275102	0,653	0,383	7,94E-156	Pyramidal (NFV)	Lpp
2,87E-156	0,865546069	0,824	0,64	7,73E-152	Pyramidal (NFV)	Wwox
8,43E-153	0,642400426	0,992	0,81	2,27E-148	Pyramidal (NFV)	Zbtb20
3,02E-152	0,782677245	0,738	0,493	8,14E-148	Pyramidal (NFV)	Pid1
4,26E-141	0,626163129	0,743	0,501	1,15E-136	Pyramidal (NFV)	Pard3
4,59E-138	0,741704551	0,593	0,371	1,24E-133	Pyramidal (NFV)	06. Mrz
6,93E-131	0,936556275	0,685	0,516	1,87E-126	Pyramidal (NFV)	Stk39
3,96E-130	0,642642806	0,895	0,767	1,07E-125	Pyramidal (NFV)	Diaph2
2,67E-129	0,594115706	0,658	0,417	7,19E-125	Pyramidal (NFV)	Fchsd2
2,48E-128	0,689709496	0,653	0,449	6,69E-124	Pyramidal (NFV)	Gnas
3,01E-127	0,870848498	0,598	0,373	8,11E-123	Pyramidal (NFV)	Tmtc1
3,16E-122	0,933603196	0,844	0,749	8,52E-118	Pyramidal (NFV)	Prkag2
9,31E-122	0,58603901	0,591	0,346	2,51E-117	Pyramidal (NFV)	Akap13
4,88E-118	0,739134363	0,664	0,456	1,32E-113	Pyramidal (NFV)	Ust
5,55E-118	0,535007958	0,565	0,306	1,50E-113	Pyramidal (NFV)	Stard13
5,96E-117	0,594646931	0,805	0,598	1,61E-112	Pyramidal (NFV)	Foxn3
1,01E-113	0,662383321	0,669	0,455	2,72E-109	Pyramidal (NFV)	Sh3d19
2,13E-106	0,503684123	0,85	0,726	5,73E-102	Pyramidal (NFV)	Ddx17
1,06E-100	0,539462409	0,561	0,344	2,84E-96	Pyramidal (NFV)	Phkb
5,79E-96	0,508419012	0,788	0,627	1,56E-91	Pyramidal (NFV)	Wnk1
1,19E-93	0,793649931	0,656	0,514	3,20E-89	Pyramidal (NFV)	Plxna2
3,98E-93	0,502829637	0,789	0,643	1,07E-88	Pyramidal (NFV)	Zeb1
6,02E-91	0,641419337	0,561	0,37	1,62E-86	Pyramidal (NFV)	Fhit
1,12E-89	0,519349797	0,77	0,645	3,02E-85	Pyramidal (NFV)	Afdn
6,59E-55	0,73453275	0,528	0,399	1,78E-50	Pyramidal (NFV)	Flrt2
4,95E-45	0,828719861	0,501	0,408	1,33E-40	Pyramidal (NFV)	Slc23a2
6,63E-13	1,366190007	0,756	0,811	1,79E-08	Pyramidal (NFV)	Slc4a10

0	2,473123867	0,998	0,657	0	Pyramidal (CA1-D)	Galntl6
0	1,949675755	1	0,869	0	Pyramidal (CA1-D)	Cntnap2
0	1,946234305	1	0,925	0	Pyramidal (CA1-D)	Epha6
0	1,811665772	0,685	0,124	0	Pyramidal (CA1-D)	4921539H07Rik
0	1,801131286	0,995	0,815	0	Pyramidal (CA1-D)	Hs6st3
0	1,690497176	1	0,866	0	Pyramidal (CA1-D)	Cacnb2
0	1,607581311	0,999	0,852	0	Pyramidal (CA1-D)	Atp2b1
0	1,537174942	0,983	0,614	0	Pyramidal (CA1-D)	Iqgap2
0	1,502061564	0,834	0,138	0	Pyramidal (CA1-D)	Gm10754
0	1,4692644	0,915	0,193	0	Pyramidal (CA1-D)	Gm2164

0	1,468657514	0,995	0,804	0	Pyramidal (CA1-D)	Ryr3
0	1,459012245	0,997	0,826	0	Pyramidal (CA1-D)	Tafa1
0	1,447038803	0,884	0,321	0	Pyramidal (CA1-D)	Man1a
0	1,41456963	0,999	0,809	0	Pyramidal (CA1-D)	Pex5l
0	1,369736043	0,937	0,704	0	Pyramidal (CA1-D)	Chrm3
0	1,353442481	0,994	0,855	0	Pyramidal (CA1-D)	Arl15
0	1,330738966	1	0,952	0	Pyramidal (CA1-D)	Grin2a
0	1,293386634	0,883	0,315	0	Pyramidal (CA1-D)	Cntnap5c
0	1,272831546	0,996	0,891	0	Pyramidal (CA1-D)	Ppm1e
0	1,258067761	0,983	0,773	0	Pyramidal (CA1-D)	Kcnh7
0	1,251709303	0,886	0,292	0	Pyramidal (CA1-D)	Nell1
0	1,233367847	1	0,97	0	Pyramidal (CA1-D)	Kcnd2
0	1,221147413	0,999	0,917	0	Pyramidal (CA1-D)	Dmd
0	1,201853646	1	0,928	0	Pyramidal (CA1-D)	Gria1
0	1,200086675	0,839	0,243	0	Pyramidal (CA1-D)	Ndst3
0	1,157711924	0,999	0,867	0	Pyramidal (CA1-D)	Slc8a1
0	1,128903404	0,946	0,749	0	Pyramidal (CA1-D)	Kcnab1
0	1,103002821	1	0,971	0	Pyramidal (CA1-D)	Grin2b
0	1,094326177	0,979	0,737	0	Pyramidal (CA1-D)	Prkcb
0	1,091266427	0,897	0,4	0	Pyramidal (CA1-D)	Runx2
0	1,084398977	0,678	0,097	0	Pyramidal (CA1-D)	Gm2115
0	1,077946328	0,81	0,222	0	Pyramidal (CA1-D)	Cpne8
0	1,077290068	0,821	0,303	0	Pyramidal (CA1-D)	Arhgap12
0	1,070619081	0,911	0,36	0	Pyramidal (CA1-D)	Cadps2
0	1,068199173	0,997	0,892	0	Pyramidal (CA1-D)	Cnksr2
0	1,057447774	0,975	0,762	0	Pyramidal (CA1-D)	Fam189a1
0	1,045181625	0,795	0,262	0	Pyramidal (CA1-D)	Tbc1d1
0	1,043418783	0,993	0,885	0	Pyramidal (CA1-D)	Msra
0	1,040617658	0,985	0,839	0	Pyramidal (CA1-D)	Brd9
0	1,033777855	0,975	0,64	0	Pyramidal (CA1-D)	Hcn1
0	1,025234329	0,987	0,819	0	Pyramidal (CA1-D)	St6galnac5
0	1,021997896	0,776	0,231	0	Pyramidal (CA1-D)	Ror1
0	1,015113972	0,868	0,333	0	Pyramidal (CA1-D)	4930419G24Rik
0	1,004266959	0,967	0,733	0	Pyramidal (CA1-D)	Slc24a3
0	0,996464971	0,767	0,213	0	Pyramidal (CA1-D)	Sema3e
0	0,983511269	0,687	0,232	0	Pyramidal (CA1-D)	Cpne7
0	0,977433412	0,895	0,433	0	Pyramidal (CA1-D)	Sorcs3
0	0,966783086	0,998	0,945	0	Pyramidal (CA1-D)	Hivep2
0	0,959075761	0,971	0,687	0	Pyramidal (CA1-D)	Khdrbs3
0	0,952032918	0,852	0,356	0	Pyramidal (CA1-D)	Sh3rf1
0	0,938255165	0,984	0,818	0	Pyramidal (CA1-D)	5730522E02Rik
0	0,935128871	0,866	0,372	0	Pyramidal (CA1-D)	Gm49678
0	0,934063762	0,97	0,715	0	Pyramidal (CA1-D)	Shisa6

0	0,926139993	0,999	0,95	0	Pyramidal (CA1-D)	Cttnbp2
0	0,925364948	1	0,965	0	Pyramidal (CA1-D)	Ppp3ca
0	0,91869777	0,974	0,678	0	Pyramidal (CA1-D)	Pde1a
0	0,91752836	0,999	0,936	0	Pyramidal (CA1-D)	Camk2a
0	0,916088699	0,987	0,822	0	Pyramidal (CA1-D)	Nr3c2
0	0,914746437	1	0,977	0	Pyramidal (CA1-D)	Rbfox1
0	0,910204122	0,845	0,366	0	Pyramidal (CA1-D)	Trpc4
0	0,905768632	0,57	0,212	0	Pyramidal (CA1-D)	Ndst4
0	0,90390507	0,825	0,332	0	Pyramidal (CA1-D)	Prkcg
0	0,888673678	0,973	0,794	0	Pyramidal (CA1-D)	Pigk
0	0,878769201	0,984	0,827	0	Pyramidal (CA1-D)	Galnt17
0	0,871897481	0,821	0,357	0	Pyramidal (CA1-D)	Asap2
0	0,868449995	0,94	0,651	0	Pyramidal (CA1-D)	Kcnn2
0	0,857724851	0,643	0,191	0	Pyramidal (CA1-D)	Gm30382
0	0,85633224	1	0,97	0	Pyramidal (CA1-D)	Grm5
0	0,855826367	0,999	0,895	0	Pyramidal (CA1-D)	Slit3
0	0,847724956	0,998	0,924	0	Pyramidal (CA1-D)	Ralyl
0	0,839599852	0,99	0,805	0	Pyramidal (CA1-D)	Gm20754
0	0,839567238	0,712	0,2	0	Pyramidal (CA1-D)	Gm10848
0	0,839209789	0,757	0,283	0	Pyramidal (CA1-D)	Gm13269
0	0,833820912	0,822	0,397	0	Pyramidal (CA1-D)	Dgkz
0	0,829612462	1	0,986	0	Pyramidal (CA1-D)	Adgrb3
0	0,827628349	1	0,97	0	Pyramidal (CA1-D)	Lrrc7
0	0,825233021	1	0,958	0	Pyramidal (CA1-D)	Adgrl3
0	0,822978462	1	0,956	0	Pyramidal (CA1-D)	Kcnip4
0	0,820336315	1	0,978	0	Pyramidal (CA1-D)	Celf2
0	0,809758015	0,976	0,891	0	Pyramidal (CA1-D)	Eml6
0	0,804352433	0,823	0,364	0	Pyramidal (CA1-D)	Itga8
0	0,80216096	0,654	0,184	0	Pyramidal (CA1-D)	Tmem200a
0	0,788244456	0,979	0,855	0	Pyramidal (CA1-D)	Egfem1
0	0,788077698	1	0,987	0	Pyramidal (CA1-D)	Dlgap1
0	0,782475854	0,96	0,752	0	Pyramidal (CA1-D)	Sphkap
0	0,781436073	0,935	0,68	0	Pyramidal (CA1-D)	Nos1ap
0	0,77683922	0,996	0,94	0	Pyramidal (CA1-D)	Nrxn3
0	0,775627931	0,691	0,29	0	Pyramidal (CA1-D)	Lmo3
0	0,768781562	0,72	0,255	0	Pyramidal (CA1-D)	Rasgrp1
0	0,766178807	1	0,951	0	Pyramidal (CA1-D)	Dab1
0	0,764037373	0,939	0,726	0	Pyramidal (CA1-D)	Prkag2
0	0,763833023	0,863	0,462	0	Pyramidal (CA1-D)	Wipf3
0	0,76303147	0,794	0,338	0	Pyramidal (CA1-D)	Bcl11b
0	0,762361772	0,844	0,553	0	Pyramidal (CA1-D)	Zfp804a
0	0,758832086	0,963	0,805	0	Pyramidal (CA1-D)	Ext1
0	0,754586564	0,65	0,226	0	Pyramidal (CA1-D)	Hunk

0	0,754537854	0,673	0,206	0	Pyramidal (CA1-D)	Ccdc88c
0	0,754191082	0,979	0,907	0	Pyramidal (CA1-D)	01. Mrz
0	0,742058124	1	0,987	0	Pyramidal (CA1-D)	Anks1b
0	0,735839751	0,996	0,955	0	Pyramidal (CA1-D)	Large1
0	0,73411053	0,998	0,927	0	Pyramidal (CA1-D)	Dock4
0	0,734086664	0,665	0,257	0	Pyramidal (CA1-D)	Gm44257
0	0,732449846	0,715	0,275	0	Pyramidal (CA1-D)	Ppp4r4
0	0,728696887	0,84	0,428	0	Pyramidal (CA1-D)	Runx1t1
0	0,724149248	0,987	0,895	0	Pyramidal (CA1-D)	Dok6
0	0,720587062	0,955	0,848	0	Pyramidal (CA1-D)	Ptprg
0	0,719849583	1	0,983	0	Pyramidal (CA1-D)	Gria2
0	0,715552236	0,799	0,383	0	Pyramidal (CA1-D)	Homer1
0	0,715245243	0,677	0,212	0	Pyramidal (CA1-D)	Pkp2
0	0,715169694	0,997	0,882	0	Pyramidal (CA1-D)	Zeb2
0	0,714527309	0,703	0,239	0	Pyramidal (CA1-D)	Mpped1
0	0,71449977	0,645	0,204	0	Pyramidal (CA1-D)	Tspan13
0	0,714424399	1	0,982	0	Pyramidal (CA1-D)	Opcml
0	0,713347507	0,809	0,434	0	Pyramidal (CA1-D)	Trpc5
0	0,713223587	0,971	0,84	0	Pyramidal (CA1-D)	Rasgrf1
0	0,706058795	0,631	0,215	0	Pyramidal (CA1-D)	Zdhhc2
0	0,701793085	0,892	0,615	0	Pyramidal (CA1-D)	Adcy9
0	0,701017763	0,975	0,88	0	Pyramidal (CA1-D)	Pde10a
0	0,700859171	0,676	0,258	0	Pyramidal (CA1-D)	Chrd
0	0,694878258	0,993	0,93	0	Pyramidal (CA1-D)	Epha5
0	0,693691007	0,814	0,425	0	Pyramidal (CA1-D)	Gm44151
0	0,691785469	0,766	0,35	0	Pyramidal (CA1-D)	Zfp462
0	0,689521731	0,998	0,949	0	Pyramidal (CA1-D)	Gabrb3
0	0,689359791	0,657	0,226	0	Pyramidal (CA1-D)	Itpka
0	0,68701803	0,631	0,197	0	Pyramidal (CA1-D)	Ociad2
0	0,686065049	0,732	0,346	0	Pyramidal (CA1-D)	A330015K06Rik
0	0,685661559	0,92	0,707	0	Pyramidal (CA1-D)	Itpr1
0	0,685180393	1	0,972	0	Pyramidal (CA1-D)	Tenm2
0	0,683246251	0,981	0,832	0	Pyramidal (CA1-D)	Nell2
0	0,670583959	1	0,896	0	Pyramidal (CA1-D)	Snhg11
0	0,667740839	0,608	0,199	0	Pyramidal (CA1-D)	Pantr1
0	0,666884484	0,973	0,876	0	Pyramidal (CA1-D)	Exoc6b
0	0,666695029	0,802	0,42	0	Pyramidal (CA1-D)	Lmo7
0	0,66577606	0,623	0,201	0	Pyramidal (CA1-D)	Stum
0	0,664379502	0,816	0,435	0	Pyramidal (CA1-D)	Sipa1l3
0	0,662035279	0,587	0,178	0	Pyramidal (CA1-D)	Matn2
0	0,661811661	0,926	0,762	0	Pyramidal (CA1-D)	Aff3
0	0,661706312	0,657	0,253	0	Pyramidal (CA1-D)	Scn3b
0	0,656039677	0,849	0,535	0	Pyramidal (CA1-D)	Wasf1

0	0,655529704	0,639	0,222	0	Pyramidal (CA1-D)	Gm37459
0	0,653259473	0,956	0,792	0	Pyramidal (CA1-D)	Dock9
0	0,648354728	1	0,967	0	Pyramidal (CA1-D)	Kalrn
0	0,647184955	0,805	0,412	0	Pyramidal (CA1-D)	4930509J09Rik
0	0,646430631	1	0,969	0	Pyramidal (CA1-D)	Phactr1
0	0,645593266	0,655	0,259	0	Pyramidal (CA1-D)	Dnah9
0	0,640658725	0,968	0,849	0	Pyramidal (CA1-D)	Mical2
0	0,640361723	0,913	0,727	0	Pyramidal (CA1-D)	Epha7
0	0,635838384	0,752	0,373	0	Pyramidal (CA1-D)	Camkv
0	0,635327407	0,76	0,41	0	Pyramidal (CA1-D)	Cdc40
0	0,634400744	0,614	0,206	0	Pyramidal (CA1-D)	Thsd4
0	0,634295927	0,951	0,832	0	Pyramidal (CA1-D)	Sipa1l1
0	0,6341431	1	0,985	0	Pyramidal (CA1-D)	Tcf4
0	0,632953065	0,927	0,758	0	Pyramidal (CA1-D)	Cdh11
0	0,632299568	0,86	0,605	0	Pyramidal (CA1-D)	Reps2
0	0,630863084	0,601	0,206	0	Pyramidal (CA1-D)	Plk2
0	0,629391135	1	0,976	0	Pyramidal (CA1-D)	Meg3
0	0,628603381	0,787	0,376	0	Pyramidal (CA1-D)	Rab3c
0	0,626783608	0,989	0,913	0	Pyramidal (CA1-D)	Gria3
0	0,618282626	0,907	0,74	0	Pyramidal (CA1-D)	Osbpl6
0	0,616781006	0,714	0,332	0	Pyramidal (CA1-D)	Cacng8
0	0,615184879	0,505	0,128	0	Pyramidal (CA1-D)	AC163685.1
0	0,613981911	1	0,978	0	Pyramidal (CA1-D)	Fam155a
0	0,611853501	0,994	0,946	0	Pyramidal (CA1-D)	Myt1l
0	0,610566764	0,771	0,41	0	Pyramidal (CA1-D)	Sorl1
0	0,607193533	0,642	0,241	0	Pyramidal (CA1-D)	Dkk3
0	0,606199163	0,988	0,922	0	Pyramidal (CA1-D)	Ptprn2
0	0,605586865	0,989	0,891	0	Pyramidal (CA1-D)	Cacna1e
0	0,599467046	0,775	0,441	0	Pyramidal (CA1-D)	Snd1
0	0,599241251	0,958	0,795	0	Pyramidal (CA1-D)	Tenm4
0	0,596677492	0,607	0,223	0	Pyramidal (CA1-D)	Galnt9
0	0,595852947	0,537	0,15	0	Pyramidal (CA1-D)	Neurod6
0	0,595788194	0,919	0,736	0	Pyramidal (CA1-D)	Dgkg
0	0,594885073	0,762	0,397	0	Pyramidal (CA1-D)	Susd6
0	0,594817945	0,993	0,941	0	Pyramidal (CA1-D)	R3hdm1
0	0,592600979	0,618	0,248	0	Pyramidal (CA1-D)	Adgrl2
0	0,590711131	1	0,976	0	Pyramidal (CA1-D)	Nav3
0	0,588187101	0,633	0,26	0	Pyramidal (CA1-D)	Htr1f
0	0,587592834	0,673	0,257	0	Pyramidal (CA1-D)	Raver2
0	0,583535463	0,828	0,554	0	Pyramidal (CA1-D)	2010300C02Rik
0	0,583454024	0,907	0,776	0	Pyramidal (CA1-D)	Khdrbs2
0	0,582467137	0,726	0,337	0	Pyramidal (CA1-D)	Cpne6
0	0,581142996	0,501	0,119	0	Pyramidal (CA1-D)	Ptpru

0	0,576243498	0,728	0,378	0	Pyramidal (CA1-D)	Ripor2
0	0,575963827	0,755	0,4	0	Pyramidal (CA1-D)	Shank1
0	0,573421101	0,969	0,856	0	Pyramidal (CA1-D)	Trim2
0	0,571899225	0,908	0,72	0	Pyramidal (CA1-D)	Bcl11a
0	0,571464809	0,903	0,708	0	Pyramidal (CA1-D)	Arhgap39
0	0,571077586	0,989	0,936	0	Pyramidal (CA1-D)	Mapk10
0	0,567947693	0,643	0,278	0	Pyramidal (CA1-D)	Samd5
0	0,560947733	0,71	0,358	0	Pyramidal (CA1-D)	Ksr1
0	0,557569502	1	0,987	0	Pyramidal (CA1-D)	Dlg2
0	0,557216509	1	0,96	0	Pyramidal (CA1-D)	Dlgap2
0	0,55660034	0,701	0,363	0	Pyramidal (CA1-D)	Arpc2
0	0,556277725	0,696	0,361	0	Pyramidal (CA1-D)	Sptbn2
0	0,553554338	0,979	0,862	0	Pyramidal (CA1-D)	Cacna2d3
0	0,553123438	1	0,987	0	Pyramidal (CA1-D)	Lsamp
0	0,552737275	0,711	0,372	0	Pyramidal (CA1-D)	Kctd1
0	0,552533991	0,787	0,526	0	Pyramidal (CA1-D)	Ap2b1
0	0,549748788	0,892	0,741	0	Pyramidal (CA1-D)	Mllt3
0	0,544611251	0,975	0,934	0	Pyramidal (CA1-D)	Kcnq3
0	0,544443437	0,583	0,231	0	Pyramidal (CA1-D)	Homer2
0	0,543721047	0,864	0,659	0	Pyramidal (CA1-D)	Csnk1a1
0	0,543503342	0,656	0,306	0	Pyramidal (CA1-D)	Wscd2
0	0,540346149	0,932	0,736	0	Pyramidal (CA1-D)	9530059O14Rik
0	0,539324196	0,804	0,519	0	Pyramidal (CA1-D)	Cdk17
0	0,537829503	0,916	0,694	0	Pyramidal (CA1-D)	Epha4
0	0,537774417	0,967	0,873	0	Pyramidal (CA1-D)	Foxp1
0	0,536492731	0,678	0,331	0	Pyramidal (CA1-D)	Lrrn2
0	0,536315771	0,948	0,843	0	Pyramidal (CA1-D)	Acap2
0	0,535890863	0,932	0,847	0	Pyramidal (CA1-D)	Tspan5
0	0,534507063	0,653	0,29	0	Pyramidal (CA1-D)	Gm12296
0	0,533992807	0,84	0,577	0	Pyramidal (CA1-D)	Calm2
0	0,530047517	0,93	0,77	0	Pyramidal (CA1-D)	Miat
0	0,525300098	0,653	0,332	0	Pyramidal (CA1-D)	Actr3b
0	0,52493041	0,992	0,957	0	Pyramidal (CA1-D)	Xkr4
0	0,522325732	0,694	0,391	0	Pyramidal (CA1-D)	Tfdp2
0	0,516284181	0,866	0,59	0	Pyramidal (CA1-D)	Pip5k1b
0	0,513983992	0,687	0,357	0	Pyramidal (CA1-D)	Celf5
0	0,513026796	0,767	0,431	0	Pyramidal (CA1-D)	Slc17a7
0	0,510704938	0,81	0,576	0	Pyramidal (CA1-D)	Chl1
0	0,500974392	0,659	0,32	0	Pyramidal (CA1-D)	Ankrd33b
0	0,500193087	0,928	0,804	0	Pyramidal (CA1-D)	Adcy2
5,15E-288	0,760595371	0,752	0,686	1,39E-283	Pyramidal (CA1-D)	Tenm3
0	1,985637788	0,978	0,709	0	Pyramidal (CA1-V)	Nrg1
0	1,983733719	0,946	0,582	0	Pyramidal (CA1-V)	Dpp10

0	1,815361695	0,561	0,191	0	Pyramidal (CA1-V)	Tshz2
0	1,669679296	0,906	0,524	0	Pyramidal (CA1-V)	Sgcz
0	1,411394872	0,513	0,236	0	Pyramidal (CA1-V)	Il1rapl2
0	1,383967735	0,861	0,578	0	Pyramidal (CA1-V)	Car10
0	1,383066102	0,819	0,343	0	Pyramidal (CA1-V)	Unc5d
0	1,349602806	1	0,948	0	Pyramidal (CA1-V)	Ptprd
0	1,300372276	0,681	0,176	0	Pyramidal (CA1-V)	Pdzrn3
0	1,262545332	0,892	0,526	0	Pyramidal (CA1-V)	Pcdh15
0	1,232706294	0,989	0,893	0	Pyramidal (CA1-V)	Lrrc4c
0	1,219832334	0,902	0,623	0	Pyramidal (CA1-V)	Mef2c
0	1,208638223	0,999	0,908	0	Pyramidal (CA1-V)	Arpp21
0	1,203949748	0,958	0,844	0	Pyramidal (CA1-V)	Lingo2
0	1,19449634	0,71	0,299	0	Pyramidal (CA1-V)	Rgs6
0	1,168001764	0,903	0,704	0	Pyramidal (CA1-V)	Cntn5
0	1,166042736	0,709	0,248	0	Pyramidal (CA1-V)	Prr16
0	1,076206401	0,816	0,619	0	Pyramidal (CA1-V)	Cntn4
0	1,04033773	0,99	0,891	0	Pyramidal (CA1-V)	Asic2
0	1,019050806	0,77	0,258	0	Pyramidal (CA1-V)	Rasgef1b
0	0,987849173	0,991	0,939	0	Pyramidal (CA1-V)	R3hdm1
0	0,987322556	0,999	0,971	0	Pyramidal (CA1-V)	Tenm2
0	0,967856959	0,689	0,187	0	Pyramidal (CA1-V)	Satb2
0	0,951494355	0,985	0,884	0	Pyramidal (CA1-V)	Pcsk2
0	0,945571294	0,936	0,657	0	Pyramidal (CA1-V)	Gria4
0	0,929048126	0,823	0,463	0	Pyramidal (CA1-V)	Chsy3
0	0,927730993	0,683	0,225	0	Pyramidal (CA1-V)	Grm8
0	0,925028668	0,784	0,315	0	Pyramidal (CA1-V)	Ldb2
0	0,923531864	0,59	0,192	0	Pyramidal (CA1-V)	Efna5
0	0,895716376	0,691	0,27	0	Pyramidal (CA1-V)	Nwd2
0	0,879577317	0,589	0,252	0	Pyramidal (CA1-V)	Tox
0	0,878102775	0,569	0,176	0	Pyramidal (CA1-V)	Gm15398
0	0,858418816	0,675	0,187	0	Pyramidal (CA1-V)	Cobl
0	0,849926349	0,999	0,967	0	Pyramidal (CA1-V)	Phactr1
0	0,84426227	0,967	0,785	0	Pyramidal (CA1-V)	Tenm4
0	0,839928256	0,638	0,216	0	Pyramidal (CA1-V)	Tshz3
0	0,837980769	0,616	0,386	0	Pyramidal (CA1-V)	Gpc6
0	0,831817608	0,928	0,766	0	Pyramidal (CA1-V)	Khdrbs2
0	0,82205559	0,944	0,815	0	Pyramidal (CA1-V)	Hs6st3
0	0,815860359	0,908	0,715	0	Pyramidal (CA1-V)	Etl4
0	0,814779333	0,933	0,77	0	Pyramidal (CA1-V)	Kcnq5
0	0,811819041	0,979	0,924	0	Pyramidal (CA1-V)	Ralyl
0	0,811392958	0,949	0,8	0	Pyramidal (CA1-V)	Cacnb4
0	0,810409409	0,883	0,706	0	Pyramidal (CA1-V)	Cntn3
0	0,808709476	0,964	0,899	0	Pyramidal (CA1-V)	Kcnb2

0	0,807383228	0,591	0,164	0	Pyramidal (CA1-V)	Fhod3
0	0,805842926	0,71	0,251	0	Pyramidal (CA1-V)	Fnbp1l
0	0,793361515	0,863	0,737	0	Pyramidal (CA1-V)	Cdh12
0	0,778964094	0,999	0,963	0	Pyramidal (CA1-V)	Kcnma1
0	0,773059709	1	0,975	0	Pyramidal (CA1-V)	Meg3
0	0,772142719	0,743	0,412	0	Pyramidal (CA1-V)	Fstl4
0	0,767594625	0,614	0,274	0	Pyramidal (CA1-V)	Camk2d
0	0,766080736	0,695	0,317	0	Pyramidal (CA1-V)	Mlip
0	0,764452899	1	0,962	0	Pyramidal (CA1-V)	Grm7
0	0,756771045	0,908	0,683	0	Pyramidal (CA1-V)	Hecw1
0	0,754216826	1	0,891	0	Pyramidal (CA1-V)	Snhg11
0	0,751781161	0,94	0,749	0	Pyramidal (CA1-V)	Agbl4
0	0,751760491	0,857	0,532	0	Pyramidal (CA1-V)	A230006K03Rik
0	0,750760182	0,894	0,684	0	Pyramidal (CA1-V)	Asap1
0	0,750333141	0,951	0,769	0	Pyramidal (CA1-V)	Kcnh7
0	0,73177503	0,712	0,378	0	Pyramidal (CA1-V)	Homer1
0	0,727344646	1	0,985	0	Pyramidal (CA1-V)	Csmd1
0	0,720267686	0,99	0,921	0	Pyramidal (CA1-V)	Fgf12
0	0,709838689	0,946	0,918	0	Pyramidal (CA1-V)	Kctd16
0	0,705545104	0,991	0,903	0	Pyramidal (CA1-V)	Snap25
0	0,705204928	0,869	0,699	0	Pyramidal (CA1-V)	Pcdh7
0	0,698313349	0,94	0,893	0	Pyramidal (CA1-V)	Sorbs2
0	0,6975107	0,537	0,172	0	Pyramidal (CA1-V)	Necab1
0	0,686271718	0,643	0,424	0	Pyramidal (CA1-V)	Brinp3
0	0,682980986	1	0,977	0	Pyramidal (CA1-V)	Celf2
0	0,675966567	0,995	0,954	0	Pyramidal (CA1-V)	Kcnip4
0	0,666383116	1	0,973	0	Pyramidal (CA1-V)	Syt1
0	0,649048975	0,546	0,277	0	Pyramidal (CA1-V)	Pdzrn4
0	0,632751758	0,861	0,635	0	Pyramidal (CA1-V)	D430041D05Rik
0	0,63172577	0,798	0,512	0	Pyramidal (CA1-V)	Vsnl1
0	0,629582526	0,999	0,979	0	Pyramidal (CA1-V)	Fgf14
0	0,62367953	0,739	0,401	0	Pyramidal (CA1-V)	Camk4
0	0,612128418	0,812	0,539	0	Pyramidal (CA1-V)	Fut9
0	0,607741321	0,641	0,296	0	Pyramidal (CA1-V)	Npas2
0	0,604788857	0,698	0,34	0	Pyramidal (CA1-V)	Nyap2
0	0,603385116	0,961	0,873	0	Pyramidal (CA1-V)	Rims2
0	0,602686238	0,924	0,821	0	Pyramidal (CA1-V)	5730522E02Rik
0	0,602369589	0,98	0,894	0	Pyramidal (CA1-V)	Cacna2d1
0	0,602059066	0,664	0,452	0	Pyramidal (CA1-V)	Sorcs3
0	0,598829286	0,918	0,847	0	Pyramidal (CA1-V)	Gpr158
0	0,590720798	0,638	0,318	0	Pyramidal (CA1-V)	Adcy8
0	0,57801582	0,652	0,307	0	Pyramidal (CA1-V)	11. Sep
0	0,574694349	0,537	0,203	0	Pyramidal (CA1-V)	Satb1

0	0,574617948	0,988	0,96	0	Pyramidal (CA1-V)	Il1rapl1
0	0,571498209	0,765	0,518	0	Pyramidal (CA1-V)	Lrrtm3
0	0,569731495	0,966	0,884	0	Pyramidal (CA1-V)	Dock3
0	0,568036187	0,728	0,36	0	Pyramidal (CA1-V)	Sv2b
0	0,567263246	1	0,979	0	Pyramidal (CA1-V)	Nrg3
0	0,565765064	0,551	0,276	0	Pyramidal (CA1-V)	Tmem132d
0	0,562267824	0,975	0,937	0	Pyramidal (CA1-V)	Rgs7
0	0,56172553	0,817	0,658	0	Pyramidal (CA1-V)	Nlk
0	0,559759294	0,913	0,717	0	Pyramidal (CA1-V)	Hecw2
0	0,556109272	0,975	0,834	0	Pyramidal (CA1-V)	Rian
0	0,556098444	0,946	0,881	0	Pyramidal (CA1-V)	Pde10a
0	0,550489359	0,728	0,41	0	Pyramidal (CA1-V)	Phf24
0	0,549917192	0,999	0,975	0	Pyramidal (CA1-V)	Nav3
0	0,541766813	0,953	0,873	0	Pyramidal (CA1-V)	Samd12
0	0,536840264	0,912	0,868	0	Pyramidal (CA1-V)	Cacna2d3
0	0,533267615	0,551	0,333	0	Pyramidal (CA1-V)	Meis2
0	0,529989864	0,606	0,224	0	Pyramidal (CA1-V)	Cit
0	0,527908724	0,909	0,807	0	Pyramidal (CA1-V)	Ext1
0	0,522232996	0,549	0,222	0	Pyramidal (CA1-V)	Igsf9b
0	0,517118702	0,572	0,275	0	Pyramidal (CA1-V)	Cacng3
0	0,516478812	0,796	0,596	0	Pyramidal (CA1-V)	Galnt13
0	0,515734723	0,632	0,308	0	Pyramidal (CA1-V)	Ankrd33b
0	0,504019271	0,591	0,275	0	Pyramidal (CA1-V)	Garnl3
0	0,500120902	0,875	0,745	0	Pyramidal (CA1-V)	Atp2b2
4,43E-262	0,918311349	0,675	0,662	1,19E-257	Pyramidal (CA1-V)	Sgcd
0	2,017019125	0,952	0,423	0	Pyramidal (CA2)	Ntng1
0	1,581295007	0,953	0,664	0	Pyramidal (CA2)	Hcn1
0	1,555879622	0,822	0,285	0	Pyramidal (CA2)	Ndst3
0	1,504615188	0,9	0,266	0	Pyramidal (CA2)	Grm8
0	1,500359657	0,893	0,567	0	Pyramidal (CA2)	Sgcz
0	1,483248245	0,822	0,366	0	Pyramidal (CA2)	Ldb2
0	1,478113736	0,92	0,624	0	Pyramidal (CA2)	Dpp10
0	1,460577898	0,603	0,235	0	Pyramidal (CA2)	Ndst4
0	1,339656103	0,779	0,405	0	Pyramidal (CA2)	Cadps2
0	1,320416538	0,994	0,768	0	Pyramidal (CA2)	Agbl4
0	1,274460766	0,685	0,229	0	Pyramidal (CA2)	Etv1
0	1,269242091	0,742	0,252	0	Pyramidal (CA2)	Gm2164
0	1,26740653	0,654	0,228	0	Pyramidal (CA2)	Tshz2
0	1,263810105	0,688	0,23	0	Pyramidal (CA2)	Fras1
0	1,261664638	0,983	0,787	0	Pyramidal (CA2)	Kcnh7
0	1,19974074	1	0,977	0	Pyramidal (CA2)	Meg3
0	1,170823513	0,699	0,284	0	Pyramidal (CA2)	Tox
0	1,168087475	0,568	0,077	0	Pyramidal (CA2)	Rxfp1

0	1,147603478	0,717	0,445	0	Pyramidal (CA2)	Brinp3
0	1,146010038	0,941	0,551	0	Pyramidal (CA2)	Sox5
0	1,13878385	0,935	0,751	0	Pyramidal (CA2)	Cntnap5a
0	1,127109888	0,923	0,654	0	Pyramidal (CA2)	Astn2
0	1,101609199	0,884	0,638	0	Pyramidal (CA2)	Cntn4
0	1,052416175	0,892	0,684	0	Pyramidal (CA2)	Tenm3
0	1,025232728	0,994	0,879	0	Pyramidal (CA2)	Lrrtm4
0	1,018612519	0,658	0,221	0	Pyramidal (CA2)	Gm30382
0	1,01164438	1	0,954	0	Pyramidal (CA2)	Ptprd
0	1,011089088	0,994	0,959	0	Pyramidal (CA2)	Kcnip4
0	0,99846195	0,964	0,722	0	Pyramidal (CA2)	Cntn3
0	0,985109226	0,963	0,824	0	Pyramidal (CA2)	Pex5l
0	0,983603548	0,914	0,702	0	Pyramidal (CA2)	Pde1a
0	0,935630815	1	0,903	0	Pyramidal (CA2)	Snhg11
0	0,932915372	0,998	0,926	0	Pyramidal (CA2)	Ptprn2
0	0,928144582	0,998	0,929	0	Pyramidal (CA2)	Ralyl
0	0,920722324	0,999	0,972	0	Pyramidal (CA2)	Grm5
0	0,916806215	0,965	0,829	0	Pyramidal (CA2)	Hs6st3
0	0,907169567	0,79	0,515	0	Pyramidal (CA2)	Slc35f1
0	0,903036823	0,966	0,782	0	Pyramidal (CA2)	Khdrbs2
0	0,898743356	0,889	0,393	0	Pyramidal (CA2)	Sv2b
0	0,897849326	0,917	0,672	0	Pyramidal (CA2)	Kcnn2
0	0,897690642	0,866	0,488	0	Pyramidal (CA2)	Ptprt
0	0,894163504	0,981	0,932	0	Pyramidal (CA2)	Schip1
0	0,886858931	0,809	0,404	0	Pyramidal (CA2)	Efr3a
0	0,886190278	0,992	0,869	0	Pyramidal (CA2)	Cacna2d3
0	0,885923436	0,849	0,401	0	Pyramidal (CA2)	Rab3c
0	0,86567342	0,925	0,674	0	Pyramidal (CA2)	Thrb
0	0,860432064	0,816	0,578	0	Pyramidal (CA2)	Cntnap5b
0	0,85363101	0,936	0,66	0	Pyramidal (CA2)	Caln1
0	0,852585333	0,998	0,974	0	Pyramidal (CA2)	Tenm2
0	0,847205634	0,994	0,849	0	Pyramidal (CA2)	Rian
0	0,839729965	0,541	0,123	0	Pyramidal (CA2)	Rspo2
0	0,834963101	0,907	0,721	0	Pyramidal (CA2)	Chrm3
0	0,832837895	0,949	0,63	0	Pyramidal (CA2)	Gabrg3
0	0,832411918	0,979	0,753	0	Pyramidal (CA2)	Prkcb
0	0,829475878	0,629	0,192	0	Pyramidal (CA2)	Cntn6
0	0,80403629	0,677	0,302	0	Pyramidal (CA2)	Samd5
0	0,801396578	0,633	0,235	0	Pyramidal (CA2)	Efna5
0	0,796381452	0,981	0,903	0	Pyramidal (CA2)	Asic2
0	0,795950967	0,703	0,35	0	Pyramidal (CA2)	Sorcs1
0	0,789255526	0,77	0,375	0	Pyramidal (CA2)	4930419G24Rik
0	0,788171669	0,948	0,694	0	Pyramidal (CA2)	Rapgef5

0	0,788050238	0,682	0,27	0	Pyramidal (CA2)	Adgrl2
0	0,77883627	0,816	0,463	0	Pyramidal (CA2)	Rit2
0	0,765627168	0,915	0,566	0	Pyramidal (CA2)	A230006K03Rik
0	0,764989735	0,997	0,876	0	Pyramidal (CA2)	Slc8a1
0	0,76191349	1	0,971	0	Pyramidal (CA2)	Phactr1
0	0,756526175	0,995	0,954	0	Pyramidal (CA2)	Cttnbp2
0	0,749901469	0,997	0,912	0	Pyramidal (CA2)	Snap25
0	0,739206126	0,976	0,707	0	Pyramidal (CA2)	Khdrbs3
0	0,733358298	0,755	0,26	0	Pyramidal (CA2)	Cit
0	0,722856609	0,75	0,293	0	Pyramidal (CA2)	Serpini1
0	0,721268099	0,931	0,75	0	Pyramidal (CA2)	Osbpl6
0	0,717617539	0,701	0,406	0	Pyramidal (CA2)	Trpc4
0	0,713137814	0,945	0,913	0	Pyramidal (CA2)	Oxr1
0	0,712689562	0,997	0,983	0	Pyramidal (CA2)	Opcml
0	0,712502675	0,99	0,923	0	Pyramidal (CA2)	Dmd
0	0,710256891	0,913	0,68	0	Pyramidal (CA2)	Zfp385b
0	0,706974171	0,876	0,709	0	Pyramidal (CA2)	Asap1
0	0,706212228	0,957	0,816	0	Pyramidal (CA2)	Cacnb4
0	0,700588191	0,589	0,222	0	Pyramidal (CA2)	Parm1
0	0,696911412	0,705	0,278	0	Pyramidal (CA2)	Elavl4
0	0,693423326	0,854	0,541	0	Pyramidal (CA2)	Vsnl1
0	0,692387571	0,98	0,805	0	Pyramidal (CA2)	Tenm4
0	0,690584495	0,72	0,34	0	Pyramidal (CA2)	Ankrd33b
0	0,686746479	0,562	0,141	0	Pyramidal (CA2)	Adgra1
0	0,686575394	0,996	0,919	0	Pyramidal (CA2)	Arpp21
0	0,686492774	0,645	0,217	0	Pyramidal (CA2)	Tmem200a
0	0,680169674	0,725	0,376	0	Pyramidal (CA2)	Ptprr
0	0,678218434	0,815	0,504	0	Pyramidal (CA2)	Chsy3
0	0,674013258	0,56	0,168	0	Pyramidal (CA2)	4933413L06Rik
0	0,661004564	0,992	0,945	0	Pyramidal (CA2)	R3hdm1
0	0,659494924	0,616	0,266	0	Pyramidal (CA2)	Cpne7
0	0,659409605	0,748	0,338	0	Pyramidal (CA2)	Pcnx2
0	0,657981091	0,707	0,271	0	Pyramidal (CA2)	Mpped1
0	0,656796548	0,683	0,3	0	Pyramidal (CA2)	Tmem132d
0	0,656347109	0,896	0,737	0	Pyramidal (CA2)	Galnt18
0	0,656087364	0,99	0,855	0	Pyramidal (CA2)	Lingo2
0	0,654683972	0,996	0,952	0	Pyramidal (CA2)	Lrfn5
0	0,651633706	0,74	0,379	0	Pyramidal (CA2)	Nyap2
0	0,648576491	0,975	0,887	0	Pyramidal (CA2)	Pde10a
0	0,646986434	0,837	0,703	0	Pyramidal (CA2)	Nos1ap
0	0,636872932	0,835	0,542	0	Pyramidal (CA2)	Lrrtm3
0	0,63491601	0,977	0,909	0	Pyramidal (CA2)	Stxbp5l
0	0,63226475	0,755	0,373	0	Pyramidal (CA2)	Esrrg

0	0,63171214	0,955	0,778	0	Pyramidal (CA2)	Fam189a1
0	0,631218927	0,584	0,267	0	Pyramidal (CA2)	Man2a1
0	0,631062939	0,556	0,203	0	Pyramidal (CA2)	Etv5
0	0,625120668	0,977	0,935	0	Pyramidal (CA2)	Epha5
0	0,624240559	0,588	0,284	0	Pyramidal (CA2)	Pcsk5
0	0,623254389	0,658	0,353	0	Pyramidal (CA2)	Adcy8
0	0,621862637	0,996	0,965	0	Pyramidal (CA2)	Ube3a
0	0,610385265	0,986	0,786	0	Pyramidal (CA2)	Kcnq5
0	0,605371169	0,614	0,249	0	Pyramidal (CA2)	Galnt9
0	0,604654584	0,906	0,67	0	Pyramidal (CA2)	Arhgap26
0	0,602945092	0,763	0,377	0	Pyramidal (CA2)	B830012L14Rik
0	0,602748567	1	0,98	0	Pyramidal (CA2)	Celf2
0	0,599831293	0,504	0,112	0	Pyramidal (CA2)	Chst8
0	0,586441592	0,751	0,37	0	Pyramidal (CA2)	Prkcg
0	0,585427644	0,977	0,906	0	Pyramidal (CA2)	Kcnb2
0	0,584151719	0,675	0,342	0	Pyramidal (CA2)	Crim1
0	0,582713307	0,78	0,437	0	Pyramidal (CA2)	Camk4
0	0,582507732	0,625	0,306	0	Pyramidal (CA2)	Cacng3
0	0,581039673	0,996	0,949	0	Pyramidal (CA2)	Hivep2
0	0,580140596	0,511	0,161	0	Pyramidal (CA2)	Pbx3
0	0,575790875	0,553	0,291	0	Pyramidal (CA2)	Dnah9
0	0,571861556	0,955	0,78	0	Pyramidal (CA2)	Miat
0	0,571579128	0,713	0,368	0	Pyramidal (CA2)	Man1a
0	0,570561689	0,774	0,412	0	Pyramidal (CA2)	Plcl2
0	0,568072923	0,891	0,773	0	Pyramidal (CA2)	Grid2
0	0,567546243	0,584	0,252	0	Pyramidal (CA2)	Rbms1
0	0,566115802	0,948	0,921	0	Pyramidal (CA2)	Gria3
0	0,565523076	0,643	0,254	0	Pyramidal (CA2)	Igsf9b
0	0,55985888	0,876	0,585	0	Pyramidal (CA2)	Xylt1
0	0,559807954	0,654	0,308	0	Pyramidal (CA2)	Garnl3
0	0,551886349	0,647	0,288	0	Pyramidal (CA2)	Chrd
0	0,551120846	0,869	0,819	0	Pyramidal (CA2)	Cdh8
0	0,547938505	0,606	0,252	0	Pyramidal (CA2)	Gm37459
0	0,540880355	0,836	0,616	0	Pyramidal (CA2)	Galnt13
0	0,540321599	0,758	0,436	0	Pyramidal (CA2)	Sorl1
0	0,539562736	0,994	0,94	0	Pyramidal (CA2)	Snhg14
0	0,53523042	0,543	0,165	0	Pyramidal (CA2)	Plekha7
0	0,525201714	0,714	0,355	0	Pyramidal (CA2)	Mirg
0	0,523845397	0,54	0,304	0	Pyramidal (CA2)	A330008L17Rik
0	0,52234069	1	0,982	0	Pyramidal (CA2)	Nrg3
0	0,516480388	0,733	0,409	0	Pyramidal (CA2)	Arid5b
0	0,513695634	1	0,988	0	Pyramidal (CA2)	Anks1b
0	0,512205119	0,621	0,316	0	Pyramidal (CA2)	Sorcs2

0	0,510985901	0,629	0,307	0	Pyramidal (CA2)	Fnbp1l
0	0,510905779	0,526	0,191	0	Pyramidal (CA2)	Gap43
0	0,508364301	0,745	0,399	0	Pyramidal (CA2)	Peg3
1,91E-303	0,67562102	0,513	0,291	5,15E-299	Pyramidal (CA2)	Htr1f
2,05E-266	0,507760563	0,935	0,915	5,52E-262	Pyramidal (CA2)	Cadm1
1,99E-226	0,578108079	0,821	0,731	5,35E-222	Pyramidal (CA2)	Cntn5
5,87E-191	0,678022845	0,797	0,816	1,58E-186	Pyramidal (CA2)	Robo1
1,66E-172	0,641870871	0,795	0,696	4,48E-168	Pyramidal (CA2)	Gria4
3,15E-118	0,64658309	0,778	0,75	8,49E-114	Pyramidal (CA2)	Nrg1

0	2,428899419	0,757	0,119	0	Pyramidal (CA3)	Gm32647
0	2,218682164	0,984	0,521	0	Pyramidal (CA3)	Hs3st4
0	2,208578291	0,998	0,617	0	Pyramidal (CA3)	Mgat4c
0	2,144766068	0,977	0,45	0	Pyramidal (CA3)	Grik4
0	1,904977186	0,971	0,779	0	Pyramidal (CA3)	Kcnq5
0	1,86514711	0,957	0,366	0	Pyramidal (CA3)	Trhde
0	1,858629894	0,887	0,466	0	Pyramidal (CA3)	Trps1
0	1,806901804	0,978	0,368	0	Pyramidal (CA3)	Cpne4
0	1,590012988	0,948	0,836	0	Pyramidal (CA3)	Tafa1
0	1,576283557	0,897	0,179	0	Pyramidal (CA3)	Ccbe1
0	1,541100645	0,977	0,452	0	Pyramidal (CA3)	Spock1
0	1,462196661	0,884	0,332	0	Pyramidal (CA3)	Cntnap5c
0	1,429231031	1	0,928	0	Pyramidal (CA3)	Schip1
0	1,416425935	1	0,909	0	Pyramidal (CA3)	Pde4d
0	1,408181972	0,997	0,946	0	Pyramidal (CA3)	Frmpd4
0	1,370032839	0,816	0,281	0	Pyramidal (CA3)	Slit2
0	1,357960448	0,917	0,382	0	Pyramidal (CA3)	Gm49678
0	1,337316603	0,995	0,86	0	Pyramidal (CA3)	Prkca
0	1,330694993	0,791	0,141	0	Pyramidal (CA3)	Nectin3
0	1,313315225	0,996	0,694	0	Pyramidal (CA3)	Khdrbs3
0	1,302999052	0,988	0,721	0	Pyramidal (CA3)	Shisa6
0	1,265721094	0,999	0,927	0	Pyramidal (CA3)	Epha6
0	1,258400798	0,998	0,941	0	Pyramidal (CA3)	Cacna1a
0	1,244630352	0,841	0,254	0	Pyramidal (CA3)	Raver2
0	1,230567583	1	0,941	0	Pyramidal (CA3)	Nrxn3
0	1,220658787	0,99	0,832	0	Pyramidal (CA3)	Camk1d
0	1,219699734	0,996	0,893	0	Pyramidal (CA3)	Clstn2
0	1,202419755	0,978	0,694	0	Pyramidal (CA3)	Nkain3
0	1,193137667	0,985	0,737	0	Pyramidal (CA3)	9530059O14Rik
0	1,191650226	1	0,954	0	Pyramidal (CA3)	Cadps
0	1,190586012	0,999	0,929	0	Pyramidal (CA3)	Dock4
0	1,183362319	0,879	0,506	0	Pyramidal (CA3)	Neto1
0	1,147172246	0,992	0,832	0	Pyramidal (CA3)	Unc5c

0	1,116518008	0,701	0,244	0	Pyramidal (CA3)	Cpne7
0	1,101235706	0,991	0,887	0	Pyramidal (CA3)	Dcc
0	1,072262639	0,961	0,935	0	Pyramidal (CA3)	Epha5
0	1,065433384	0,788	0,44	0	Pyramidal (CA3)	Epha3
0	1,062314552	0,795	0,19	0	Pyramidal (CA3)	Rnf182
0	1,062173265	0,802	0,192	0	Pyramidal (CA3)	Fstl5
0	1,04084277	0,997	0,81	0	Pyramidal (CA3)	Ryr3
0	1,031615815	0,916	0,411	0	Pyramidal (CA3)	Thsd7b
0	1,029769159	0,979	0,689	0	Pyramidal (CA3)	Ak5
0	1,007365626	0,905	0,371	0	Pyramidal (CA3)	Sv2b
0	1,000923459	0,999	0,906	0	Pyramidal (CA3)	Cacna1c
0	0,982896257	0,795	0,301	0	Pyramidal (CA3)	Peak1
0	0,979822343	0,944	0,628	0	Pyramidal (CA3)	Nrp1
0	0,975863066	0,852	0,25	0	Pyramidal (CA3)	Elavl2
0	0,97160338	0,806	0,307	0	Pyramidal (CA3)	Akap13
0	0,969219696	0,72	0,197	0	Pyramidal (CA3)	Cachd1
0	0,962553904	0,645	0,115	0	Pyramidal (CA3)	Rerg
0	0,956597488	0,996	0,983	0	Pyramidal (CA3)	Opcml
0	0,953863456	0,997	0,905	0	Pyramidal (CA3)	Prkg1
0	0,94524484	0,974	0,707	0	Pyramidal (CA3)	Chrm3
0	0,940014972	0,793	0,39	0	Pyramidal (CA3)	Cdh9
0	0,937186778	0,797	0,287	0	Pyramidal (CA3)	Gm12296
0	0,930478316	0,788	0,322	0	Pyramidal (CA3)	Brinp2
0	0,922566449	0,719	0,206	0	Pyramidal (CA3)	Dpf3
0	0,922250113	0,999	0,978	0	Pyramidal (CA3)	Rbfox1
0	0,918525126	0,911	0,51	0	Pyramidal (CA3)	Rnf112
0	0,909015599	0,989	0,864	0	Pyramidal (CA3)	Cacna2d3
0	0,905816668	0,996	0,83	0	Pyramidal (CA3)	Galnt17
0	0,900132662	0,991	0,891	0	Pyramidal (CA3)	Pcsk2
0	0,888451594	0,851	0,394	0	Pyramidal (CA3)	Kcnc2
0	0,885930721	0,763	0,218	0	Pyramidal (CA3)	Pkp2
0	0,876791777	0,794	0,294	0	Pyramidal (CA3)	Efr3b
0	0,872717853	0,709	0,21	0	Pyramidal (CA3)	Thsd4
0	0,871102739	0,898	0,521	0	Pyramidal (CA3)	Luzp2
0	0,870508103	0,994	0,904	0	Pyramidal (CA3)	Cdk14
0	0,868645241	0,937	0,621	0	Pyramidal (CA3)	Focad
0	0,865719067	0,985	0,898	0	Pyramidal (CA3)	Dok6
0	0,862911565	0,996	0,879	0	Pyramidal (CA3)	Celf4
0	0,859121678	0,973	0,906	0	Pyramidal (CA3)	Stxbp5l
0	0,849055519	0,841	0,4	0	Pyramidal (CA3)	Susd6
0	0,845338038	0,64	0,113	0	Pyramidal (CA3)	9530026P05Rik
0	0,842439107	1	0,964	0	Pyramidal (CA3)	Rims1
0	0,841946035	0,964	0,696	0	Pyramidal (CA3)	Epha4

0	0,840504701	0,724	0,234	0	Pyramidal (CA3)	Sema3e
0	0,830989186	0,752	0,296	0	Pyramidal (CA3)	Nwd2
0	0,830695985	0,925	0,681	0	Pyramidal (CA3)	Sh3gl2
0	0,824869162	1	0,795	0	Pyramidal (CA3)	Zbtb20
0	0,82009738	0,629	0,227	0	Pyramidal (CA3)	6530403H02Rik
0	0,807749351	0,993	0,874	0	Pyramidal (CA3)	Cntnap2
0	0,786543055	0,974	0,905	0	Pyramidal (CA3)	Brinp1
0	0,775062081	0,972	0,827	0	Pyramidal (CA3)	Rapgef2
0	0,774316069	0,969	0,857	0	Pyramidal (CA3)	Dgkb
0	0,773527579	1	0,93	0	Pyramidal (CA3)	Gria1
0	0,769190538	0,792	0,343	0	Pyramidal (CA3)	Cpne6
0	0,765205633	0,987	0,827	0	Pyramidal (CA3)	Nr3c2
0	0,7472869	0,949	0,667	0	Pyramidal (CA3)	Zfp385b
0	0,745254774	0,74	0,572	0	Pyramidal (CA3)	Zfp804a
0	0,734459093	0,98	0,852	0	Pyramidal (CA3)	Gm26871
0	0,732709054	1	0,967	0	Pyramidal (CA3)	Erc2
0	0,732223893	0,862	0,51	0	Pyramidal (CA3)	Tanc1
0	0,727577369	0,688	0,235	0	Pyramidal (CA3)	4921534H16Rik
0	0,725644223	0,66	0,225	0	Pyramidal (CA3)	Ccnd2
0	0,725288535	0,815	0,373	0	Pyramidal (CA3)	Arhgef25
0	0,722542129	1	0,985	0	Pyramidal (CA3)	Nlgn1
0	0,72237539	0,898	0,489	0	Pyramidal (CA3)	Slit1
0	0,72090104	0,925	0,734	0	Pyramidal (CA3)	Ano3
0	0,718298982	0,965	0,788	0	Pyramidal (CA3)	Klhl29
0	0,710863892	0,864	0,432	0	Pyramidal (CA3)	Slc17a7
0	0,709580267	0,715	0,286	0	Pyramidal (CA3)	Gpi1
0	0,701541581	0,991	0,836	0	Pyramidal (CA3)	Nell2
0	0,698651754	0,714	0,303	0	Pyramidal (CA3)	Sgk1
0	0,694418921	0,756	0,329	0	Pyramidal (CA3)	Stim2
0	0,691540046	0,904	0,743	0	Pyramidal (CA3)	Dgkg
0	0,689123034	0,654	0,227	0	Pyramidal (CA3)	Adam11
0	0,6883862	0,758	0,334	0	Pyramidal (CA3)	Lrrn2
0	0,688083892	0,999	0,977	0	Pyramidal (CA3)	Nav3
0	0,685615594	0,994	0,917	0	Pyramidal (CA3)	Enox1
0	0,680635893	0,807	0,42	0	Pyramidal (CA3)	Camk4
0	0,680633471	0,964	0,754	0	Pyramidal (CA3)	Mctp1
0	0,680079708	0,984	0,871	0	Pyramidal (CA3)	Cacnb2
0	0,677628115	0,659	0,331	0	Pyramidal (CA3)	Asph
0	0,671091067	0,758	0,411	0	Pyramidal (CA3)	Parp8
0	0,660971293	0,993	0,896	0	Pyramidal (CA3)	Cnksr2
0	0,660753863	1	0,981	0	Pyramidal (CA3)	Nrg3
0	0,658900969	0,786	0,351	0	Pyramidal (CA3)	Prkcg
0	0,657747457	0,605	0,163	0	Pyramidal (CA3)	Arhgef26

0	0,65558596	0,82	0,468	0	Pyramidal (CA3)	Dpyd
0	0,652890404	0,682	0,254	0	Pyramidal (CA3)	Col4a2
0	0,650676993	0,959	0,855	0	Pyramidal (CA3)	Syn2
0	0,650007533	0,81	0,522	0	Pyramidal (CA3)	Gm36975
0	0,64930511	0,843	0,444	0	Pyramidal (CA3)	Sipa1l3
0	0,646894291	0,603	0,156	0	Pyramidal (CA3)	Neurod6
0	0,645417638	0,836	0,468	0	Pyramidal (CA3)	Nin
0	0,64309957	0,685	0,272	0	Pyramidal (CA3)	Rasgrp1
0	0,639682412	0,726	0,313	0	Pyramidal (CA3)	Traf3
0	0,63906802	1	0,975	0	Pyramidal (CA3)	Syt1
0	0,633864665	0,626	0,206	0	Pyramidal (CA3)	2900026A02Rik
0	0,632946755	0,998	0,947	0	Pyramidal (CA3)	Ryr2
0	0,632315	0,998	0,969	0	Pyramidal (CA3)	Ccser1
0	0,630896529	0,531	0,139	0	Pyramidal (CA3)	Smoc2
0	0,630817966	0,522	0,108	0	Pyramidal (CA3)	Nrip3
0	0,630148292	0,989	0,889	0	Pyramidal (CA3)	Msra
0	0,629060634	0,982	0,799	0	Pyramidal (CA3)	Robo1
0	0,624183043	0,709	0,311	0	Pyramidal (CA3)	Wscd2
0	0,623667772	0,628	0,239	0	Pyramidal (CA3)	Gm10649
0	0,623539349	0,676	0,273	0	Pyramidal (CA3)	Iqsec3
0	0,620429671	0,852	0,562	0	Pyramidal (CA3)	Mpped2
0	0,619656387	0,538	0,156	0	Pyramidal (CA3)	Clmp
0	0,616910964	0,574	0,334	0	Pyramidal (CA3)	Tead1
0	0,616611957	0,954	0,689	0	Pyramidal (CA3)	Pde1a
0	0,615664524	1	0,961	0	Pyramidal (CA3)	Dlgap2
0	0,615035687	0,878	0,511	0	Pyramidal (CA3)	Ptk2b
0	0,61327467	0,504	0,096	0	Pyramidal (CA3)	Galnt3
0	0,611118542	0,516	0,111	0	Pyramidal (CA3)	2310002F09Rik
0	0,610382762	0,654	0,255	0	Pyramidal (CA3)	Stmn2
0	0,607950381	0,958	0,828	0	Pyramidal (CA3)	Aak1
0	0,607188333	0,946	0,782	0	Pyramidal (CA3)	Aopep
0	0,60241073	0,595	0,23	0	Pyramidal (CA3)	4921511C10Rik
0	0,60108078	0,942	0,688	0	Pyramidal (CA3)	Tmem108
0	0,600103415	0,966	0,859	0	Pyramidal (CA3)	Gm20642
0	0,598643933	0,925	0,701	0	Pyramidal (CA3)	Grm1
0	0,593358586	0,967	0,829	0	Pyramidal (CA3)	Dync1i1
0	0,591910864	0,85	0,607	0	Pyramidal (CA3)	Rtl4
0	0,589427179	0,808	0,479	0	Pyramidal (CA3)	Wipf3
0	0,589289923	0,968	0,796	0	Pyramidal (CA3)	Dock9
0	0,589125514	0,949	0,851	0	Pyramidal (CA3)	Ptprg
0	0,58709048	0,94	0,762	0	Pyramidal (CA3)	Cdh11
0	0,585873469	0,591	0,2	0	Pyramidal (CA3)	Gm16183
0	0,582699862	0,931	0,727	0	Pyramidal (CA3)	Galnt18

0	0,582295914	0,904	0,733	0	Pyramidal (CA3)	Epha7
0	0,580156568	0,975	0,872	0	Pyramidal (CA3)	Nrg3os
0	0,578547715	0,537	0,125	0	Pyramidal (CA3)	Hpca
0	0,57827399	0,996	0,909	0	Pyramidal (CA3)	Snap25
0	0,577713045	0,519	0,121	0	Pyramidal (CA3)	Socs2
0	0,577380133	0,789	0,447	0	Pyramidal (CA3)	Trpc5
0	0,57287271	0,944	0,765	0	Pyramidal (CA3)	Aff3
0	0,572207024	0,997	0,936	0	Pyramidal (CA3)	Prkce
0	0,570128707	0,929	0,733	0	Pyramidal (CA3)	Prkag2
0	0,569853307	0,887	0,624	0	Pyramidal (CA3)	Adcy9
0	0,567505316	0,653	0,283	0	Pyramidal (CA3)	Gabra5
0	0,565814544	0,907	0,726	0	Pyramidal (CA3)	Bcl11a
0	0,563616545	0,636	0,238	0	Pyramidal (CA3)	Tgfbr3
0	0,563173964	0,69	0,442	0	Pyramidal (CA3)	Fstl4
0	0,560595136	0,576	0,2	0	Pyramidal (CA3)	Col4a1
0	0,560453112	0,795	0,428	0	Pyramidal (CA3)	Kcnq2
0	0,557589348	0,761	0,392	0	Pyramidal (CA3)	Matk
0	0,555128324	0,523	0,132	0	Pyramidal (CA3)	Golm1
0	0,554775813	0,707	0,356	0	Pyramidal (CA3)	Slc4a7
0	0,554764108	0,654	0,281	0	Pyramidal (CA3)	Nrp2
0	0,553416477	0,993	0,937	0	Pyramidal (CA3)	Mapk10
0	0,54956183	0,967	0,88	0	Pyramidal (CA3)	Pclo
0	0,548065014	0,942	0,774	0	Pyramidal (CA3)	Amph
0	0,547374586	0,713	0,353	0	Pyramidal (CA3)	Myrip
0	0,547027666	0,657	0,307	0	Pyramidal (CA3)	Gm13269
0	0,544100325	0,745	0,395	0	Pyramidal (CA3)	Madd
0	0,539233338	0,999	0,965	0	Pyramidal (CA3)	Grm7
0	0,538363165	0,743	0,394	0	Pyramidal (CA3)	Efr3a
0	0,534878922	0,875	0,608	0	Pyramidal (CA3)	Acp1
0	0,533808648	0,653	0,301	0	Pyramidal (CA3)	Smpd3
0	0,532655073	0,809	0,479	0	Pyramidal (CA3)	Dnm1
0	0,531053748	0,618	0,32	0	Pyramidal (CA3)	Lmo4
0	0,530801848	0,993	0,958	0	Pyramidal (CA3)	Xkr4
0	0,529934499	0,668	0,307	0	Pyramidal (CA3)	Cul4a
0	0,525225994	0,987	0,841	0	Pyramidal (CA3)	Tmem178b
0	0,52425159	0,675	0,31	0	Pyramidal (CA3)	Lingo1
0	0,520205866	0,997	0,95	0	Pyramidal (CA3)	Gabrb3
0	0,520125858	0,999	0,938	0	Pyramidal (CA3)	Camk2a
0	0,516455449	0,559	0,205	0	Pyramidal (CA3)	Bicc1
0	0,51434705	0,91	0,706	0	Pyramidal (CA3)	Dnajc6
0	0,513592719	0,595	0,23	0	Pyramidal (CA3)	Cabp1
0	0,510202692	0,88	0,683	0	Pyramidal (CA3)	Rimbp2
0	0,509125432	0,849	0,676	0	Pyramidal (CA3)	Slc35f3

0	0,50865304	0,988	0,857	0	Pyramidal (CA3)	Atp2b1
0	0,508198108	0,802	0,444	0	Pyramidal (CA3)	Runx1t1
0	0,506727478	0,999	0,899	0	Pyramidal (CA3)	Snhg11
0	0,504438784	0,935	0,913	0	Pyramidal (CA3)	Oxr1
0	0,503039261	1	0,983	0	Pyramidal (CA3)	Gria2
0	0,502069281	1	1	0	Pyramidal (CA3)	Malat1
0	0,501784806	0,551	0,197	0	Pyramidal (CA3)	Rgs11
0	0,500187109	0,891	0,697	0	Pyramidal (CA3)	Kcnt2

0	3,022500861	0,879	0,331	0	Interneurons	Erbb4
0	2,564408104	0,984	0,744	0	Interneurons	Grip1
0	2,466047163	0,764	0,1	0	Interneurons	Nxph1
0	2,439878623	0,647	0,457	0	Interneurons	Adarb2
0	2,229098967	0,774	0,148	0	Interneurons	Grik1
0	2,149632303	0,747	0,035	0	Interneurons	Kcnmb2
0	2,06579625	0,921	0,24	0	Interneurons	Rbms3
0	2,05164543	0,836	0,386	0	Interneurons	Kcnc2
0	1,787834702	0,581	0,094	0	Interneurons	C130073E24Rik
0	1,724532885	0,61	0,246	0	Interneurons	Col25a1
0	1,640692222	0,551	0,074	0	Interneurons	Col19a1
0	1,628223802	0,894	0,615	0	Interneurons	Gabrg3
0	1,601500209	0,741	0,192	0	Interneurons	Ptprm
0	1,507424934	0,683	0,34	0	Interneurons	Fgf13
0	1,506854574	0,932	0,668	0	Interneurons	Galntl6
0	1,470972289	0,697	0,077	0	Interneurons	Kcnip1
0	1,419474203	0,587	0,013	0	Interneurons	Dlx6os1
0	1,39134954	0,619	0,112	0	Interneurons	Cntnap4
0	1,376874885	0,629	0,063	0	Interneurons	Alk
0	1,322858748	0,814	0,37	0	Interneurons	Unc5d
0	1,310465589	0,975	0,668	0	Interneurons	Gria4
0	1,277010909	0,657	0,014	0	Interneurons	Gad2
0	1,219116736	0,585	0,239	0	Interneurons	Zfp804b
0	1,215602511	0,781	0,244	0	Interneurons	Elavl2
0	1,206727088	0,556	0,025	0	Interneurons	Btbd11
0	1,192905251	0,941	0,8	0	Interneurons	Robo1
0	1,18364845	0,996	0,94	0	Interneurons	Nrxn3
0	1,17733423	1	0,897	0	Interneurons	Snhg11
0	1,156868232	0,789	0,223	0	Interneurons	Cit
0	1,147369808	0,988	0,925	0	Interneurons	Fgf12
0	1,11204349	0,647	0,064	0	Interneurons	Gad1
0	1,098338684	0,999	0,976	0	Interneurons	Meg3
0	1,096668758	0,873	0,719	0	Interneurons	Cntn5
0	1,025035039	0,519	0,106	0	Interneurons	Kctd8

0	1,019525709	0,522	0,07	0	Interneurons	Gm38505
0	1,013311363	0,795	0,282	0	Interneurons	Sox2ot
0	1,00884157	0,629	0,07	0	Interneurons	Nhs
0	1,003012674	0,895	0,727	0	Interneurons	Etl4
0	0,997194269	0,8	0,325	0	Interneurons	Usp29
0	0,994023585	0,554	0,045	0	Interneurons	Maf
0	0,963753278	0,887	0,641	0	Interneurons	Astn2
0	0,95314958	0,663	0,316	0	Interneurons	Cdh13
0	0,942826709	0,841	0,419	0	Interneurons	Spock3
0	0,938858277	0,992	0,871	0	Interneurons	Cntnap2
0	0,919906295	0,822	0,555	0	Interneurons	Sgcz
0	0,919239493	0,872	0,46	0	Interneurons	5330434G04Rik
0	0,913738904	0,588	0,22	0	Interneurons	Sdk1
0	0,911724863	0,787	0,339	0	Interneurons	Zfp536
0	0,908250376	0,761	0,419	0	Interneurons	Npas3
0	0,888988839	0,828	0,366	0	Interneurons	Peg3
0	0,887151673	0,627	0,203	0	Interneurons	Myo16
0	0,879379157	0,816	0,376	0	Interneurons	Rab3c
0	0,867769649	0,866	0,449	0	Interneurons	A330076H08Rik
0	0,857625486	0,708	0,253	0	Interneurons	Dner
0	0,855749344	0,584	0,204	0	Interneurons	Fstl5
0	0,852956186	0,866	0,673	0	Interneurons	Tenm3
0	0,852734403	0,633	0,258	0	Interneurons	Cdh18
0	0,838776504	0,745	0,349	0	Interneurons	Esrrg
0	0,830616338	0,652	0,157	0	Interneurons	Slc6a1
0	0,822793249	0,877	0,706	0	Interneurons	Tenm1
0	0,804978242	0,988	0,84	0	Interneurons	Rian
0	0,799037067	0,873	0,63	0	Interneurons	Slc2a13
0	0,797224859	0,992	0,957	0	Interneurons	Xkr4
0	0,792841897	0,943	0,902	0	Interneurons	Asic2
0	0,788851742	0,801	0,614	0	Interneurons	Zmat4
0	0,788653158	0,594	0,168	0	Interneurons	Vstm2a
0	0,786658792	0,577	0,143	0	Interneurons	Kcnc1
0	0,76521104	0,715	0,358	0	Interneurons	Nyap2
0	0,764060993	0,954	0,77	0	Interneurons	A230057D06Rik
0	0,751226624	0,613	0,238	0	Interneurons	Ubash3b
0	0,750680273	0,656	0,376	0	Interneurons	Thsd7a
0	0,740245003	0,646	0,238	0	Interneurons	Rasgrf2
0	0,732155714	0,991	0,952	0	Interneurons	Sntg1
0	0,727969762	0,983	0,875	0	Interneurons	Rims2
0	0,724432356	0,894	0,652	0	Interneurons	Hcn1
0	0,721243335	0,876	0,683	0	Interneurons	Pam
0	0,707588417	0,549	0,173	0	Interneurons	Cntn6

0	0,703160504	0,761	0,368	0	Interneurons	Ptchd4
0	0,699064361	0,772	0,463	0	Interneurons	Sh3rf3
0	0,696299529	0,538	0,268	0	Interneurons	Grm8
0	0,690982246	0,989	0,908	0	Interneurons	Pde4d
0	0,680930546	0,571	0,201	0	Interneurons	Osbpl3
0	0,679133807	0,859	0,666	0	Interneurons	Thrb
0	0,662836883	0,707	0,279	0	Interneurons	Garnl3
0	0,658135826	0,687	0,444	0	Interneurons	Slc44a5
0	0,655633072	0,584	0,283	0	Interneurons	Serpini1
0	0,655621632	0,703	0,34	0	Interneurons	Cntnap5c
0	0,637231201	0,52	0,126	0	Interneurons	Ank1
0	0,633432753	0,732	0,329	0	Interneurons	Mirg
0	0,632795484	0,858	0,671	0	Interneurons	Zfp385b
0	0,623716117	0,814	0,494	0	Interneurons	Slc35f1
0	0,620484529	0,994	0,964	0	Interneurons	Ube3a
0	0,615235101	0,739	0,338	0	Interneurons	Dlc1
0	0,613278957	0,839	0,553	0	Interneurons	Scn1a
0	0,608037131	0,602	0,285	0	Interneurons	Tmem132d
0	0,603601216	0,763	0,636	0	Interneurons	Cntn4
0	0,603150614	0,962	0,878	0	Interneurons	Pclo
0	0,598451447	0,993	0,952	0	Interneurons	Dpp6
0	0,597553279	0,994	0,936	0	Interneurons	Snhg14
0	0,596779155	0,707	0,572	0	Interneurons	Zfp804a
0	0,596156496	0,728	0,351	0	Interneurons	C530008M17Rik
0	0,591604714	0,735	0,43	0	Interneurons	Srrm4
0	0,590607406	0,633	0,429	0	Interneurons	Synpr
0	0,587057441	0,993	0,954	0	Interneurons	Cadps
0	0,583481656	0,519	0,173	0	Interneurons	Ccdc136
0	0,583127809	0,565	0,203	0	Interneurons	Plcb4
0	0,57741461	0,508	0,135	0	Interneurons	Ankrd24
0	0,570510954	0,516	0,198	0	Interneurons	Utrn
0	0,560129923	0,711	0,517	0	Interneurons	Neto1
0	0,556102352	0,87	0,637	0	Interneurons	Klf12
0	0,555927997	0,522	0,164	0	Interneurons	Cux2
0	0,555270204	0,705	0,384	0	Interneurons	Ripor2
0	0,55437427	0,722	0,356	0	Interneurons	B830012L14Rik
0	0,554310045	0,769	0,701	0	Interneurons	A830018L16Rik
0	0,550642584	0,54	0,22	0	Interneurons	Efna5
0	0,54485896	0,799	0,557	0	Interneurons	A230006K03Rik
0	0,542441588	0,505	0,14	0	Interneurons	Abat
0	0,530627484	0,52	0,161	0	Interneurons	Dcx
0	0,530409111	0,979	0,961	0	Interneurons	Grik2
0	0,527246068	0,8	0,557	0	Interneurons	Pcdh15

0	0,526216448	0,51	0,166	0	Interneurons	9630014M24Rik
0	0,525436485	0,84	0,646	0	Interneurons	Mef2c
0	0,525318811	0,777	0,43	0	Interneurons	Xkr6
0	0,522339612	0,805	0,487	0	Interneurons	Atp8a2
0	0,520332304	0,571	0,235	0	Interneurons	Las1l
0	0,514998312	0,565	0,37	0	Interneurons	Tmtc2
0	0,505784081	0,579	0,288	0	Interneurons	Pdzrn4
1,79E-301	0,524758823	0,661	0,488	4,82E-297	Interneurons	Ptprt
1,44E-93	0,522027992	0,522	0,439	3,88E-89	Interneurons	Ntng1

0	2,211808811	0,857	0,132	0	Microglia	Tgfbr1
0	2,100596115	0,822	0,016	0	Microglia	Inpp5d
0	2,047516344	0,937	0,445	0	Microglia	Plxdc2
0	1,992814237	0,832	0,1	0	Microglia	Zfhx3
0	1,934250729	0,925	0,362	0	Microglia	Elmo1
0	1,779319937	0,706	0,048	0	Microglia	Hexb
0	1,743197463	0,968	0,676	0	Microglia	Srgap2
0	1,735080728	0,708	0,032	0	Microglia	Apbb1ip
0	1,724262378	0,765	0,146	0	Microglia	Epb41l2
0	1,720889318	0,805	0,131	0	Microglia	Mertk
0	1,699178059	0,864	0,352	0	Microglia	Tmcc3
0	1,605334545	0,673	0,012	0	Microglia	Dock8
0	1,560857606	0,665	0,111	0	Microglia	Lrmda
0	1,504000178	0,656	0,008	0	Microglia	Csf1r
0	1,494208939	0,919	0,564	0	Microglia	Mef2a
0	1,463072206	0,602	0,009	0	Microglia	Ly86
0	1,44886489	0,587	0,007	0	Microglia	Siglech
0	1,432167435	0,829	0,447	0	Microglia	Mbnl1
0	1,417523202	0,718	0,264	0	Microglia	Pde3b
0	1,41445148	0,613	0,019	0	Microglia	Dock2
0	1,412726299	0,708	0,192	0	Microglia	Lyn
0	1,41152623	0,597	0,008	0	Microglia	Fyb
0	1,382242262	0,571	0,007	0	Microglia	Ikzf1
0	1,380443742	0,993	0,948	0	Microglia	Nav2
0	1,37815751	0,6	0,05	0	Microglia	Cx3cr1
0	1,371394904	0,598	0,02	0	Microglia	Fli1
0	1,354670274	0,993	0,95	0	Microglia	Tanc2
0	1,346142	0,622	0,008	0	Microglia	Arhgap45
0	1,307788947	0,564	0,007	0	Microglia	Gm10790
0	1,303639616	0,547	0,008	0	Microglia	Runx1
0	1,293990051	0,689	0,205	0	Microglia	Rreb1
0	1,287954799	0,571	0,013	0	Microglia	Slco2b1
0	1,2758885	0,691	0,247	0	Microglia	Pag1

0	1,27331454	0,816	0,374	0	Microglia	Maml3
0	1,241396915	0,814	0,464	0	Microglia	Numb
0	1,208109351	0,951	0,768	0	Microglia	Ldlrad4
0	1,200955953	0,541	0,04	0	Microglia	Itgb5
0	1,194985642	0,536	0,039	0	Microglia	Entpd1
0	1,192843874	0,901	0,541	0	Microglia	Dleu2
0	1,191045956	0,502	0,006	0	Microglia	Selplg
0	1,190771168	0,669	0,177	0	Microglia	Ankrd44
0	1,189280235	0,592	0,144	0	Microglia	Hpgds
0	1,182589469	0,613	0,158	0	Microglia	Zfp710
0	1,17918239	0,61	0,193	0	Microglia	P2ry12
0	1,170536062	0,92	0,615	0	Microglia	Slc9a9
0	1,159256083	0,727	0,389	0	Microglia	Ophn1
0	1,151739995	0,703	0,312	0	Microglia	Gab2
0	1,142792165	0,535	0,021	0	Microglia	Cyth4
0	1,142399639	0,954	0,586	0	Microglia	Foxn3
0	1,136464228	0,516	0,013	0	Microglia	Hk2
0	1,127680155	0,508	0,013	0	Microglia	Adap2
0	1,105249559	0,697	0,397	0	Microglia	4933406I18Rik
0	1,100996628	0,743	0,407	0	Microglia	Fchsd2
0	1,097101604	0,741	0,371	0	Microglia	Pip4k2a
0	1,086392883	0,559	0,112	0	Microglia	Lgmn
0	1,079335072	0,538	0,111	0	Microglia	Lpcat2
0	1,075486818	0,549	0,179	0	Microglia	E230029C05Rik
0	1,074442759	0,571	0,182	0	Microglia	Ivns1abp
0	1,035209264	0,997	0,973	0	Microglia	Csmd3
0	1,022152758	0,507	0,083	0	Microglia	St3gal6
0	1,016073853	0,955	0,805	0	Microglia	Ssh2
0	0,996251757	0,719	0,405	0	Microglia	Snx29
0	0,962661505	0,551	0,141	0	Microglia	Bmp2k
0	0,949832626	0,934	0,655	0	Microglia	Mef2c
0	0,945037214	0,906	0,488	0	Microglia	Frmd4b
0	0,941901771	0,63	0,263	0	Microglia	Ubash3b
0	0,926408679	0,573	0,251	0	Microglia	Sirpa
0	0,91919561	0,547	0,161	0	Microglia	Chn2
0	0,91753115	0,966	0,842	0	Microglia	Chd9
0	0,914935661	0,672	0,366	0	Microglia	Picalm
0	0,892359913	0,741	0,389	0	Microglia	Inpp4b
0	0,886237329	0,515	0,167	0	Microglia	Serinc3
0	0,884938151	0,826	0,554	0	Microglia	Fam49b
0	0,876298013	0,54	0,136	0	Microglia	Cst3
0	0,868739435	0,721	0,449	0	Microglia	Arhgap5
0	0,866298191	0,684	0,416	0	Microglia	Lrch1

0	0,85493726	0,507	0,158	0	Microglia	Wasf2
0	0,834571393	0,956	0,553	0	Microglia	Qk
0	0,833337285	0,532	0,187	0	Microglia	Rrbp1
0	0,811152886	0,769	0,522	0	Microglia	Dennd4a
0	0,778038563	0,563	0,298	0	Microglia	Smap2
0	0,773670531	0,877	0,699	0	Microglia	Rapgef5
0	0,767296796	0,838	0,594	0	Microglia	Rap1gds1
0	0,764651635	0,947	0,787	0	Microglia	Dip2b
0	0,739829321	0,927	0,772	0	Microglia	Ptprj
0	0,733511381	0,583	0,362	0	Microglia	Abhd12
0	0,732504675	0,95	0,526	0	Microglia	Dock10
0	0,695673786	0,922	0,763	0	Microglia	Diaph2
0	0,692300964	0,723	0,49	0	Microglia	Cyfip1
0	0,688840854	0,532	0,295	0	Microglia	Etv6
0	0,686546622	0,577	0,34	0	Microglia	Akap13
0	0,682022834	0,873	0,619	0	Microglia	Wnk1
0	0,671319732	0,905	0,676	0	Microglia	Tbc1d5
0	0,643256301	0,804	0,538	0	Microglia	Bin1
0	0,633056207	0,95	0,639	0	Microglia	Plcl1
0	0,62555676	0,519	0,267	0	Microglia	Ttc28
0	0,607415663	0,58	0,316	0	Microglia	Camk2d
0	0,589458187	0,988	0,933	0	Microglia	Dock4
0	0,576025783	0,806	0,568	0	Microglia	Tacc1
0	0,538464423	0,915	0,543	0	Microglia	Maml2
1,11E-301	0,785456573	0,622	0,421	2,99E-297	Microglia	Plcl2
1,78E-301	0,540534721	0,972	0,891	4,79E-297	Microglia	Ddx5
8,60E-280	0,617647609	0,824	0,622	2,32E-275	Microglia	Zswim6
9,81E-280	0,514677772	0,884	0,71	2,64E-275	Microglia	Phf14
2,26E-274	0,547379544	0,798	0,59	6,08E-270	Microglia	Ppm1h
3,96E-271	0,521516919	0,844	0,646	1,07E-266	Microglia	Pan3
1,22E-264	0,72103933	0,565	0,365	3,28E-260	Microglia	Fhit
4,46E-254	0,616133849	0,651	0,459	1,20E-249	Microglia	Gm35188
2,21E-237	0,542309999	0,746	0,557	5,96E-233	Microglia	Ulk2
1,49E-235	0,613312317	0,542	0,363	4,01E-231	Microglia	Ints6l
2,22E-232	0,63902284	0,955	0,879	5,97E-228	Microglia	Slc8a1
1,04E-225	0,510010623	0,913	0,787	2,80E-221	Microglia	AC149090.1
2,22E-222	0,582028482	0,635	0,479	5,98E-218	Microglia	Slc12a6
3,87E-220	0,562158752	0,708	0,513	1,04E-215	Microglia	Atp8a2
1,12E-212	0,500734691	0,512	0,315	3,01E-208	Microglia	Pik3r1
2,42E-207	0,520633481	0,527	0,351	6,52E-203	Microglia	Arhgap17
1,59E-206	0,545742028	0,503	0,323	4,29E-202	Microglia	Mitf
9,25E-203	0,583808935	0,544	0,37	2,49E-198	Microglia	Fgf13
2,03E-198	0,52317046	0,527	0,351	5,47E-194	Microglia	Sh3kbp1

8,21E-193	0,550043476	0,557	0,397	2,21E-188	Microglia	Snx24
4,46E-173	0,517124051	0,551	0,403	1,20E-168	Microglia	Rnf216
3,06E-158	0,500098879	0,604	0,482	8,24E-154	Microglia	Tmem135
0	3,645919163	0,999	0,513	0	Astrocytes	Slc1a2
0	3,332532409	0,995	0,517	0	Astrocytes	Gpc5
0	3,124710935	0,929	0,11	0	Astrocytes	Apoe
0	2,607687096	0,945	0,163	0	Astrocytes	Slc1a3
0	2,540073128	0,993	0,624	0	Astrocytes	Wdr17
0	2,510057443	0,812	0,087	0	Astrocytes	Cst3
0	2,391858574	0,911	0,095	0	Astrocytes	Atp1a2
0	2,381933802	0,898	0,096	0	Astrocytes	Plpp3
0	2,333349335	0,916	0,244	0	Astrocytes	Prex2
0	2,298988092	0,892	0,157	0	Astrocytes	Rgs20
0	2,284756658	0,993	0,403	0	Astrocytes	Npas3
0	2,23719826	0,985	0,58	0	Astrocytes	Gm3764
0	2,236840213	0,873	0,151	0	Astrocytes	Rorb
0	2,185874299	0,989	0,539	0	Astrocytes	Msi2
0	2,127492628	0,989	0,657	0	Astrocytes	Tspan7
0	2,104436112	0,944	0,279	0	Astrocytes	Ptprz1
0	2,069112999	1	0,794	0	Astrocytes	Ntm
0	2,017016081	1	0,991	0	Astrocytes	Nrxn1
0	1,940185242	0,832	0,223	0	Astrocytes	Sparcl1
0	1,897742701	0,901	0,359	0	Astrocytes	Pitpnc1
0	1,862035579	0,996	0,756	0	Astrocytes	Dtna
0	1,85176405	0,999	0,908	0	Astrocytes	Rora
0	1,850367811	0,775	0,098	0	Astrocytes	Mertk
0	1,825404254	0,982	0,672	0	Astrocytes	Cpe
0	1,815274413	0,955	0,472	0	Astrocytes	Slc4a4
0	1,799074503	0,898	0,398	0	Astrocytes	Farp1
0	1,789073622	0,663	0,044	0	Astrocytes	Clu
0	1,763163618	0,748	0,047	0	Astrocytes	Gm6145
0	1,747973897	0,901	0,519	0	Astrocytes	Luzp2
0	1,697948031	0,805	0,144	0	Astrocytes	Lama2
0	1,689498956	0,947	0,48	0	Astrocytes	Gm42418
0	1,685704904	0,764	0,185	0	Astrocytes	Acsl3
0	1,66113309	1	0,957	0	Astrocytes	Gabrb1
0	1,635081282	0,798	0,268	0	Astrocytes	Sfxn5
0	1,623880033	0,727	0,157	0	Astrocytes	Gm20713
0	1,602907925	0,993	0,703	0	Astrocytes	Nfia
0	1,601821209	0,999	0,931	0	Astrocytes	Mir99ahg
0	1,593177874	0,701	0,059	0	Astrocytes	Nwd1
0	1,571544915	0,741	0,156	0	Astrocytes	Bcan

0	1,527642704	0,765	0,233	0	Astrocytes	Cspg5
0	1,513017373	0,656	0,056	0	Astrocytes	Pla2g7
0	1,501504725	0,632	0,101	0	Astrocytes	Nhsl1
0	1,494380478	0,567	0,047	0	Astrocytes	Mt1
0	1,489383899	1	0,979	0	Astrocytes	Son
0	1,48170897	0,648	0,081	0	Astrocytes	Bmpr1b
0	1,473784175	0,634	0,04	0	Astrocytes	Gli3
0	1,468377544	1	0,982	0	Astrocytes	Ctnnd2
0	1,456166406	0,628	0,056	0	Astrocytes	Htra1
0	1,454360752	0,957	0,525	0	Astrocytes	Grm3
0	1,440835153	0,999	0,963	0	Astrocytes	Macf1
0	1,434500113	0,91	0,585	0	Astrocytes	Hdac8
0	1,432973471	1	0,988	0	Astrocytes	Lsamp
0	1,420061947	0,991	0,698	0	Astrocytes	Mir100hg
0	1,405102891	0,656	0,147	0	Astrocytes	Paqr8
0	1,402180691	0,622	0,135	0	Astrocytes	Phka1
0	1,380218807	0,95	0,463	0	Astrocytes	Ptprt
0	1,373936279	0,763	0,312	0	Astrocytes	Sash1
0	1,371293	0,62	0,08	0	Astrocytes	Phkg1
0	1,363437786	0,61	0,05	0	Astrocytes	Ndrg2
0	1,359818679	0,798	0,367	0	Astrocytes	Nckap5
0	1,357958887	0,765	0,15	0	Astrocytes	Sox6
0	1,327130993	0,529	0,03	0	Astrocytes	Gja1
0	1,325099359	0,589	0,057	0	Astrocytes	Ntsr2
0	1,317668507	0,675	0,177	0	Astrocytes	Appl2
0	1,317614952	0,671	0,244	0	Astrocytes	Ckb
0	1,317254289	0,982	0,758	0	Astrocytes	Gnao1
0	1,301553155	0,573	0,044	0	Astrocytes	Slco1c1
0	1,290648626	0,999	0,942	0	Astrocytes	B3galt1
0	1,281670846	0,994	0,864	0	Astrocytes	Gpm6b
0	1,281188278	0,775	0,429	0	Astrocytes	Arhgap5
0	1,252902351	0,936	0,46	0	Astrocytes	Trps1
0	1,238590116	0,712	0,29	0	Astrocytes	Cmss1
0	1,229745606	0,648	0,162	0	Astrocytes	Rmst
0	1,22593624	0,572	0,089	0	Astrocytes	Glul
0	1,206844723	0,998	0,93	0	Astrocytes	Pbx1
0	1,173063079	0,692	0,332	0	Astrocytes	Ttyh1
0	1,172592387	0,512	0,037	0	Astrocytes	Slc39a12
0	1,169427452	0,731	0,335	0	Astrocytes	Ptn
0	1,168922501	0,508	0,037	0	Astrocytes	F3
0	1,167298249	0,632	0,24	0	Astrocytes	Glud1
0	1,156665639	0,769	0,427	0	Astrocytes	Myo6
0	1,138603304	0,993	0,854	0	Astrocytes	Dgkb

0	1,129953013	0,589	0,163	0	Astrocytes	Arhgef26
0	1,12876213	0,517	0,12	0	Astrocytes	Tprkb
0	1,119340199	0,6	0,123	0	Astrocytes	Hepacam
0	1,108479636	0,688	0,413	0	Astrocytes	Ddhd1
0	1,095707192	0,516	0,05	0	Astrocytes	Prdm16
0	1,093421877	0,707	0,428	0	Astrocytes	Adk
0	1,080116946	0,533	0,113	0	Astrocytes	Prdx6
0	1,071682627	0,986	0,529	0	Astrocytes	Qk
0	1,068667516	0,719	0,442	0	Astrocytes	Gm35188
0	1,059042938	0,586	0,147	0	Astrocytes	Daam2
0	1,051979954	0,956	0,52	0	Astrocytes	Maml2
0	1,049778913	0,958	0,769	0	Astrocytes	Fmn2
0	1,039804921	0,996	0,926	0	Astrocytes	Syne1
0	1,016433445	0,559	0,169	0	Astrocytes	Pard3b
0	1,015889769	0,906	0,619	0	Astrocytes	Zeb1
0	1,014534422	0,524	0,125	0	Astrocytes	Myo10
0	1,012702279	0,546	0,177	0	Astrocytes	Slc27a1
0	1,000841475	0,844	0,38	0	Astrocytes	Gpc6
0	0,999864716	0,962	0,745	0	Astrocytes	Stox2
0	0,967075127	0,987	0,874	0	Astrocytes	Gpm6a
0	0,964975352	0,678	0,491	0	Astrocytes	Cpeb4
0	0,964789176	0,624	0,207	0	Astrocytes	Dock1
0	0,939447803	0,998	0,948	0	Astrocytes	Tnik
0	0,934957136	0,687	0,364	0	Astrocytes	Prkd1
0	0,932141712	0,999	0,977	0	Astrocytes	Frmd4a
0	0,925579878	0,602	0,301	0	Astrocytes	Csgalnact1
0	0,914316444	0,513	0,19	0	Astrocytes	Ugp2
0	0,887490274	0,579	0,35	0	Astrocytes	Camk2g
0	0,885764033	0,611	0,297	0	Astrocytes	Clmn
0	0,882248159	0,535	0,178	0	Astrocytes	Slc6a1
0	0,878638212	0,539	0,249	0	Astrocytes	Mmd2
0	0,876305968	0,991	0,795	0	Astrocytes	Zbtb20
0	0,872444478	0,531	0,278	0	Astrocytes	Vcl
0	0,865435218	0,638	0,47	0	Astrocytes	Ahcyl1
0	0,863321009	0,823	0,62	0	Astrocytes	Arhgef12
0	0,862669147	0,55	0,272	0	Astrocytes	Pygb
0	0,859702162	0,535	0,289	0	Astrocytes	Arhgef4
0	0,843615577	0,973	0,701	0	Astrocytes	Pcdh7
0	0,824104188	0,568	0,382	0	Astrocytes	Mgll
0	0,818132923	0,988	0,871	0	Astrocytes	Ntrk2
0	0,816430101	0,953	0,766	0	Astrocytes	Hnrnpa2b1
0	0,811757579	0,951	0,796	0	Astrocytes	C130071C03Rik
0	0,80910836	0,994	0,908	0	Astrocytes	Cadm1

0	0,808891267	0,999	0,988	0	Astrocytes	Mdga2
0	0,808351986	0,593	0,358	0	Astrocytes	Cdk8
0	0,792107349	0,975	0,654	0	Astrocytes	Trpm3
0	0,791219566	0,641	0,333	0	Astrocytes	Cdh20
0	0,790717857	0,594	0,408	0	Astrocytes	Add3
0	0,783525764	0,518	0,256	0	Astrocytes	Psap
0	0,767123759	0,617	0,479	0	Astrocytes	Mgat5
0	0,763302203	0,999	0,963	0	Astrocytes	Dclk1
0	0,753491507	0,552	0,332	0	Astrocytes	Eps15
0	0,745620257	0,522	0,322	0	Astrocytes	Cep85l
0	0,74268738	0,985	0,884	0	Astrocytes	4930402H24Rik
0	0,735038099	0,623	0,416	0	Astrocytes	Tle4
0	0,731512347	0,969	0,831	0	Astrocytes	Hivep3
0	0,731020229	0,986	0,906	0	Astrocytes	Dennd1a
0	0,729858083	0,547	0,224	0	Astrocytes	Plekhg1
0	0,727285452	0,526	0,335	0	Astrocytes	Acsl6
0	0,723108931	0,694	0,547	0	Astrocytes	Spire1
0	0,722914885	0,981	0,758	0	Astrocytes	Grid2
0	0,692647695	0,991	0,888	0	Astrocytes	Nfib
0	0,683448984	0,964	0,836	0	Astrocytes	Ncoa1
0	0,673090785	0,546	0,396	0	Astrocytes	Fermt2
0	0,671381148	0,998	0,98	0	Astrocytes	Plcb1
0	0,666755033	0,969	0,834	0	Astrocytes	Spag9
0	0,665128026	0,967	0,692	0	Astrocytes	Phlpp1
0	0,660306191	0,591	0,475	0	Astrocytes	Garem1
0	0,658163392	0,547	0,365	0	Astrocytes	Mapk4
0	0,656621723	0,965	0,835	0	Astrocytes	Chd9
0	0,653722542	0,798	0,56	0	Astrocytes	Fut9
0	0,653611719	0,99	0,82	0	Astrocytes	Ppp2r2b
0	0,647459855	0,996	0,965	0	Astrocytes	Gphn
0	0,642383637	0,954	0,703	0	Astrocytes	Cdh10
0	0,638456416	0,538	0,372	0	Astrocytes	Lpp
0	0,635748294	0,973	0,852	0	Astrocytes	Trim9
0	0,632809238	0,75	0,598	0	Astrocytes	Pde8b
0	0,628733844	0,5	0,291	0	Astrocytes	Stard13
0	0,623431745	0,545	0,35	0	Astrocytes	Meis2
0	0,619428175	0,855	0,643	0	Astrocytes	Klf12
0	0,614762231	0,903	0,662	0	Astrocytes	Kcnn2
0	0,607080108	0,622	0,452	0	Astrocytes	Arap2
0	0,606479153	0,989	0,9	0	Astrocytes	Kif1b
0	0,592337461	0,665	0,558	0	Astrocytes	Phactr3
0	0,590926682	0,532	0,448	0	Astrocytes	Fam171b
0	0,588615883	0,886	0,749	0	Astrocytes	Abr

0	0,585288055	0,599	0,294	0	Astrocytes	Glis3
0	0,575856552	0,983	0,878	0	Astrocytes	Fgfr2
0	0,573022954	0,953	0,717	0	Astrocytes	Sema6d
0	0,569843055	0,56	0,375	0	Astrocytes	Tmtc2
0	0,569282497	0,96	0,804	0	Astrocytes	Adcy2
0	0,550005697	0,811	0,578	0	Astrocytes	Xylt1
0	0,547754552	0,505	0,417	0	Astrocytes	Pou2f1
0	0,538323605	0,871	0,734	0	Astrocytes	Kmt2c
0	0,532148583	0,938	0,665	0	Astrocytes	Fnbp1
0	0,525369557	0,955	0,836	0	Astrocytes	Sorbs1
0	0,518084526	0,915	0,778	0	Astrocytes	Phf21a
0	0,516153201	0,836	0,544	0	Astrocytes	Sox5
0	0,503543707	0,654	0,576	0	Astrocytes	Itsn1
0	0,503299502	0,966	0,86	0	Astrocytes	Apc
0	4,457513133	0,994	0,517	0	Endothelial	Gm42418
0	4,345415464	0,99	0,008	0	Endothelial	Flt1
0	3,816933527	0,857	0,018	0	Endothelial	Bsg
0	3,61962414	0,969	0,009	0	Endothelial	Slco1a4
0	3,368482811	0,933	0,321	0	Endothelial	Cmss1
0	3,338833031	0,716	0,004	0	Endothelial	Rgs5
0	3,296079476	0,833	0,002	0	Endothelial	Cldn5
0	3,148259037	0,861	0,09	0	Endothelial	AY036118
0	3,040749438	0,934	0,027	0	Endothelial	Ebf1
0	2,952836094	0,921	0,01	0	Endothelial	Mecom
0	2,894517512	0,674	0,01	0	Endothelial	Cxcl12
0	2,811212958	0,894	0,015	0	Endothelial	Ptprb
0	2,782458732	0,839	0,017	0	Endothelial	Hmcn1
0	2,762096489	0,867	0,321	0	Endothelial	Spock2
0	2,696057601	0,882	0,003	0	Endothelial	Adgrl4
0	2,593441723	0,775	0,034	0	Endothelial	Igfbp7
0	2,583319359	0,889	0,072	0	Endothelial	Ccdc141
0	2,578129707	0,859	0,084	0	Endothelial	Slco1c1
0	2,564936686	0,977	0,592	0	Endothelial	Igf1r
0	2,555934243	0,831	0,007	0	Endothelial	Pltp
0	2,456026994	0,516	0,009	0	Endothelial	Vtn
0	2,447129764	0,911	0,374	0	Endothelial	Cdk8
0	2,429938699	0,943	0,224	0	Endothelial	Utrn
0	2,421492375	0,892	0,023	0	Endothelial	Egfl7
0	2,354999037	0,866	0,027	0	Endothelial	Lef1
0	2,262950975	0,946	0,519	0	Endothelial	Gpcpd1
0	2,244336253	0,713	0,048	0	Endothelial	Tmsb4x
0	2,242180502	0,942	0,244	0	Endothelial	Ptprm

0	2,202917642	0,942	0,516	0	Endothelial	Sgms1
0	2,135635945	0,997	0,858	0	Endothelial	Ptprg
0	2,13103492	0,801	0,004	0	Endothelial	Abcb1a
0	2,100693308	0,681	0,026	0	Endothelial	Sparc
0	2,099880987	0,998	0,903	0	Endothelial	Sptbn1
0	2,096628864	0,791	0,051	0	Endothelial	Slc2a1
0	2,096470152	0,795	0,041	0	Endothelial	Epas1
0	2,093685494	0,79	0,003	0	Endothelial	Cyyr1
0	2,00220929	0,742	0,058	0	Endothelial	Slc7a5
0	1,982523139	0,77	0,053	0	Endothelial	Atp10a
0	1,978568613	0,827	0,088	0	Endothelial	Abcg2
0	1,971434702	0,767	0,004	0	Endothelial	Adgrf5
0	1,96577334	0,64	0,002	0	Endothelial	Itm2a
0	1,939148602	0,708	0,064	0	Endothelial	Actb
0	1,9297688	0,772	0,106	0	Endothelial	Kitl
0	1,897295128	0,613	0,001	0	Endothelial	Ly6c1
0	1,883322718	0,726	0,049	0	Endothelial	Sema3c
0	1,861619706	0,707	0,024	0	Endothelial	Ets1
0	1,849496513	0,765	0,036	0	Endothelial	Fli1
0	1,839159521	0,657	0,003	0	Endothelial	Klf2
0	1,824183661	0,852	0,154	0	Endothelial	Arhgap31
0	1,817807149	0,782	0,193	0	Endothelial	Slc39a10
0	1,794682641	0,88	0,386	0	Endothelial	Tmtc2
0	1,790445225	0,741	0,086	0	Endothelial	Tbc1d4
0	1,789427585	0,668	0,05	0	Endothelial	Rpl13
0	1,756064497	0,671	0,037	0	Endothelial	Ubb
0	1,747814585	0,7	0,028	0	Endothelial	Itga1
0	1,740304184	0,754	0,163	0	Endothelial	Lars2
0	1,725253318	0,78	0,093	0	Endothelial	St3gal6
0	1,724528501	0,7	0,097	0	Endothelial	Jcad
0	1,713049093	0,778	0,134	0	Endothelial	Heg1
0	1,691875773	0,625	0,017	0	Endothelial	Ramp2
0	1,669232228	0,666	0,009	0	Endothelial	Clec2d
0	1,65775646	0,61	0,015	0	Endothelial	Id1
0	1,650394736	0,638	0,102	0	Endothelial	Slc7a1
0	1,646938373	0,834	0,49	0	Endothelial	Slc6a6
0	1,643844894	0,874	0,513	0	Endothelial	Ablim1
0	1,6318099	0,688	0,006	0	Endothelial	Nostrin
0	1,630146652	0,692	0,197	0	Endothelial	Calm1
0	1,619938663	0,836	0,291	0	Endothelial	Hip1
0	1,606827008	0,725	0,165	0	Endothelial	Ly6e
0	1,606759927	0,6	0,039	0	Endothelial	mt-Co1
0	1,603351135	0,726	0,132	0	Endothelial	Cobll1

0	1,602275528	0,707	0,072	0	Endothelial	Arhgap29
0	1,600788264	0,758	0,215	0	Endothelial	Itm2b
0	1,598793379	0,664	0,05	0	Endothelial	Apcdd1
0	1,589947452	0,754	0,252	0	Endothelial	Hsp90ab1
0	1,581591134	0,687	0,007	0	Endothelial	Pecam1
0	1,581056213	0,811	0,356	0	Endothelial	Rundc3b
0	1,574584842	0,723	0,1	0	Endothelial	Tek
0	1,568441773	0,685	0,011	0	Endothelial	Emcn
0	1,566536649	0,661	0,014	0	Endothelial	Cgnl1
0	1,56399994	0,587	0,004	0	Endothelial	Rplp1
0	1,562112915	0,668	0,144	0	Endothelial	Ptma
0	1,552463033	0,622	0,009	0	Endothelial	Ahnak
0	1,54380203	0,638	0,179	0	Endothelial	Tfrc
0	1,531040437	0,747	0,127	0	Endothelial	Abcc4
0	1,52561409	0,663	0,042	0	Endothelial	Tns1
0	1,520837685	0,682	0,078	0	Endothelial	Kank3
0	1,505532713	0,661	0,148	0	Endothelial	Myl6
0	1,490413262	0,666	0,006	0	Endothelial	Eng
0	1,461722397	0,621	0,024	0	Endothelial	Podxl
0	1,458638122	0,616	0,046	0	Endothelial	Id3
0	1,454173123	0,611	0,002	0	Endothelial	Zfp366
0	1,449999036	0,629	0,163	0	Endothelial	Atp1a2
0	1,440783024	0,726	0,153	0	Endothelial	9530026P05Rik
0	1,435376379	0,563	0,021	0	Endothelial	Fau
0	1,432856419	0,621	0,043	0	Endothelial	Fn1
0	1,431252113	0,652	0,016	0	Endothelial	Rbpms
0	1,430057137	0,689	0,095	0	Endothelial	Fth1
0	1,417078208	0,662	0,05	0	Endothelial	Pdgfd
0	1,411358442	0,694	0,112	0	Endothelial	Dach1
0	1,39162658	0,604	0,028	0	Endothelial	Slc9a3r2
0	1,364404605	0,725	0,155	0	Endothelial	Myo10
0	1,359938976	0,673	0,111	0	Endothelial	Itga6
0	1,357075454	0,767	0,263	0	Endothelial	Rbms1
0	1,355720711	0,507	0,001	0	Endothelial	Hspb1
0	1,353201749	0,996	0,929	0	Endothelial	Fry
0	1,338656286	0,659	0,19	0	Endothelial	H3f3b
0	1,337064872	0,776	0,333	0	Endothelial	Col4a3bp
0	1,335312716	0,574	0,108	0	Endothelial	Rps8
0	1,325016287	0,602	0,072	0	Endothelial	Rps21
0	1,314390303	0,545	0,018	0	Endothelial	Nfkbia
0	1,306946816	0,6	0,017	0	Endothelial	Prom1
0	1,292686867	0,735	0,27	0	Endothelial	Abhd2
0	1,287367042	0,523	0,01	0	Endothelial	Rps29

0	1,286123334	0,654	0,19	0	Endothelial	Ctnnb1
0	1,279820276	0,535	0,012	0	Endothelial	Rpl8
0	1,274405782	0,574	0,056	0	Endothelial	Hspa8
0	1,272734349	0,614	0,02	0	Endothelial	Prkch
0	1,271261711	0,757	0,272	0	Endothelial	Sparcl1
0	1,264044275	0,533	0,018	0	Endothelial	mt-Co3
0	1,262375908	0,538	0,015	0	Endothelial	Tpt1
0	1,256504852	0,619	0,131	0	Endothelial	Ltbp4
0	1,253684819	0,747	0,263	0	Endothelial	4931406P16Rik
0	1,251799397	0,995	0,888	0	Endothelial	Tjp1
0	1,250079941	0,581	0,008	0	Endothelial	Erg
0	1,247463461	0,587	0,131	0	Endothelial	Ppia
0	1,244673464	0,565	0,107	0	Endothelial	Car4
0	1,241910136	0,648	0,228	0	Endothelial	Col4a1
0	1,234279613	0,624	0,104	0	Endothelial	St8sia4
0	1,229328715	0,543	0,009	0	Endothelial	Vwa1
0	1,228980991	0,625	0,159	0	Endothelial	Eif1
0	1,212779679	0,631	0,06	0	Endothelial	Rasgrp3
0	1,206232524	0,526	0,021	0	Endothelial	Itih5
0	1,203919166	0,574	0,069	0	Endothelial	Pdgfb
0	1,200767981	0,608	0,062	0	Endothelial	Wwtr1
0	1,171694986	0,511	0,003	0	Endothelial	Kdr
0	1,171242817	0,577	0,01	0	Endothelial	Fgd5
0	1,170023203	0,53	0,024	0	Endothelial	H2-D1
0	1,168179012	0,53	0,021	0	Endothelial	Actg1
0	1,16600751	0,734	0,146	0	Endothelial	Cst3
0	1,161939094	0,535	0,039	0	Endothelial	Crip2
0	1,161077064	0,621	0,211	0	Endothelial	Eef1a1
0	1,160765728	0,553	0,002	0	Endothelial	Esam
0	1,15083757	0,594	0,09	0	Endothelial	Eogt
0	1,148050331	0,624	0,189	0	Endothelial	H3f3a
0	1,147722681	0,505	0,02	0	Endothelial	Rpl38
0	1,146788767	0,546	0,047	0	Endothelial	Dock6
0	1,143711576	0,558	0,056	0	Endothelial	Ubc
0	1,139079734	0,713	0,232	0	Endothelial	Sema6a
0	1,137921936	0,534	0,005	0	Endothelial	Paqr5
0	1,137454949	0,633	0,115	0	Endothelial	Clic4
0	1,130499079	0,664	0,235	0	Endothelial	Extl3
0	1,129224778	0,552	0,015	0	Endothelial	Fzd6
0	1,1252742	0,626	0,21	0	Endothelial	Palmd
0	1,117724328	0,551	0,007	0	Endothelial	Adgre5
0	1,112649811	0,573	0,078	0	Endothelial	Mtus1
0	1,11223495	0,616	0,137	0	Endothelial	Polk

0	1,096086742	0,563	0,129	0	Endothelial	Rpl27a
0	1,074426937	0,571	0,116	0	Endothelial	Myo1b
0	1,07351741	0,51	0,051	0	Endothelial	Rpl41
0	1,070560365	0,507	0,006	0	Endothelial	Slc22a8
0	1,065331669	0,523	0,019	0	Endothelial	Hspa12b
0	1,059113357	0,529	0,095	0	Endothelial	Rpl37a
0	1,056464423	0,588	0,111	0	Endothelial	Elk3
0	1,05417015	0,551	0,094	0	Endothelial	Arl4a
0	1,049811838	0,512	0,024	0	Endothelial	Rps14
0	1,049695812	0,505	0,028	0	Endothelial	Rpl18
0	1,048396079	0,624	0,131	0	Endothelial	Cd81
0	1,040769097	0,503	0,003	0	Endothelial	Cdh5
0	1,037240498	0,564	0,061	0	Endothelial	Afap1l1
0	1,018249973	0,518	0,071	0	Endothelial	Gnai2
0	1,017034603	0,535	0,062	0	Endothelial	Parvb
0	1,003033562	0,535	0,089	0	Endothelial	Mt1
0	0,99912615	0,59	0,177	0	Endothelial	Serinc3
0	0,991609714	0,562	0,103	0	Endothelial	Itpkb
0	0,989520559	0,53	0,045	0	Endothelial	Tgfbr2
0	0,979132935	0,622	0,184	0	Endothelial	Mpzl1
0	0,975096067	0,563	0,094	0	Endothelial	Nxn
0	0,972519224	0,515	0,075	0	Endothelial	Nxpe2
0	0,971420466	0,519	0,05	0	Endothelial	Ocln
0	0,946000339	0,581	0,141	0	Endothelial	Ctnna1
0	0,942906516	0,504	0,064	0	Endothelial	Tuba1a
0	0,934821965	0,528	0,067	0	Endothelial	Rgs12
0	0,933335169	0,538	0,107	0	Endothelial	Sema7a
0	0,927210464	0,754	0,177	0	Endothelial	Apoe
0	0,927126556	0,514	0,062	0	Endothelial	S1pr1
0	0,917680451	0,507	0,053	0	Endothelial	Jam2
0	0,908422052	0,502	0,054	0	Endothelial	Zfp36l1
0	0,900803729	0,515	0,097	0	Endothelial	Rhobtb1
0	0,875769153	0,535	0,119	0	Endothelial	Ifnar2
0	0,859950828	0,515	0,113	0	Endothelial	Maoa
0	0,851450687	0,534	0,127	0	Endothelial	Ppfibp1
0	0,802633761	0,529	0,128	0	Endothelial	Myo1e
7,13E-306	0,989074707	0,788	0,299	1,92E-301	Endothelial	Prex2
7,32E-306	0,89598443	0,577	0,168	1,97E-301	Endothelial	Wasf2
7,45E-305	0,831856309	0,549	0,145	2,01E-300	Endothelial	Pdlim5
1,48E-301	1,450257107	0,791	0,366	3,98E-297	Endothelial	Ptn
1,60E-300	1,128508755	0,985	0,883	4,30E-296	Endothelial	Foxp1
3,64E-293	1,039050357	0,669	0,237	9,79E-289	Endothelial	Mcc
1,59E-288	0,982302189	0,587	0,173	4,29E-284	Endothelial	Chn2

3,22E-288	0,947674447	0,633	0,2	8,67E-284	Endothelial	Pard3b
4,73E-283	0,836199482	0,638	0,189	1,27E-278	Endothelial	Gab1
2,23E-280	1,284775506	0,829	0,547	6,01E-276	Endothelial	Esyt2
7,11E-277	0,830185545	0,525	0,154	1,92E-272	Endothelial	Ece1
1,46E-275	1,286381857	0,608	0,213	3,92E-271	Endothelial	Cald1
1,38E-273	0,996547929	0,669	0,238	3,71E-269	Endothelial	Plcb4
3,08E-272	1,288926279	0,78	0,449	8,29E-268	Endothelial	Gnas
1,99E-270	1,095022081	0,707	0,316	5,35E-266	Endothelial	Nxpe4
3,07E-267	0,810372106	0,53	0,155	8,27E-263	Endothelial	Golim4
2,29E-261	0,91997154	1	0,966	6,17E-257	Endothelial	Macf1
8,31E-259	1,150351661	0,762	0,418	2,24E-254	Endothelial	Bmpr2
2,86E-258	1,006691377	0,631	0,247	7,70E-254	Endothelial	Dab2ip
3,65E-255	0,918360154	0,596	0,208	9,84E-251	Endothelial	Ldlrad3
5,20E-253	0,737976636	0,511	0,144	1,40E-248	Endothelial	Rin2
8,62E-253	0,896675781	0,693	0,249	2,32E-248	Endothelial	Plekhg1
3,29E-251	1,217731476	0,811	0,403	8,85E-247	Endothelial	Pitpnc1
8,67E-244	1,073113805	0,894	0,653	2,34E-239	Endothelial	Apbb2
1,63E-243	0,714864598	0,532	0,15	4,38E-239	Endothelial	Gatm
2,62E-243	0,907775206	0,987	0,882	7,05E-239	Endothelial	App
3,36E-243	0,535376563	0,745	0,244	9,06E-239	Endothelial	Tshz2
1,93E-237	1,161800471	0,956	0,844	5,19E-233	Endothelial	Camk1d
4,17E-232	1,020671017	0,921	0,659	1,12E-227	Endothelial	Klf12
1,96E-224	0,694655038	0,521	0,155	5,29E-220	Endothelial	Itpr2
1,12E-222	0,914840519	0,912	0,686	3,01E-218	Endothelial	Srgap2
6,64E-221	1,056131672	0,722	0,399	1,79E-216	Endothelial	Luzp1
5,95E-220	0,739885433	0,668	0,24	1,60E-215	Endothelial	Dock1
5,27E-213	0,873430575	0,572	0,222	1,42E-208	Endothelial	Rhoa
4,09E-210	0,959222467	0,622	0,275	1,10E-205	Endothelial	Myh9
1,42E-206	1,046503567	0,711	0,386	3,83E-202	Endothelial	Pakap.1
4,98E-206	0,79481536	0,514	0,183	1,34E-201	Endothelial	Oaz1
5,35E-203	1,593599157	0,698	0,378	1,44E-198	Endothelial	Dlc1
5,34E-201	0,811854047	0,656	0,279	1,44E-196	Endothelial	Myo1d
2,50E-194	0,859966315	0,612	0,247	6,73E-190	Endothelial	Fbxl7
9,41E-191	0,775339031	0,56	0,225	2,54E-186	Endothelial	Snrk
1,76E-189	0,829343417	0,678	0,306	4,74E-185	Endothelial	Stard13
1,03E-183	0,804208218	0,568	0,227	2,78E-179	Endothelial	Adipor2
1,43E-182	1,129877402	0,705	0,463	3,85E-178	Endothelial	Tsc22d1
3,51E-181	0,916558853	0,67	0,333	9,45E-177	Endothelial	Efr3b
7,97E-171	0,847710511	0,584	0,274	2,15E-166	Endothelial	Slc3a2
8,19E-170	0,790732098	0,937	0,671	2,20E-165	Endothelial	Dnm3
8,76E-169	0,920843478	0,708	0,419	2,36E-164	Endothelial	Insr
1,10E-166	0,90858262	0,849	0,659	2,98E-162	Endothelial	Fut8
1,46E-164	0,844700431	0,598	0,288	3,92E-160	Endothelial	Col4a2

1,28E-162	0,763216626	0,585	0,266	3,46E-158	Endothelial	Klf13
7,88E-158	0,780372513	0,841	0,636	2,12E-153	Endothelial	Arhgef12
1,09E-156	0,806226634	0,659	0,346	2,93E-152	Endothelial	Akap13
1,06E-155	0,590134858	0,868	0,576	2,86E-151	Endothelial	Msi2
2,57E-155	0,70171028	0,612	0,274	6,94E-151	Endothelial	Ttc28
4,69E-155	0,810999999	0,58	0,28	1,26E-150	Endothelial	Itgb1
7,05E-154	0,810145808	0,692	0,407	1,90E-149	Endothelial	Fermt2
1,97E-152	0,683230529	0,521	0,217	5,29E-148	Endothelial	Gpd2
1,13E-149	0,801385201	0,534	0,239	3,06E-145	Endothelial	Usp53
2,41E-146	0,666628396	0,534	0,229	6,50E-142	Endothelial	Klhl5
5,49E-144	0,765224389	0,586	0,3	1,48E-139	Endothelial	Sec62
6,11E-142	0,717959655	0,575	0,283	1,65E-137	Endothelial	Iqgap1
2,03E-135	0,711482094	0,567	0,274	5,46E-131	Endothelial	Bcl2l1
3,52E-135	0,791379975	0,785	0,572	9,48E-131	Endothelial	Fryl
9,89E-134	0,684060244	0,597	0,287	2,67E-129	Endothelial	Adgrl2
1,02E-130	0,614481741	0,945	0,782	2,74E-126	Endothelial	Tcf12
4,62E-129	0,637028904	0,512	0,229	1,24E-124	Endothelial	Rdx
1,29E-126	0,593659117	0,511	0,212	3,48E-122	Endothelial	St6gal1
3,99E-120	0,737675544	0,672	0,424	1,08E-115	Endothelial	Lrch1
4,12E-120	0,631920643	0,507	0,241	1,11E-115	Endothelial	Ppp1r2
2,95E-119	0,639137508	0,511	0,245	7,93E-115	Endothelial	Cyth3
3,73E-119	0,720407541	0,563	0,309	1,00E-114	Endothelial	Cnot6l
8,65E-119	0,661243319	0,531	0,26	2,33E-114	Endothelial	Wwc2
2,70E-115	0,656826685	0,566	0,305	7,26E-111	Endothelial	Ywhae
1,76E-112	0,679081312	0,704	0,483	4,75E-108	Endothelial	Fcho2
7,51E-110	0,578281377	0,514	0,237	2,02E-105	Endothelial	Mtss1
2,11E-109	0,625618957	0,935	0,81	5,70E-105	Endothelial	Dock9
3,67E-107	0,665223032	0,515	0,266	9,89E-103	Endothelial	Eif4g2
8,12E-107	0,715826286	0,571	0,324	2,19E-102	Endothelial	Lims1
3,67E-103	0,647323719	0,503	0,257	9,89E-99	Endothelial	Hmgb1
1,06E-102	0,719963297	0,671	0,465	2,85E-98	Endothelial	Cd2ap
3,13E-100	0,52659661	0,54	0,277	8,42E-96	Endothelial	Psap
4,68E-100	0,632067664	0,931	0,856	1,26E-95	Endothelial	Tspan5
9,47E-100	0,624133277	0,785	0,602	2,55E-95	Endothelial	Map4
2,60E-99	0,554743737	0,509	0,257	7,00E-95	Endothelial	Git2
5,05E-99	0,66438908	0,642	0,418	1,36E-94	Endothelial	Nbeal1
5,78E-99	0,666021599	0,656	0,443	1,56E-94	Endothelial	Kif5b
3,92E-98	0,588955835	0,623	0,349	1,06E-93	Endothelial	Sash1
1,96E-97	0,67302127	0,691	0,519	5,28E-93	Endothelial	Pcmtd1
2,15E-97	0,630871209	0,799	0,66	5,78E-93	Endothelial	Mprip
6,06E-97	0,645537239	0,513	0,277	1,63E-92	Endothelial	Lamc1
1,32E-95	0,603454765	0,567	0,329	3,55E-91	Endothelial	Adam10
2,52E-94	0,537031264	0,932	0,839	6,79E-90	Endothelial	Rapgef2

8,31E-94	0,56544312	0,511	0,273	2,24E-89	Endothelial	Snx3
1,35E-93	0,592453657	0,537	0,297	3,63E-89	Endothelial	Actn4
2,97E-90	0,614824976	0,524	0,29	7,99E-86	Endothelial	Slc16a2
2,58E-89	0,64484274	0,585	0,383	6,94E-85	Endothelial	Cdc42
2,80E-89	0,577719324	0,554	0,302	7,55E-85	Endothelial	Etv6
6,91E-89	0,566713245	0,792	0,631	1,86E-84	Endothelial	Nedd4
3,76E-86	0,614909744	0,634	0,422	1,01E-81	Endothelial	Klf7
4,96E-86	0,581903799	0,569	0,341	1,34E-81	Endothelial	Ctnnd1
3,24E-85	0,590976353	0,567	0,345	8,74E-81	Endothelial	Agrn
1,44E-84	0,654080456	0,545	0,34	3,88E-80	Endothelial	Rpl6
1,97E-84	0,601147515	0,648	0,436	5,29E-80	Endothelial	Susd6
2,09E-83	0,589099954	0,71	0,502	5,62E-79	Endothelial	Pard3
2,48E-82	0,622694712	0,65	0,472	6,68E-78	Endothelial	Aplp2
3,22E-81	0,566582956	0,524	0,307	8,68E-77	Endothelial	Ddx6
1,05E-80	0,601678075	0,514	0,295	2,83E-76	Endothelial	Pls3
1,29E-80	0,595479508	0,6	0,391	3,48E-76	Endothelial	Btbd7
1,23E-78	0,586623724	0,636	0,456	3,32E-74	Endothelial	Gnb1
1,38E-77	0,516239196	0,505	0,28	3,72E-73	Endothelial	Pak2
2,45E-75	0,550705491	0,605	0,35	6,59E-71	Endothelial	Pde7b
2,02E-74	0,546453866	0,532	0,33	5,43E-70	Endothelial	Agfg1
3,11E-73	0,516315419	0,722	0,577	8,38E-69	Endothelial	Mef2a
3,43E-72	0,542347203	0,559	0,361	9,25E-68	Endothelial	Znrf1
9,61E-71	0,628867311	0,933	0,87	2,59E-66	Endothelial	Arl15
2,81E-68	0,521261857	0,636	0,461	7,58E-64	Endothelial	Mbnl1
6,90E-66	0,511295717	0,898	0,817	1,86E-61	Endothelial	Rapgef4
1,81E-64	0,520787903	0,583	0,402	4,86E-60	Endothelial	Hectd1
6,82E-62	0,523404074	0,588	0,42	1,84E-57	Endothelial	Mcf2l
1,12E-58	0,591060566	0,695	0,596	3,03E-54	Endothelial	Pten
4,77E-58	0,523919398	0,973	0,912	1,29E-53	Endothelial	Prkg1
4,46E-55	0,502049119	0,818	0,705	1,20E-50	Endothelial	Rapgef5
3,23E-25	0,64345728	0,642	0,611	8,69E-21	Endothelial	Pde8b

0	3,34150895	0,919	0,038	0	Pericyte	Atp13a5
0	2,817512741	0,95	0,033	0	Pericyte	Ebf1
0	2,427758307	0,791	0,011	0	Pericyte	Vtn
0	2,378232106	0,947	0,165	0	Pericyte	Atp1a2
0	2,07512444	0,827	0,024	0	Pericyte	Slc6a20a
0	2,041060437	0,911	0,215	0	Pericyte	Cald1
0	1,993776024	0,864	0,049	0	Pericyte	Pdgfrb
0	1,782972588	0,799	0,089	0	Pericyte	Plce1
0	1,702820571	0,724	0,033	0	Pericyte	Itga1
0	1,695462494	0,713	0,033	0	Pericyte	Notch3
0	1,658271359	0,699	0,011	0	Pericyte	Morrbid

0	1,631934347	0,688	0,079	0	Pericyte	Trpc3
0	1,597947081	0,677	0,041	0	Pericyte	Colec12
0	1,59432469	0,682	0,01	0	Pericyte	Slc38a11
0	1,594007245	0,621	0,005	0	Pericyte	Abcc9
0	1,552359485	0,694	0,005	0	Pericyte	Tbx3os1
0	1,528364797	0,682	0,056	0	Pericyte	Phldb2
0	1,504515607	0,727	0,118	0	Pericyte	Myo1b
0	1,502507372	0,685	0,098	0	Pericyte	Arhgap6
0	1,481957169	0,696	0,031	0	Pericyte	Adap2
0	1,477374296	0,688	0,05	0	Pericyte	Dock6
0	1,4758308	0,657	0,02	0	Pericyte	Rbpms
0	1,450908074	0,599	0,039	0	Pericyte	Igfbp7
0	1,43849224	0,643	0,006	0	Pericyte	Carmn
0	1,339064296	0,538	0,009	0	Pericyte	Rgs5
0	1,178924232	0,554	0,012	0	Pericyte	Egflam
0	1,073298305	0,535	0,046	0	Pericyte	Cped1
8,92E-299	1,131210157	0,579	0,076	2,40E-294	Pericyte	Arhgap29
2,52E-283	1,066394873	0,518	0,062	6,78E-279	Pericyte	Sntb1
7,15E-275	1,481436164	0,733	0,142	1,93E-270	Pericyte	Arhgap42
3,93E-264	2,901401637	0,978	0,379	1,06E-259	Pericyte	Dlc1
3,04E-222	1,628317636	0,85	0,229	8,19E-218	Pericyte	Utrn
1,30E-214	1,277221258	0,674	0,136	3,50E-210	Pericyte	Cobll1
8,30E-211	0,936859375	0,518	0,079	2,23E-206	Pericyte	Ccdc141
3,86E-195	1,362868018	0,691	0,157	1,04E-190	Pericyte	9530026P05Rik
4,44E-188	1,908743223	0,95	0,405	1,19E-183	Pericyte	Pitpnc1
5,10E-170	1,927944131	0,964	0,61	1,37E-165	Pericyte	Pde8b
3,26E-163	1,532354389	0,735	0,235	8,78E-159	Pericyte	Airn
4,07E-158	1,024744426	0,51	0,096	1,10E-153	Pericyte	AY036118
5,66E-157	1,723481947	0,942	0,464	1,52E-152	Pericyte	Plxdc2
2,67E-150	1,213406094	0,738	0,201	7,20E-146	Pericyte	Lama2
9,97E-141	1,687869267	0,992	0,651	2,69E-136	Pericyte	Plcl1
1,99E-140	1,016056214	0,621	0,157	5,36E-136	Pericyte	Itpr2
6,52E-128	1,483782816	0,844	0,368	1,76E-123	Pericyte	Ptn
1,11E-119	0,983274259	0,66	0,201	2,99E-115	Pericyte	04. Sep
3,93E-119	0,904899572	0,507	0,123	1,06E-114	Pericyte	Svil
2,39E-111	0,852938144	0,557	0,15	6,43E-107	Pericyte	Eps8
1,79E-106	0,981970187	0,632	0,192	4,83E-102	Pericyte	Gab1
4,96E-104	1,100700567	0,607	0,203	1,34E-99	Pericyte	Lhfp
6,59E-101	1,126590304	0,682	0,266	1,78E-96	Pericyte	Rbms1
1,82E-99	1,238610095	0,953	0,562	4,90E-95	Pericyte	Grm3
2,52E-99	0,837291208	0,571	0,169	6,79E-95	Pericyte	Epb41l2
3,01E-95	1,284508102	0,933	0,52	8,10E-91	Pericyte	Gm42418
5,50E-90	1,091218939	0,975	0,667	1,48E-85	Pericyte	Ptprk

9,34E-90	0,958818066	0,596	0,203	2,52E-85	Pericyte	Pard3b
2,67E-89	1,132481738	0,805	0,402	7,20E-85	Pericyte	Inpp4b
4,09E-89	1,046996291	0,747	0,326	1,10E-84	Pericyte	Cmss1
9,91E-83	0,995734432	0,864	0,596	2,67E-78	Pericyte	Pten
9,25E-82	0,971517575	0,903	0,654	2,49E-77	Pericyte	Apbb2
3,49E-81	0,780008414	1	1	9,39E-77	Pericyte	Malat1
2,72E-80	0,880379934	0,727	0,311	7,31E-76	Pericyte	Rbms3
1,55E-79	0,905859076	0,992	0,863	4,17E-75	Pericyte	Mast4
5,98E-79	1,085698794	0,986	0,929	1,61E-74	Pericyte	Fry
4,45E-77	1,183280109	0,836	0,42	1,20E-72	Pericyte	Gpc6
3,55E-72	0,990930857	0,616	0,278	9,57E-68	Pericyte	Lamc1
4,45E-69	1,285148113	0,986	0,912	1,20E-64	Pericyte	Prkg1
9,19E-68	0,90072087	0,969	0,768	2,47E-63	Pericyte	Diaph2
1,06E-65	0,920419481	0,646	0,325	2,87E-61	Pericyte	Lims1
1,92E-63	0,837288832	0,994	0,916	5,16E-59	Pericyte	Rora
2,28E-63	0,827150202	0,936	0,557	6,15E-59	Pericyte	Maml2
2,69E-62	0,843371175	0,577	0,258	7,24E-58	Pericyte	St5
4,84E-62	1,082366458	0,908	0,705	1,31E-57	Pericyte	Rapgef5
4,51E-57	0,648176172	0,507	0,185	1,21E-52	Pericyte	Daam2
4,92E-56	0,696988889	0,978	0,903	1,33E-51	Pericyte	Sptbn1
2,06E-55	0,841295622	0,627	0,315	5,55E-51	Pericyte	Slc12a2
2,68E-51	0,79764528	0,889	0,644	7,23E-47	Pericyte	Zeb1
1,42E-48	0,738769569	0,805	0,607	3,83E-44	Pericyte	Zfand3
1,86E-48	0,820163336	0,696	0,393	5,02E-44	Pericyte	Pdzd2
2,39E-47	0,830774733	0,657	0,403	6,43E-43	Pericyte	Ctdspl
7,72E-45	0,802417709	0,532	0,275	2,08E-40	Pericyte	Gucy1b1
8,41E-43	0,597862254	0,978	0,728	2,27E-38	Pericyte	Nfia
3,41E-42	0,63780066	0,872	0,569	9,18E-38	Pericyte	Sox5
2,78E-41	0,827074321	0,733	0,496	7,48E-37	Pericyte	Pid1
9,21E-41	0,644688349	0,744	0,481	2,48E-36	Pericyte	07. Sep
1,94E-40	0,680481426	0,585	0,309	5,24E-36	Pericyte	Stard13
3,01E-40	0,700422542	0,524	0,278	8,10E-36	Pericyte	Myh9
7,24E-38	0,646506641	0,955	0,884	1,95E-33	Pericyte	Foxp1
3,44E-34	0,622518769	0,677	0,442	9,27E-30	Pericyte	Farp1
5,18E-34	0,70658948	0,501	0,27	1,40E-29	Pericyte	E130308A19Rik
4,95E-33	0,508723258	0,992	0,894	1,33E-28	Pericyte	Ddx5
1,58E-32	0,667507712	0,696	0,504	4,27E-28	Pericyte	Pard3
1,64E-32	0,534248681	0,95	0,783	4,42E-28	Pericyte	Tcf12
4,66E-32	0,715374517	0,568	0,36	1,26E-27	Pericyte	11. Sep
4,71E-32	0,514592066	0,989	0,812	1,27E-27	Pericyte	Zbtb20
1,93E-31	0,622255201	0,593	0,388	5,19E-27	Pericyte	Pakap.1
2,38E-30	0,680551166	0,63	0,437	6,42E-26	Pericyte	Slco3a1
5,38E-30	0,731005496	0,596	0,378	1,45E-25	Pericyte	Cdk8

2,22E-29	0,554585564	0,816	0,66	5,98E-25	Pericyte	Mprip
1,40E-28	0,512965291	0,891	0,709	3,78E-24	Pericyte	Specc1
3,22E-28	0,610932113	0,616	0,419	8,66E-24	Pericyte	Nbeal1
5,01E-26	0,555378472	0,721	0,555	1,35E-21	Pericyte	Dleu2
1,74E-25	0,531275618	0,535	0,322	4,69E-21	Pericyte	Pik3r1
6,93E-24	0,50330144	0,596	0,362	1,87E-19	Pericyte	Rgs6
1,26E-23	0,586273476	0,501	0,292	3,39E-19	Pericyte	Ror1
4,52E-23	0,595470571	0,588	0,42	1,22E-18	Pericyte	Carmil1
9,23E-23	0,543331734	0,513	0,322	2,49E-18	Pericyte	Tbc1d1
7,36E-21	0,555548509	0,649	0,516	1,98E-16	Pericyte	Daam1
2,20E-20	0,558761024	0,54	0,352	5,93E-16	Pericyte	Pde7b
3,77E-19	0,545295136	0,538	0,387	1,01E-14	Pericyte	Lpp
1,31E-17	0,598374213	0,585	0,492	3,53E-13	Pericyte	Gmds
3,80E-17	0,517962601	0,822	0,718	1,02E-12	Pericyte	Gucy1a2
1,16E-15	0,522330841	0,568	0,452	3,13E-11	Pericyte	Adk
0	5,286265831	0,906	0,128	0	ependymal	Htr2c
0	4,535243571	0,908	0,012	0	ependymal	Ttr
0	4,002256355	0,918	0,261	0	ependymal	Enpp2
0	3,908045903	0,98	0,681	0	ependymal	Trpm3
0	3,253031525	0,903	0,009	0	ependymal	Otx2os1
0	2,892439216	0,952	0,018	0	ependymal	Spag16
0	2,843325012	0,887	0,061	0	ependymal	Sulf1
0	2,78498615	0,934	0,516	0	ependymal	Stk39
0	2,662451527	0,851	0,018	0	ependymal	Gmnc
0	2,399553552	0,852	0,068	0	ependymal	Vat1l
0	2,389536554	0,854	0,018	0	ependymal	Rbm47
0	2,351219265	0,818	0,019	0	ependymal	Col8a1
0	2,338227877	0,869	0,042	0	ependymal	Col9a3
0	2,281112205	0,939	0,387	0	ependymal	Esrrg
0	2,206796868	0,925	0,455	0	ependymal	Sh3d19
0	2,1759452	0,894	0,086	0	ependymal	Prdm16
0	2,098491526	0,786	0,04	0	ependymal	Prlr
0	2,065144043	0,887	0,093	0	ependymal	Lrriq1
0	2,040549759	0,868	0,248	0	ependymal	Fras1
0	2,009325024	0,831	0,002	0	ependymal	Tmem72
0	1,986233257	0,859	0,322	0	ependymal	Nhsl2
0	1,948046135	0,925	0,305	0	ependymal	Stard13
0	1,832166383	0,869	0,254	0	ependymal	Cab39l
0	1,802235293	0,844	0,203	0	ependymal	Tbc1d9
0	1,718514907	0,782	0,012	0	ependymal	Dnah6
0	1,693541706	0,845	0,288	0	ependymal	Slc16a2
0	1,683916047	0,811	0,063	0	ependymal	Spef2

0	1,678838815	0,869	0,131	0	ependymal	Gm4876
0	1,653393289	0,749	0,006	0	ependymal	Clic6
0	1,639355104	0,697	0,014	0	ependymal	Mecom
0	1,594819765	0,738	0,035	0	ependymal	Kl
0	1,575571538	0,769	0,089	0	ependymal	Cfap44
0	1,572768312	0,839	0,34	0	ependymal	Atp2b3
0	1,569403277	0,759	0,029	0	ependymal	Frem1
0	1,557585664	0,832	0,184	0	ependymal	Cfap69
0	1,546519668	0,754	0,004	0	ependymal	Drc7
0	1,542527834	0,703	0,081	0	ependymal	Strip2
0	1,53068582	0,83	0,093	0	ependymal	Nxn
0	1,526872905	0,989	0,576	0	ependymal	Msi2
0	1,509146908	0,792	0,06	0	ependymal	Dnah7b
0	1,505908043	0,931	0,317	0	ependymal	Glis3
0	1,505191181	0,731	0,051	0	ependymal	Pdgfd
0	1,497900912	0,776	0,134	0	ependymal	Mid1
0	1,493461837	0,792	0,165	0	ependymal	Car12
0	1,461285988	0,785	0,062	0	ependymal	Shroom3
0	1,457705758	0,806	0,163	0	ependymal	Wdr78
0	1,453222099	0,776	0,209	0	ependymal	L3mbtl3
0	1,453052386	0,766	0,051	0	ependymal	Adgrv1
0	1,448629232	0,654	0,028	0	ependymal	Fap
0	1,434980507	0,776	0,116	0	ependymal	Notch2
0	1,433290332	0,811	0,201	0	ependymal	Rmst
0	1,432280289	0,725	0,051	0	ependymal	Fhad1
0	1,398566171	0,765	0,075	0	ependymal	Ccdc141
0	1,386768371	0,67	0,026	0	ependymal	Bmp6
0	1,364442308	0,666	0,04	0	ependymal	Ccdc162
0	1,354623738	0,701	0,06	0	ependymal	Sntb1
0	1,32709453	0,631	0,006	0	ependymal	Dnah11
0	1,292141069	0,652	0,011	0	ependymal	Gm29266
0	1,272579429	0,656	0,002	0	ependymal	Slc4a5
0	1,271124381	0,724	0,013	0	ependymal	Ccdc114
0	1,251899559	0,701	0,094	0	ependymal	Vwa3a
0	1,241070549	0,803	0,2	0	ependymal	Pard3b
0	1,237537176	0,721	0,074	0	ependymal	Slc39a12
0	1,233840417	0,638	0,064	0	ependymal	Cfap47
0	1,223095253	0,746	0,116	0	ependymal	Syne2
0	1,201331222	0,775	0,239	0	ependymal	Ccdc39
0	1,19504849	0,566	0,023	0	ependymal	Cfap43
0	1,19448444	0,677	0,064	0	ependymal	Gulp1
0	1,193127521	0,721	0,112	0	ependymal	Zfp521
0	1,190313094	0,625	0,002	0	ependymal	Lmx1a

0	1,186055487	0,644	0,115	0	ependymal	Spag6l
0	1,181309995	0,637	0,032	0	ependymal	Baiap2l1
0	1,181304913	0,79	0,168	0	ependymal	Neat1
0	1,167455637	0,628	0,032	0	ependymal	Cfap65
0	1,159842562	0,593	0,014	0	ependymal	Ak9
0	1,154850002	0,713	0,108	0	ependymal	Ppfibp2
0	1,149483607	0,607	0,011	0	ependymal	Mdfic
0	1,144454223	0,606	0,016	0	ependymal	Cgnl1
0	1,144204687	0,594	0,023	0	ependymal	Slc6a20a
0	1,142859143	0,63	0,002	0	ependymal	Otx2
0	1,1416262	0,718	0,189	0	ependymal	Phactr2
0	1,139655576	0,606	0,044	0	ependymal	Kcnj13
0	1,136447002	0,638	0,001	0	ependymal	Folr1
0	1,132231899	0,6	0,02	0	ependymal	Abca4
0	1,119480894	0,669	0,087	0	ependymal	Slco1c1
0	1,112358059	0,677	0,13	0	ependymal	Armc2
0	1,111825222	0,613	0,007	0	ependymal	Slc13a4
0	1,104536949	0,661	0,073	0	ependymal	Psd2
0	1,103628055	0,61	0,066	0	ependymal	Zbbx
0	1,097087336	0,655	0,095	0	ependymal	Mpp7
0	1,081574897	0,573	0,001	0	ependymal	F5
0	1,080414117	0,599	0,022	0	ependymal	Spata17
0	1,076563802	0,599	0,024	0	ependymal	Ezr
0	1,070411603	0,761	0,218	0	ependymal	Clybl
0	1,067481993	0,566	0,019	0	ependymal	Acss3
0	1,064620287	0,601	0,065	0	ependymal	Ap1s2
0	1,057306226	0,566	0,004	0	ependymal	Col4a3
0	1,053435622	0,617	0,076	0	ependymal	Whrn
0	1,053097674	0,632	0,15	0	ependymal	Gm26542
0	1,043418565	0,589	0,034	0	ependymal	Dab2
0	1,037989635	0,531	0,01	0	ependymal	Oca2
0	1,037180108	0,592	0,031	0	ependymal	Lef1
0	1,034580884	0,642	0,106	0	ependymal	Cpq
0	1,0159099	0,569	0,013	0	ependymal	Col4a4
0	0,995501005	0,544	0,063	0	ependymal	4930511M06Rik
0	0,994585743	0,555	0,005	0	ependymal	Trpv4
0	0,986005864	0,665	0,09	0	ependymal	Cpne2
0	0,97134311	0,632	0,084	0	ependymal	Prkcq
0	0,966469677	0,575	0,047	0	ependymal	Iqub
0	0,961413763	0,618	0,119	0	ependymal	Cfap74
0	0,948297074	0,625	0,088	0	ependymal	Plce1
0	0,944312089	0,52	0,024	0	ependymal	9330185C12Rik
0	0,942589748	0,604	0,104	0	ependymal	Itpkb

0	0,936975699	0,589	0,083	0	ependymal	Kif9
0	0,936427271	0,558	0,009	0	ependymal	Gm16201
0	0,926175908	0,632	0,106	0	ependymal	Elovl7
0	0,91746018	0,538	0,015	0	ependymal	Slco1a4
0	0,895082878	0,53	0,021	0	ependymal	Rdh5
0	0,885539034	0,528	0,03	0	ependymal	C330002G04Rik
0	0,877331884	0,537	0,02	0	ependymal	Lrrc23
0	0,876569111	0,538	0,045	0	ependymal	Slc4a2
0	0,875200845	0,532	0,077	0	ependymal	Parva
0	0,862636873	0,548	0,075	0	ependymal	Efcab1
0	0,853780046	0,531	0,102	0	ependymal	Cul4b
0	0,831732166	0,548	0,068	0	ependymal	10. Sep
0	0,829804687	0,508	0,032	0	ependymal	Bad
0	0,819098157	0,503	0,047	0	ependymal	Arl6ip1
0	0,8153666	0,545	0,098	0	ependymal	Arsg
0	0,811138921	0,544	0,1	0	ependymal	2810403D21Rik
0	0,802685686	0,513	0,047	0	ependymal	Tgfbr2
0	0,777624437	0,527	0,064	0	ependymal	Nme5
0	0,771561145	0,531	0,074	0	ependymal	Spata13
0	0,753482976	0,589	0,103	0	ependymal	Kank1
0	0,704783393	0,582	0,113	0	ependymal	Nwd1
6,16E-307	1,127226696	0,758	0,235	1,66E-302	ependymal	Stxbp4
1,47E-294	0,720576606	0,515	0,104	3,95E-290	ependymal	Pdgfa
6,42E-293	1,142149976	0,61	0,151	1,73E-288	ependymal	Cfap54
4,45E-290	1,91794717	0,951	0,809	1,20E-285	ependymal	Slc4a10
5,75E-287	0,826466795	0,601	0,137	1,55E-282	ependymal	Heg1
1,36E-283	0,818827842	0,597	0,141	3,66E-279	ependymal	Arhgap42
1,87E-282	1,174570633	0,759	0,265	5,05E-278	ependymal	Pcbp3
6,40E-281	0,897349002	0,613	0,15	1,72E-276	ependymal	Antxr1
9,15E-281	1,189846105	0,872	0,351	2,47E-276	ependymal	Tead1
5,48E-279	1,163071655	0,755	0,253	1,48E-274	ependymal	Ctnnal1
2,41E-277	1,350711458	0,817	0,327	6,48E-273	ependymal	Mitf
8,14E-274	1,527959629	0,992	0,777	2,19E-269	ependymal	Agbl4
9,51E-273	1,158483842	0,777	0,264	2,56E-268	ependymal	Rbms1
1,83E-271	1,278417749	0,9	0,39	4,92E-267	ependymal	Pdzd2
2,92E-271	1,415158741	0,876	0,462	7,85E-267	ependymal	Tsc22d1
8,56E-265	0,785143517	0,592	0,142	2,31E-260	ependymal	Ctnna1
7,34E-264	1,773749608	0,948	0,654	1,98E-259	ependymal	Wdr17
2,97E-263	0,748115318	0,587	0,135	8,01E-259	ependymal	Cobll1
1,72E-262	1,1523117	0,752	0,271	4,62E-258	ependymal	Abhd2
4,70E-261	0,785181726	0,603	0,146	1,27E-256	ependymal	Pdlim5
3,53E-252	0,667417119	0,507	0,113	9,51E-248	ependymal	Acad8
6,52E-250	0,712897247	0,515	0,119	1,76E-245	ependymal	Tmem237

1,57E-245	1,142250268	0,789	0,29	4,23E-241	ependymal	Ror1
5,08E-244	0,92803014	0,604	0,155	1,37E-239	ependymal	9530026P05Rik
1,20E-243	0,954519782	0,732	0,239	3,23E-239	ependymal	Cachd1
2,30E-243	1,425603908	0,958	0,729	6,21E-239	ependymal	Itpr1
7,38E-243	1,142137577	0,801	0,313	1,99E-238	ependymal	Slc12a2
1,19E-242	1,177872445	0,892	0,402	3,21E-238	ependymal	Nckap5
5,48E-240	1,03680803	0,838	0,321	1,48E-235	ependymal	Clmn
1,91E-237	1,105812498	0,913	0,404	5,15E-233	ependymal	Pitpnc1
6,51E-237	0,777292077	0,532	0,13	1,75E-232	ependymal	Atp7a
5,53E-235	0,596501841	0,52	0,116	1,49E-230	ependymal	Vegfa
1,86E-232	1,251902246	0,838	0,378	5,02E-228	ependymal	Mapk4
6,85E-231	1,341375224	0,904	0,494	1,84E-226	ependymal	Pid1
7,33E-231	0,938501284	0,687	0,225	1,97E-226	ependymal	Atp11c
7,10E-229	0,807587206	0,576	0,158	1,91E-224	ependymal	Cnst
6,40E-221	0,655408136	0,534	0,129	1,73E-216	ependymal	Myo1e
3,55E-220	0,743623064	0,549	0,142	9,56E-216	ependymal	Gm5089
4,58E-218	1,235209785	0,813	0,406	1,23E-213	ependymal	Slc23a2
4,67E-215	1,1242151	0,861	0,49	1,26E-210	ependymal	Slc22a17
8,43E-215	1,168916266	0,944	0,64	2,27E-210	ependymal	Wwox
1,45E-214	0,875579505	0,752	0,276	3,89E-210	ependymal	Psap
7,92E-214	0,834189808	0,642	0,2	2,13E-209	ependymal	Sgk3
1,34E-213	1,111919879	0,987	0,92	3,62E-209	ependymal	Exoc4
1,24E-212	1,193914473	0,861	0,485	3,34E-208	ependymal	Pcnx
7,77E-208	0,653498946	0,535	0,14	2,09E-203	ependymal	Prtg
3,59E-203	0,705154515	0,507	0,13	9,66E-199	ependymal	Tgfb2
3,27E-202	1,285998182	0,906	0,577	8,80E-198	ependymal	Naaladl2
4,21E-197	1,124064607	0,97	0,556	1,13E-192	ependymal	Maml2
2,61E-196	0,798462135	0,634	0,209	7,02E-192	ependymal	Cdc14b
4,85E-194	1,299984829	0,942	0,708	1,31E-189	ependymal	Tmem108
1,49E-193	0,718136717	0,701	0,228	4,01E-189	ependymal	Utrn
3,01E-188	0,930708978	0,863	0,424	8,11E-184	ependymal	Pacrg
5,99E-181	0,993709502	0,78	0,362	1,61E-176	ependymal	Stim2
9,82E-180	0,689307153	0,573	0,172	2,65E-175	ependymal	Wls
1,99E-179	0,669196661	0,908	0,386	5,35E-175	ependymal	Erbb4
2,64E-175	0,718540643	0,558	0,179	7,12E-171	ependymal	Gas6
4,37E-173	0,78324386	0,673	0,256	1,18E-168	ependymal	St5
7,26E-173	1,187065973	0,773	0,324	1,96E-168	ependymal	Cmss1
5,91E-172	1,036595696	0,638	0,25	1,59E-167	ependymal	Brwd3
5,23E-170	1,058218743	0,777	0,386	1,41E-165	ependymal	Kcnh1
4,20E-169	1,157312572	0,897	0,619	1,13E-164	ependymal	Pip5k1b
6,99E-166	0,625579684	0,528	0,154	1,88E-161	ependymal	Ston2
1,43E-165	0,97024084	0,908	0,635	3,85E-161	ependymal	Shank2
2,99E-164	1,212340939	0,985	0,95	8,06E-160	ependymal	Frmpd4

2,05E-163	0,575236859	0,538	0,158	5,51E-159	ependymal	Myo10
1,08E-162	0,849146432	0,785	0,385	2,90E-158	ependymal	4632427E13Rik
1,95E-162	0,896555731	0,754	0,348	5,25E-158	ependymal	Wwc1
1,03E-160	0,836024705	0,649	0,26	2,76E-156	ependymal	Larp1b
1,16E-160	0,78439724	0,592	0,202	3,13E-156	ependymal	Lhfp
1,92E-158	0,60621649	0,537	0,17	5,17E-154	ependymal	Nt5dc1
5,63E-156	0,639852002	0,592	0,198	1,52E-151	ependymal	Tmem164
4,36E-155	0,826165111	0,985	0,893	1,17E-150	ependymal	Ddx5
1,24E-153	0,796529663	0,68	0,285	3,35E-149	ependymal	Wdpcp
2,56E-152	0,674608838	0,593	0,213	6,89E-148	ependymal	Ccdc191
1,04E-151	0,74065268	0,773	0,324	2,80E-147	ependymal	Camk2d
1,48E-151	0,564481438	0,707	0,247	3,98E-147	ependymal	Ptprm
1,69E-151	0,672021793	0,606	0,218	4,55E-147	ependymal	Cuedc1
4,23E-151	0,908893446	0,842	0,507	1,14E-146	ependymal	Fars2
8,81E-151	0,886633924	0,755	0,364	2,37E-146	ependymal	Sntb2
1,59E-150	0,869595898	0,958	0,643	4,28E-146	ependymal	Rfx3
9,60E-150	0,746310268	0,569	0,202	2,59E-145	ependymal	Myo5b
1,09E-149	0,855539717	0,8	0,422	2,93E-145	ependymal	Ttll5
9,54E-149	0,920798732	0,954	0,65	2,57E-144	ependymal	Plcl1
1,62E-148	0,734280951	0,556	0,197	4,36E-144	ependymal	Grb10
1,83E-147	0,842505653	0,724	0,339	4,94E-143	ependymal	Ccdc30
2,50E-140	0,912991406	0,755	0,37	6,74E-136	ependymal	Fhit
2,61E-138	0,56676811	0,586	0,199	7,03E-134	ependymal	Cux2
4,49E-135	0,812074984	0,701	0,327	1,21E-130	ependymal	Cpeb2
5,47E-134	0,568382434	0,582	0,213	1,47E-129	ependymal	Srsf2
1,09E-133	0,611408958	0,569	0,211	2,94E-129	ependymal	Gtf2ird1
1,96E-133	0,615176675	0,511	0,172	5,29E-129	ependymal	Bcl2
7,52E-132	0,81148985	0,683	0,301	2,03E-127	ependymal	Dnah9
4,40E-131	0,744292073	0,693	0,331	1,18E-126	ependymal	Fndc3b
2,48E-128	0,556312529	0,556	0,199	6,67E-124	ependymal	Rrbp1
3,74E-128	0,788243824	0,945	0,802	1,01E-123	ependymal	Rsrp1
6,25E-128	0,592649276	0,517	0,181	1,68E-123	ependymal	Plekha7
7,23E-128	0,658748603	0,624	0,26	1,95E-123	ependymal	Pced1b
1,50E-127	0,520157738	0,665	0,241	4,05E-123	ependymal	Dock1
9,26E-124	0,691578926	0,979	0,727	2,50E-119	ependymal	Nfia
1,97E-123	0,696835082	0,662	0,301	5,30E-119	ependymal	Stim1
1,84E-122	0,592858705	0,531	0,202	4,95E-118	ependymal	Snhg20
1,98E-122	0,66965013	0,597	0,253	5,33E-118	ependymal	Mapk9
9,97E-122	0,635842297	0,608	0,239	2,69E-117	ependymal	Plcb4
2,84E-121	0,683901846	0,996	0,916	7,66E-117	ependymal	Rora
1,35E-120	0,587572627	0,554	0,21	3,62E-116	ependymal	Spats2l
5,51E-119	0,985582834	0,942	0,743	1,48E-114	ependymal	Etl4
3,53E-117	0,654075429	0,603	0,259	9,51E-113	ependymal	Tbcel

3,70E-117	0,68961488	0,715	0,372	9,97E-113	ependymal	D430042O09Rik
1,37E-116	0,618625101	0,645	0,251	3,70E-112	ependymal	Pdzrn3
2,05E-111	0,531814155	0,518	0,195	5,51E-107	ependymal	R3hcc1l
4,74E-111	0,733785915	0,904	0,746	1,28E-106	ependymal	Ehbp1
8,07E-110	0,52384316	0,559	0,215	2,17E-105	ependymal	Cald1
1,71E-108	0,681348359	0,654	0,296	4,61E-104	ependymal	Cdh18
7,55E-108	0,672346098	0,939	0,763	2,03E-103	ependymal	Stox2
8,18E-108	0,602425119	0,589	0,238	2,20E-103	ependymal	Ccbe1
2,42E-107	0,809114828	0,692	0,382	6,51E-103	ependymal	Myrip
1,01E-106	0,581215129	0,52	0,207	2,72E-102	ependymal	Crocc
1,33E-106	0,505780505	0,53	0,197	3,57E-102	ependymal	Sytl2
4,03E-104	0,527013064	0,518	0,198	1,09E-99	ependymal	Gm48742
2,04E-103	0,51409558	0,5	0,192	5,48E-99	ependymal	Kiz
4,53E-103	0,526392369	0,503	0,188	1,22E-98	ependymal	Prdm5
4,74E-103	0,764784222	0,737	0,422	1,28E-98	ependymal	Arid5b
4,96E-103	0,59057854	0,554	0,239	1,34E-98	ependymal	Cep162
1,13E-99	0,662694909	0,932	0,783	3,03E-95	ependymal	Tcf12
3,10E-99	1,087210558	0,861	0,519	8,34E-95	ependymal	Gm42418
1,09E-98	0,659004776	0,721	0,399	2,95E-94	ependymal	Luzp1
1,26E-98	0,768021114	0,72	0,407	3,40E-94	ependymal	Aff2
1,68E-98	0,570115247	0,852	0,5	4,52E-94	ependymal	Trps1
2,29E-98	0,698547056	0,835	0,616	6,17E-94	ependymal	Srsf5
2,50E-98	0,651975628	0,67	0,341	6,75E-94	ependymal	Peak1
6,55E-98	0,610303528	0,573	0,268	1,77E-93	ependymal	Nufip2
1,82E-97	0,53092769	0,524	0,221	4,90E-93	ependymal	Ubxn11
1,06E-96	0,683937204	0,989	0,938	2,85E-92	ependymal	Kcnq1ot1
4,88E-96	0,687810898	0,785	0,518	1,31E-91	ependymal	Sgms1
1,64E-95	0,633868252	0,804	0,514	4,42E-91	ependymal	Daam1
3,24E-95	0,658282723	0,862	0,631	8,74E-91	ependymal	Nedd4
2,69E-93	0,516877769	0,782	0,384	7,25E-89	ependymal	Zfp536
3,11E-93	0,61258034	0,668	0,345	8,39E-89	ependymal	Phkb
7,04E-93	0,559472332	0,62	0,299	1,90E-88	ependymal	Fam184a
1,36E-92	0,936026452	0,852	0,62	3,65E-88	ependymal	Car10
4,68E-92	0,687077645	0,839	0,627	1,26E-87	ependymal	Pcca
5,11E-92	0,608940341	0,746	0,432	1,38E-87	ependymal	Fgd4
1,46E-91	0,525472685	0,525	0,228	3,94E-87	ependymal	Arhgef18
1,86E-91	0,521808892	0,506	0,209	5,02E-87	ependymal	Itpk1
1,15E-90	0,629507511	0,939	0,723	3,10E-86	ependymal	Mir100hg
1,32E-90	0,515014664	0,53	0,231	3,57E-86	ependymal	Ift74
3,37E-90	0,582859366	0,658	0,346	9,09E-86	ependymal	Fndc3a
5,54E-90	0,668965575	0,687	0,374	1,49E-85	ependymal	Tmtc1
6,91E-90	0,584133631	0,58	0,263	1,86E-85	ependymal	Zfhx4
1,23E-89	0,539441912	0,585	0,268	3,32E-85	ependymal	Zfand6

1,73E-89	0,537620521	0,645	0,32	4,65E-85	ependymal	Firre
7,48E-88	0,525472732	0,61	0,303	2,01E-83	ependymal	Srsf1
9,49E-88	0,551502851	0,57	0,273	2,56E-83	ependymal	Srsf10
8,01E-87	0,605006553	0,631	0,323	2,16E-82	ependymal	Fam110b
5,50E-85	0,611801558	0,807	0,514	1,48E-80	ependymal	Ablim1
4,77E-84	0,61370079	0,735	0,439	1,28E-79	ependymal	Armc9
8,99E-84	0,585274672	0,513	0,233	2,42E-79	ependymal	Mamld1
9,26E-84	0,558378687	0,587	0,291	2,50E-79	ependymal	Pikfyve
2,60E-83	0,533868505	0,563	0,271	7,01E-79	ependymal	Zkscan3
4,55E-82	0,54825805	0,665	0,362	1,23E-77	ependymal	Tnrc18
5,22E-82	0,502690606	0,908	0,731	1,40E-77	ependymal	Ahcyl2
1,39E-80	0,581137213	0,93	0,842	3,74E-76	ependymal	Ppp1r9a
2,01E-80	0,532552319	0,62	0,315	5,42E-76	ependymal	Znrf3
4,37E-79	0,611197297	0,789	0,555	1,18E-74	ependymal	Mtdh
2,26E-78	0,506779902	0,583	0,28	6,09E-74	ependymal	Myo1d
4,24E-78	0,551892975	0,599	0,313	1,14E-73	ependymal	Fsd1l
4,35E-78	0,573803336	0,755	0,472	1,17E-73	ependymal	Dtx3
1,03E-77	0,52542279	0,607	0,309	2,78E-73	ependymal	Tmem245
2,61E-77	0,601873994	0,735	0,46	7,04E-73	ependymal	5031425E22Rik
7,33E-76	0,551902943	0,673	0,393	1,97E-71	ependymal	Lrp6
1,41E-75	0,531891259	0,582	0,296	3,80E-71	ependymal	Ppargc1a
4,25E-75	0,523531911	0,977	0,947	1,14E-70	ependymal	Snrnp70
4,41E-75	0,579357157	0,906	0,795	1,19E-70	ependymal	Aopep
7,62E-75	0,574932641	0,634	0,294	2,05E-70	ependymal	Grm8
7,70E-75	0,538119774	0,627	0,353	2,07E-70	ependymal	Hnrnph1
9,80E-74	0,500162995	0,639	0,326	2,64E-69	ependymal	Csgalnact1
1,77E-73	0,529474081	0,542	0,27	4,77E-69	ependymal	Tgfbr3
2,01E-72	0,531031069	0,997	0,812	5,43E-68	ependymal	Zbtb20
2,54E-72	0,635812246	0,689	0,399	6,84E-68	ependymal	Flrt2
8,10E-72	0,609504671	0,846	0,697	2,18E-67	ependymal	Setd5
2,41E-71	0,573906962	0,755	0,5	6,49E-67	ependymal	Dock7
6,03E-71	0,558700592	0,818	0,594	1,62E-66	ependymal	Igf1r
2,01E-70	0,518352508	0,57	0,303	5,42E-66	ependymal	Cnnm2
3,57E-70	0,542392166	0,587	0,319	9,62E-66	ependymal	Nfyc
9,86E-70	0,55434306	0,701	0,45	2,66E-65	ependymal	Gnas
8,54E-69	0,51714139	0,963	0,906	2,30E-64	ependymal	Prpf4b
3,78E-68	0,556697301	0,732	0,479	1,02E-63	ependymal	Dync2h1
3,64E-67	0,582696338	0,849	0,68	9,79E-63	ependymal	Fmnl2
4,88E-67	0,575035567	0,903	0,702	1,31E-62	ependymal	Magi1
9,33E-66	0,652099409	0,646	0,377	2,51E-61	ependymal	Cdk8
1,16E-65	0,50063946	0,514	0,261	3,13E-61	ependymal	Miga1
2,18E-65	0,747415992	0,59	0,325	5,86E-61	ependymal	Slit2
7,75E-64	0,517575458	0,858	0,696	2,09E-59	ependymal	Pabpn1

6,49E-62	0,556248673	0,713	0,477	1,75E-57	ependymal	Akap9
9,09E-62	0,53110497	0,77	0,566	2,45E-57	ependymal	Chchd3
1,87E-61	0,507177059	0,855	0,709	5,03E-57	ependymal	Chd6
3,46E-61	0,778961956	0,972	0,932	9,32E-57	ependymal	Syne1
1,49E-57	0,551717181	0,938	0,884	4,02E-53	ependymal	Gpm6a
1,95E-57	0,517430277	0,672	0,41	5,26E-53	ependymal	Sh3rf1
3,98E-54	0,532720657	0,632	0,406	1,07E-49	ependymal	Atp1b1
5,31E-42	0,511316821	0,811	0,69	1,43E-37	ependymal	Zfp385b
9,31E-41	0,578748336	0,779	0,678	2,51E-36	ependymal	Immp2l
5,82E-39	0,614805934	0,527	0,317	1,57E-34	ependymal	Pdzrn4
0	4,148781637	1	0,097	0	Cd274_Neuroimmune	Reln
0	2,710976315	0,959	0,114	0	Cd274_Neuroimmune	Dach1
0	2,612830745	0,9	0,026	0	Cd274_Neuroimmune	Ndnf
0	2,160547478	0,952	0,228	0	Cd274_Neuroimmune	Utrn
0	1,932017524	0,878	0,127	0	Cd274_Neuroimmune	Tmem163
0	1,861133728	0,859	0,159	0	Cd274_Neuroimmune	Cdh4
0	1,839029697	0,787	0,016	0	Cd274_Neuroimmune	Trp73
0	1,826394017	0,804	0,129	0	Cd274_Neuroimmune	Hs3st5
0	1,737682178	0,809	0,078	0	Cd274_Neuroimmune	Cacna2d2
0	1,438610103	0,691	0,011	0	Cd274_Neuroimmune	Ebf3
0	1,400829863	0,611	0,031	0	Cd274_Neuroimmune	Gm29683
0	1,07633036	0,557	0,031	0	Cd274_Neuroimmune	Eln
3,14E-301	1,024615579	0,517	0,074	8,46E-297	Cd274_Neuroimmune	B130024G19Rik
1,11E-296	2,341091852	0,987	0,452	2,99E-292	Cd274_Neuroimmune	Thsd7b
1,48E-261	1,869946582	0,943	0,39	3,97E-257	Cd274_Neuroimmune	Maml3
7,80E-255	1,939629419	1	0,831	2,10E-250	Cd274_Neuroimmune	Fat3
1,25E-253	2,059763036	1	0,909	3,38E-249	Cd274_Neuroimmune	Kcnb2
1,66E-253	1,328010838	0,717	0,181	4,48E-249	Cd274_Neuroimmune	Plekha7
1,25E-252	1,545097347	0,922	0,31	3,37E-248	Cd274_Neuroimmune	Rbms3
5,82E-244	1,827659686	0,935	0,391	1,57E-239	Cd274_Neuroimmune	Pdzd2
1,28E-243	2,097980277	1	0,885	3,45E-239	Cd274_Neuroimmune	Robo2
2,45E-228	1,777565553	1	0,901	6,61E-224	Cd274_Neuroimmune	Clstn2
3,88E-218	1,646842006	0,989	0,577	1,05E-213	Cd274_Neuroimmune	Msi2
2,13E-216	0,986612995	0,526	0,102	5,75E-212	Cd274_Neuroimmune	Pou6f2
5,60E-213	2,013072534	1	0,884	1,51E-208	Cd274_Neuroimmune	Cntnap2
3,21E-212	1,436802318	1	0,95	8,66E-208	Cd274_Neuroimmune	Nav2
5,51E-210	1,909129734	0,998	0,796	1,48E-205	Cd274_Neuroimmune	Kcnh7
3,67E-188	1,361656322	0,809	0,332	9,90E-184	Cd274_Neuroimmune	Fndc3b
2,78E-181	1,149327045	0,589	0,142	7,49E-177	Cd274_Neuroimmune	Kcnip1
2,70E-176	0,982099543	0,5	0,106	7,26E-172	Cd274_Neuroimmune	Angpt1
1,92E-167	1,276730937	0,987	0,84	5,18E-163	Cd274_Neuroimmune	Dync1i1
1,45E-163	1,298876001	0,726	0,266	3,91E-159	Cd274_Neuroimmune	Rbms1

3,24E-161	1,384319383	0,972	0,62	8,73E-157	Cd274_Neuroimmune	Car10
7,87E-157	1,282716017	0,804	0,357	2,12E-152	Cd274_Neuroimmune	Cep112
8,87E-152	1,155814204	0,998	0,947	2,39E-147	Cd274_Neuroimmune	B3galt1
3,12E-147	1,180137268	0,998	0,907	8,41E-143	Cd274_Neuroimmune	Cacna2d1
2,40E-138	1,251866018	0,989	0,75	6,47E-134	Cd274_Neuroimmune	Nrg1
4,20E-137	0,903896126	0,967	0,52	1,13E-132	Cd274_Neuroimmune	Gm42418
9,51E-134	1,189040577	0,998	0,907	2,56E-129	Cd274_Neuroimmune	Snhg11
1,94E-125	1,015778781	0,991	0,897	5,24E-121	Cd274_Neuroimmune	Nfib
3,54E-120	1,061580107	0,789	0,431	9,53E-116	Cd274_Neuroimmune	Ralgps2
8,27E-118	1,021420392	0,85	0,432	2,23E-113	Cd274_Neuroimmune	Kcnc2
1,20E-115	0,786779814	1	0,991	3,22E-111	Cd274_Neuroimmune	Nrxn1
3,81E-115	0,951295265	1	0,98	1,03E-110	Cd274_Neuroimmune	Fam155a
1,62E-113	1,088276967	0,746	0,37	4,36E-109	Cd274_Neuroimmune	Fmn1
6,98E-112	0,817538184	1	0,989	1,88E-107	Cd274_Neuroimmune	Lsamp
6,66E-108	0,777020755	1	0,99	1,79E-103	Cd274_Neuroimmune	Ank3
2,22E-107	0,944159187	0,626	0,266	5,98E-103	Cd274_Neuroimmune	Pcbp3
6,92E-107	1,133816721	0,809	0,468	1,86E-102	Cd274_Neuroimmune	Epha3
4,82E-106	1,007150023	1	0,861	1,30E-101	Cd274_Neuroimmune	Lingo2
2,75E-99	0,973775898	0,983	0,813	7,40E-95	Cd274_Neuroimmune	Tenm4
1,43E-98	0,925403276	0,889	0,655	3,86E-94	Cd274_Neuroimmune	Slc2a13
4,44E-98	0,932788129	0,983	0,9	1,19E-93	Cd274_Neuroimmune	Srgap3
1,45E-94	1,091619697	0,711	0,366	3,91E-90	Cd274_Neuroimmune	Adcy8
1,04E-90	0,903477826	0,974	0,857	2,80E-86	Cd274_Neuroimmune	Gpr158
4,01E-90	0,881701924	0,937	0,666	1,08E-85	Cd274_Neuroimmune	Astn2
6,74E-85	0,870650237	0,976	0,795	1,81E-80	Cd274_Neuroimmune	Kcnq5
5,52E-83	0,731454196	0,928	0,672	1,49E-78	Cd274_Neuroimmune	Dnm3
1,25E-79	0,806420982	0,987	0,847	3,37E-75	Cd274_Neuroimmune	Dscaml1
2,53E-76	0,754824276	1	0,982	6,81E-72	Cd274_Neuroimmune	Plcb1
8,65E-75	0,682827906	0,989	0,734	2,33E-70	Cd274_Neuroimmune	Cntn5
3,21E-74	0,952594342	0,661	0,379	8,66E-70	Cd274_Neuroimmune	5330417C22Rik
3,82E-73	0,7441705	0,55	0,239	1,03E-68	Cd274_Neuroimmune	Mtss1
3,60E-71	0,702732511	0,943	0,699	9,70E-67	Cd274_Neuroimmune	Gria4
6,06E-71	0,738099034	0,943	0,847	1,63E-66	Cd274_Neuroimmune	Ncoa1
7,84E-71	0,720831876	0,983	0,938	2,11E-66	Cd274_Neuroimmune	Kcnq1ot1
1,97E-65	0,652981732	0,611	0,282	5,31E-61	Cd274_Neuroimmune	Cit
3,52E-65	0,666596418	0,987	0,945	9,49E-61	Cd274_Neuroimmune	Sgip1
2,20E-64	0,616611243	0,998	0,988	5,92E-60	Cd274_Neuroimmune	Ctnna2
4,87E-64	0,638395675	0,976	0,883	1,31E-59	Cd274_Neuroimmune	Gpm6a
2,77E-61	0,731733534	0,841	0,717	7,47E-57	Cd274_Neuroimmune	Gucy1a2
1,37E-59	0,555954967	0,97	0,881	3,69E-55	Cd274_Neuroimmune	Ntrk2
3,89E-58	0,785012914	0,663	0,417	1,05E-53	Cd274_Neuroimmune	Ripor2
4,95E-58	0,667368323	0,83	0,707	1,33E-53	Cd274_Neuroimmune	Ubr5
5,29E-58	0,65661167	0,907	0,789	1,42E-53	Cd274_Neuroimmune	A230057D06Rik

3,29E-57	0,600282452	0,983	0,894	8,86E-53	Cd274_Neuroimmune	Ddx5
3,22E-56	0,73747683	0,774	0,527	8,67E-52	Cd274_Neuroimmune	Slc35f1
5,03E-55	0,739045824	0,624	0,394	1,35E-50	Cd274_Neuroimmune	Hdgfl3
1,65E-54	0,610186862	1	0,989	4,43E-50	Cd274_Neuroimmune	Mdga2
3,91E-54	0,528710306	0,993	0,956	1,05E-49	Cd274_Neuroimmune	Ttc3
2,33E-53	0,597251479	0,946	0,859	6,29E-49	Cd274_Neuroimmune	Ptprg
6,04E-53	0,703783314	0,598	0,329	1,63E-48	Cd274_Neuroimmune	Kcnk2
7,81E-51	0,536555857	0,993	0,946	2,10E-46	Cd274_Neuroimmune	Cacna1a
6,43E-49	0,692124331	0,807	0,688	1,73E-44	Cd274_Neuroimmune	Ralgapa2
1,09E-48	0,712656507	0,896	0,717	2,93E-44	Cd274_Neuroimmune	Nkain3
2,15E-48	0,516903059	0,957	0,885	5,80E-44	Cd274_Neuroimmune	Plekha5
9,09E-45	0,64857132	0,607	0,377	2,45E-40	Cd274_Neuroimmune	Pknox2
3,03E-44	0,665584585	0,709	0,516	8,16E-40	Cd274_Neuroimmune	Ablim1
3,47E-44	0,604315132	0,776	0,692	9,34E-40	Cd274_Neuroimmune	Ankrd12
8,55E-43	0,727038065	0,874	0,759	2,30E-38	Cd274_Neuroimmune	Slc24a3
6,90E-37	0,554374397	0,526	0,298	1,86E-32	Cd274_Neuroimmune	Zmiz1
2,26E-36	0,520115101	0,846	0,741	6,09E-32	Cd274_Neuroimmune	Bcl11a
4,87E-33	0,521821701	0,724	0,504	1,31E-28	Cd274_Neuroimmune	Frmd4b
4,89E-32	0,528886679	0,5	0,298	1,32E-27	Cd274_Neuroimmune	Elavl4
1,54E-30	0,514393558	0,661	0,516	4,15E-26	Cd274_Neuroimmune	Daam1
4,96E-26	0,510191651	0,535	0,391	1,34E-21	Cd274_Neuroimmune	Ppp2r2c
2,19E-23	0,511313216	0,611	0,493	5,89E-19	Cd274_Neuroimmune	A330076H08Rik
2,74E-23	0,575063456	0,548	0,408	7,37E-19	Cd274_Neuroimmune	Aff2

	**Blood**						**Bone Marrow**						**Lymph Node**						**Spleen**					
**(% of total live CD45+ cells)**	WT	Het	Test	p-value	FDR	d	WT	Het	Test	p-value	FDR	d	WT	Het	Test	p-value	FDR	d	WT	Het	Test	p-value	FDR	d
Total B cells	42,23 ± 10,15	37,86 ± 9,86	t-test	0,2341	0,7123	-0,44	18,47 ± 7,28	15,27 ± 3,72	Wcc	0,0636	0,7952	-0,56	41,41 ± 5,06	36,69 ± 5,53	t-test	0,0238	0,2979	-0,89	55,45 ± 3,75	53,83 ± 2,05	Welch	0,1658	0,6907	-0,55
Total CD4+ T-cells	13,03 ± 2,15	12,83 ± 2,10	t-test	0,7964	0,9765	-0,09	1,21 ± 0,98	1,09 ± 0,62	Wcc	0,9131	0,9720	-0,14	28,60 ± 1,96	31,65 ± 2,41	t-test	0,0009	**0,0230**	1,38	17,98 ± 2,80	17,46 ± 1,96	Wcc	0,5194	0,8943	-0,22
Total CD8+ T-cells	13,21 ± 3,36	13,38 ± 3,11	Wcc	0,5532	0,8779	0,05	0,90 ± 0,51	0,89 ± 0,33	Wcc	0,9304	0,9720	-0,02	21,51 ± 2,67	23,34 ± 3,23	t-test	0,1086	0,4732	0,62	12,07 ± 2,48	12,07 ± 1,68	Wcc	0,7870	0,8943	0,00
Total NK cells	7,05 ± 2,43	7,24 ± 2,07	t-test	0,8203	0,9765	0,08	1,90 ± 0,33	1,70 ± 0,39	Wcc	0,1494	0,9720	-0,57	1,17 ± 0,13	1,10 ± 0,12	t-test	0,1280	0,4732	-0,58	3,50 ± 0,50	4,20 ± 0,55	t-test	0,0011	**0,0275**	1,33
Total Granulocytes	13,63 ± 2,51	17,33 ± 3,64	t-test	0,0028	0,0699	1,17	49,80 ± 8,21	54,16 ± 3,44	Wcc	0,0275	0,6881	0,70	0,81 ± 0,31	0,76 ± 0,30	t-test	0,6633	0,7311	-0,16	4,38 ± 1,54	5,46 ± 1,63	t-test	0,0736	0,6131	0,68
Total Monocytes	7,11 ± 2,02	7,88 ± 1,82	t-test	0,2759	0,7123	0,40	11,39 ± 3,40	10,27 ± 1,29	Wcc	0,3947	0,9720	-0,44	1,11 ± 0,51	0,87 ± 0,37	t-test	0,1680	0,5251	-0,53	1,46 ± 0,57	1,94 ± 0,74	t-test	0,0603	0,6131	0,72
Total CD11b+ FcR+ Myeloid cells	1,69 ± 0,78	1,46 ± 0,56	t-test	0,3478	0,7246	-0,34	13,80 ± 3,72	14,23 ± 2,65	Wcc	0,8272	0,9720	0,13	1,75 ± 0,54	1,99 ± 0,62	Wcc	0,4069	0,6334	0,40	1,84 ± 0,42	1,73 ± 0,50	Wcc	0,1342	0,6710	-0,24
**B cells (% of B cells)**																								
CD138+ B cells	6,26 ± 1,56	5,14 ± 1,21	Wcc	0,0418	0,5222	-0,80	16,59 ± 6,28	15,26 ± 4,14	Wcc	0,8443	0,9720	-0,25	2,01 ± 1,17	1,49 ± 1,13	Wcc	0,2386	0,5491	-0,45	1,33 ± 0,63	1,32 ± 0,52	t-test	0,9636	0,9636	-0,02
CD27-/CD138low B cells	91,47 ± 1,73	92,33 ± 1,18	t-test	0,1157	0,7123	0,58	70,59 ± 6,28	71,43 ± 4,11	t-test	0,6706	0,9720	0,16	92,84 ± 2,30	92,98 ± 2,53	t-test	0,8801	0,8801	0,06	89,62 ± 4,01	89,49 ± 3,37	Wcc	0,6473	0,8943	-0,03
CD27+/CD138low B cells	2,15 ± 0,34	2,43 ± 0,52	Wcc	0,1007	0,7123	0,61	12,64 ± 3,80	13,02 ± 3,95	t-test	0,7935	0,9720	0,10	4,99 ± 1,37	5,34 ± 1,72	t-test	0,5539	0,6924	0,22	8,89 ± 4,06	9,05 ± 3,17	Wcc	0,5467	0,8943	0,04
**CD4+ T-cells (% of CD4+ T-cells)**																								
CD44-/CD62L+ Naive CD4+ T-cells	63,42 ± 5,28	62,91 ± 4,19	Wcc	0,6073	0,8930	-0,11	14,05 ± 6,03	17,20 ± 7,57	Wcc	0,2301	0,9720	0,46	73,30 ± 2,54	75,52 ± 3,34	t-test	0,0553	0,3613	0,74	41,94 ± 11,21	45,29 ± 10,49	t-test	0,4050	0,8943	0,31
CD44+/CD62L+ Central Memory CD4+ T-cells	3,65 ± 1,41	4,05 ± 1,61	Wcc	0,2948	0,7123	0,27	10,05 ± 4,61	8,52 ± 2,67	t-test	0,2787	0,9720	-0,41	7,98 ± 1,28	7,65 ± 1,15	t-test	0,4814	0,6334	-0,27	9,45 ± 3,09	9,80 ± 2,65	t-test	0,7429	0,8943	0,12
CD44+/CD62L- Effector (Memory) CD4+ T-cells	20,44 ± 3,70	19,94 ± 2,78	t-test	0,6748	0,9372	-0,15	72,59 ± 8,83	71,27 ± 7,93	t-test	0,6748	0,9720	-0,16	14,53 ± 1,26	13,01 ± 2,62	Welch	0,0578	0,3613	-0,73	36,33 ± 7,01	33,52 ± 6,84	Wcc	0,2360	0,7375	-0,41
CD44-/CD62L- CD4+ T-cells	12,48 ± 2,40	13,11 ± 2,44	t-test	0,4799	0,8779	0,26	3,29 ± 2,27	3,02 ± 2,73	t-test	0,7732	0,9720	-0,11	4,18 ± 1,36	3,82 ± 1,02	t-test	0,4232	0,6334	-0,30	12,28 ± 7,26	11,42 ± 6,14	t-test	0,7273	0,8943	-0,13
**CD8+ T-cells (% of CD8+ T-cells)**																								
CD44-/CD62L+ Naive CD8+ T-cells	65,25 ± 4,49	65,48 ± 4,97	t-test	0,8946	0,9842	0,05	42,24 ± 9,72	41,84 ± 8,36	t-test	0,9054	0,9720	-0,04	83,57 ± 2,08	84,87 ± 3,20	t-test	0,2084	0,5491	0,48	68,63 ± 7,77	68,52 ± 5,70	Wcc	0,5743	0,8943	-0,02
CD44+/CD62L+ Central Memory CD8+ T-cells	18,80 ± 4,22	19,13 ± 4,58	Wcc	0,9842	0,9842	0,07	34,70 ± 7,27	33,29 ± 4,73	t-test	0,5393	0,9720	-0,23	12,08 ± 2,22	11,30 ± 2,67	t-test	0,4000	0,6334	-0,32	18,83 ± 4,10	20,47 ± 5,67	t-test	0,3765	0,8943	0,33
CD44+/CD62L- Effector (Memory) CD8+ T-cells	9,12 ± 2,51	8,01 ± 2,84	Wcc	0,3134	0,7123	-0,41	20,53 ± 7,25	22,56 ± 7,37	t-test	0,4610	0,9720	0,28	2,53 ± 0,56	2,30 ± 0,96	t-test	0,4318	0,6334	-0,30	6,09 ± 2,26	6,03 ± 2,83	Wcc	0,5747	0,8943	-0,02
CD44-/CD62L- CD8+ T-cells	6,81 ± 1,74	7,38 ± 2,50	Wcc	0,9527	0,9842	0,26	2,52 ± 2,35	2,29 ± 1,92	t-test	0,7730	0,9720	-0,11	1,83 ± 0,76	1,52 ± 0,69	t-test	0,2524	0,5491	-0,43	6,45 ± 5,53	4,98 ± 4,13	Wcc	0,5467	0,8943	-0,30
**NK cells (% of NK cells)**																								
CD27-/CD62L+	65,17 ± 4,92	63,38 ± 4,39	t-test	0,2929	0,7123	-0,38	10,06 ± 2,71	10,56 ± 2,69	t-test	0,6191	0,9720	0,19	28,22 ± 3,87	30,65 ± 4,52	t-test	0,1325	0,4732	0,58	35,93 ± 5,31	36,58 ± 5,79	t-test	0,7514	0,8943	0,12
CD27+/CD62L+	17,57 ± 4,29	19,26 ± 4,30	t-test	0,2848	0,7123	0,39	66,94 ± 4,93	66,19 ± 4,67	t-test	0,6777	0,9720	-0,16	51,38 ± 6,01	49,01 ± 8,02	t-test	0,3794	0,6334	-0,33	27,20 ± 7,02	27,89 ± 5,64	t-test	0,7663	0,8943	0,11
CD27+/CD62L-	4,55 ± 1,60	5,16 ± 1,11	t-test	0,2254	0,7123	0,45	20,09 ± 4,39	20,15 ± 3,78	t-test	0,9720	0,9720	0,01	13,46 ± 3,83	12,87 ± 3,58	t-test	0,6726	0,7311	-0,16	23,35 ± 6,35	23,18 ± 3,66	Welch	0,9285	0,9636	-0,03
CD27-/CD62L-	12,73 ± 5,55	12,22 ± 2,22	Welch	0,7428	0,9765	-0,12	2,92 ± 1,27	3,11 ± 1,86	Wcc	0,9652	0,9720	0,12	6,95 ± 4,34	7,45 ± 4,82	Wcc	0,7767	0,8090	0,11	13,52 ± 8,13	12,36 ± 7,72	Wcc	0,8516	0,9256	-0,15
**Monocytes (% of Monocytes)**																								
CD11b-/CD62L+	0,31 ± 0,20	0,31 ± 0,18	t-test	0,9493	0,9842	-0,02	10,54 ± 2,81	9,99 ± 1,79	t-test	0,5305	0,9720	-0,24	1,70 ± 1,47	1,19 ± 0,86	t-test	0,2636	0,5491	-0,42	1,87 ± 1,76	1,26 ± 1,20	Wcc	0,3275	0,8943	-0,41
CD11b+/CD62L-	62,81 ± 7,55	61,45 ± 5,25	t-test	0,5619	0,8779	-0,21	9,27 ± 3,03	8,77 ± 3,41	t-test	0,6777	0,9720	-0,16	16,13 ± 7,83	17,96 ± 5,54	t-test	0,4713	0,6334	0,27	40,09 ± 8,42	44,40 ± 6,15	t-test	0,1178	0,6710	0,59
CD11b+/CD62L+	36,79 ± 7,59	38,22 ± 5,22	t-test	0,5431	0,8779	0,22	79,99 ± 4,92	81,07 ± 4,01	t-test	0,5210	0,9720	0,24	81,91 ± 8,12	80,61 ± 5,88	t-test	0,6233	0,7311	-0,18	56,88 ± 7,74	53,52 ± 6,44	t-test	0,2049	0,7318	-0,47
